# Transcriptome coexpression map of human embryonic stem cells

**DOI:** 10.1186/1471-2164-7-103

**Published:** 2006-05-02

**Authors:** Huai Li, Ying Liu, Soojung Shin, Yu Sun, Jeanne F Loring, Mark P Mattson, Mahendra S Rao, Ming Zhan1

**Affiliations:** 1 Bioinformatics Unit, Branch of Research Resources, National Institute on Aging, NIH, Baltimore, MD 21224, USA; 2 Laboratory of Neurosciences, National Institute on Aging, NIH, Baltimore, MD 21224, USA; 3 The Burnham Institute, La Jolla, CA92037, USA; 4 Neurosciences Program, Johns Hopkins University School of Medicine, Baltimore, MD 21224, USA; 5 The CRL, Invitrogen Corp, 1620 Faraday Ave, Carlsbad, CA 92008, USA

## Abstract

**Background:**

Human embryonic stem (ES) cells hold great promise for medicine and science. The transcriptome of human ES cells has been studied in detail in recent years. However, no systematic analysis has yet addressed whether gene expression in human ES cells may be regulated in chromosomal domains, and no chromosomal domains of coexpression have been identified.

**Results:**

We report the first transcriptome coexpression map of the human ES cell and the earliest stage of ES differentiation, the embryoid body (EB), for the analysis of how transcriptional regulation interacts with genomic structure during ES self-renewal and differentiation. We determined the gene expression profiles from multiple ES and EB samples and identified chromosomal domains showing coexpression of adjacent genes on the genome. The coexpression domains were not random, with significant enrichment in chromosomes 8, 11, 16, 17, 19, and Y in the ES state, and 6, 11, 17, 19 and 20 in the EB state. The domains were significantly associated with Giemsa-negative bands in EB, yet showed little correlation with known cytogenetic structures in ES cells. Different patterns of coexpression were revealed by comparative transcriptome mapping between ES and EB.

**Conclusion:**

The findings and methods reported in this investigation advance our understanding of how genome organization affects gene expression in human ES cells and help to identify new mechanisms and pathways controlling ES self-renewal or differentiation.

## Background

Large-scale transcriptional profiling and the availability of the complete genome sequences have made it possible for transcriptome mapping analysis in various organisms [1]. Transcriptome maps showing the density of expressed genes along the chromosome have revealed genomic regions that correspond to known amplicons of human tumors [2-4]. Regional similarity of expression on the chromosome have been observed in the yeast *Saccharomyces cerevisiae *[1], nematode *Caenorhabditis elegans *[5], fruit fly *Drosophila melanogaster*[1,6,7], and human [2,8]. Transcriptome maps showing regional similarities illustrate the existence of chromosomal domains of gene coexpression and transcriptional regulation operating at the local chromosome level. Transcriptome mapping analyses have been based on data generated from a variety of experimental techniques, including Expressed Sequence Tags [9], Serial Analysis of Gene Expression [8], and microarray [7]. All of these studies have revealed interesting and novel patterns of transcriptome in relation to genomic organization, molecular evolution, and biological functions.

Human embryonic stem (ES) cells have the ability to differentiate into a variety of cell lineages and hold promise for drug discovery, toxicology, and replacement therapies. The embryoid body (EB) is the earliest stage of ES differentiation in culture. The transcriptome of human ES and EB cells has been studied in detail in recent years [10-16]. These studies have suggested that ES cells have an open transcriptome with few cold spots or hot spots of gene expression in the undifferentiated state and a more complex global regulation in the EB stage of differentiation. However, no systematic analysis has yet addressed whether gene expression in human ES cells may be regulated in chromosomal domains, and no chromosomal domains of coexpression have been identified. Here, we describe the first analysis of coexpression of neighboring genes on the chromosome in ES and EB cells. We determined gene expression profiles by BeadArray™ [17] and constructed transcriptome maps for both ES and EB cells. The map showed a significant pattern of gene coexpression on chromosome domains. The coexpression remained significant regardless of the effect of gene duplication. The genomic distribution of coexpression chromosomal domains was found to be non-random, with different coexpression patterns observed in ES and EB cells. The coexpression chromosome domains were biological and physiological significant. ESC – important molecular functions or biological processes were found to be enriched in the domains. The transcriptome map provided a basis to examine transcriptional regulation operating at the level of chromosomal domains in human ES cells and differential coexpression of gene clusters during the ES differentiation. The findings of this study advance our understanding of how genome organization affects gene expression and hence the self-renewal or differentiation of ES cells.

## Results

The overall goal of this study was to elucidate general coexpression patterns at the domain level in ES and EB. The coexpression profiling was based on the combination of six different cell lines representing ES or EB. Each cell line had a single sample, except I6 (2 samples). An additional sample was derived from pooled culture of different cell lines. The six cell lines and their relatedness to each other are illustrated in Supplementary Table S1 [see Additional file 6]. The cell line samples were similar to each other on the expression profiles in ES and EB, with a bit higher heterogeneity in EB than ES. The gene expression profile of each human ES cell line and its EB counterpart were determined using the high-density BeadArray™. The array contains 23,584 probes, representing 20,692 unique genes. Based on the expression data, we calculated the coexpression index for each gene in a sliding window across each chromosome. The coexpression index for a given gene was defined as the average of Pearson's correlation coefficient values of gene expression levels between this gene and every neighboring gene upstream and downstream within a certain window. The correlation of between genes was calculated from the expression values in the seven samples of ES or EB. The coexpression index, which measures the degree of coexpression among neighboring genes on the chromosome, was used in the subsequent construction and analysis of transcriptome coexpression maps in ES and EB cells.

### 1. ES and EB cells show significant coexpression patterns along the chromosome

In order to statistically examine whether genes are significantly coexpressed on the chromosome, we calculated the mean value of the coexpression index for the entire set of expressed genes on the genome. The mean coexpression index was determined for different window sizes and from two different genomic data sources: a) the real genome, to which the expressed genes were mapped; and b) the randomized genome, which was created by shuffling position indexes of the same number of genes on each chromosome. Supplementary Fig. S1 [see Additional file 1] presents plots of the mean coexpression index values for both ES and EB states. As shown, the mean coexpression index from the real genome data was consistently higher than that from the random genome data across different window sizes in both ES and EB. This suggested a significant pattern of coexpression of neighboring genes in both ES and EB cells. The plots (Supplementary Fig. S1 [see Additional file 1]) further showed that the mean coexpression index decreased greatly when the neighboring gene number increased from 2 to 20. Beyond 20 neighboring genes, the decrease of the coexpression level became less significant, and this continued for domains of up to 50 neighboring genes. This finding suggested that clusters of up to 20 neighboring genes may be coexpressed on the chromosome. We therefore used the window size of 20 genes for subsequent coexpression index analyses in this study.

We next determined the P value of both the coexpression index and mean coexpression index at the window size of 20 genes by Monte-Carlo simulation. Coexpression index and mean co-expression index values were calculated from 10,000 randomized genome data (see Methods). The derived Monte-Carlo distributions (Supplementary Fig. S2 [see Additional file 2] and S3 [see Additional file 3]) allowed determination of the *P *value for the coexpression index of a given gene or the mean co-expression index of a given set of genes. For the ES and EB expression data, the mean coexpression index was 0.027 and 0.021 respectively. The *P *values for both mean co-expression index values were below 0.00001 (Supplementary Fig. S2 [see Additional file 2] and S3 [see Additional file 3]). The Monte-Carlo simulation thus provided further evidence that coexpression of neighboring genes was significant in the real genome in ES and EB cells.

The coexpression of neighboring genes may be due to duplicated genes, which often remain adjacent and have similar expression patterns [18]. To assess the effect of gene duplication on the coexpression index in ES and EB, we re-generated randomized genome data sets where all tandem duplication genes [19] were removed, and conducted Monte-Carlo simulation again. The mean coexpression index values were 0.026 and 0.020 in ES and EB, respectively, from the real genome data where tandem duplication genes were removed. The *P *value of the mean coexpression index values after the removal of tandem duplication genes was still very low (below 0.00001 in both ES and EB). Therefore, gene coexpression on the chromosome in ES and EB cells was statistically significant regardless the effect of tandem duplication of genes, which had little impact on the observed pattern.

### 2. Transcriptome coexpression map

**Figure 1:**
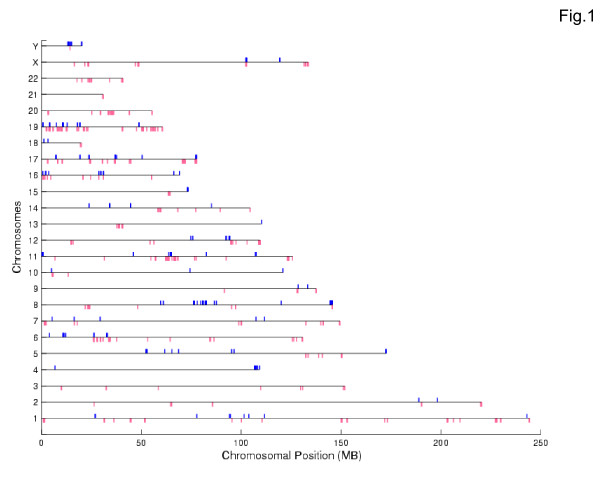
Genomic overview of coexpression chromosomal domains in human ES and EB cells. The domains were identified by the genes which have significant correlation in the expression profile with adjacent genes (10 upstream and 10 downstream) along the chromosome, at a coexpression index threshold of 0.3. Blue bars on the chromosome represent coexpression domains in ES, while red bars represent coexpression domains in EB.

**Figure 2:**
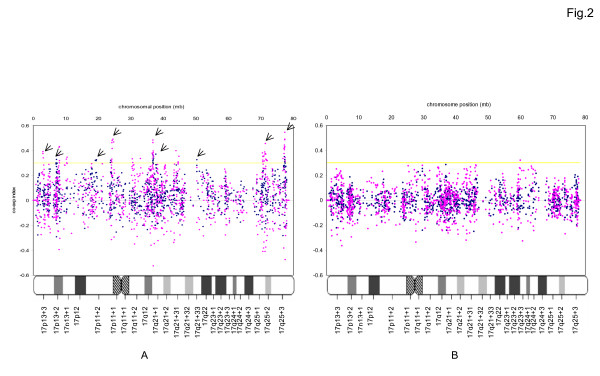
Comparative transcriptome map of human ES and EB on chromosome 17. The transcriptome map shows the coexpression index value of genes displayed according to their positions along the chromosome (horizontal axis). Each dot represents a gene expressed in ES (blue) or EB (red) cells. The yellow line represents the threshold coexpression index (0.3). The coexpression index was calculated as the average Pearson's correlation coefficient between the expression level of a given gene with that of every neighboring gene (10 up- and 10 downstream). The chromosomal position on the horizontal axis is illustrated by both coordinates (mega base pair) and the ideogram with markers of cytogenetic bands [20]. The statistical significance of the coexpression pattern was confirmed by Monte-Carlo simulation (see Methods). ***A*. **Transcriptome map constructed with the real genome data, where multiple peaks of coexpression above the threshold are visible and appear as non-random. The co-expressed genes include, from left to right, ORIA1, SOX15, ALDH3A2, PIPOX, KRTHA8, STAT3, COX11, MIRPL38, and PCYT2 (indicated by arrows). ***B*. **Transcriptome map by randomized genome data, where peaks of coexpression are random and few are above the threshold.

Given the significant pattern of coexpression, we used the window size of 20 and the coexpression index value of 0.3 as the threshold for transcriptome mapping of ES and EB. The threshold coexpression index corresponded to the *P *value of 0.0004 in the ES expression set and 0.01 in EB. At the threshold value and above, 205 genes expressed in ES showed coexpression with their neighbors. These genes, along with the 20 genes in the neighborhood, defined 205 chromosomal domains of coexpression. Some of the domains overlapped with each other and represented physically continuous longer stretches of genes on the chromosome. In total, 1,925 unique genes were recognized from all the identified coexpression domains in the ES transcriptome. Among the 205 genes which had the coexpression index above the threshold, 62 genes were differentially expressed between ES and EB (*P *= 0.05). Similarly, 549 chromosomal domains of coexpression with a total of 3,860 unique genes were identified from the EB expression data. Among the 549 genes that were above the threshold, 173 genes were differentially expressed (*P *= 0.05). The results of the coexpression chromosomal domains are provided in Supplementary Tables S2 [see Additional file 7] and S3 [see Additional file 8] for ES and EB, respectively. Fig. 1 presents a genomic overview of the coexpression chromosomal domains. Fig. 2 presents the transcriptome coexpression maps of ES and EB on chromosome 17. Besides the transcriptome map constructed using the real genome data, a transcriptome map constructed from the randomized genome data is also presented (Fig. 2). On the transcriptome map, the coexpression index value of each gene is displayed according to the position of the gene along the chromosome (horizontal axis). Each dot represents a gene expressed in ES (blue color) or EB (red). The yellow line represents the threshold value of the coexpression index (i.e. 0.3). As illustrated on chromosome 17 (Fig. 2), the transcriptome map by the real genome data (Fig. 2A) showed various peaks of coexpression on the chromosome. Among all the genes analyzed on this chromosome, 21 (or 61) genes had coexpression index values above the threshold in ES (or EB). The coexpressed genes included ORIA1, SOX15, ALDH3A2, PIPOX, KRTHA8, STAT3, COX11, MIRPL38, and PCYT2, as displayed on the map. Many coexpressed genes were adjacent to each other, forming even larger chromosome domains. In contrast, the transcriptome map by the random genome data (Fig. 2B) showed more uniform peaks of coexpression on the chromosome and few peaks were above threshold value. The comparative transcriptome maps of ES and EB for the other chromosomes are presented in Supplementary Fig. S4 [see Additional file 4].

### 3. Coexpression pattern of chromosomal domain

**Figure 3:**
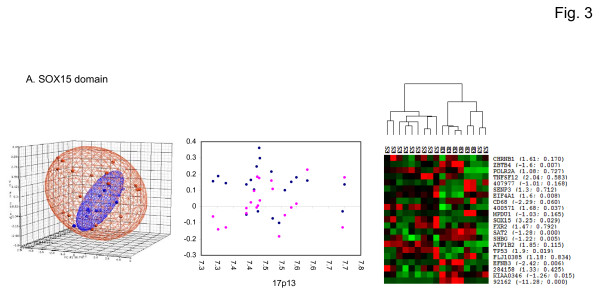
Coexpression chromosomal domains of SOX15 (**Fig 3**), PTPRCAP (**Fig 4**), and NGFRAP1 (**Fig 5**), which represent three different coexpression patterns in ES and EB. ***Left***, principal component analysis map. Each dot represents a gene of a domain expressed in ES (blue dots) or EB (brown dots). The wire mesh was constructed based on a standard error of 2. Genes with correlated expression profiles tend to cluster tightly together into a small-size ellipsoid by the wire mesh, while genes with less similar expression profiles form a looser cluster with larger size of ellipsoid. The SOX15 domain is shown to have a higher coexpression level in ES than EB, PTPRCAP domain has a higher coexpression in EB than ES, while NGFRAP1 domain shows a non-differential coexpression pattern between ES and EB. ***Middle***, transcriptome map of the chromosome domain (genomic locations are in mega base pairs). Each gene on the heatmap is indicated. In the SOX15 domain, most genes have higher coexpression index values in ES than in EB. In the PTPRCAP domain, most genes have higher coexpression index values in EB than ES. In the NGFRAP1 domain, most genes have similar coexpression index values between ES and EB. ***Right*, **heatmap of gene expression and unsupervised hierarchical cluster analysis. Each column in the heatmap corresponds to an ES or EB sample. Each row represents the relative level of expression of a single gene. The genes are arranged from top to bottom in the same order as they appear on the chromosome from 5' to 3' shown on the transcriptome map. Each gene is denoted by its symbol, followed by the fold-change and ANOVA *P *value (in brackets) indicating the level of differential expression of the gene between ES and EB. *Red*, high expression relative to the mean expression; *green*, low expressions relative to the mean expression; *black*, no significant change in expression level between the sample and mean.

**Figure 4:**
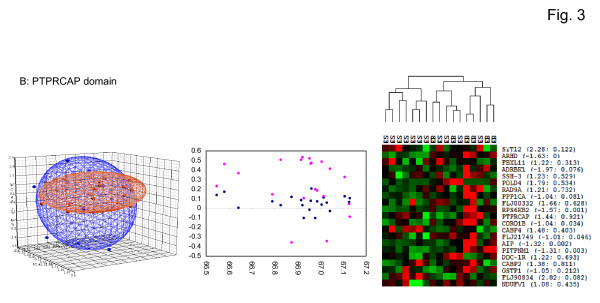
Coexpression chromosomal domains of SOX15 (***A***), PTPRCAP (***B***), and NGFRAP1 (***C***), which represent three different coexpression patterns in ES and EB. ***Left***, principal component analysis map. Each dot represents a gene of a domain expressed in ES (blue dots) or EB (brown dots). The wire mesh was constructed based on a standard error of 2. Genes with correlated expression profiles tend to cluster tightly together into a small-size ellipsoid by the wire mesh, while genes with less similar expression profiles form a looser cluster with larger size of ellipsoid. The SOX15 domain is shown to have a higher coexpression level in ES than EB, PTPRCAP domain has a higher coexpression in EB than ES, while NGFRAP1 domain shows a non-differential coexpression pattern between ES and EB. ***Middle***, transcriptome map of the chromosome domain (genomic locations are in mega base pairs). Each gene on the heatmap is indicated. In the SOX15 domain, most genes have higher coexpression index values in ES than in EB. In the PTPRCAP domain, most genes have higher coexpression index values in EB than ES. In the NGFRAP1 domain, most genes have similar coexpression index values between ES and EB. ***Right*, **heatmap of gene expression and unsupervised hierarchical cluster analysis. Each column in the heatmap corresponds to an ES or EB sample. Each row represents the relative level of expression of a single gene. The genes are arranged from top to bottom in the same order as they appear on the chromosome from 5' to 3' shown on the transcriptome map. Each gene is denoted by its symbol, followed by the fold-change and ANOVA *P *value (in brackets) indicating the level of differential expression of the gene between ES and EB. *Red*, high expression relative to the mean expression; *green*, low expressions relative to the mean expression; *black*, no significant change in expression level between the sample and mean.

**Figure 5:**
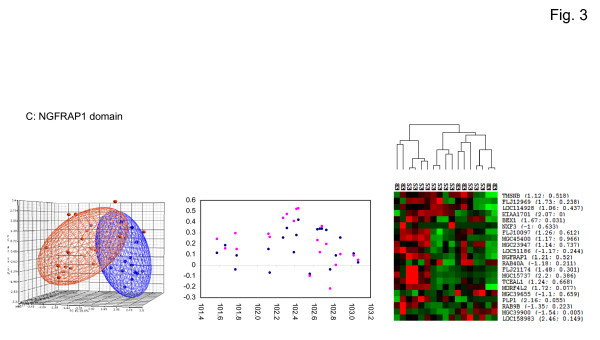
Coexpression chromosomal domains of SOX15 (***A***), PTPRCAP (***B***), and NGFRAP1 (***C***), which represent three different coexpression patterns in ES and EB. ***Left***, principal component analysis map. Each dot represents a gene of a domain expressed in ES (blue dots) or EB (brown dots). The wire mesh was constructed based on a standard error of 2. Genes with correlated expression profiles tend to cluster tightly together into a small-size ellipsoid by the wire mesh, while genes with less similar expression profiles form a looser cluster with larger size of ellipsoid. The SOX15 domain is shown to have a higher coexpression level in ES than EB, PTPRCAP domain has a higher coexpression in EB than ES, while NGFRAP1 domain shows a non-differential coexpression pattern between ES and EB. ***Middle***, transcriptome map of the chromosome domain (genomic locations are in mega base pairs). Each gene on the heatmap is indicated. In the SOX15 domain, most genes have higher coexpression index values in ES than in EB. In the PTPRCAP domain, most genes have higher coexpression index values in EB than ES. In the NGFRAP1 domain, most genes have similar coexpression index values between ES and EB. ***Right*, **heatmap of gene expression and unsupervised hierarchical cluster analysis. Each column in the heatmap corresponds to an ES or EB sample. Each row represents the relative level of expression of a single gene. The genes are arranged from top to bottom in the same order as they appear on the chromosome from 5' to 3' shown on the transcriptome map. Each gene is denoted by its symbol, followed by the fold-change and ANOVA *P *value (in brackets) indicating the level of differential expression of the gene between ES and EB. *Red*, high expression relative to the mean expression; *green*, low expressions relative to the mean expression; *black*, no significant change in expression level between the sample and mean.

The transcriptome map highlighted different coexpression patterns of chromosomal domains between ES and EB (Fig. 1; Fig. 2; Supplementary Fig. S4 [see Additional file 4]). Among the total 754 coexpression domains identified from ES and EB expression data, 18 domains had the coexpression index above the threshold in both ES and EB. The remaining domains had coexpression index values above the threshold in either ES or EB but not in both, indicating that there was differential coexpression between ES and EB. Three specific patterns of coexpression were recognized from the chromosome domains: 1) coexpression observed in ES but not in EB; 2) coexpression observed in EB but not ES, and 3) coexpression observed in both ES and EB. We employed principal component analysis (PCA) and hierarchical clustering analysis to further explore the three different coexpression patterns, using SOX15, PTPRCAP, and NGFRAP1 domains as examples. Figs. 3, 4 and 5 shows the results of the PCA and clustering analysis, along with the transcriptome map and gene expression heatmap. Each gene is also shown with the fold-change and ANOVA *P *value for information of differential expression in the figures. The parallel presentation of the results in Fig. 3 allow the direct visualization of differential or similar coexpression (or expression) of domains (or domain genes) between ES and EB.

As shown in Fig. 3, the SOX15 domain represented a pattern in which the degree of coexpression was higher in ES than EB. The domain extended for about 410 kb at the 17p13 region of chromosome 17. As illustrated in the transcriptome map (Fig. 3, middle), most genes on the domain had higher coexpression index values in ES (blue dots) than EB (red dots), with the highest score observed in SOX15 (0.36 in ES *vs *0.176 in EB), followed by FXR2 (0.2985 in ES *vs *0.037 in EB). No gene expressed in EB had the coexpression index value above the threshold, and the highest one was only 0.184. PCA is robust in capturing and presenting major variations of expression profiles on leading principal components. The PCA map (Fig. 3, left) revealed a large difference in the clustering of domain genes and thus the differential coexpression of the gene cluster between ES and EB. As shown, the domain genes were clustered tightly together to a small size of ellipsoid by the correlated expression profiles in ES (blue dots), but clustered loosely to a larger ellipsoid by the less correlated expression profiles in EB (brown dots). The heatmap and cluster analysis showed less diversity on the expression profile among genes in ES than EB samples, indicating a higher correlation of expression in ES. Interestingly, although the domain was differentially coexpressed, 11 genes of the domain were not differentially expressed between ES and EB samples (ANOVA *P *value = 0.05). The other 10 genes were differentially expressed, but displayed various degrees of up- and down-regulation in comparison to the mean expression level.

For the PTPRCAP domain (Fig. 4), on the other hand, the degree of coexpression was higher in EB than ES. The domain stretched for 583 kb at 11q13.3. The transcriptome map (Fig. 4, middle) showed that most genes in the domain had higher coexpression index values in EB (blue dots) than ES (red dots). Ten genes on the domain had coexpression indices above the threshold in EB, with the highest observed in PPP1CA (0.535 in EB *vs *0.031 in ES). No gene expressed in ES had the coexpression index above the threshold, and the highest coexpression index was 0.202 (CORO1B). The higher coexpression level of the domain in EB than ES was also illustrated by the PCA and cluster analyses. The PCA map showed that the genes expressed in EB (blue dots) were tightly clustered to a small ellipsoid, whereas the genes expressed in ES (brown dots) were loosely clustered to a much larger ellipsoid. The heatmap showed less diversity on the expression level among genes expressed in EB than ES. Although the gene cluster was differentially coexpressed, 13 genes on the domain were not differentially expressed between ES and EB, the other 8 genes were differentially expressed, but exhibited mixed patterns of up- or down-regulation.

While the SOX15 and PTPRCAP domains showed differential coexpression, the NGFRAP1 domain (Fig. 5) displayed a pattern of similar coexpression between ES and EB. The domain was 1,498 kb long and located at Xq22.2. Five or six genes had the coexpression index above the threshold in ES and EB, respectively, with the highest observed in NGFRAP1 (0.418 in ES and 0.523 in EB). The PCA map showed similar clustering of the genes and the heatmap showed similar expression profiles between ES and EB, suggesting a similar degree of coexpression in ES and EB. Although similarly coexpressed, six genes of the domain were differentially expressed between ES and EB.

### 4. Distribution of coexpression domain

The transcriptome map highlighted a non-random distribution pattern of the coexpression chromosomal domains on the genome (Fig. 1; Fig. 2; supplementary Fig. S4 [see Additional file 4]). We sought to determine whether any chromosome was significantly enriched or over-represented by coexpression domains or domain genes. A Fisher's exact test was conducted with the number of domains and domain genes located on each chromosome and on the entire genome. Table 1 lists the frequency of domains and domain genes on each chromosome and the *P *value derived from the Fisher's exact test (detailed information is provided in supplementary Tables S2 [see Additional file 7] and S3 [see Additional file 8]). As shown, the coexpression domains and the domain genes were highly frequent on chromosomes 8, 11, 16, 17, 19, and Y in ES, while on chromosomes 6, 11, 17, 19, and 20 in EB. These chromosomes were associated with low Fisher's *P *values (= 0.05), suggesting a significant enrichment (or over-representation) in genes coexpressed at the domain level.

We next determined whether the identified coexpression chromosomal domains correlated with any known cytogenetic bands on the chromosome. Giemsa positive or negative bands, centromeric regions, and variable length heterochromatic regions were examined at the 850-band resolution [20]. These cytogenetic patterns represent distinct and reproducible structure of extended and compacted regions on the chromosome. Table 3 shows the frequency of domain genes in each structural pattern and the P value by the Fisher's exact test (detailed information is provided in Supplementary Tables S2 [see Additional file 7] and S3 [see Additional file 8]). Among all of the genes in the coexpression chromosomal domains in ES, 62.2% (783 genes) were located in Giemsa-negative bands, and 36.6% (461) in Giemsa-positive bands. These proportions were similar to those predicted from all known genes on the entire genome [20]. Ten coexpression domains genes were located in the variable length heterochromatic region (19q-11 to 19q-13), and the *P *value 0.028. The variable length heterochromatic region was thus significantly enriched by the coexpression genes in ES. On the other hand, the Giemsa-negative region was significantly enriched by the coexpression genes in EB (*P *value 0.008).

### 5. Gene ontology analysis

**Figure 6:**
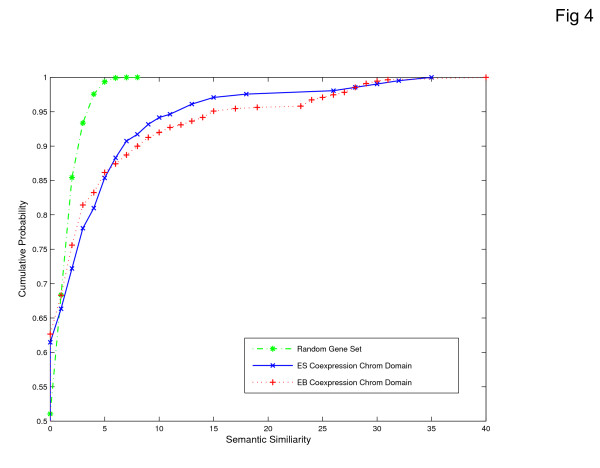
The distributions of functional semantic similarity scores derived from coexpression chromosome domains in ESC and EB, and from a randomized gene set. The green line on the plot represents the distribution from the random data, the blue and red lines are those from ESC and EB, respectively. The X-axis indicates functional semantic similarity scores, and the Y-axis indicates the accumulative probability of coexpressed genes in domains. For the random gene set, the cumulative probability reached to 1 when the functional similarity was up to 8. That means all the random gene pairs have the functional similarity 8 or below. For coexpression domain genes of ESC and EB, the cumulated probability did not reaches 1 (i.e. 100% of gene pairs) until the functional similarity was over 35, indicating higher functional similarities from more coexpressed genes. The distributions from ESC and EB domain gene sets were similar to each other but significantly different from that from the random gene set (p < 10E-10 by the Kolmogorov-Smirnov test).

In order to examine the physiological and biological significance of the identified coexpression chromosome domains, we conducted a functional semantic similarity analysis based on the Gene Ontology (GO). The semantic similarity is a measure of the number of GO terms shared by coexpressed genes of a domain [21,22]. We expected that if coexpression domains were biologically significant, they would be more likely related to certain functions. Otherwise, they would be little functional relevant. Fig. 6 shows the distribution of semantic similarity scores calculated from coexpression chromosome domains in ES and EB, respectively, and from a randomized gene set. For the random data, the cumulative probability of genes reached 1 when the functional similarity was only as high as 8. That meant all genes in the random data set had the functional similarity of 8 or below. For the coexpression domains, the cumulated probability of 1 (i.e. 100% of coexpressed gene pairs) corresponded to the semantic similarity above 35, indicative of much higher functional similarities in the domain genes. The distributions by the ES and EB coexpression domains were significantly different from that by the random data set (Kolmogorov-Smirnov test *P *< 10E-10). The results suggested that the coexpression chromosome domains were biologically and physiologically significant.

We next determined the GO terms which were significant associated with coexpression chromosome domains, using the Fisher's exact test. The results are shown in Supplementary Tables S4 [see Additional file 9] and S5 [see Additional file 10] (*P *value ≤ 0.05). Many domains were associated with biological functions, particularly with the regulation of transcription, transcription factor activity, and chromosome organization. Some domains were associated with ES – important functions or biological processes, such as apoptosis, pattern specification, histogenesis and organogenesis, embryogenesis and morphogenesis.

## Discussion

ES cell gene expression is carefully regulated and cells either maintain the pluripotent state by self-renewal or undergo differentiation. This is the first study to investigate the coexpression of genes along the chromosome in human ES cells and their earliest stage of differentiation in culture, EB's. Significant coexpression patterns were revealed and confirmed by random tests and Monte-Carlo simulation. The coexpression is suggestive of transcriptional regulation operating at the chromosome domain level in ES and EB cells. The coexpression domains do not appear to represent amplicons or regions of chromosome imbalance that were previously described in cancer cells [23]. The chromosome region with adjacent localization of the genes NANOG, STELLAR, and GDF3 has been considered as a hotspot for teratocarcinoma [24]. Our study however indicated that the genes in the region were not coexpressed, suggestive of no transcriptional regulation operating at this domain in ES or EB. Nevertheless, the identified coexpression chromosome domains are biologically and physiologically significant, some of which are associated with functions important to ES development. New coexpression chromosome domains would possibly be observed when each cell line had been analyzed separately. Recent studies have shown that some ES cell lines exhibit unique morphological and genetic features [25]. The cell line BG01V, for example, shows abnormal chromosome and karyotype, different from other ES cells [26,27].It is thus important to examine cell line specific patterns of local coexpression, which will be the future direction of our studies.

The genes LIFR, GP130, STAT3, OCT3/4, SOX2, UTF-1, FOXD3, ERAS, TEL1, FGF4, NANOG, NODAL, TDFG1, CER1, and ABCG2 have shown to be critical for ESC self-renewal and self-renewal and regarded as the "signature" [15,16,28]. Some of these genes were not coexpressed on the chromosome (Table 3), suggesting that global regions still tend to be involved in determining the overall state of the ES cell and provide context for cell-type specific signaling. Nonetheless, the other ES-signature genes did show coexpression along the chromosome. The genes that were adjacent and coexpressed with the signature genes were often related to development and transcriptional regulation. STAT3, for example, is a transcription factor which plays a central role within ES self-renewal pathways and feed-back loops [29,30]. The STAT3 gene, located at 17q21, was coexpressed in ES (coexpression index 0.37) but not in EB (-0.17) (Table 3). The coexpression chromosome domain where STAT3 resides also contained the duplicated genes STAT5B and STAT5A, as well as TCF1, a transcription factor important in proliferation and differentiation. Other ES-signature genes, UTF1, TLE1, and OCT3/4, showed higher coexpression index in EB (0.299, 0.285, and 0.23, respectively, although slightly lower than the threshold value) than in ES (0.125, 0.08, and 0.10, respectively). UTF1 is a transcription factor, and the domain where the UTF1 gene is located (at 10q26) contained two other transcriptional factors, VENTX2 [a homeodomain protein implicated in mesodermal patterning and hemopoietic stem cell maintenance [31]], and NKX6-2. TLE1 is an ES cell-specific gene coding a RNA-binding protein which functions downstream of the LIF and Oct3/4 pathways [32,33]. The TLE1 gene domain is located at 9q21.32 and the coexpressed genes included the duplicated gene TLE4 and signal transduction genes GNAQ, GKAP42, and GNA14. OCT3/4 is also a transcriptional factor critical for ES cell self-renewal [34]. The OCT3/4 domain, located at 6p21.31, contained NFKBIL1 and MHC class I genes. In addition to the ES signature genes, other genes important for ES cell development were also found to be coexpressed on the chromosome in ES or EB. SOX15, for example, is a transcription factor involved in the regulation of embryonic development and transcriptional control in ES cells [35]. The gene was significantly up-regulated in ES cells (*P *value 0.029, fold-change 3.25). The SOX15 domain (Fig. 3) showed coexpression in ES cells but not in EB cells, as described above. Among the genes on this domain, EFNB3 belongs to the ephrin gene family and is implicated in development, TNFSF1 is a cytokine belonging to the tumor necrosis factor (TNF) ligand family, and POLR2A, ZBTB4, TP53, and FXR2 are all involved in transcription. Apparently, the differentiation or self-renewal of ES cells was evidenced not only by the differential expression of individual genes at the global level, but also by the differential coexpression of genes at the chromosomal domain level.

Chromosomal clustering of functionally related genes has been demonstrated in various eukaryotes, including the yeast, fruit fly, nematode, and human [1,5,7,8]. Natural selection might have organized genes to clusters on the chromosome according to the molecular function or biological process so that their expression can be coordinately regulated. The coexpression of physically adjacent genes may be caused by the long range effect of transcription factors, chromatin structure modifications, or increased concentration of components of the transcriptional machinery (such as transcription factors) in a particular subnuclear location of chromosomal segments [18]. The coexpression could also be due to duplicated genes, which often remain adjacent and have similarexpression patterns [18]. Our study revealed that gene duplication had a minimal impact and was not a major contributing factor for the observed coexpression pattern in ES and EB. Our study also revealed differential local coexpression between ES and EB. Differentially coexpressed genes may not be differentially expressed, while similarly coexpressed genes may be differentially expressed. The transcriptome map thus provides a basis for examining how transcriptional regulation interacts with genomic structure and how genes clustered on the chromosome are coexpressed during the ES self-renewal and differentiation.

## Conclusion

Taken together, the transcriptome map provides information on transcriptional events operating at the local chromosome level in ES cells and localized coexpression of genes during differentiation. The identified coexpression chromosome domains are significantly associated with biological or physiological functions, some of which were important for ES development. Global and local regions are both involved in determining the overall state of the ES cell and provide context for cell-type specific signaling. The findings and methods reported in this investigation advance our understanding of how genome organization affects gene expression in human ES cells and help to identify new mechanisms and pathways controlling ES self-renewal or differentiation.

## Methods

### Human embryonic stem cell culture

The human ES cell lines I6, BG01V, BG01, BG02, and BG03 [36]used in this work are registered with the NIH [37]. The relatedness of the cell lines to each other in ES and EB are shown in Supplementary Table S1 [see Additional file 6]. Each cell line was represented by a single sample, except I6 (2 samples). Addition samples were from pooled cultures of different samples. Human ES cells were primarily cultured on mitomycin C inactivated mouse embryonic fibroblasts (MEF) or mitotically inactivated HS27 human fibroblast cells (HS27, ATCC), in DMEM/F12-Glutamax 1:1, 20% Knockout Serum Replacement, 2mM nonessential amino acids, 100 μM beta-mercaptoethanol, 50 μg/ml Pen-Strep (all from Invitrogen), and 4 ng/ml human recombinant basic fibroblast growth factor (bFGF/FGF2; PeproTech Inc., Rocky Hill, NJ). Cultures were expanded by passaging clumps of ~ 10–100 cells. Before harvesting RNA, human ES cells were passaged on Matrigel (BD Biosciences) or fibronectin (Sigma)-coated plates and cultured in human ES medium conditioned with inactivated MEF [36] for at least an additional 3 passages. A detailed manual of the human ES cell described here is available online at the NIH stem cell web site [37].

### Differentiation of ESC as embryoid bodies

Human ES cells growing on feeders or feeder-free conditions were harvested by collagenase (1 mg/ml, Invitrogen or Sigma) and resupsended in DMEM/F12 with 15% FCS, 5% KSR, 20 mM L-Glutamine, 0.5 U/ml penicillin, 0.5 U/ml streptomycin, 0.1 mM β-mercaptoethanol, and 1x non-essential amino acids. Floating spheres were grown for up to 14 days in the same medium before RNA extraction. Supplementary Fig. S5 [see Additional file 5] shows undifferentiated human ES cell lines cultured on inactivated MEF and grown in a feeder free condition, and embryoid bodies generated by growing ES cells in ultralow attachment plates to form floating spheres.

### RNA extraction, BeadArray preparation, and data processing

RNA was extracted from 14 ES and EB samples using a standard TriZol (Invitrogen) method. The BeadArray used in this study contained 23,584 probes, representing 20,692 genes recognized by RefSeq [38]. Each gene or transcript was represented on the BeadArray by 3–10 oligonucleotides, each 50-base long. The intensity data on the array were calculated from the images generated by the BeadArray Reader (Illumina). Details of the RNA amplification, labeling, and hybridization steps are available from [39]. The mean intensity of an individual probe was calculated across all arrays, normalized by the quantile method, and the log_2 _ratio of each value to this mean was calculated. When several probes corresponded to the same gene (*i.e*. if different probes had the same gene symbol or GenBank ID), a single probe was kept for the analysis. Data of the chromosomal location and cytogenetic structural pattern of each gene were obtained from the RefSeq database [38].

### Construction of the transcriptome map

The transcriptome map was constructed based on the correlation of expression profiles among neighboring genes along the chromosome, using a method similar to that described previously [1,7,23]. The correlation of expression profiles between genes was calculated as the Pearson's correlation coefficient from the expression values of the seven samples representing ES or EB. For each gene, its correlation values with every upstream and downstream neighbor genes within a certain window size were first determined. The average of the correlation values was defined as the coexpression index of this gene. The number of neighboring genes (or 'window size') used to calculate the coexpression index was determined by repeated analysis with different neighboring gene numbers (ranging from 4 to 50), followed by assessment of changes of the coexpression pattern. The statistical significance of coexpression was assessed by Monte-Carlo simulation. In the simulation, random genome data sets were created by shuffling position indices of the same number of genes on each chromosome and the expression profiles of the genes. The coexpression index of each gene and mean values of the coexpression index of each data set were then calculated from the random data. This process was repeated for 10,000 times; the resulting distributions of both coexpression index and mean coexpression index were fit to the Gaussian density function. The *P *values of the coexpression index and mean coexpression index from the real data were determined according to the derived probability distribution by the simulation. The graphical presentation of the transcriptome map on each chromosome was generated by plotting the coexpression index value of each gene displayed according to its position along the chromosome.

### Biological significance of coexpression chromosome domain

In assessing biological and physiological significance of the identified coexpression chromosome domains, each gene in the domains was characterized by the set of associated Gene Ontology (GO) terms. GO terms for all human genes were obtained from the GO database [40]. The semantic similarity k of a pair of genes A and B was measured by the number of terms they share, (GO_A _∩ GO_B_) where GO_X _denotes the set of GO terms for gene x. k is set to zero if one or both genes have no terms. The semantic similarity was calculated between the gene with the highest coexpression index value and every other gene in a domain from ES and EB, respectively. The semantic similarity was also calculated from 10,000 randomly selected gene pairs. The resulting distributions of functional semantic scores were examined by the Kolmogorov-Smirnov test for the statistical differences.

### Significantly enriched GO terms, chromosomes, and cytogenetic patterns

The Fisher's exact test was conducted to calculate the hypergeometric probability of observing a GO term as enriched in each group of genes. In specific, the probability *p *that a GO term is significantly enriched in a group of genes was calculated with the following formula:

p=1−∑i=0k−1(Ai)(G−An−1)(Gn)
 MathType@MTEF@5@5@+=feaafiart1ev1aaatCvAUfKttLearuWrP9MDH5MBPbIqV92AaeXatLxBI9gBaebbnrfifHhDYfgasaacH8akY=wiFfYdH8Gipec8Eeeu0xXdbba9frFj0=OqFfea0dXdd9vqai=hGuQ8kuc9pgc9s8qqaq=dirpe0xb9q8qiLsFr0=vr0=vr0dc8meaabaqaciaacaGaaeqabaqabeGadaaakeaacqWGWbaCcqGH9aqpcqaIXaqmcqGHsisldaaeWbqaamaalaaabaWaaeWaaeaafaqabeGabaaabaGaemyqaeeabaGaemyAaKgaaaGaayjkaiaawMcaamaabmaabaqbaeqabiqaaaqaaiabdEeahjabgkHiTiabdgeabbqaaiabd6gaUjabgkHiTiabigdaXaaaaiaawIcacaGLPaaaaeaadaqadaqaauaabeqaceaaaeaacqWGhbWraeaacqWGUbGBaaaacaGLOaGaayzkaaaaaaWcbaGaemyAaKMaeyypa0JaeGimaadabaGaem4AaSMaeyOeI0IaeGymaedaniabggHiLdaaaa@49CA@

where *k *is the number of genes in the group, *G *is the total number of genes, *n *is the number of genes in the group with a given GO term, and *A *is the total number of genes with a given GO term. The domains which had at least four genes associated with GO terms at *P *= 0.05 were selected. Like-wisely, significantly enriched chromosomes and cytogenetic structural patterns by each group of genes were also determined by the Fisher's exact test.

### Principal component analysis, clustering analysis, and identification of differentially expressed genes

Unsupervised hierarchical clustering analysis and principal component analysis (PCA) were conducted using software Cluster [41], TreeView, and Partek™, based on the Pearson's correlation. Differentially expressed genes between ES and EB were identified by ANOVA using Partek™.

## Authors' contributions

MZ conceived this project, participated in the design and bioinformatics analysis, and drafted the manuscript. MSR, MPM, and JL also participated in the project design and coordination. HL and YS performed the bioinformatics analysis. YL and SS conducted the laboratory work. All authors read and approved the final manuscript.

## Supplementary Material

Additional File 1 **Supplementary Figure S1 **(Supplementary Fig S1 Window size *vs *domain gene number.ppt). Plots of the means of the coexpression index calculated from the real genome data set (blue line) and 10,000 randomized genome data sets (red line) under the neighboring gene numbers of 4 to 50. The mean coexpression index decreased greatly when the domain size increased from 2 to 20 neighbor genes. Beyond 20 neighboring genes, the decrease of the coexpression level was slower, and this trend continued for domains of up to 50 neighboring genes. ***A***. Plot based on the expression data in ES; ***B***. Plot based on the expression data in EB. Click here for file

Additional File 2 **Supplementary Figure S2 **(Supplementary Fig S2 Distribution of co-exp index.ppt). Monte-Carlo distribution of the coexpression index generated from 10,000 randomized genome data. The coexpression index was calculated in the sliding window of 20 neighboring genes. The distribution allows the determination of the *P *value of the coexpression index. ***A***. By the expression data from ES; the *P *value for the coexpression index threshold 0.3 is 0.0004. ***B***. By the expression data from EB; the *P *value for the co-expression index threshold 0.3 is 0.01. Click here for file

Additional File 3 **Supplementary Figure S3 **(Supplementary Fig S3 Distribution of mean co-exp index.ppt). Monte-Carlo distribution of the mean coexpression index generated from 10,000 randomized genome data. The mean coexpression index was calculated in the sliding window of 20 neighboring genes. The mean coexpression index calculated from the real genome data set is marked with an arrow. The distribution allows determination of the *P *value of the mean co-expression index. ***A***. By the expression data in ES; the mean coexpression index of the real expression data is 0.026 (*P *< 0.00001). ***B***. By the expressed data in EB; the mean coexpression index from the real expression data is 0.021 (*P *< 0.00001). Click here for file

Additional File 4 **Supplementary Figure S4 **(Supplementary Fig S4 Transcriptome map of each chromosome.pdf). Comparative transcriptome map of human ES and EB on each chromosome of the genome. The transcriptome map shows the coexpression index value of genes that are displayed according to the position along the chromosome (horizontal axis). Each dot represents a gene expressed in ES (blue color) or EB (red). The yellow line represents the threshold of the coexpression index (0.3). The coexpression index was calculated as the average Person's correlation coefficient between the expression level of a given gene with that of every neighboring gene (10 up- and 10 downstream). The statistical significance of the co-expression pattern was confirmed by Monte-Carlo simulation (see Methods). Click here for file

Additional File 5 **Supplementary Figure S5 **(Supplementary Fig S5 ES and EB photo.ppt). Undifferentiated human ES cell lines cultured on inactivated MEF (**A**) or grown in a feeder free condition (**B**). To differentiate ES cells, embryoid bodies were generated by growing ES cells in ultralow attachment plates to form floating spheres (**C**). Click here for file

Additional File 6 **Supplementary Table S1 **(Supplementary Table S1 Cell lines and relatedness.doc). Cell lines used in the study and their relatedness to each other in ES and EB. The correlation matrix was calculated by Pearson's correlations coefficient of expression profiles. Click here for file

Additional File 7 **Supplementary Table S2 **(Supplementary Table S2 Coexpression chrom domains in ES.doc). List of coexpression chromosomal domains identified in ES at the co-expression index threshold 0.3 and the window size of 20, and associated GO terms (Fisher P ≤ 0.05) Click here for file

Additional File 8 **Supplementary Table S3 **(Supplementary Table S3 Coexpression chrom domains in EB.doc). List of coexpression chromosomal domains identified in EB at the co-expression index threshold 0.3 and the window size of 20, and associated GO terms (Fisher P ≤ 0.05). Click here for file

Additional File 9 **Supplementary Table S4 **(Supplementary Table S4 GO terms of each domain in ES.xls). Gene Ontology terms associated with each coexpression chromosomal domains identified in ES (Fisher P ≤ 0.05). Click here for file

Additional File 10 **Supplementary Table S5 **(Supplementary Table S5 GO terms of each domain in EB.xls). Gene Ontology terms associated with each coexpression chromosomal domains identified in EB (Fisher P ≤ 0.05). Click here for file

## Figures and Tables

**Table 1:** The number of coexpression chromosomal domains and genes located in the domains on each chromosome, and the associated P values of by the Fishers exact test (**p value < 0.01; *p value < 0.05). The domains were identified at the coexpression index threshold 0.3 and window size 20.

Chromosome	Total genes	ES		EB	
		
		Number of coexpression domains	Number of genes located in the domains	Number of coexpression domains	Number of genes located in the domains
1	2610	11	127	52	373
2	1748	2	40	21	130
3	1381	0	0	10	156
4	1024	7	50	0	0
5	1190	11	118**	10	99
6	1394	11	96	38*	251**
7	1378	5	95	23	186
8	927	26**	164**	17	137
9	1076	2	46	14	80
10	983	4	63	4	51
11	1692	23*	189**	55**	353**
12	1268	12	62	22	160
13	496	1	21	8	37
14	1173	8	90	9	111
15	906	5	30	5	35
16	1032	15*	188**	16	208**
17	1394	21**	198**	61**	337**
18	400	2	28	2	25
19	1592	27**	211**	98**	569**
20	710	0	0	28*	176**
21	337	0	0	3	30
22	701	0	0	14	133**
X	1141	8	59	28	174
Y	255	7*	30*	1	19

**Table 2:** Frequency of coexpressed genes in different cytogenetic structural patterns and P values by Fisher's exact tests.

Cytogenetic Regions	ES		EB	
	Gene number and percentage in total	P value	Gene number and percentage in total	P value
Giemsa-negative band	783 ; 62.2%	0.396	1549; 54.1%	0.008
Giemsa-positive band	461; 36.6%	0.733	844; 34.9%	0.997
centromeric band	5; 0.4%	0.757	11; 0.5%	0.668
Variable length heterochromatic band	10; 0.8%	0.028	14; 0.6%	0.097

**Table 3:** ES – signature genes and coexpression with the neighboring genes. The genes in domains are arranged as they are on the chromosome from 5' to 3'.

Gene	Chromosome Location	Coexpression Index (ES)	Coexpression Index (EB)	Gene in Domain*** (20 Neighboring genes: 10 upstrea, 10 downstream)
FOXD3	1p32-p31	0.069	-0.083	INADL; FLJ10884; LOC163782; USP1; DOCK7; ANGPTL3; 400756; AUTL1; 199897; LOC199899; FOXD3; ALG6; ITGB3BP; PGM1; ROR1; 219612; MGC35130; KRTAP4–7; KIAA1573; KIAA1579; JAK1
TDGF1	3p21.31	-0.157	-0.016	401062; XCR1; CCR1; CCR3; CCR2; CCR5; CCRL2; LTF; TMEM7; LRRC2; TDGF1; FLJ36525; TMIE; TSP50; TESSP5; TESSP2; MYL3; PTHR1; MGC23918; HYPB; KIF9
SOX2	3q26.3–q27	0	0	GNB4; BAF53A; MRPL47; 133993; NDUFB5; USP13; PEX5R; TTC14; FXR1; LOC131118; SOX2; 401103; 402152; LOC142678; ATP11B; RP42; MCCC1; LAMP3; KIAA0861; B3GNT5; KLHL6
ABCG2	4q22	-0.039	-0.029	DHRS8; NUDT9; SPARCL1; DSPP; DMP1; LOC153218; IBSP; MEPE; SPP1; PKD2; ABCG2; DKFZp761G058; CEB1; MGC14156; DRLM; TIGD2; LOC285513; SNCA; MMRN; IRAK1BP1; TMSL3
LIFR	5p13-p12	0.187	-0.042	FLJ30596; FLJ25422; SLC1A3; IDN3; FLJ13231; NUP155; FLJ10233; GDNF; 147975; FLJ39155; LIFR; 253254; 401182; OSMR; MGC39830; FYB; C9; DAB2; PTGER4; OSRF; PRKAA1
IL6ST	5q11	0	0	GZMA; FLJ37927; 345643; UNG2; DHX29; KIAA0052; PPAP2A; FLJ90709; DDX4; CRL3; IL6ST; FLJ11795; 345645; MGC33648; FLJ35954; DKFZp761C169; 345651; SNK; FLJ33641; RAB3C; PDE4D
OCT3/4	6p21.31	0.102	0.23	IER3; DDR1; 389376; DPCR1; C6orf15; PSORS1C1; CDSN; PSORS1C2; C6orf18; TCF19; OCT3/4; LOC253018; HLA-C; HLA-B; MICA; HCP5; MICB; BAT1; ATP6V1G2; NFKBIL1; LTA
CER1	9p23-p22	-0.003	0.023	NIRF; GLDC; GASC1; PTPRD; TYRP1; 286343; MPDZ; 401492; NFIB; ZDHHC21; CER1; FLJ25461; C9orf52; SNAPC3; PSIP2; FLJ39267; C9orf39; SH3GL2; ADAMTSL1; FLJ35283; MGC35182
TLE1	9q21.32	0.084	0.285	PCSK5; FLJ11149; GCNT1; C9orf65; CHAC; GNA14; GNAQ; FLJ12643; PSAT1; TLE4; TLE1; FLJ43950; 389763; FLJ31614; MGC20553; UBQLN1; GKAP42; KIF27; C9orf64; HNRPK; C9orf76
NODAL	10q22.1	-0.001	-0.161	C10orf35; COL13A1; H2AFY2; AMID; MGC34695; SARA1; PP; OT7T022; FLJ10751; EIF4EBP2; NODAL; KIAA1274; PRF1; ADAMTS14; C10orf27; 338611; SGPL1; PCBD; UNC5B; SLC29A3; CDH23
UTF1	10q26	0.125	0.298	C10orf39; DPYSL4; PKE; LOC170394; LOC170393; INPP5A; NKX6-2; FLJ25954; GPR123; KIAA1768; UTF1; VENTX2; ADAM8; TUBGCP2; ZNF511; CALCYON; UPA; FLJ26016; ECHS1; PAOX; LOC92170
FGF4	11q13.3	-0.057	0	CPT1A; MRPL21; IGHMBP2; MRGD; MGC21621; TPCN2; MYEOV; CCND1; ORAOV1; FGF19; FGF4; FGF3; 399920; ORAOV2; FADD; PPFIA1; EMS1; SHANK2; 399921; LOC220070; DHCR7
NANOG	12p13.31	-0.06	0.007	RBP5; CLSTN3; PXR1; 341392; M160; CD163; APOBEC1; GDF3; DPPA3; CLECSF11; NANOG; SLC2A14; SLC2A3; FHX; C3AR1; DKFZP566B183; CLECSF6; FLJ10408; CLECSF8; CLECSF9; AICDA
STAT3	17q21	0.37	-0.171	201181; LGP2; GCN5L2; HspB9; RAB5C; KCNH4; HCRT; LGP1; STAT5B; STAT5A; STAT3; PTRF; ATP6V0A1; NAGLU; HSD17B1; DPCK; TCFL4; HUMGT198A; LOC162427; TUBG1; TUBG2
ERAS	Xp11.23	-0.036	0.171	SLC38A5; FTSJ1; PPN; EBP; RBM3; WDR13; WAS; SUV39H1; GATA1; HDAC6; ERAS; PCSK1N; TIMM17B; PQBP1; SLC35A2; PIM2; DKFZp761A052; KCND1; TFE3; JM11; JM4
