# Supplementary figures and images for: Transcriptome coexpression map of human embryonic stem cells
Source: BMC Genomics. 2006 May 2;7:103. doi: 10.1186/1471-2164-7-103 (PMC1523211; doi:10.1186/1471-2164-7-103)

## Slide 1
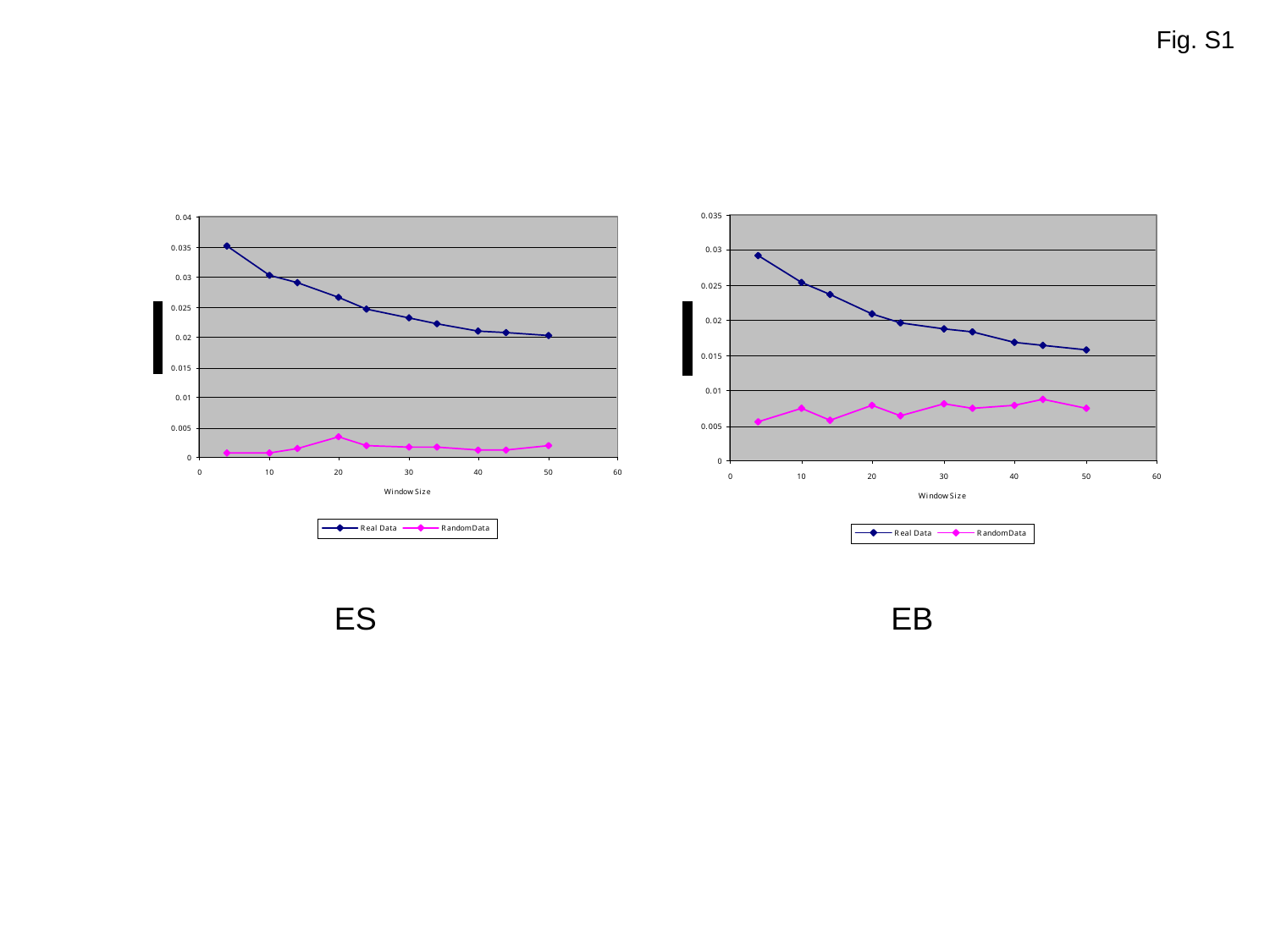

Fig. S1
ES
EB

Supplement: Additional File 1 — Supplementary Figure S1 (Supplementary Fig S1 Window size vs domain gene number.ppt). Plots of the means of the coexpression index calculated from the real genome data set (blue line) and 10,000 randomized genome data sets (red line) under the neighboring gene numbers of 4 to 50. The mean coexpression index decreased greatly when the domain size increased from 2 to 20 neighbor genes. Beyond 20 neighboring genes, the decrease of the coexpression level was slower, and this trend continued for domains of up to 50 neighboring genes. A. Plot based on the expression data in ES; B. Plot based on the expression data in EB. [file 1471-2164-7-103-S1.ppt]

## Slide 1
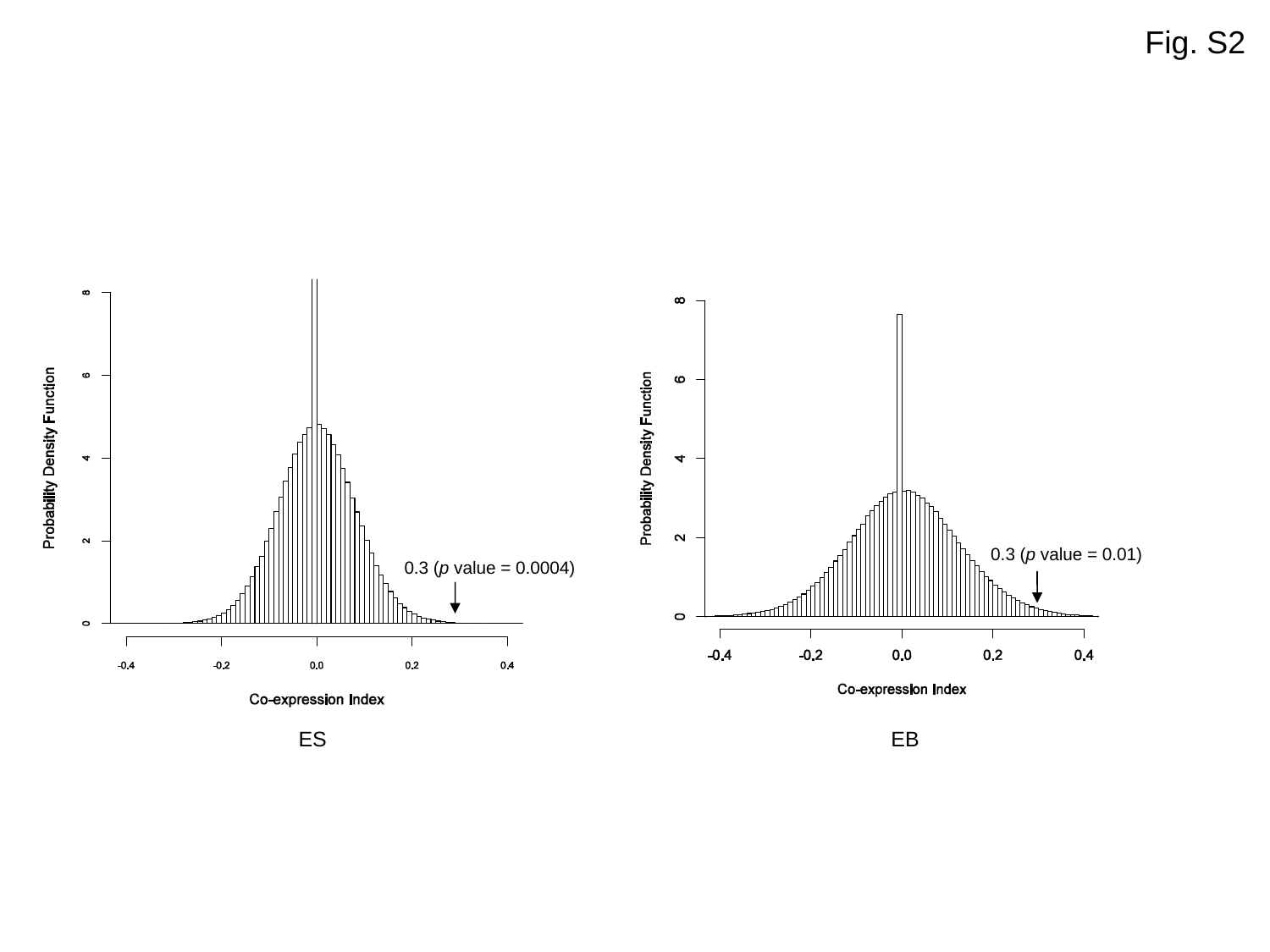

Fig. S2
0.3 (p value = 0.01)
0.3 (p value = 0.0004)
ES
EB

Supplement: Additional File 2 — Supplementary Figure S2 (Supplementary Fig S2 Distribution of co-exp index.ppt). Monte-Carlo distribution of the coexpression index generated from 10,000 randomized genome data. The coexpression index was calculated in the sliding window of 20 neighboring genes. The distribution allows the determination of the P value of the coexpression index. A. By the expression data from ES; the P value for the coexpression index threshold 0.3 is 0.0004. B. By the expression data from EB; the P value for the co-expression index threshold 0.3 is 0.01. [file 1471-2164-7-103-S2.ppt]

## Slide 1
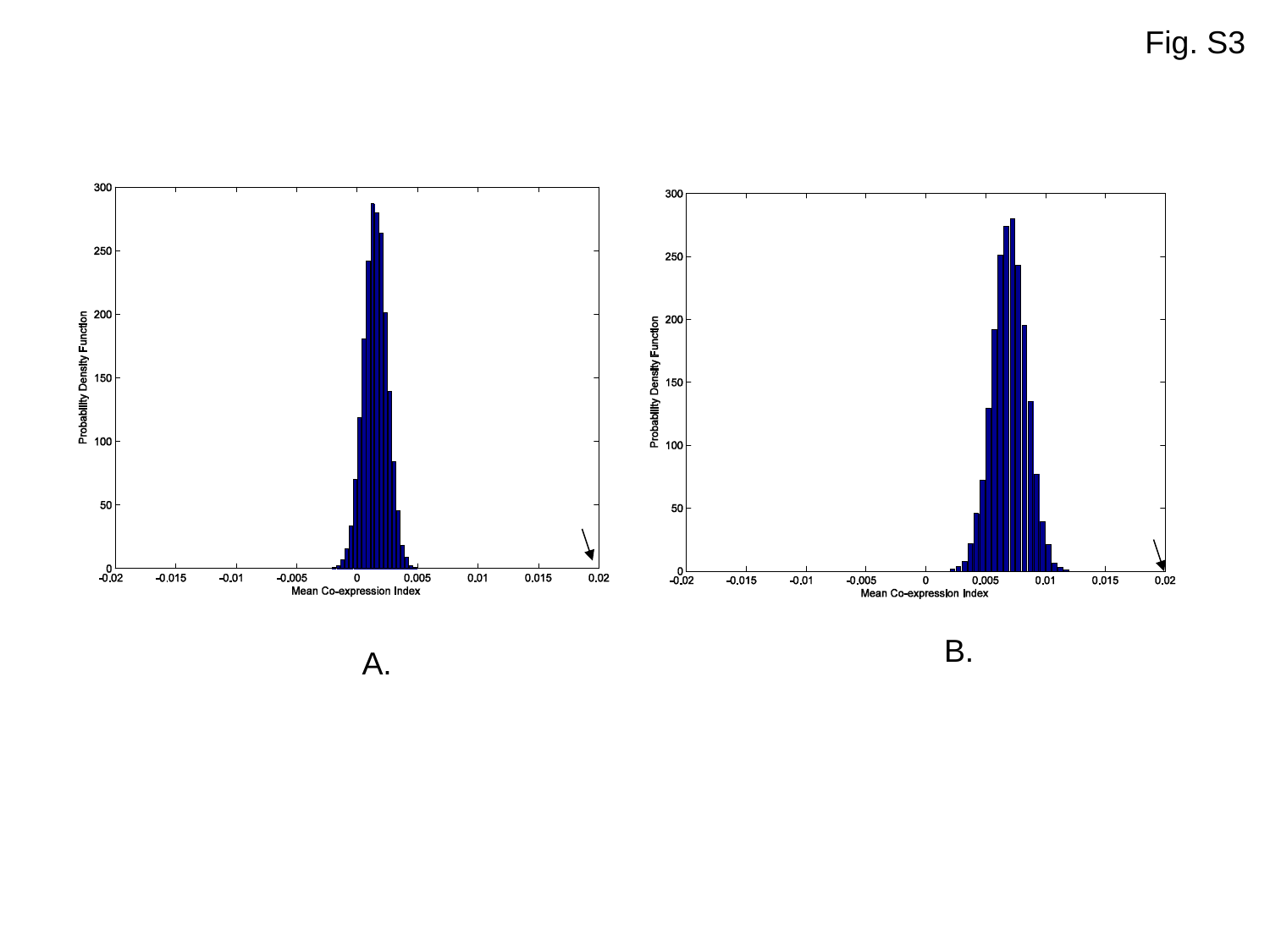

Fig. S3
B.
A.

Supplement: Additional File 3 — Supplementary Figure S3 (Supplementary Fig S3 Distribution of mean co-exp index.ppt). Monte-Carlo distribution of the mean coexpression index generated from 10,000 randomized genome data. The mean coexpression index was calculated in the sliding window of 20 neighboring genes. The mean coexpression index calculated from the real genome data set is marked with an arrow. The distribution allows determination of the P value of the mean co-expression index. A. By the expression data in ES; the mean coexpression index of the real expression data is 0.026 (P < 0.00001). B. By the expressed data in EB; the mean coexpression index from the real expression data is 0.021 (P < 0.00001). [file 1471-2164-7-103-S3.ppt]

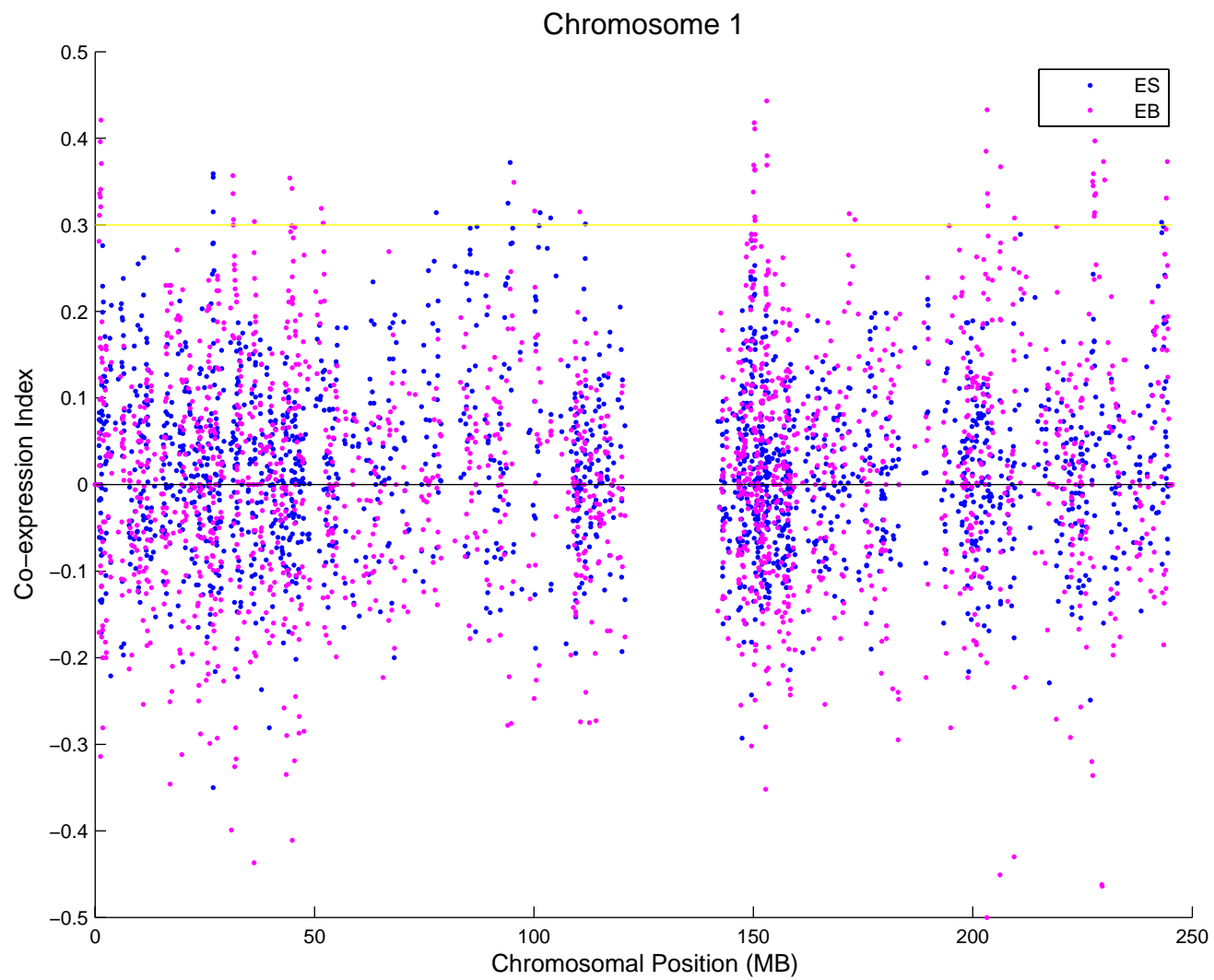

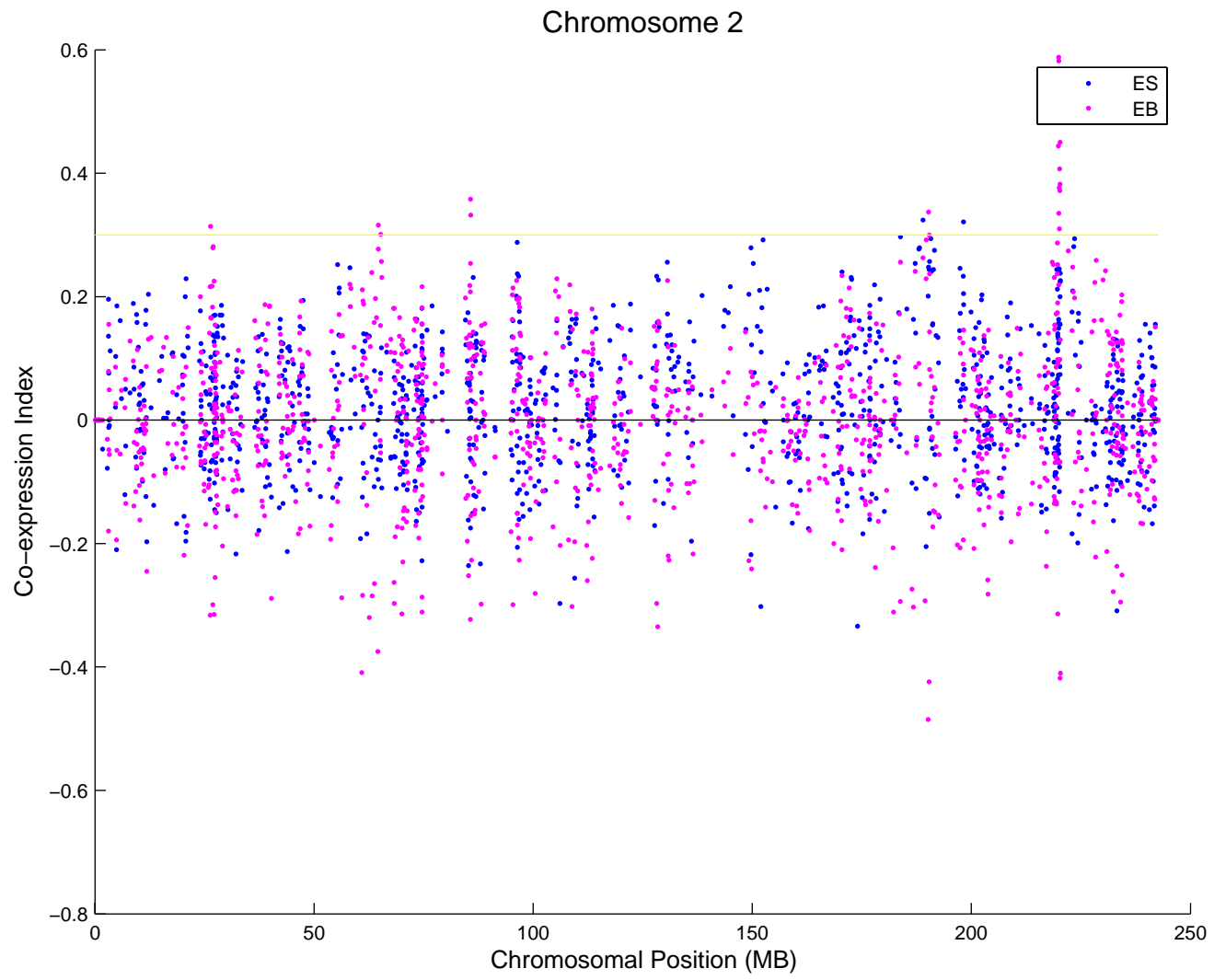

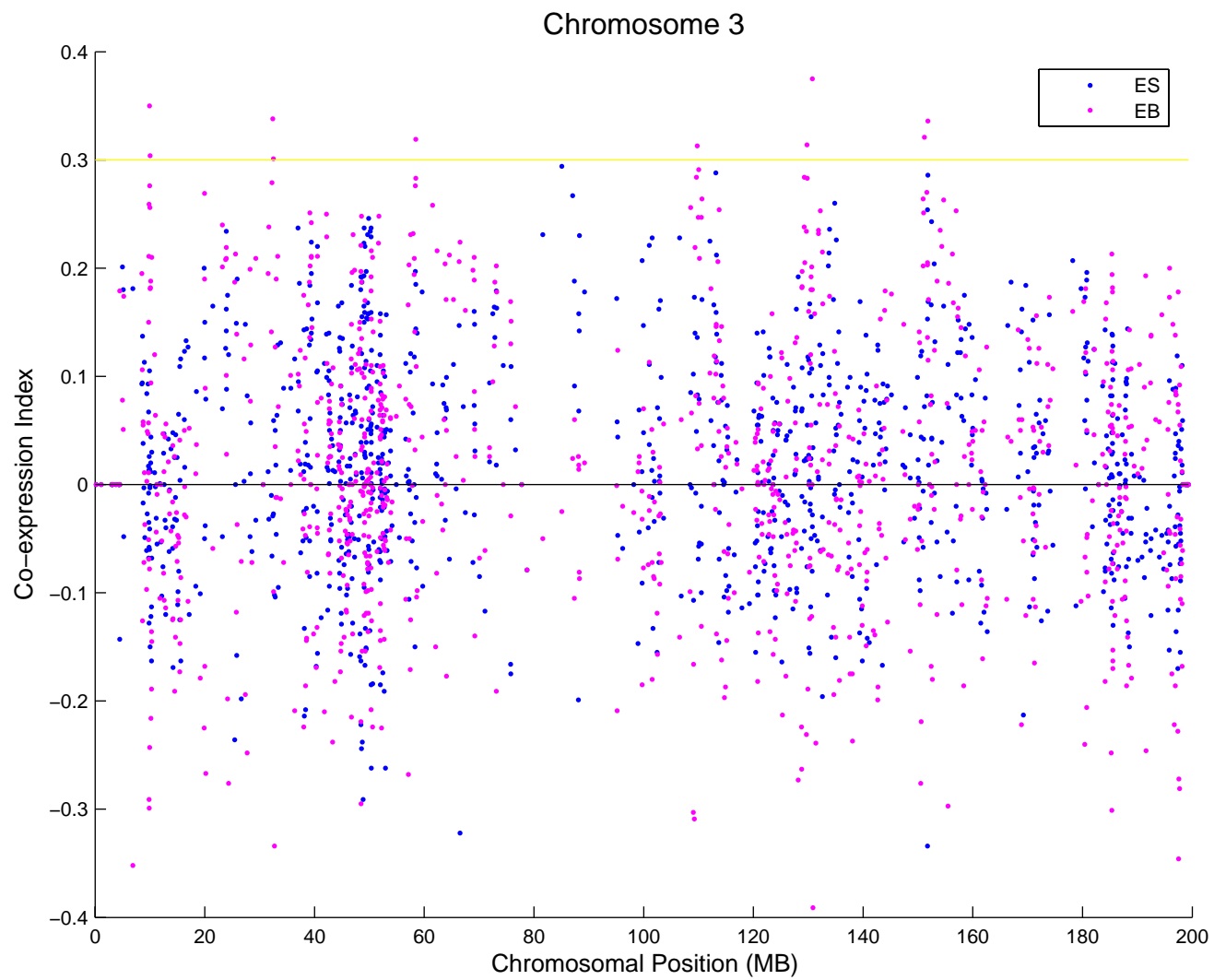

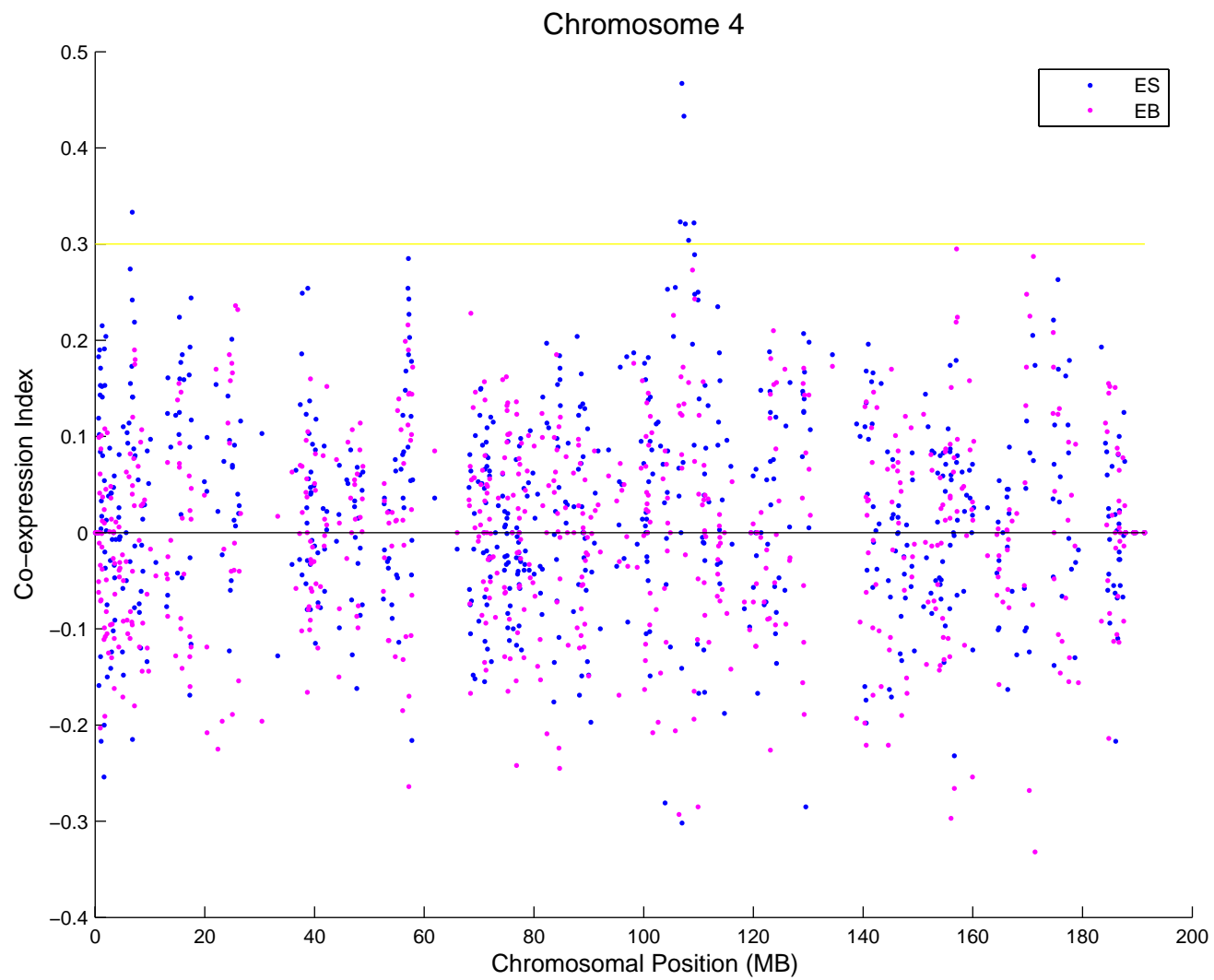

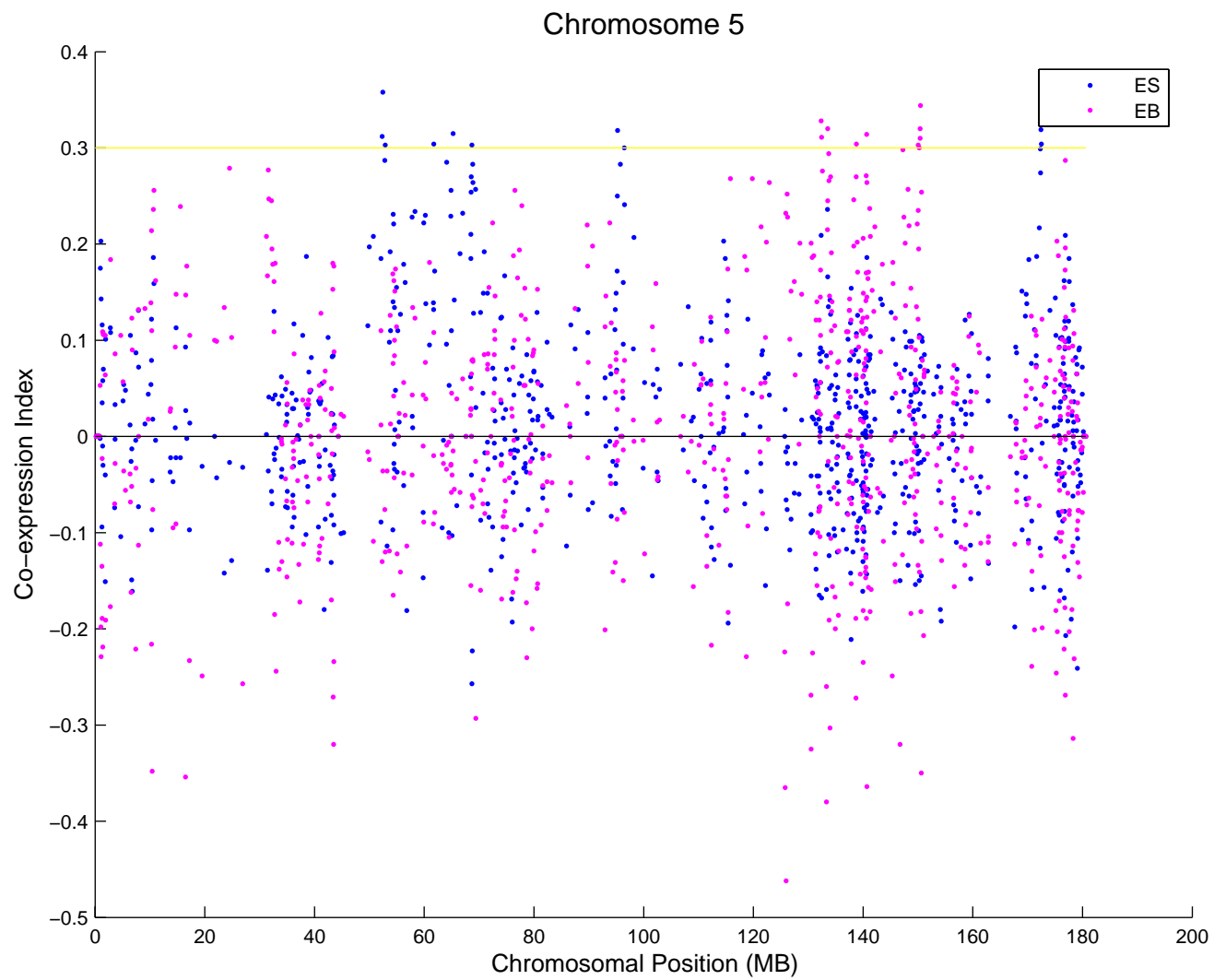

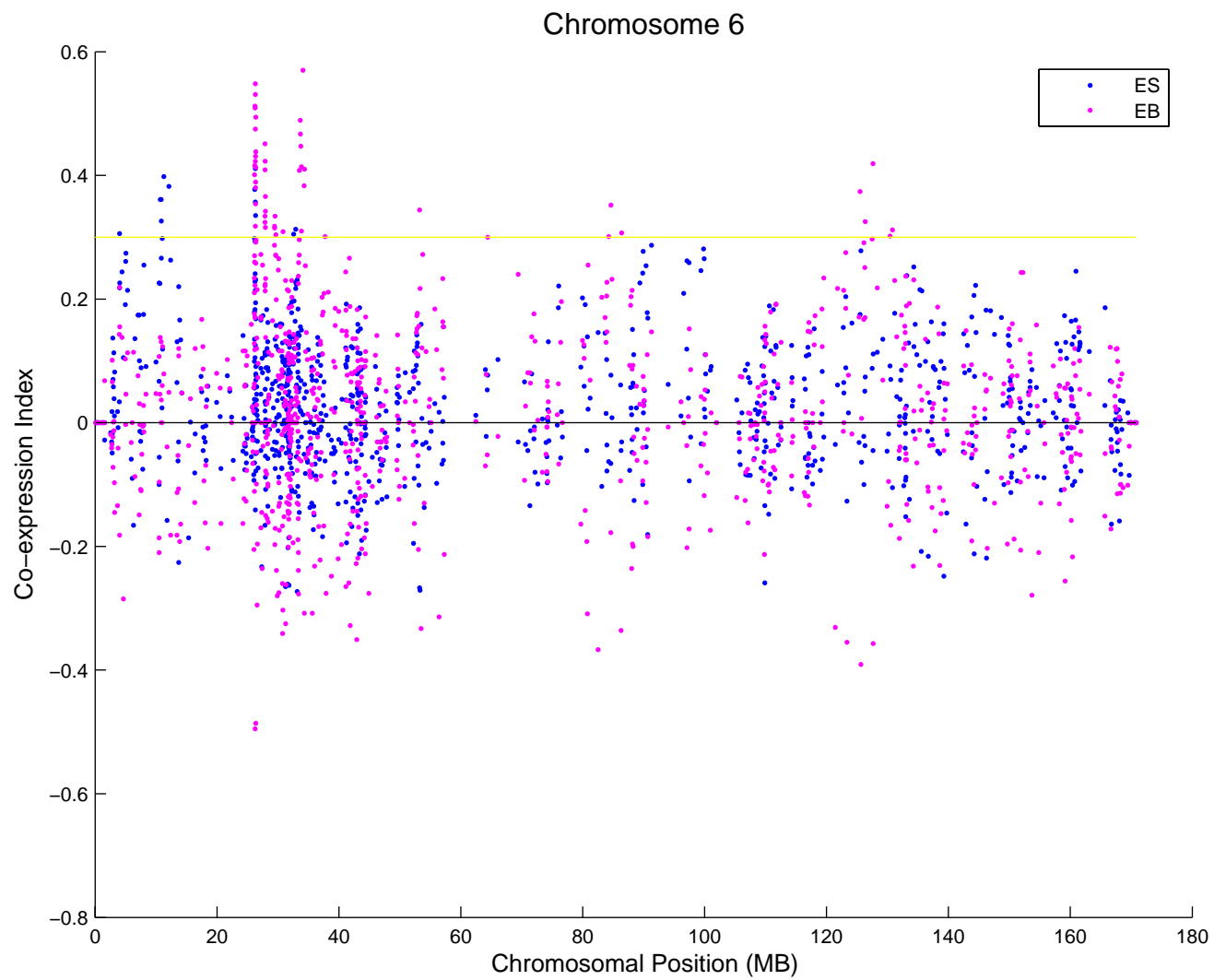

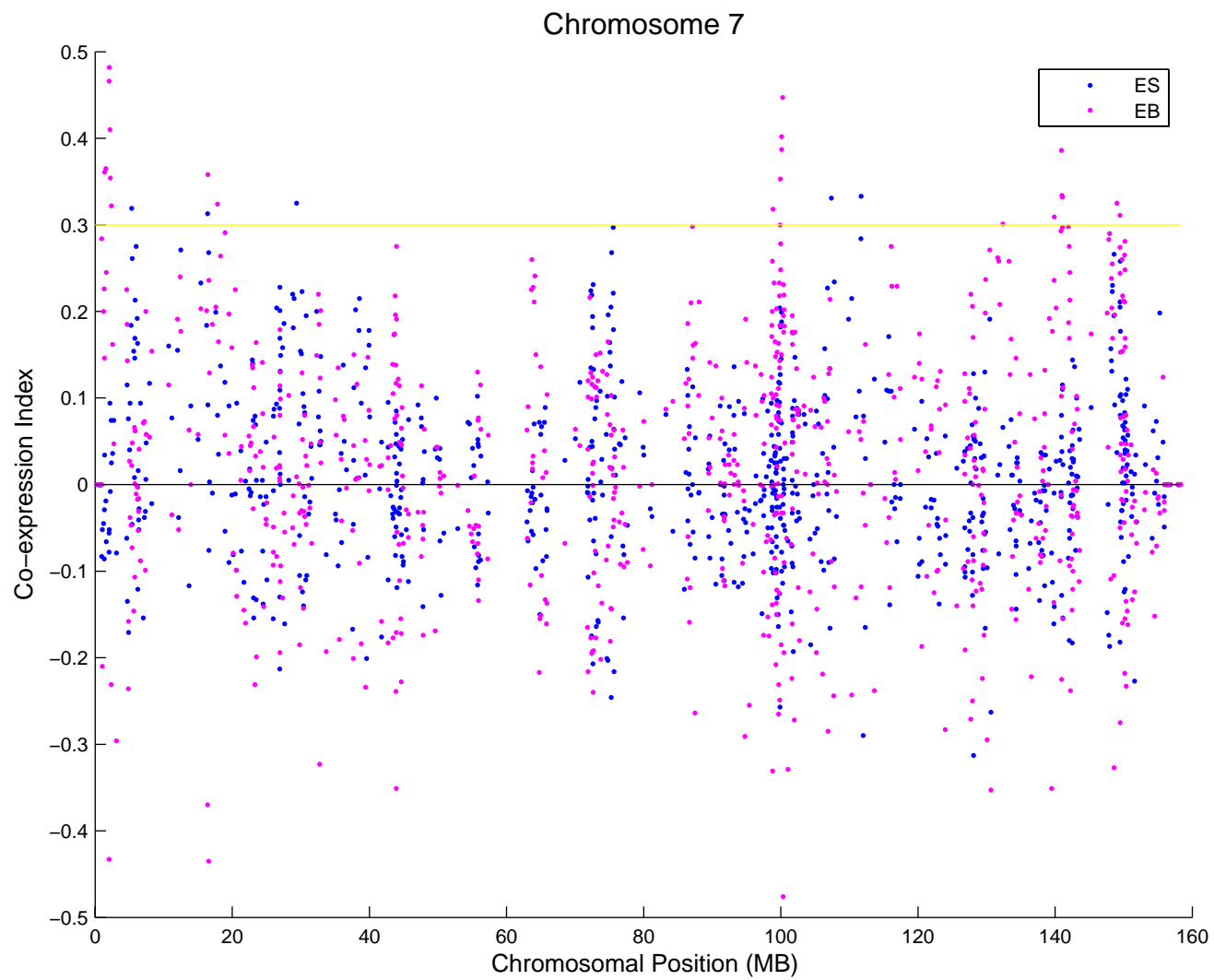

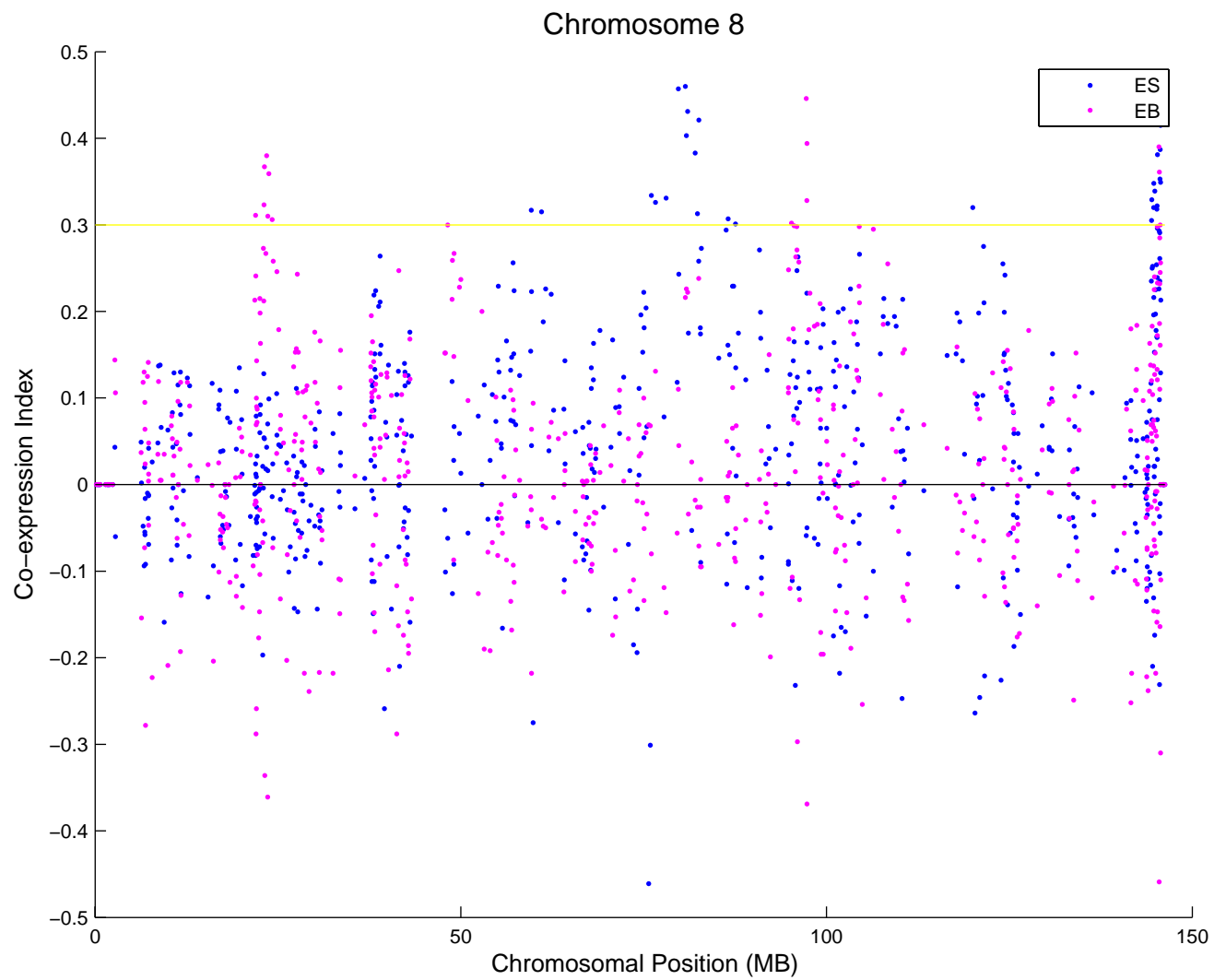

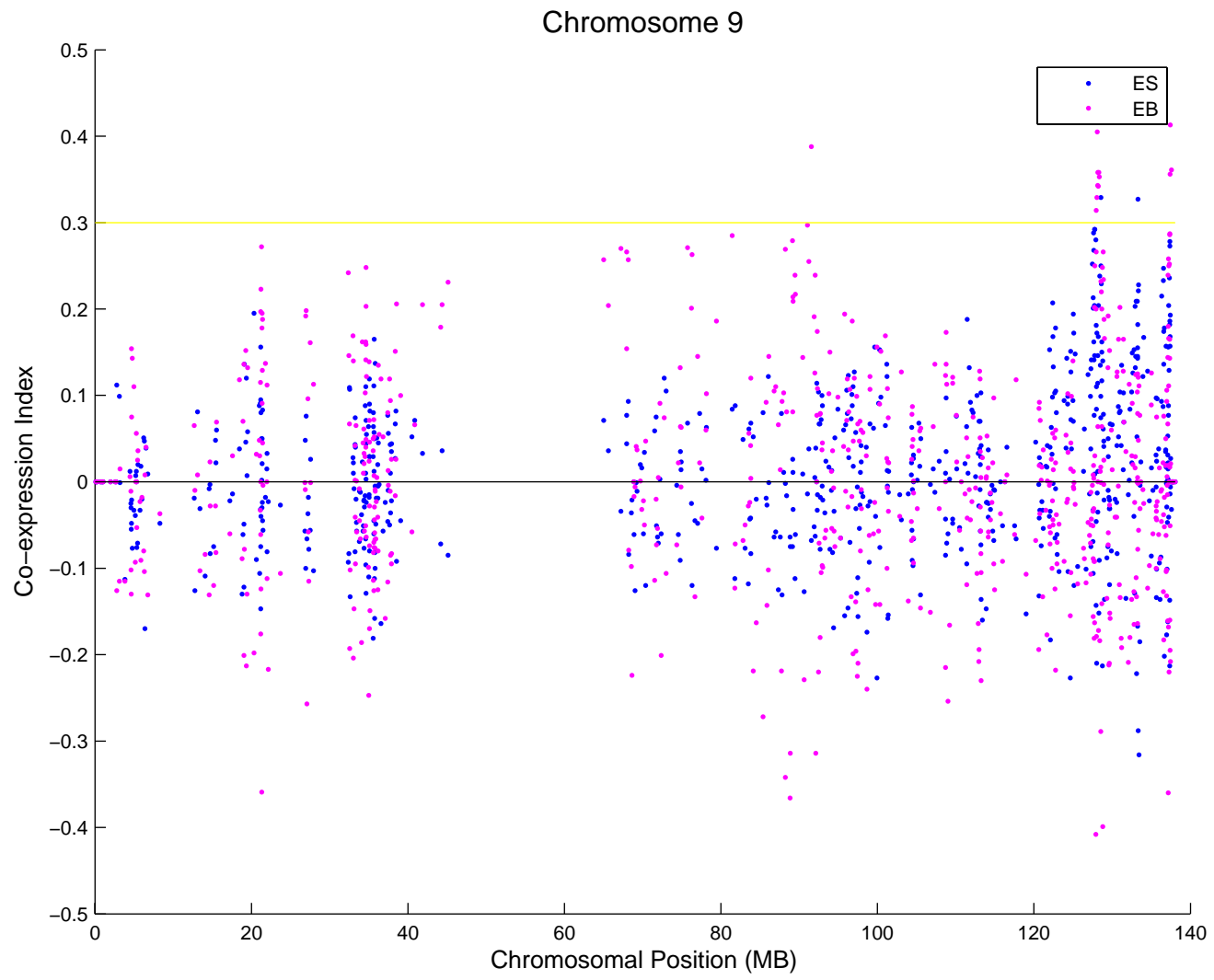

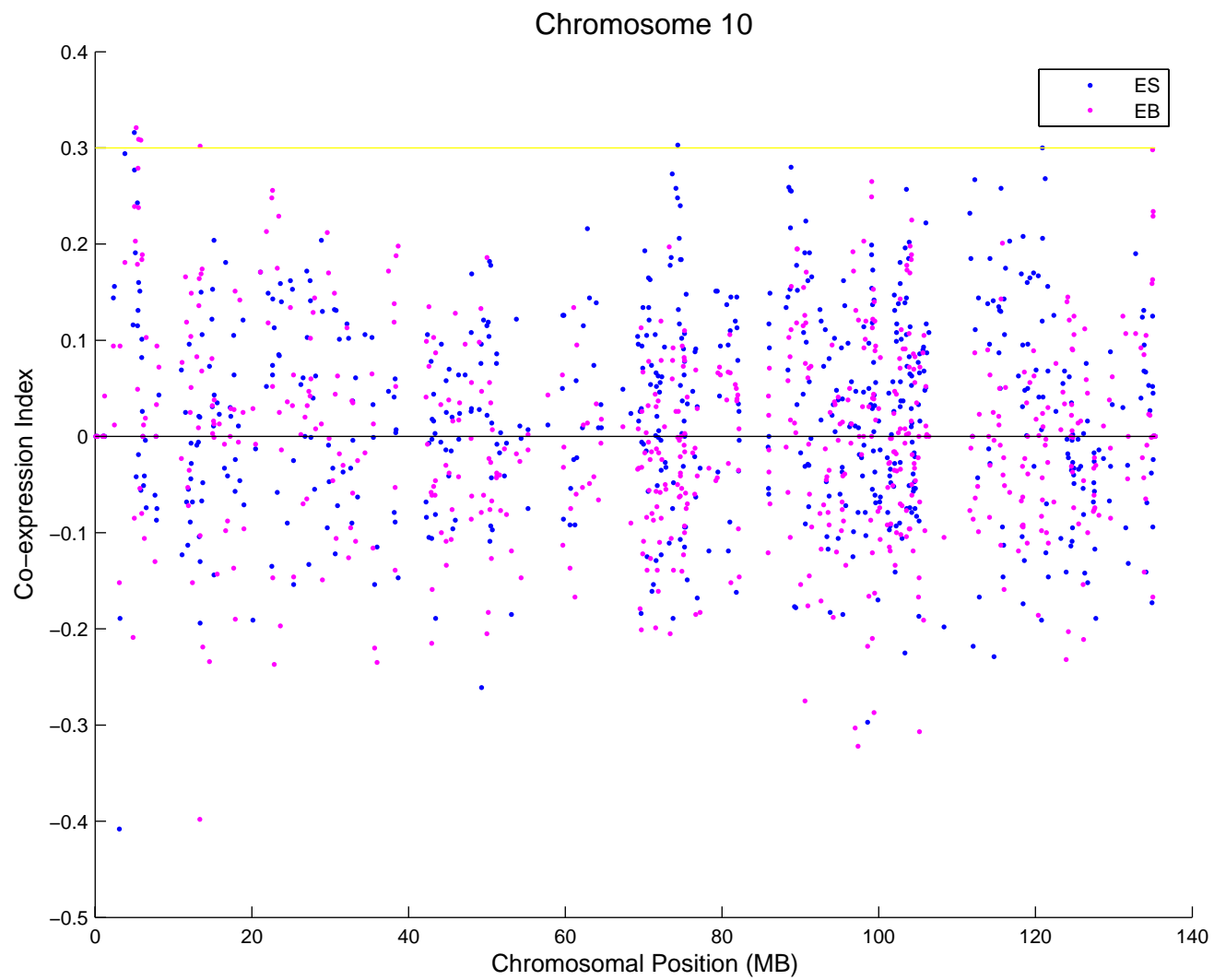

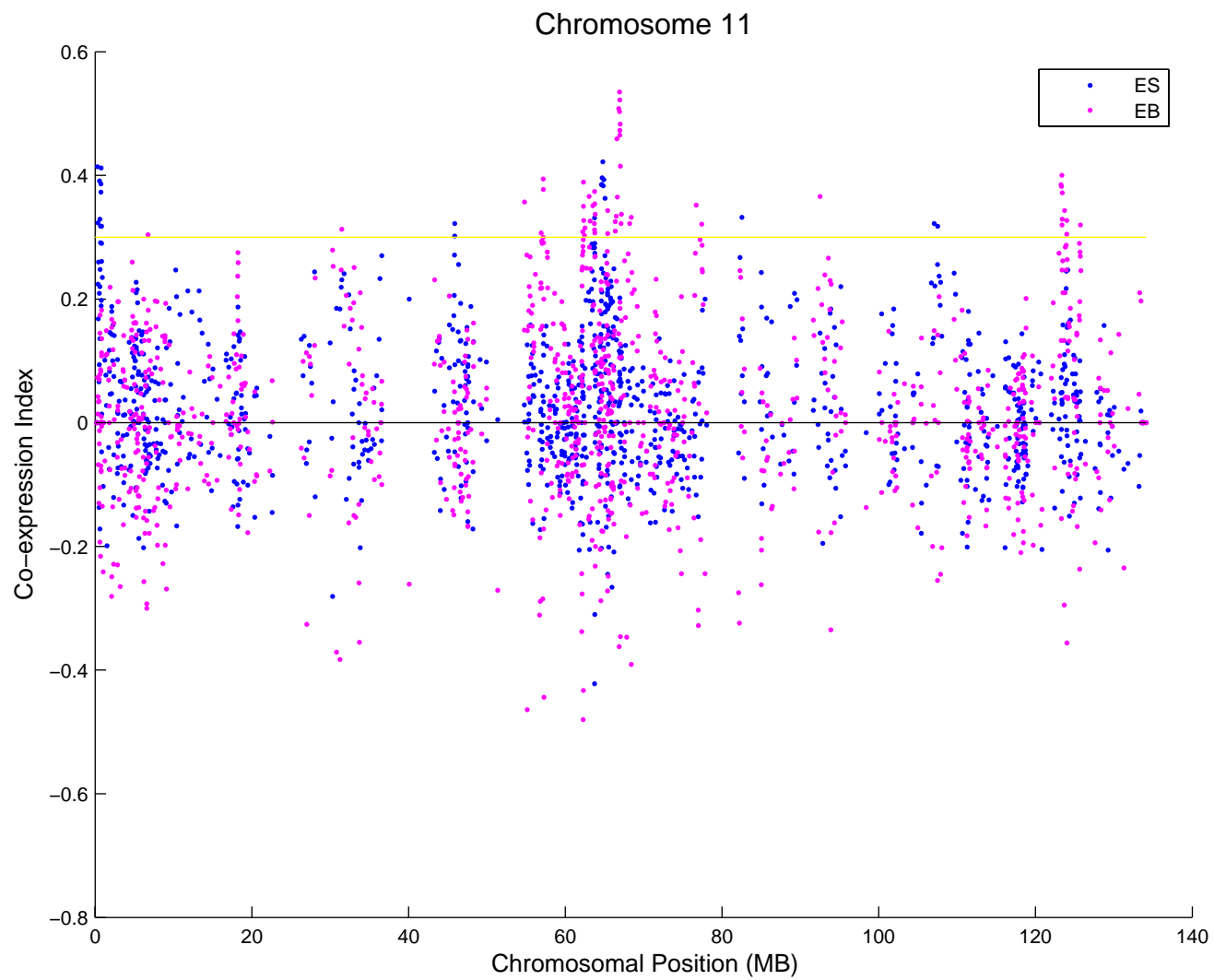

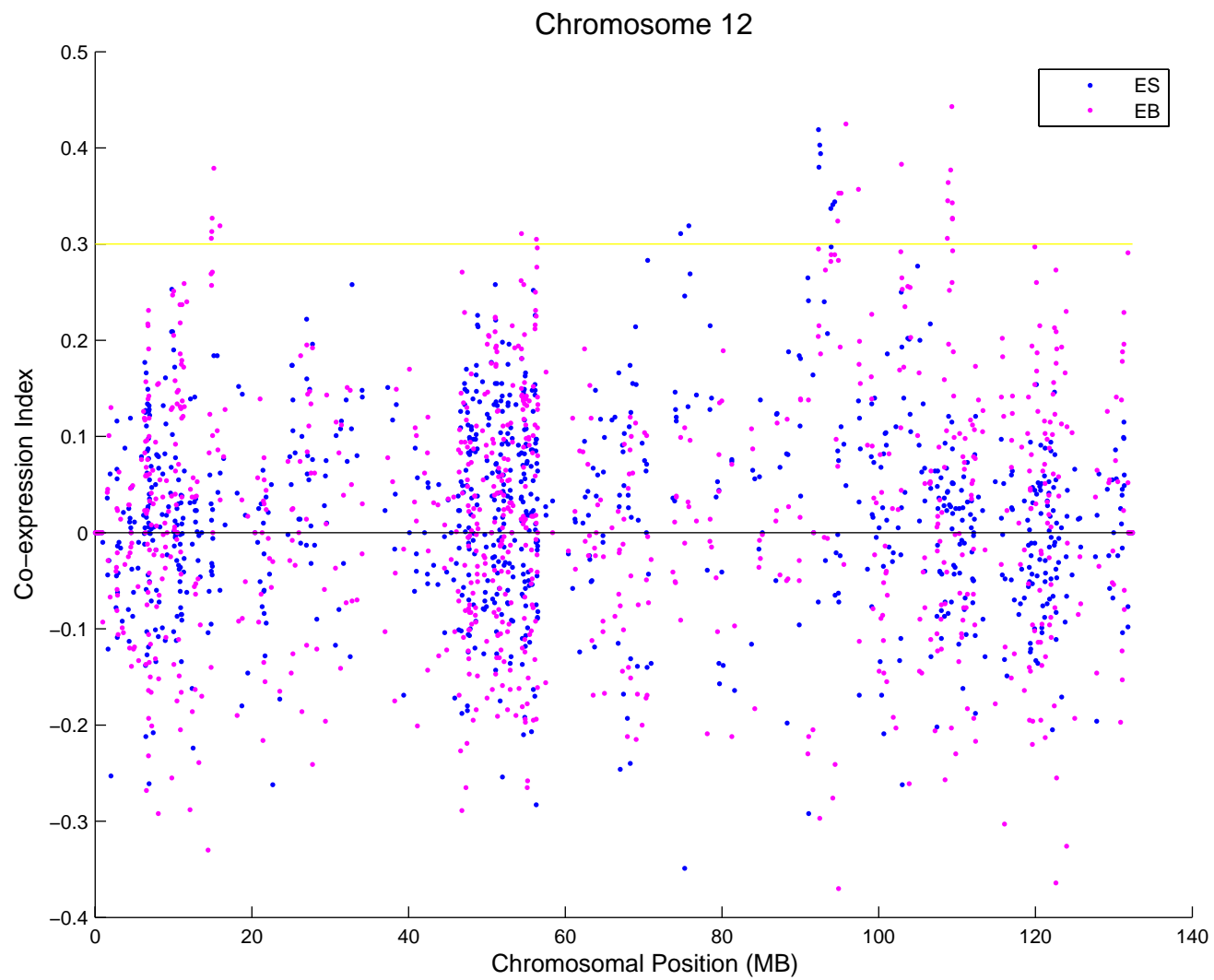

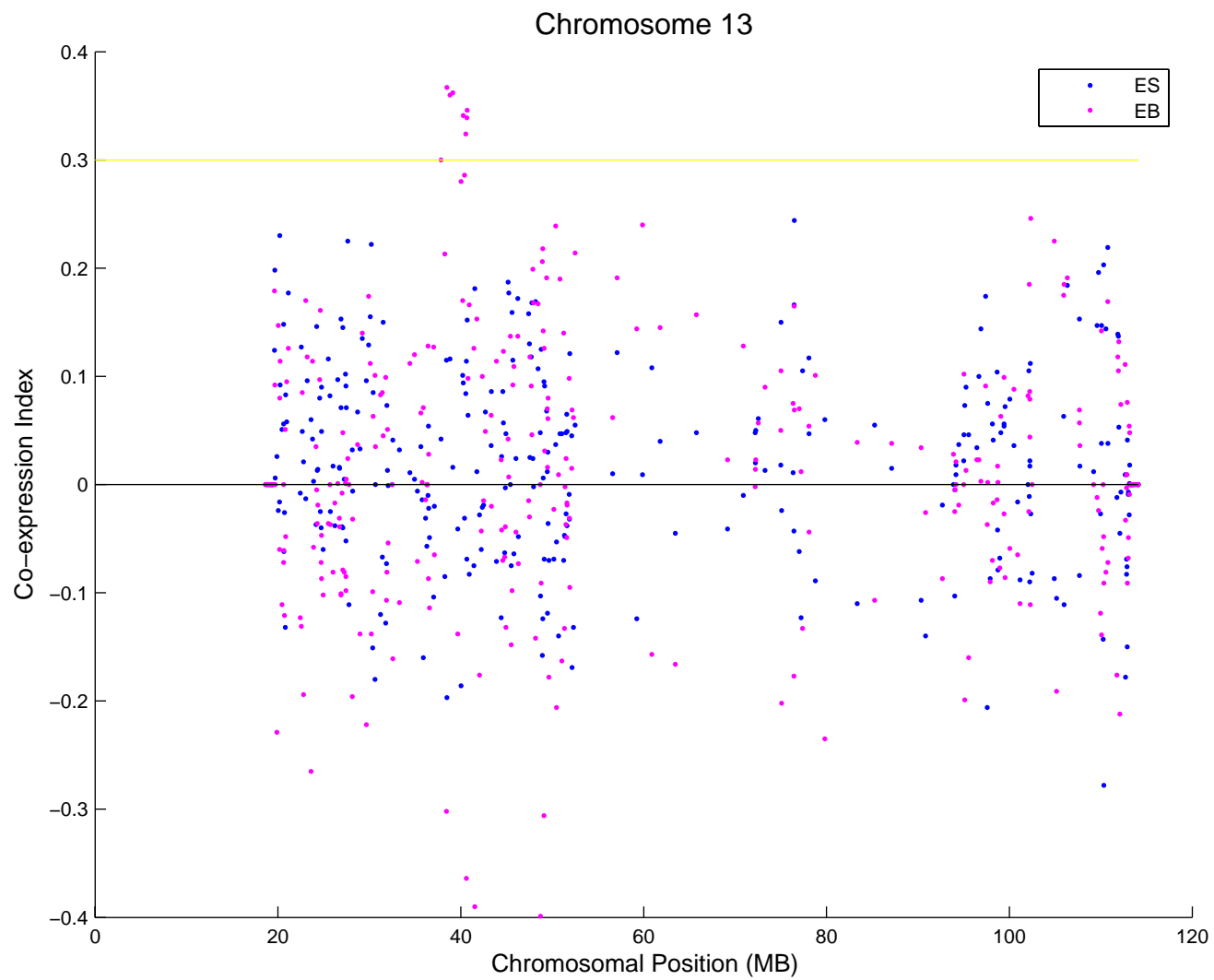

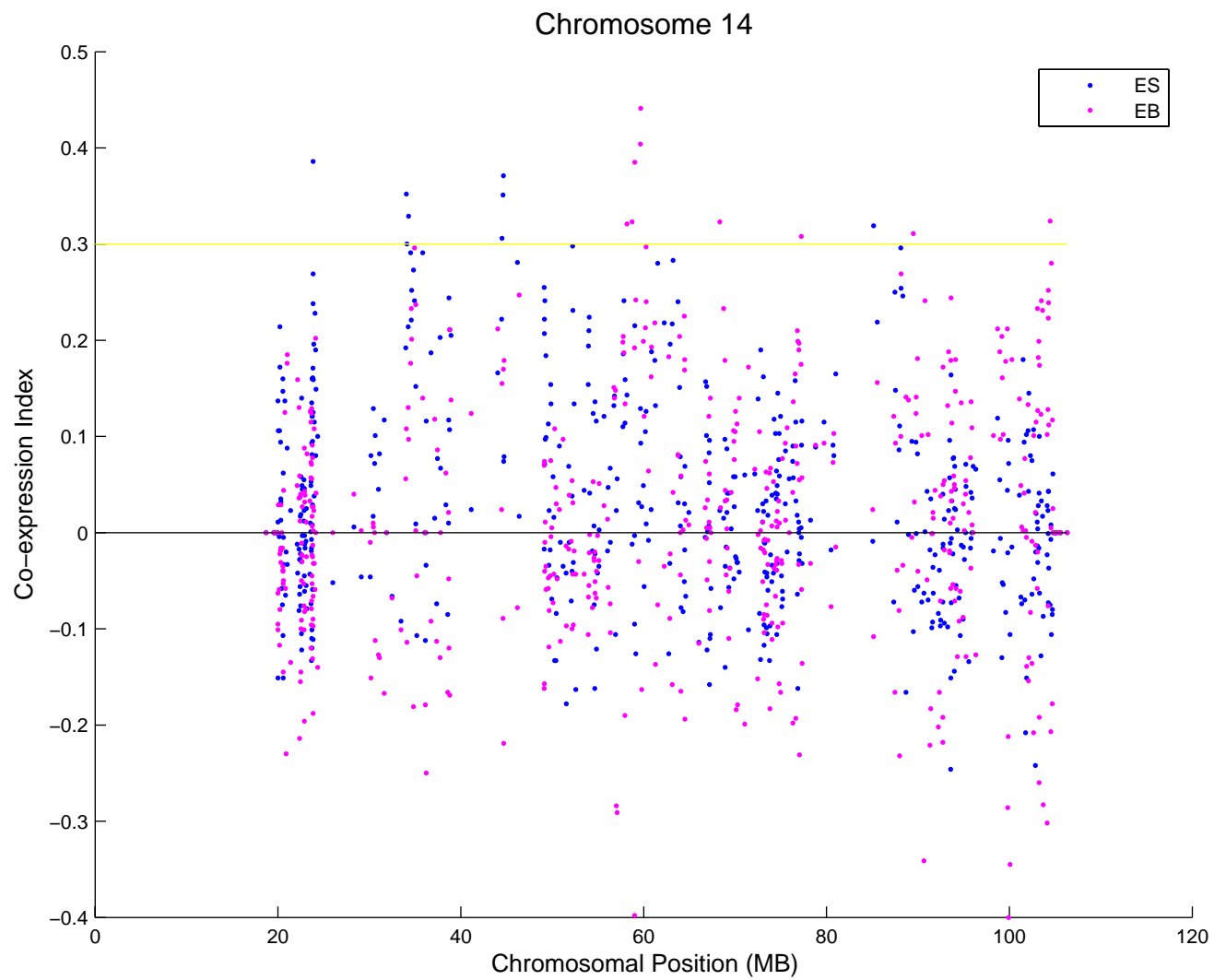

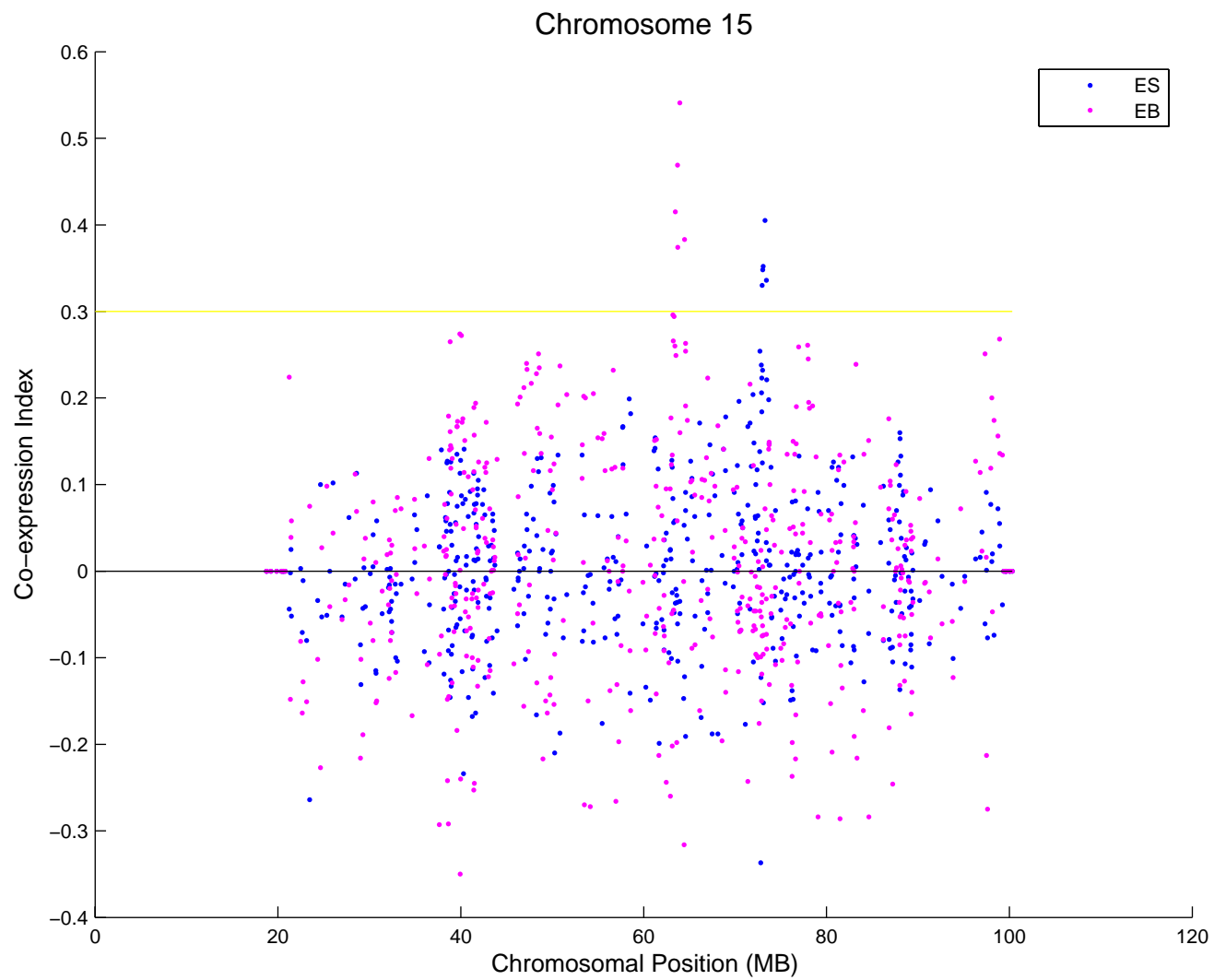

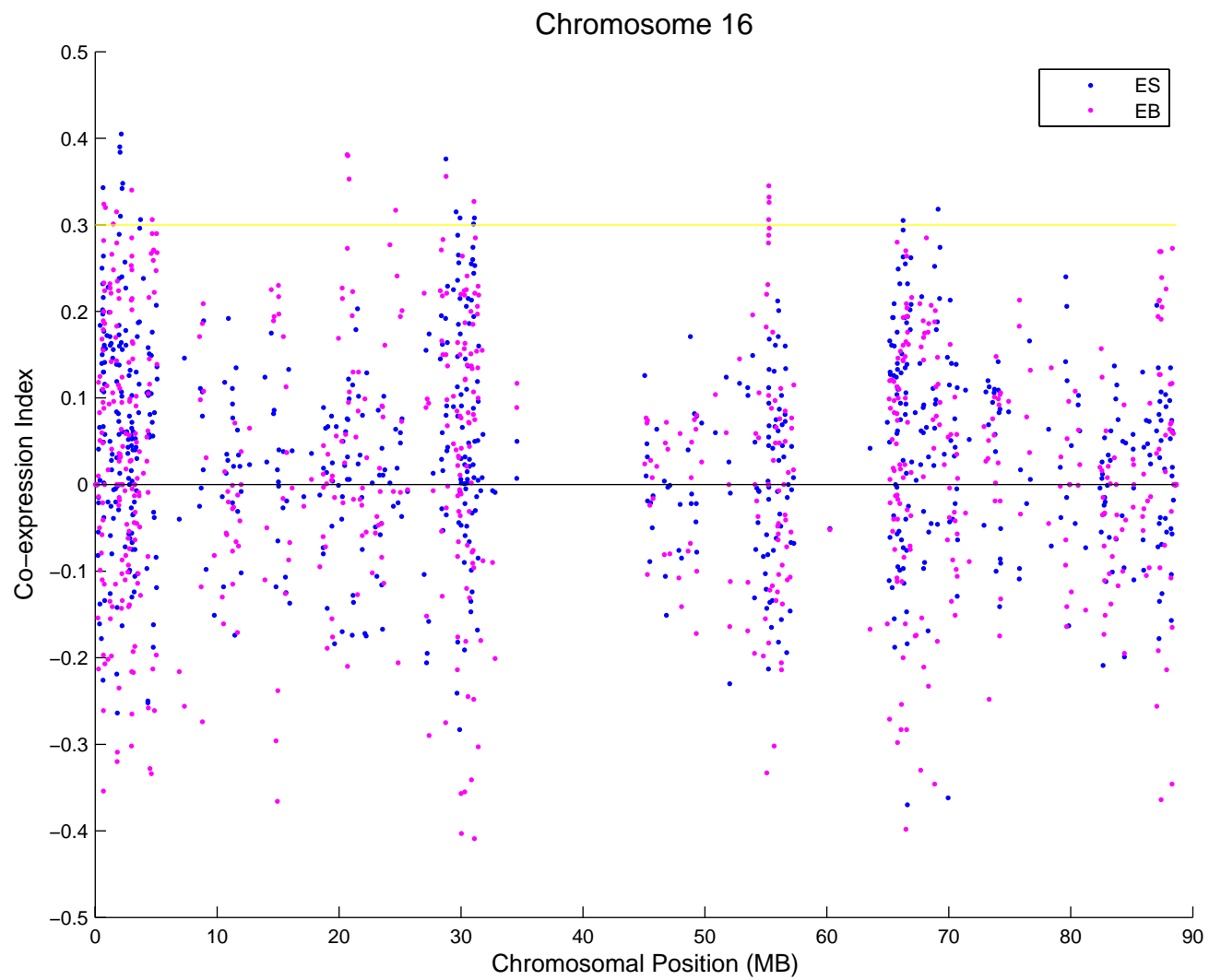

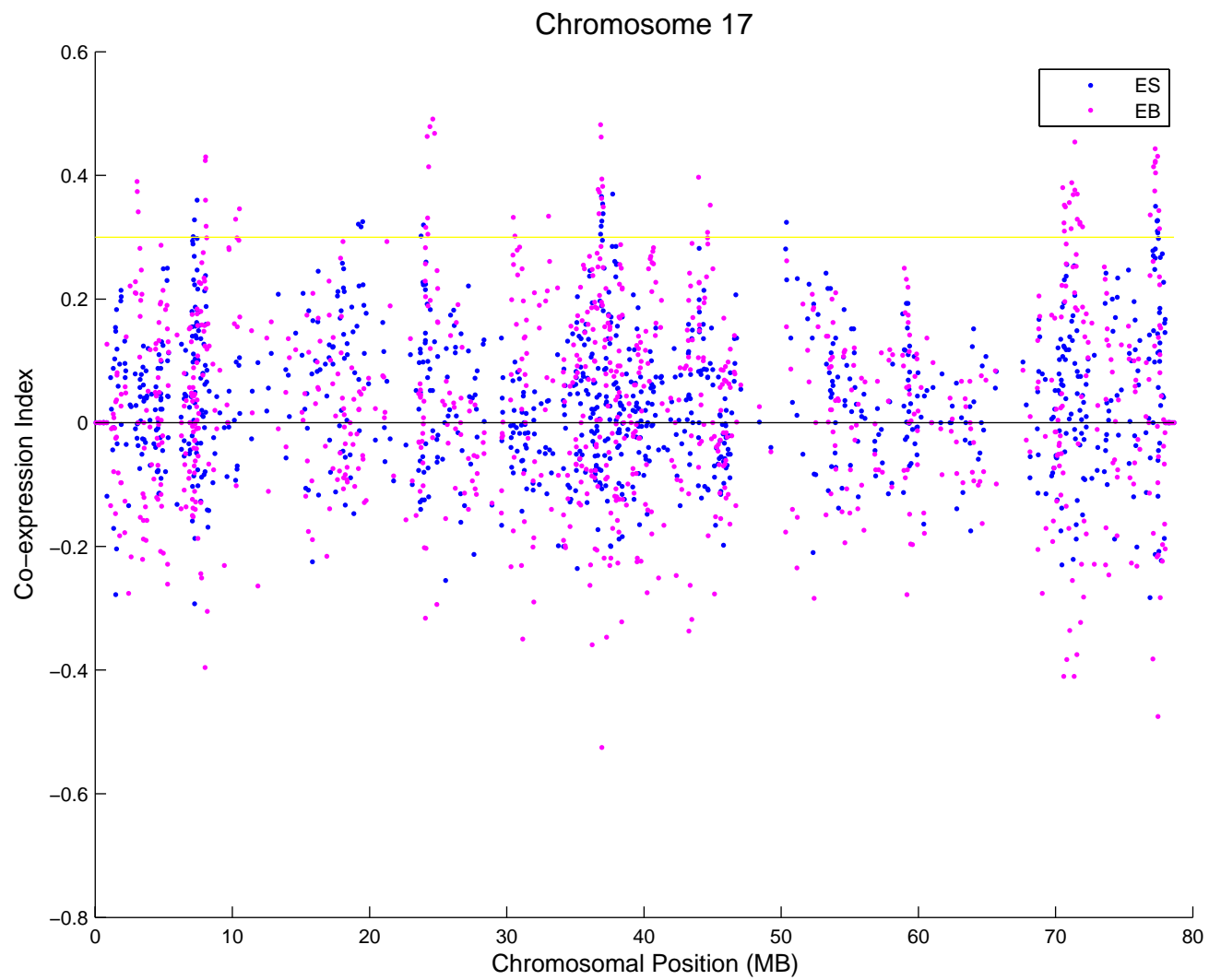

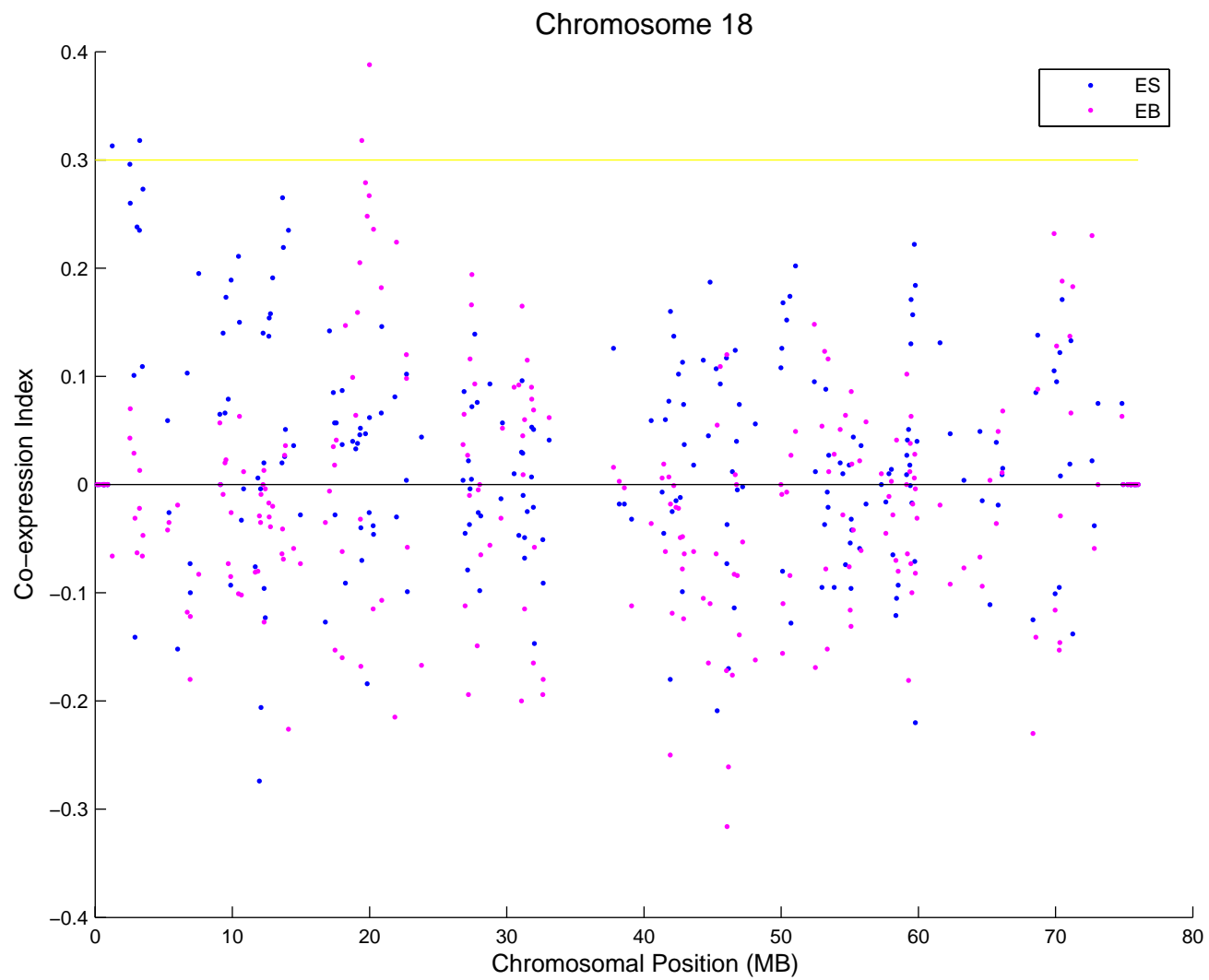

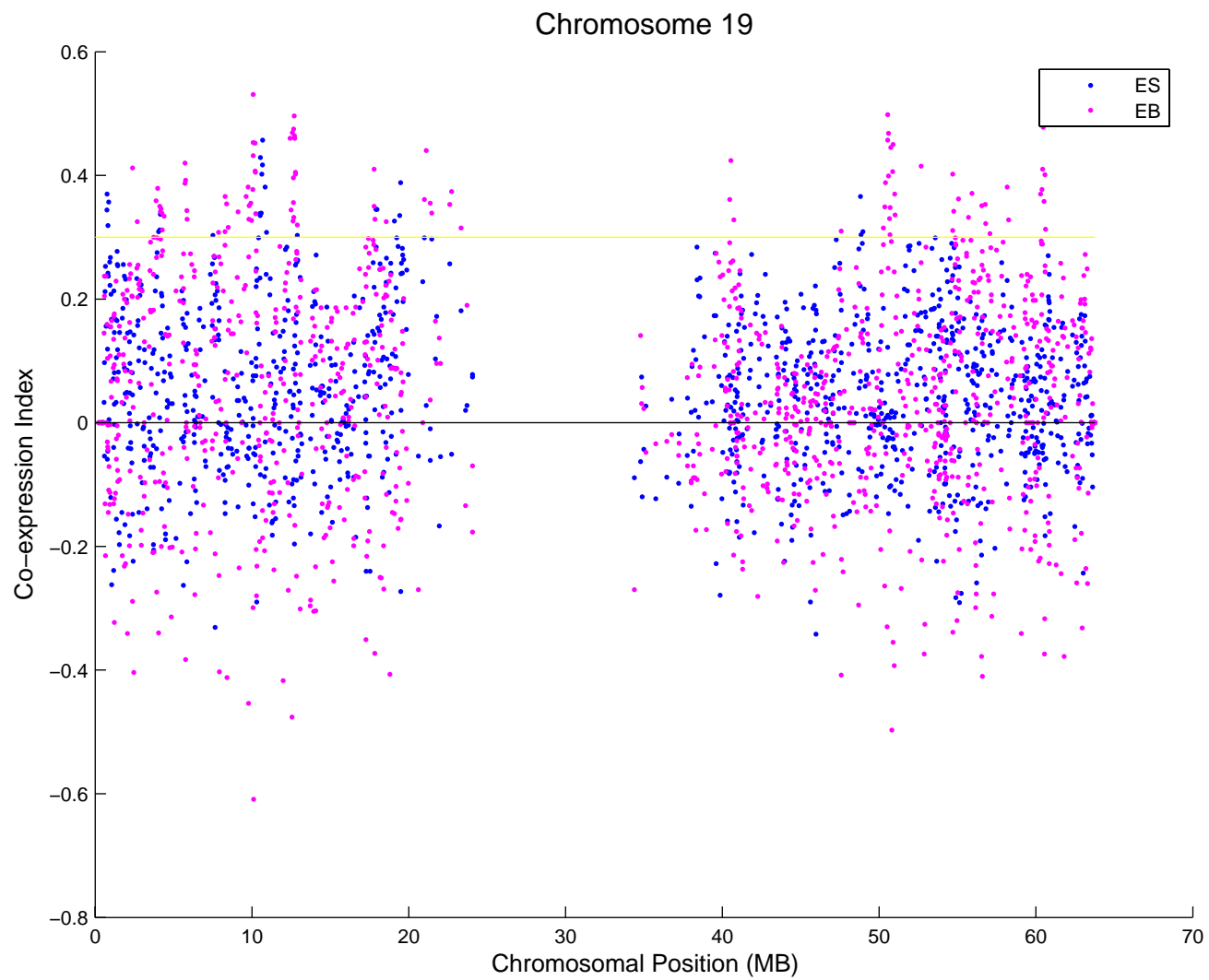

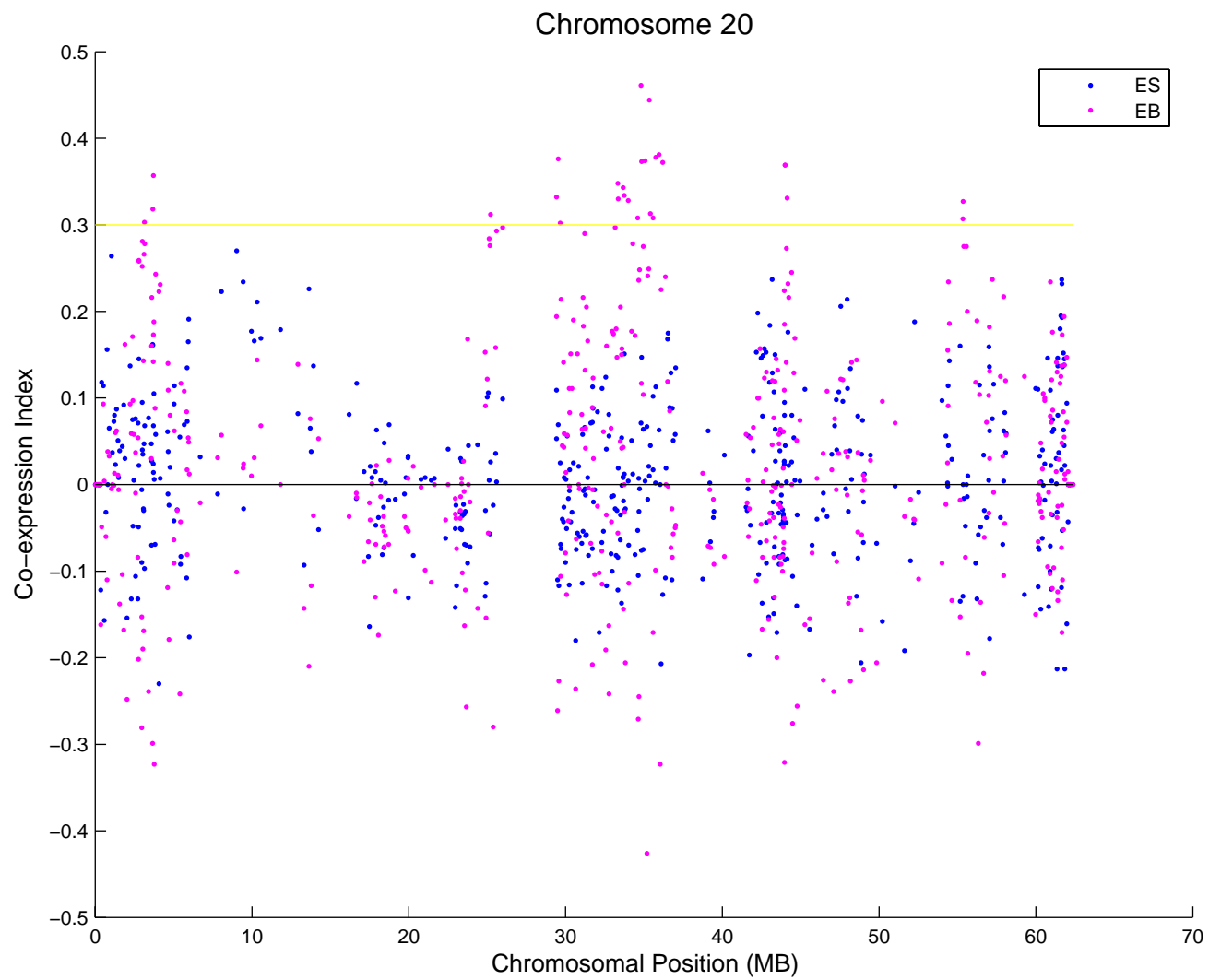

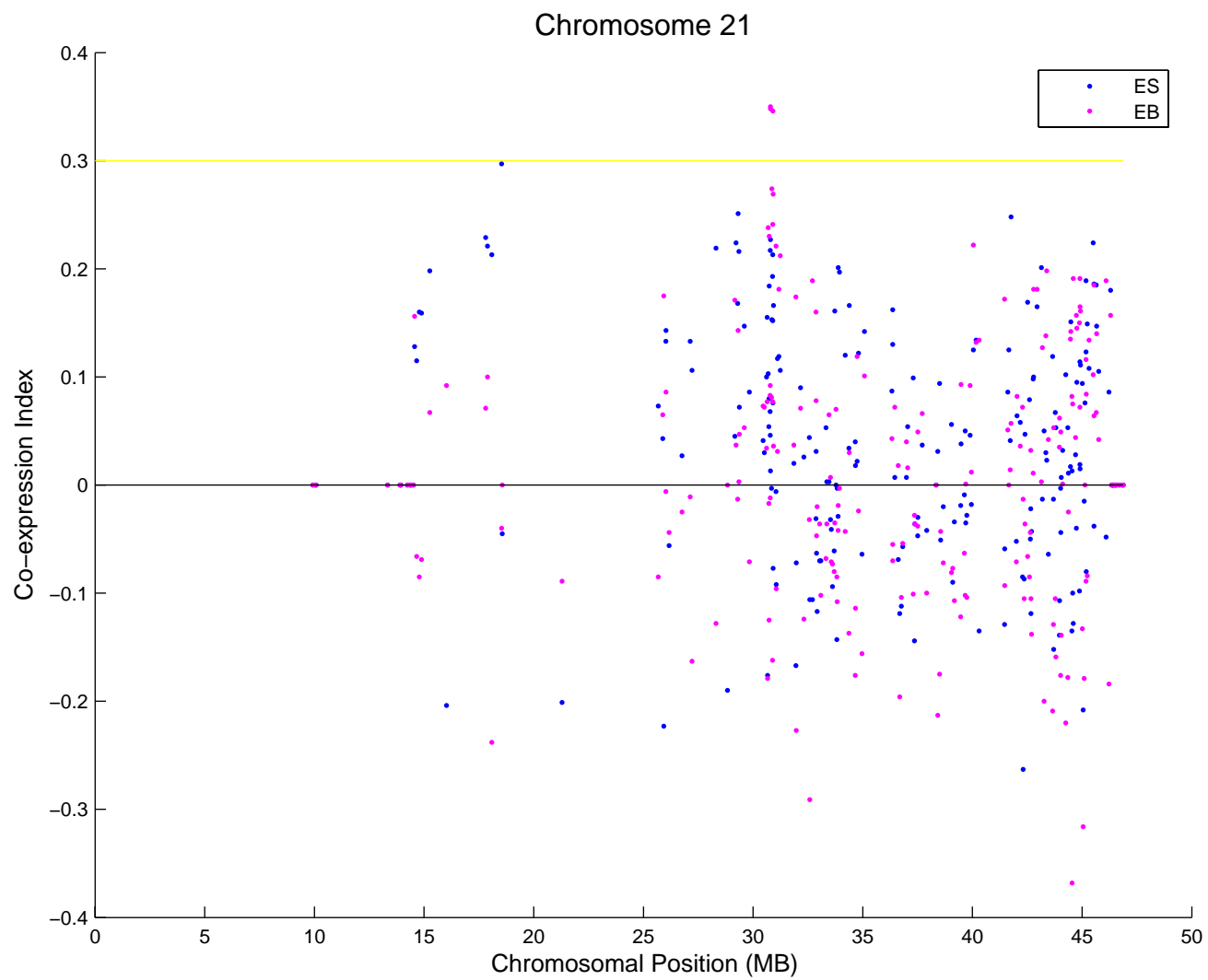

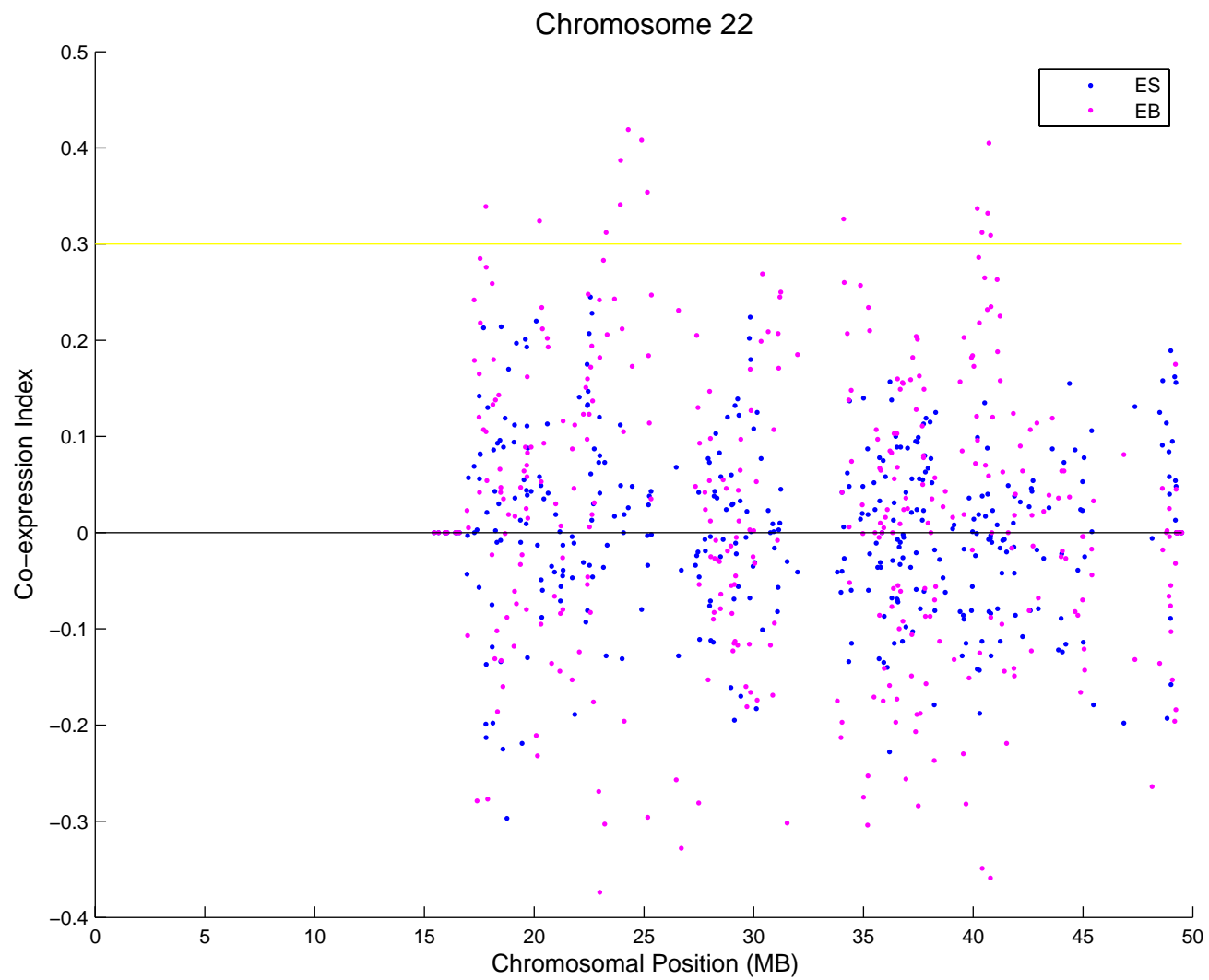

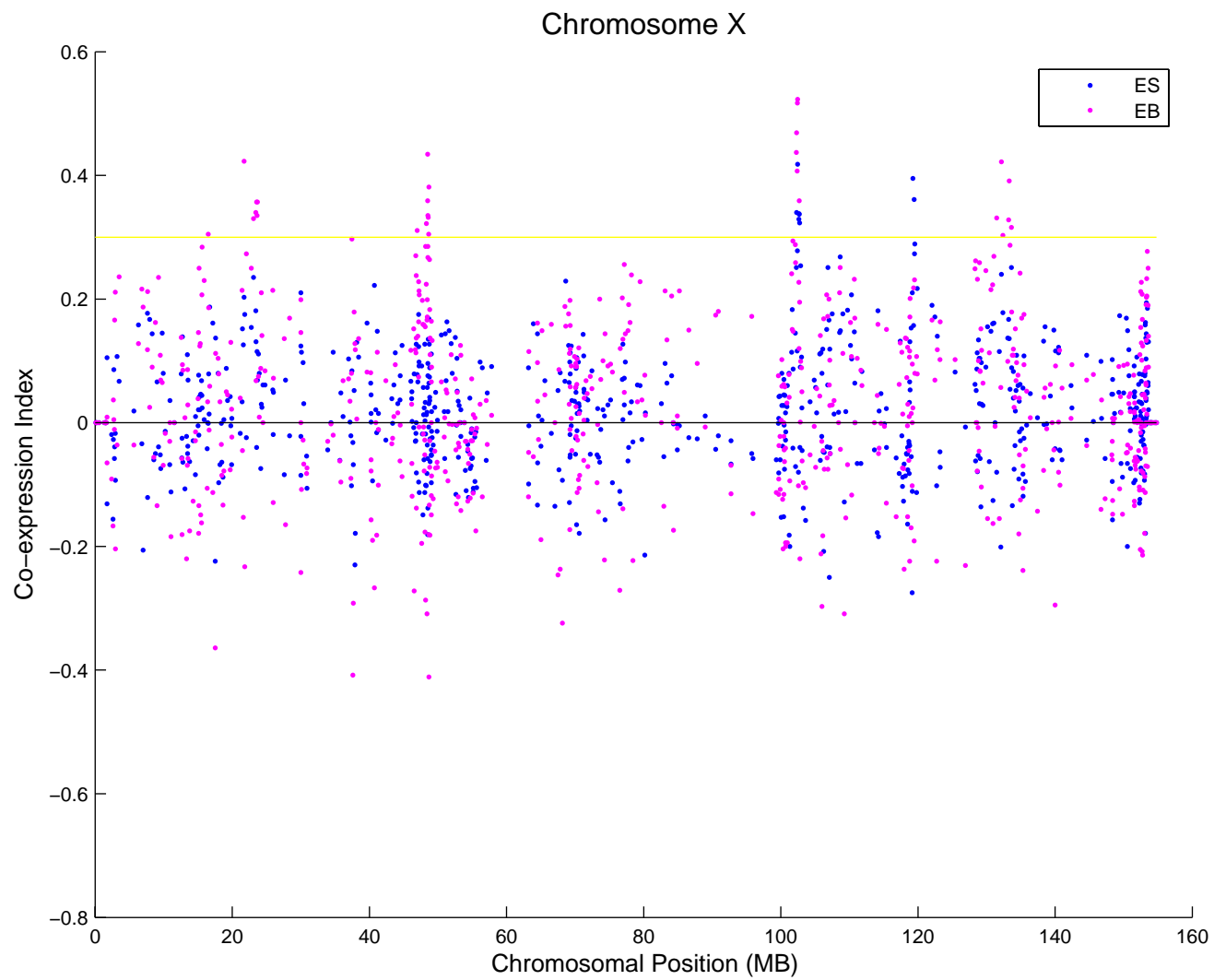

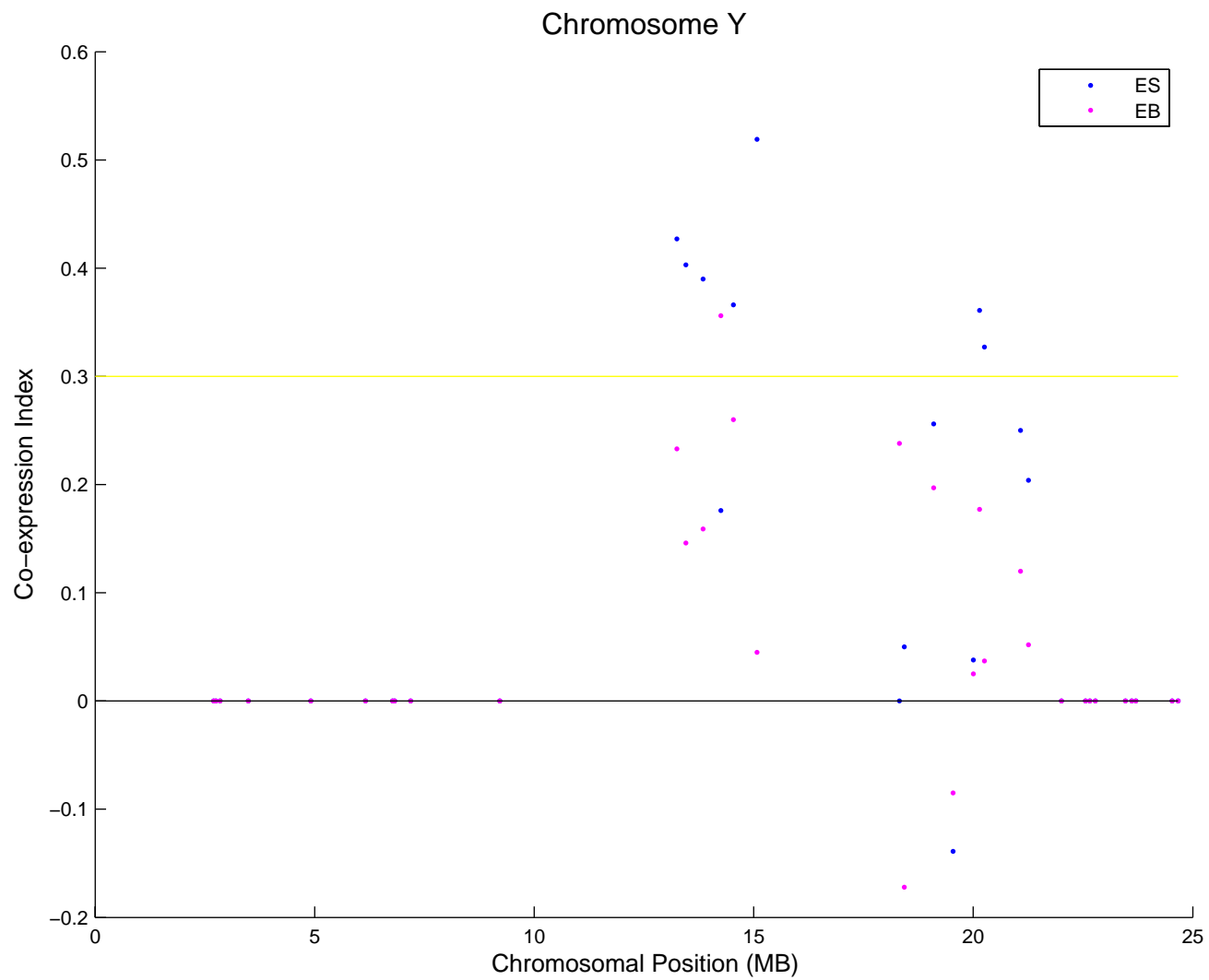

Supplement: Additional File 4 — Supplementary Figure S4 (Supplementary Fig S4 Transcriptome map of each chromosome.pdf). Comparative transcriptome map of human ES and EB on each chromosome of the genome. The transcriptome map shows the coexpression index value of genes that are displayed according to the position along the chromosome (horizontal axis). Each dot represents a gene expressed in ES (blue color) or EB (red). The yellow line represents the threshold of the coexpression index (0.3). The coexpression index was calculated as the average Person's correlation coefficient between the expression level of a given gene with that of every neighboring gene (10 up- and 10 downstream). The statistical significance of the co-expression pattern was confirmed by Monte-Carlo simulation (see Methods). [file 1471-2164-7-103-S4.pdf]

## Slide 1
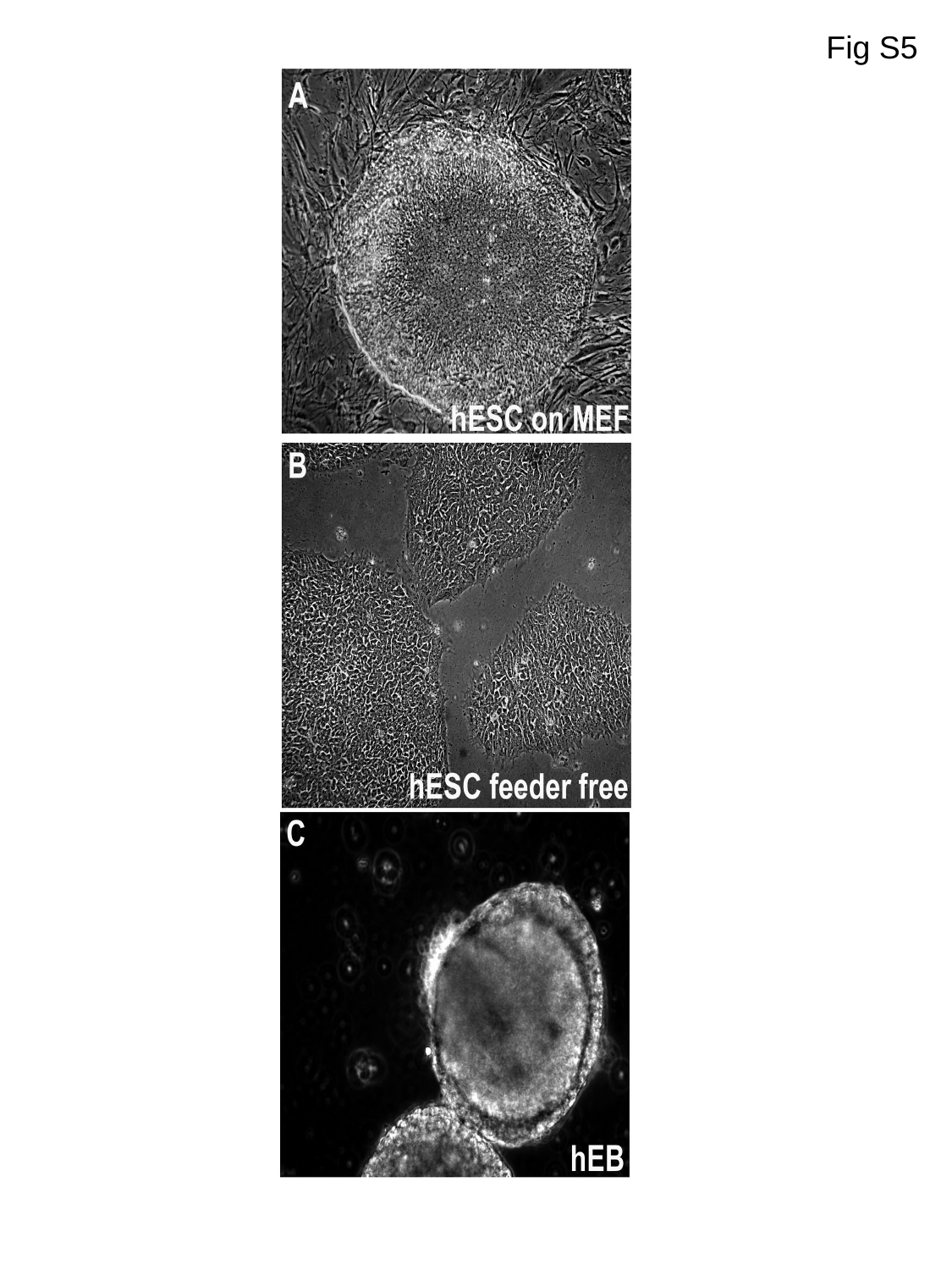

Fig S5

Supplement: Additional File 5 — Supplementary Figure S5 (Supplementary Fig S5 ES and EB photo.ppt). Undifferentiated human ES cell lines cultured on inactivated MEF (A) or grown in a feeder free condition (B). To differentiate ES cells, embryoid bodies were generated by growing ES cells in ultralow attachment plates to form floating spheres (C). [file 1471-2164-7-103-S5.ppt]
