# Supplementary material for: Transcriptome coexpression map of human embryonic stem cells
Source: BMC Genomics. 2006 May 2;7:103. doi: 10.1186/1471-2164-7-103 (PMC1523211; doi:10.1186/1471-2164-7-103)

**Supplementary Tab. S1.** Cell lines used in this study and their relatedness to each other in ES and EB. The correlation matrix was calculated by Pearson’s correlations coefficient of expression profiles.


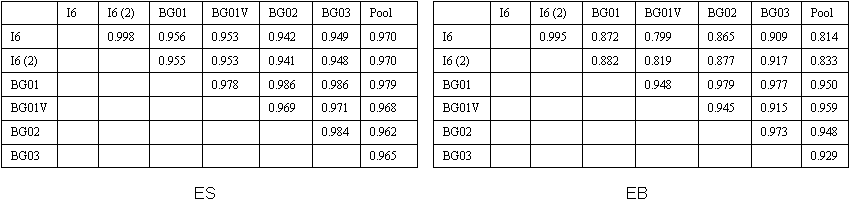

Supplement: Additional File 6 — Supplementary Table S1 (Supplementary Table S1 Cell lines and relatedness.doc). Cell lines used in the study and their relatedness to each other in ES and EB. The correlation matrix was calculated by Pearson's correlations coefficient of expression profiles. [file 1471-2164-7-103-S6.doc]
