# Supplementary material for: Transcriptome coexpression map of human embryonic stem cells
Source: BMC Genomics. 2006 May 2;7:103. doi: 10.1186/1471-2164-7-103 (PMC1523211; doi:10.1186/1471-2164-7-103)
Supplement: Additional File 7 — Supplementary Table S2 (Supplementary Table S2 Coexpression chrom domains in ES.doc). List of coexpression chromosomal domains identified in ES at the co-expression index threshold 0.3 and the window size of 20, and associated GO terms (Fisher P ≤ 0.05) [file 1471-2164-7-103-S7.doc]

Supplementary Table S3. List of coexpression chromosomal domains identified in ES cells at the coexpression index threshold 0.3 and the window size of 20 genes. *gneg - Giemsa negative bands, gpos - Giemsa positive bands, acen - centromeric regions, Gvar - variable length heterochromatic regions. ** P value is derived by ANOVA test between ES and EB. *** the genes are in the from5’ to 3’ order as on the chromosome

| **Gene ID** | **Gene Symbol** | **Chromosome** | **Chromosome position** | **Chromosomal band** | **Freq in cytogeneric pattern*** | **Fold change (ES:EB) **** | ***P* value (ES - EB)** | **Co-Exp Index (ES)** | **Exp_value (ES)** | **Co-Exp Index (EB)** | **Exp_value (EB)** | **Gene in Domain***** |
| --- | --- | --- | --- | --- | --- | --- | --- | --- | --- | --- | --- | --- |
| 54707 | FLJ10349 | 1 | 26886768 | 1p35.3 | gneg 1; gpos 13; | 1.41 | 0.2428651 | 0.355 | 0.221 | -0.083 | -0.406 | AIM1L; MGC33414; LIN28; DHDDS; HMGN2; RPS6KA1; SMARCF1; FLJ20477; ZDHHC18; SFN; FLJ10349; FLJ12455; NR0B2; NUDC; GPR108; FLJ34633; 401945; MGC16491; SLC9A1; WDTC1; DKFZP564D0478 |
| 63906 | FLJ12455 | 1 | 26901121 | 1p35.3-p35.1 | gneg 1; gpos 13; | 1.25 | 0.8970505 | 0.315 | 0.025 | 0.032 | -0.046 | MGC33414; LIN28; DHDDS; HMGN2; RPS6KA1; SMARCF1; FLJ20477; ZDHHC18; SFN; FLJ10349; FLJ12455; NR0B2; NUDC; GPR108; FLJ34633; 401945; MGC16491; SLC9A1; WDTC1; DKFZP564D0478; JFC1 |
| 10726 | NUDC | 1 | 26932365 | 1p35-p34 | gneg 1; gpos 13; | 1.45 | 0.2620365 | 0.359 | 0.213 | -0.099 | -0.39 | DHDDS; HMGN2; RPS6KA1; SMARCF1; FLJ20477; ZDHHC18; SFN; FLJ10349; FLJ12455; NR0B2; NUDC; GPR108; FLJ34633; 401945; MGC16491; SLC9A1; WDTC1; DKFZP564D0478; JFC1; MAP3K6; FCN3 |
| 26009 | ZZZ3 | 1 | 77742210 | 1p31.1 | gpos 17; | 1.68 | 0.0410861 | 0.314 | 0.369 | 0.111 | -0.677 | ACADM; RABGGTB; MSH4; ASB17; SIAT7C; ARHCL1; SIAT7E; 256483; PIGK; AK5; ZZZ3; USP33; FLJ35093; FLJ90637; NEXN; FUBP1; DNAJB4; GIPC2; MGC27382; PTGFR; C1orf29 |
| 2730 | GCLM | 1 | 94064610 | 1p22.1 | gneg 13; | 1.95 | 0.0397891 | 0.325 | 0.371 | 0.18 | -0.68 | GFI1; EVI5; RPL5; M96; CGI-100; LOC90673; DR1; FLJ20275; BCAR3; HSU15552; GCLM; ABCA4; PARG1; ABCD3; F3; 400763; MGC45474; CNN3; MGC19780; FLJ31842; RWDD3 |
| 5825 | ABCD3 | 1 | 94596031 | 1p22-p21 | gneg 12; gpos 1; | 1.69 | 0.0356581 | 0.372 | 0.378 | 0.226 | -0.693 | M96; CGI-100; LOC90673; DR1; FLJ20275; BCAR3; HSU15552; GCLM; ABCA4; PARG1; ABCD3; F3; 400763; MGC45474; CNN3; MGC19780; FLJ31842; RWDD3; PTBP2; DPYD; SNX7 |
| 1901 | EDG1 | 1 | 101414596 | 1p21 | gneg 4; gpos 3; | -7.61 | 0.0019277 | 0.314 | -0.514 | 0.047 | 0.942 | FLJ10287; MGC14816; DBT; RTCD1; CDC14A; GPR88; VCAM1; EXTL2; SLC30A7; CGI-30; EDG1; 391062; COL11A1; FLJ25070; AMY2B; AMY2A; AMY1A; 401500; 389028; 126987; PRMT6 |
| 55599 | FLJ25070 | 1 | 103780619 | 1p21 | gneg 7; gpos 2; | 1.43 | 0.198791 | 0.308 | 0.242 | 0.061 | -0.444 | RTCD1; CDC14A; GPR88; VCAM1; EXTL2; SLC30A7; CGI-30; EDG1; 391062; COL11A1; FLJ25070; AMY2B; AMY2A; AMY1A; 401500; 389028; 126987; PRMT6; NTNG1; VAV3; DKFZp586G0123 |
| 515 | ATP5F1 | 1 | 111703807 | 1p13.2 | gneg 4; gpos 10; | 1.39 | 0.0198038 | 0.301 | 0.412 | -0.077 | -0.756 | RIF1; MGC54289; CEPT1; FLJ22457; CHI3L2; 149620; CHIA; LOC128344; OVGP1; MEP50; ATP5F1; MGC24133; AD026; ADORA3; RAP1A; LOC55924; DDX20; KCND3; DKFZp547A023; TUBB2; WNT2B |
| 64216 | TFB2M | 1 | 243029908 | 1q44 | gneg 19; gpos 1; | 2.16 | 0.0069883 | 0.303 | 0.464 | 0.009 | -0.85 | ADSS; MGC33370; PNAS-4; LOC116228; HNRPU; KIAA1674; MGC12458; MGC35030; SMYD3; KIAA1170; TFB2M; FLJ32001; KIAA1765; CGI-49; ELYS; SBZF3; LOC90333; MGC12466; FLJ12606; ZNF124; VN1R5 |
| 51454 | GULP1 | 2 | 188983087 | 2q32.3-q33 | gneg 5; gpos 6; | 2.29 | 0.0004496 | 0.324 | 0.558 | 0.263 | -1.023 | LOC129401; LOC91752; FLJ34780; 401025; LEREPO4; ITGAV; KIAA1946; ZSWIM2; CALCRL; TFPI; GULP1; DIRC1; COL3A1; COL5A2; FLJ12519; SLC40A1; NS3TP1; NUP160; OSGEPL1; ORMDL1; PMS1 |
| 3336 | HSPE1 | 2 | 198190625 | 2q33.1 | gneg 17; gpos 1; | 2.27 | 1.08E-09 | 0.321 | 0.709 | 0.073 | -1.3 | NEDL2; FLJ39660; 200624; DKFZp434P055; GTF3C3; FLJ12377; LOC91526; SF3B1; FLJ13448; HSPD1; HSPE1; PREI3; C2orf11; LOC92935; BOLL; PLCL1; SATB2; FLJ32063; TTC7L1; KCNT1; FLJ38973 |
| 93622 | LOC93622 | 4 | 6793794 | 4p16.1 | gneg 9; gpos 4; | 1.28 | 0.7318704 | 0.333 | 0.066 | -0.096 | -0.122 | C4orf6; EVC2; EVC; CRMP1; FLJ31564; LOC285484; WFS1; PPP2R2C; KIAA0935; PGR1; LOC93622; S100P; MGC9651; CNO; KIAA1322; FLJ90575; MGC21874; GRPEL1; 401119; SORCS2; AFAP |
| 27068 | PPA2 | 4 | 106648323 | 4q25 | gneg 9; gpos 6; | 1.14 | 0.5456471 | 0.323 | -0.116 | 0.124 | 0.213 | UBE2D3; LOC150159; LOC133308; DHRS6; CENPE; TACR3; KIAA0877; IDAX; 390732; FLJ20032; PPA2; FLJ20184; 401147; LOC57117; FLJ13273; LOC255743; MGC16169; SCYE1; DKK2; PAPSS1; MGC26963 |
| 57117 | LOC57117 | 4 | 106961388 | 4q25 | gneg 10; gpos 3; | 1.11 | 0.8367958 | 0.467 | -0.04 | 0.067 | 0.073 | DHRS6; CENPE; TACR3; KIAA0877; IDAX; 390732; FLJ20032; PPA2; FLJ20184; 401147; LOC57117; FLJ13273; LOC255743; MGC16169; SCYE1; DKK2; PAPSS1; MGC26963; LOC113612; HADHSC; LEF1 |
| 93627 | MGC16169 | 4 | 107324843 | 4q25 | gneg 12; gpos 2; | -1.08 | 0.0865092 | 0.433 | -0.316 | 0.134 | 0.58 | KIAA0877; IDAX; 390732; FLJ20032; PPA2; FLJ20184; 401147; LOC57117; FLJ13273; LOC255743; MGC16169; SCYE1; DKK2; PAPSS1; MGC26963; LOC113612; HADHSC; LEF1; FLJ37673; RPL34; DC2 |
| 9255 | SCYE1 | 4 | 107595278 | 4q25 | gneg 13; gpos 2; | 1.83 | 0.0014147 | 0.321 | 0.524 | -0.023 | -0.961 | IDAX; 390732; FLJ20032; PPA2; FLJ20184; 401147; LOC57117; FLJ13273; LOC255743; MGC16169; SCYE1; DKK2; PAPSS1; MGC26963; LOC113612; HADHSC; LEF1; FLJ37673; RPL34; DC2; AGXT2L1 |
| 27123 | DKK2 | 4 | 108200564 | 4q25 | gneg 14; gpos 2; | -1.28 | 0.1313486 | 0.304 | -0.281 | 0.156 | 0.516 | 390732; FLJ20032; PPA2; FLJ20184; 401147; LOC57117; FLJ13273; LOC255743; MGC16169; SCYE1; DKK2; PAPSS1; MGC26963; LOC113612; HADHSC; LEF1; FLJ37673; RPL34; DC2; AGXT2L1; COL25A1 |
| 166929 | MGC26963 | 4 | 109172033 | 4q25 | gneg 16; gpos 1; | 1.1 | 0.2517339 | 0.322 | -0.217 | -0.194 | 0.398 | PPA2; FLJ20184; 401147; LOC57117; FLJ13273; LOC255743; MGC16169; SCYE1; DKK2; PAPSS1; MGC26963; LOC113612; HADHSC; LEF1; FLJ37673; RPL34; DC2; AGXT2L1; COL25A1; SEC24B; FLJ20647 |
| 3673 | ITGA2 | 5 | 52321013 | 5q23-q31 | acen 1; gneg 10; gpos 3; | 1.38 | 0.9848573 | 0.312 | 0.004 | -0.13 | -0.007 | PAIP1; NNT; FGF10; MRPS30; HCN1; MGC71745; FLJ21308; ISL1; PELO; ITGA1; ITGA2; MOCS2; FST; NDUFS4; FLJ20051; OATL1; HSPB3; SNAG1; 153134; ESM1; GZMK |
| 4338 | MOCS2 | 5 | 52429653 | 5q11 | acen 1; gneg 10; gpos 2; | -1.07 | 0.0684621 | 0.358 | -0.334 | -0.036 | 0.612 | NNT; FGF10; MRPS30; HCN1; MGC71745; FLJ21308; ISL1; PELO; ITGA1; ITGA2; MOCS2; FST; NDUFS4; FLJ20051; OATL1; HSPB3; SNAG1; 153134; ESM1; GZMK; GZMA |
| 4724 | NDUFS4 | 5 | 52892241 | 5q11.1 | acen 1; gneg 12; gpos 2; | 1.42 | 0.2272665 | 0.303 | 0.228 | -0.12 | -0.419 | MRPS30; HCN1; MGC71745; FLJ21308; ISL1; PELO; ITGA1; ITGA2; MOCS2; FST; NDUFS4; FLJ20051; OATL1; HSPB3; SNAG1; 153134; ESM1; GZMK; GZMA; FLJ37927; 345643 |
| 51194 | IPO11 | 5 | 61744350 | 5q12.2 | gneg 1; gpos 11; | 2.08 | 0.0002981 | 0.304 | 0.569 | 0.108 | -1.043 | RAB3C; PDE4D; 390468; PART1; XTP1; CKN1; LOC91942; FLJ37543; KIF2; HSA9761; IPO11; SLRN; HTR1A; LOC285671; FLJ36754; SDCCAG10; LOC345667; ADAMTS6; FKSG14; KIAA0073; ARFD1 |
| 55914 | ERBB2IP | 5 | 65258139 | 5q13.1 | gneg 4; gpos 12; | 1.01 | 0.6685543 | 0.315 | -0.083 | -0.015 | 0.152 | FLJ36754; SDCCAG10; LOC345667; ADAMTS6; FKSG14; KIAA0073; ARFD1; FLJ13611; SGTB; NLN; ERBB2IP; SFRS12; LOC375449; LY64; 391282; PIK3R1; SLC30A5; CCNB1; CENPH; MRPS36; CDK7 |
| 6880 | TAF9 | 5 | 68683310 | 5q11.2-q13.1 | gneg 6; gpos 3; | 2.02 | 0.0263509 | 0.303 | 0.396 | -0.073 | -0.727 | LY64; 391282; PIK3R1; SLC30A5; CCNB1; CENPH; MRPS36; CDK7; KENAE; SMA3; TAF9; RAD17; FLJ30532; OCLN; GTF2H2; 401486; SERF1B; SMN2; SMN1; BIRC1; PMCHL2 |
| 22936 | ELL2 | 5 | 95248856 | 5q15 | gneg 14; gpos 1; | 2.67 | 0.038321 | 0.318 | 0.374 | -0.025 | -0.685 | 389310; FLJ25333; KIAA0372; DKFZp313G1735; LOC285601; LOC317671; SPATA9; RHOBTB3; GLRX; FIS; ELL2; PCSK1; CAST; ARTS-1; LRAP; FLJ39485; LNPEP; FLJ25534; RIOK2; FLJ90406; CHD1 |
| 167410 | FLJ25534 | 5 | 96453329 | 5q15 | gneg 12; gpos 2; | -5.88 | 0.0035328 | 0.3 | -0.492 | -0.079 | 0.901 | RHOBTB3; GLRX; FIS; ELL2; PCSK1; CAST; ARTS-1; LRAP; FLJ39485; LNPEP; FLJ25534; RIOK2; FLJ90406; CHD1; UNQ1912; SIAT8D; SLCO4C1; SLCO6A1; PAM; FLJ20125; KIAA0433 |
| 153222 | LOC153222 | 5 | 172415975 | 5q35.2 | gneg 4; gpos 12; | -1.04 | 0.4366144 | 0.319 | -0.149 | -0.124 | 0.274 | FGF18; FBXW1B; STK10; 285588; DC-UbP; 401217; DUSP1; KIAA1181; RPL26L1; ATP6V0E; LOC153222; BNIP1; NKX2-5; STC2; LOC91272; CPEB4; HMP19; MSX2; DRD1; SFXN1; HRH2 |
| 662 | BNIP1 | 5 | 172504145 | 5q33-q34 | gneg 5; gpos 11; | 1.3 | 0.7253302 | 0.304 | -0.068 | 0.033 | 0.125 | FBXW1B; STK10; 285588; DC-UbP; 401217; DUSP1; KIAA1181; RPL26L1; ATP6V0E; LOC153222; BNIP1; NKX2-5; STC2; LOC91272; CPEB4; HMP19; MSX2; DRD1; SFXN1; HRH2; CPLX2 |
| 1482 | NKX2-5 | 5 | 172591743 | 5q34 | gneg 5; gpos 11; | 1.07 | 0.4006833 | 0.336 | -0.161 | 0.122 | 0.295 | STK10; 285588; DC-UbP; 401217; DUSP1; KIAA1181; RPL26L1; ATP6V0E; LOC153222; BNIP1; NKX2-5; STC2; LOC91272; CPEB4; HMP19; MSX2; DRD1; SFXN1; HRH2; CPLX2; DKFZp547D155 |
| 222826 | C6orf146 | 6 | 4013600 | 6p25.1 | gneg 9; gpos 3; | 1.38 | 0.4387914 | 0.306 | -0.149 | 0.219 | 0.272 | SERPINB6; NQO2; RIPK1; BPHL; TUBB; MGC8685; C6orf85; C6orf145; D6S2654E; PRPF4B; C6orf146; 404220; PECI; 285777; CDYL; RNASEP1; 389364; C6orf149; FARS1; NRN1; F13A1 |
| 2651 | GCNT2 | 6 | 10693980 | 6p24 | gneg 1; gpos 11; | 3.67 | 0.1699582 | 0.361 | 0.258 | 0.138 | -0.472 | MGC26597; 63915; EEF1E1; SLC35B3; OFCC1; PAK1IP1; KIAA1623; TFAP2A; MGC40222; 401234; GCNT2; C6orf53; TMEM14B; MAK; GCM2; LOC221711; ELOVL2; NEDD9; C6orf105; HIVEP1; EDN1 |
| 51522 | C6orf53 | 6 | 10831323 | 6p24.1 | gneg 2; gpos 10; | 1.23 | 0.6414732 | 0.361 | 0.09 | 0.131 | -0.165 | 63915; EEF1E1; SLC35B3; OFCC1; PAK1IP1; KIAA1623; TFAP2A; MGC40222; 401234; GCNT2; C6orf53; TMEM14B; MAK; GCM2; LOC221711; ELOVL2; NEDD9; C6orf105; HIVEP1; EDN1; TBC1D7 |
| 81853 | TMEM14B | 6 | 10856035 | 6p25.1-p23 | gneg 2; gpos 10; | 1.32 | 0.4078894 | 0.326 | 0.159 | 0.037 | -0.291 | EEF1E1; SLC35B3; OFCC1; PAK1IP1; KIAA1623; TFAP2A; MGC40222; 401234; GCNT2; C6orf53; TMEM14B; MAK; GCM2; LOC221711; ELOVL2; NEDD9; C6orf105; HIVEP1; EDN1; TBC1D7; GFOD1 |
| 4739 | NEDD9 | 6 | 11291519 | 6p25-p24 | gneg 6; gpos 6; | 1.62 | 0.8688141 | 0.398 | 0.032 | 0.079 | -0.059 | TFAP2A; MGC40222; 401234; GCNT2; C6orf53; TMEM14B; MAK; GCM2; LOC221711; ELOVL2; NEDD9; C6orf105; HIVEP1; EDN1; TBC1D7; GFOD1; C6orf114; SIRT5; RARG-1; RANBP9; C6orf79 |
| 3096 | HIVEP1 | 6 | 12120556 | 6p24-p22.3 | gneg 8; gpos 5; | 1.27 | 0.5901646 | 0.382 | 0.104 | 0.049 | -0.19 | 401234; GCNT2; C6orf53; TMEM14B; MAK; GCM2; LOC221711; ELOVL2; NEDD9; C6orf105; HIVEP1; EDN1; TBC1D7; GFOD1; C6orf114; SIRT5; RARG-1; RANBP9; C6orf79; MGC33993; CD83 |
| 8364 | HIST1H4C | 6 | 26212154 | 6p21.3 | gneg 1; | 1.13 | 0.1773319 | 0.377 | 0.254 | 0.416 | -0.465 | HIST1H1A; HIST1H3A; HIST1H4A; HIST1H4B; HIST1H3B; HIST1H2AB; HIST1H2BB; HIST1H3C; HIST1H1C; HFE; HIST1H4C; HIST1H1T; HIST1H2BC; HIST1H2AC; LOC286144; HIST1H1E; HIST1H2BD; HIST1H2BE; HIST1H4D; HIST1H3D; HIST1H2AD |
| 8344 | HIST1H2BE | 6 | 26292002 | 6p21.3 | gneg 1; | -1.19 | 0.0927229 | 0.357 | -0.311 | 0.423 | 0.57 | HIST1H3C; HIST1H1C; HFE; HIST1H4C; HIST1H1T; HIST1H2BC; HIST1H2AC; LOC286144; HIST1H1E; HIST1H2BD; HIST1H2BE; HIST1H4D; HIST1H3D; HIST1H2AD; HIST1H2BF; HIST1H4E; HIST1H2BG; HIST1H2AE; HIST1H3E; HIST1H1D; HIST1H4F |
| 8360 | HIST1H4D | 6 | 26296916 | 6p21.3 | gneg 1; | 1.19 | 0.570058 | 0.411 | -0.11 | 0.475 | 0.201 | HIST1H1C; HFE; HIST1H4C; HIST1H1T; HIST1H2BC; HIST1H2AC; LOC286144; HIST1H1E; HIST1H2BD; HIST1H2BE; HIST1H4D; HIST1H3D; HIST1H2AD; HIST1H2BF; HIST1H4E; HIST1H2BG; HIST1H2AE; HIST1H3E; HIST1H1D; HIST1H4F; HIST1H4G |
| 8353 | HIST1H3E | 6 | 26333361 | 6p21.3 | gpos 3; | 1.41 | 0.7666822 | 0.335 | -0.057 | 0.389 | 0.105 | HIST1H1E; HIST1H2BD; HIST1H2BE; HIST1H4D; HIST1H3D; HIST1H2AD; HIST1H2BF; HIST1H4E; HIST1H2BG; HIST1H2AE; HIST1H3E; HIST1H1D; HIST1H4F; HIST1H4G; HIST1H3F; HIST1H2BH; HIST1H3G; HIST1H2BI; HIST1H4H; BTN3A2; BTN2A2 |
| 3127 | HLA-DRB5 | 6 | 32593133 | 6p21.3 | gneg 1; | -1.78 | 0.3854107 | 0.305 | -0.166 | 0.143 | 0.305 | EGFL8; AGPAT1; RNF5; AGER; PBX2; C6orf9; NOTCH4; C6orf10; BTNL2; HLA-DRA; HLA-DRB5; HLA-DRB1; HLA-DRB3; HLA-DQA1; HLA-DQA2; HLA-DQB2; HLA-DOB; TAP2; PSMB8; TAP1; PSMB9 |
| 6890 | TAP1 | 6 | 32920964 | 6p21.3 |  | 3.05 | 0.0285155 | 0.313 | 0.392 | 0.12 | -0.718 | HLA-DRA; HLA-DRB5; HLA-DRB1; HLA-DRB3; HLA-DQA1; HLA-DQA2; HLA-DQB2; HLA-DOB; TAP2; PSMB8; TAP1; PSMB9; HLA-DMB; HLA-DMA; BRD2; HLA-DOA; HLA-DPA1; HLA-DPB1; COL11A2; RXRB; SLC39A7 |
| 60 | ACTB | 7 | 5340026 | 7p15-p12 | gpos 11; | -1 | 0.7174731 | 0.319 | 0.07 | 0.022 | -0.128 | CARD11; SDK1; FLJ10324; PAPOLB; PAQR10; RBAK; 389458; DKFZP434J154; SLC29A4; FLJ11467; ACTB; FSCN1; TRIAD3; 402455; LOC222967; PMS2; JTV1; HRI; PSCD3; MGC12966; RAC1 |
| 57037 | DKFZP564O043 | 7 | 16412651 | 7p21 | gneg 5; gpos 9; | -1.4 | 0.0689617 | 0.313 | -0.333 | -0.37 | 0.611 | KIAA0960; FLJ11273; FLJ14712; SCIN; ARL4; ETV1; DGKB; 392636; MEOX2; SOSTDC1; DKFZP564O043; BZW2; TM4SF13; AGR2; BCMP11; AHR; SNX13; PRPS1L1; 402463; HDAC9; TWIST1 |
| 222171 | LOC222171 | 7 | 29376666 | 7p15.1 | gneg 8; gpos 1; | 1.35 | 0.8896065 | 0.325 | -0.027 | 0.009 | 0.049 | HOXA11; HOXA13; EVX1; HIBADH; TAX1BP1; JAZF1; CREB5; CPVL; C14orf120; CHN2; LOC222171; SES1; FKBP14; FAPP2; Ells1; LOC223082; DKFZp586I1420; CARD4; C7orf24; GARS; CRHR2 |
| 4897 | NRCAM | 7 | 107382057 | 7q31.1-q31.2 | gneg 3; gpos 4; | 1.36 | 0.6087085 | 0.331 | 0.099 | -0.089 | -0.181 | HBP1; COG5; GPR22; PP35; BCAP29; SLC26A4; CBLL1; SLC26A3; DLD; LAMB1; NRCAM; IPLA2(GAMMA); THAP5; DNAJB9; 154907; IMMP2L; LRRN3; DOCK4; ZNF277; IFRD1; FLJ39575 |
| 286006 | FLJ39575 | 7 | 111714996 | 7q31.1 | gneg 6; gpos 7; | -1.16 | 0.6778311 | 0.333 | -0.08 | -0.118 | 0.147 | NRCAM; IPLA2(GAMMA); THAP5; DNAJB9; 154907; IMMP2L; LRRN3; DOCK4; ZNF277; IFRD1; FLJ39575; FLJ13576; FLJ31818; GPR85; 402587; PPP1R3A; FOXP2; TFEC; TES; CAV2; CAV1 |
| 6386 | SDCBP | 8 | 59628289 | 8q12 | gneg 5; gpos 6; | -1.09 | 0.5836131 | 0.317 | -0.106 | -0.029 | 0.194 | LYN; RPS20; MOS; PLAG1; MGC2217; RDH-E2; PENK; FLJ20421; MGC39325; CYP7A1; SDCBP; 401584; NSMAF; TOX; MGC9913; CA8; RAB2; 157813; MGC34646; ASPH; FLJ39630 |
| 386759 | MGC9913 | 8 | 61044926 | 19q13.43 | gneg 8; gpos 6; | 1.32 | 0.4684385 | 0.315 | -0.139 | -0.04 | 0.255 | MGC2217; RDH-E2; PENK; FLJ20421; MGC39325; CYP7A1; SDCBP; 401584; NSMAF; TOX; MGC9913; CA8; RAB2; 157813; MGC34646; ASPH; FLJ39630; GGH; TTPA; FLJ31657; SPN |
| 83690 | LOC83690 | 8 | 76059530 | 8q13.3 | gneg 9; gpos 3; | 1.29 | 0.1358409 | 0.334 | 0.278 | -0.08 | -0.51 | RDH10; STAU2; FLJ11011; TCEB1; FLJ20533; LY96; JPH1; GDAP1; 286157; PI15; LOC83690; HNF4G; ZFH4; PXMP3; PKIA; CGI-62; IL7; STMN2; HEY1; MRPS28; TPD52 |
| 3174 | HNF4G | 8 | 76614757 | 8q13.3-q21.11 | gneg 8; gpos 3; | 1.77 | 0.1842818 | 0.326 | 0.25 | 0.131 | -0.458 | STAU2; FLJ11011; TCEB1; FLJ20533; LY96; JPH1; GDAP1; 286157; PI15; LOC83690; HNF4G; ZFH4; PXMP3; PKIA; CGI-62; IL7; STMN2; HEY1; MRPS28; TPD52; RINZF |
| 5828 | PXMP3 | 8 | 78057712 | 8q21.1 | gneg 8; gpos 4; | -1.11 | 0.1384162 | 0.331 | -0.277 | -0.148 | 0.507 | TCEB1; FLJ20533; LY96; JPH1; GDAP1; 286157; PI15; LOC83690; HNF4G; ZFH4; PXMP3; PKIA; CGI-62; IL7; STMN2; HEY1; MRPS28; TPD52; RINZF; PAG; FABP5 |
| 51101 | CGI-62 | 8 | 79740884 | 8q21.11 | gneg 6; gpos 4; | -1.58 | 0.0234417 | 0.457 | -0.403 | 0.11 | 0.739 | LY96; JPH1; GDAP1; 286157; PI15; LOC83690; HNF4G; ZFH4; PXMP3; PKIA; CGI-62; IL7; STMN2; HEY1; MRPS28; TPD52; RINZF; PAG; FABP5; PMP2; FABP4 |
| 11075 | STMN2 | 8 | 80685934 | 8q21.11-q21.12 | gneg 5; gpos 5; | -5.22 | 6.15E-05 | 0.46 | -0.604 | 0.216 | 1.107 | GDAP1; 286157; PI15; LOC83690; HNF4G; ZFH4; PXMP3; PKIA; CGI-62; IL7; STMN2; HEY1; MRPS28; TPD52; RINZF; PAG; FABP5; PMP2; FABP4; IMPA1; FLJ14007 |
| 23462 | HEY1 | 8 | 80838800 | 8q21 | gneg 4; gpos 6; | -5.24 | 9.28E-07 | 0.403 | -0.664 | 0.226 | 1.217 | 286157; PI15; LOC83690; HNF4G; ZFH4; PXMP3; PKIA; CGI-62; IL7; STMN2; HEY1; MRPS28; TPD52; RINZF; PAG; FABP5; PMP2; FABP4; IMPA1; FLJ14007; Shax3 |
| 28957 | MRPS28 | 8 | 80993650 | 8q21.1-q21.2 | gneg 3; gpos 7; | 1.68 | 0.0022265 | 0.431 | 0.509 | 0.222 | -0.933 | PI15; LOC83690; HNF4G; ZFH4; PXMP3; PKIA; CGI-62; IL7; STMN2; HEY1; MRPS28; TPD52; RINZF; PAG; FABP5; PMP2; FABP4; IMPA1; FLJ14007; Shax3; SNX16 |
| 55824 | PAG | 8 | 82048962 | 8q21.12 | gneg 3; gpos 8; | -7.24 | 0.0001697 | 0.383 | -0.582 | -0.018 | 1.068 | ZFH4; PXMP3; PKIA; CGI-62; IL7; STMN2; HEY1; MRPS28; TPD52; RINZF; PAG; FABP5; PMP2; FABP4; IMPA1; FLJ14007; Shax3; SNX16; LOC138046; E2F5; MGC59868 |
| 2171 | FABP5 | 8 | 82355339 | 8q21.13 | gneg 3; gpos 7; | 1.42 | 0.0782446 | 0.313 | 0.324 | -0.026 | -0.594 | PXMP3; PKIA; CGI-62; IL7; STMN2; HEY1; MRPS28; TPD52; RINZF; PAG; FABP5; PMP2; FABP4; IMPA1; FLJ14007; Shax3; SNX16; LOC138046; E2F5; MGC59868; CA1 |
| 2167 | FABP4 | 8 | 82553480 | 8q21 | gneg 3; gpos 6; | -2.04 | 0.1702502 | 0.421 | -0.257 | -0.071 | 0.472 | CGI-62; IL7; STMN2; HEY1; MRPS28; TPD52; RINZF; PAG; FABP5; PMP2; FABP4; IMPA1; FLJ14007; Shax3; SNX16; LOC138046; E2F5; MGC59868; CA1; CA3; CA2 |
| 760 | CA2 | 8 | 86563497 | 8q22 | gneg 7; gpos 5; | -1.29 | 0.9022202 | 0.307 | -0.024 | 0.01 | 0.044 | FABP4; IMPA1; FLJ14007; Shax3; SNX16; LOC138046; E2F5; MGC59868; CA1; CA3; CA2; GOR; PSKH2; ATP6V0D2; SLC7A13; WWP1; CGI-90; CPNE3; CNGB3; FLJ35802; FLJ35775 |
| 51115 | CGI-90 | 8 | 87555453 | 8q21.2 | gneg 7; gpos 2; | 1.25 | 0.8529689 | 0.301 | 0.036 | -0.089 | -0.066 | E2F5; MGC59868; CA1; CA3; CA2; GOR; PSKH2; ATP6V0D2; SLC7A13; WWP1; CGI-90; CPNE3; CNGB3; FLJ35802; FLJ35775; MMP16; RIPK2; C8orf1; NBS1; DECR1; CALB1 |
| 4982 | TNFRSF11B | 8 | 120004977 | 8q24 | gneg 4; gpos 6; | 3.13 | 0.6430315 | 0.32 | 0.09 | -0.013 | -0.164 | KCNV1; 392262; CSMD3; TRPS1; EIF3S3; MGC14595; RAD21; SLC30A8; TRAP25; EXT1; TNFRSF11B; COLEC10; MAL2; NOV; ENPP2; TAF2; MGC5528; FLJ12428; COL14A1; MRPL13; MTBP |
| 79943 | FLJ14129 | 8 | 144444970 | 8q24.3 | gneg 16; | 1.26 | 0.1984796 | 0.305 | 0.242 | 0.12 | -0.444 | E48; GML; CYP11B1; CYP11B2; LY6E; FLJ37131; LY6H; LOC338328; FLJ38705; GLI4; FLJ14129; TOP1MT; MGC3113; RHPN1; 389692; KIAA0150; FLJ12150; PP3856; EEF1D; TIGD5; FLJ13852 |
| 116447 | TOP1MT | 8 | 144462904 | 8q24.3 | gneg 17; | 2.51 | 0.0083167 | 0.329 | 0.456 | -0.08 | -0.836 | GML; CYP11B1; CYP11B2; LY6E; FLJ37131; LY6H; LOC338328; FLJ38705; GLI4; FLJ14129; TOP1MT; MGC3113; RHPN1; 389692; KIAA0150; FLJ12150; PP3856; EEF1D; TIGD5; FLJ13852; TSTA3 |
| 79792 | FLJ12150 | 8 | 144712102 | 8q24.3 | gneg 18; | 1.42 | 0.5337397 | 0.32 | 0.12 | -0.045 | -0.22 | LY6H; LOC338328; FLJ38705; GLI4; FLJ14129; TOP1MT; MGC3113; RHPN1; 389692; KIAA0150; FLJ12150; PP3856; EEF1D; TIGD5; FLJ13852; TSTA3; KIAA0628; LOC286075; 286076; ERK8; FLJ46072 |
| 84948 | TIGD5 | 8 | 144751363 | 8q24.3 | gneg 17; | 1.09 | 0.7028917 | 0.348 | 0.074 | 0.074 | -0.135 | GLI4; FLJ14129; TOP1MT; MGC3113; RHPN1; 389692; KIAA0150; FLJ12150; PP3856; EEF1D; TIGD5; FLJ13852; TSTA3; KIAA0628; LOC286075; 286076; ERK8; FLJ46072; SCRIB; SIAHBP1; LOC340371 |
| 286077 | FLJ46072 | 8 | 144878091 | 8q24.3 | gneg 17; | 1.34 | 0.1549179 | 0.339 | 0.266 | -0.019 | -0.488 | FLJ12150; PP3856; EEF1D; TIGD5; FLJ13852; TSTA3; KIAA0628; LOC286075; 286076; ERK8; FLJ46072; SCRIB; SIAHBP1; LOC340371; 389697; PLEC1; FLJ14464; MGC61633; OPLAH; RRP41; GPAA1 |
| 26873 | OPLAH | 8 | 145178164 | 8q24.3 | gneg 17; | 1.15 | 0.5317943 | 0.318 | 0.12 | -0.159 | -0.221 | 286076; ERK8; FLJ46072; SCRIB; SIAHBP1; LOC340371; 389697; PLEC1; FLJ14464; MGC61633; OPLAH; RRP41; GPAA1; CYC1; DKFZP434N1923; MAF1; KIAA1875; LOC51236; BOP1; HSF1; DGAT1 |
| 8733 | GPAA1 | 8 | 145209526 | 8q24.3 | gneg 18; | -1.1 | 0.0432993 | 0.322 | -0.366 | -0.028 | 0.67 | FLJ46072; SCRIB; SIAHBP1; LOC340371; 389697; PLEC1; FLJ14464; MGC61633; OPLAH; RRP41; GPAA1; CYC1; DKFZP434N1923; MAF1; KIAA1875; LOC51236; BOP1; HSF1; DGAT1; DKFZp547F072; SCRT1 |
| 51236 | LOC51236 | 8 | 145264659 | 8q24.3 | gneg 19; gpos 1; | 1.81 | 0.0128645 | 0.381 | 0.435 | -0.147 | -0.797 | PLEC1; FLJ14464; MGC61633; OPLAH; RRP41; GPAA1; CYC1; DKFZP434N1923; MAF1; KIAA1875; LOC51236; BOP1; HSF1; DGAT1; DKFZp547F072; SCRT1; 340393; FBXL6; FLJ11856; ADCK5; CPSF1 |
| 29894 | CPSF1 | 8 | 145589257 | 8q24.23 | gneg 19; gpos 1; | -1.06 | 0.2235037 | 0.353 | -0.23 | -0.164 | 0.422 | LOC51236; BOP1; HSF1; DGAT1; DKFZp547F072; SCRT1; 340393; FBXL6; FLJ11856; ADCK5; CPSF1; SLC39A4; VPS28; NFKBIL2; CYHR1; KIFC2; FOXH1; PPP1R16A; GPT; LOC113655; RECQL4 |
| 4796 | NFKBIL2 | 8 | 145624998 | 8q24.3 | gneg 19; gpos 1; | 1.27 | 0.5373284 | 0.387 | 0.119 | 0.161 | -0.218 | DGAT1; DKFZp547F072; SCRT1; 340393; FBXL6; FLJ11856; ADCK5; CPSF1; SLC39A4; VPS28; NFKBIL2; CYHR1; KIFC2; FOXH1; PPP1R16A; GPT; LOC113655; RECQL4; LRRC14; ZNF34; RPL8 |
| 50626 | CYHR1 | 8 | 145660015 | 8 | gneg 18; gpos 1; | -1.07 | 0.0305333 | 0.349 | -0.388 | -0.31 | 0.711 | DKFZp547F072; SCRT1; 340393; FBXL6; FLJ11856; ADCK5; CPSF1; SLC39A4; VPS28; NFKBIL2; CYHR1; KIFC2; FOXH1; PPP1R16A; GPT; LOC113655; RECQL4; LRRC14; ZNF34; RPL8; ZNF7 |
| 90990 | KIFC2 | 8 | 145662545 | 8q24.3 | gneg 17; gpos 1; | 1.35 | 0.6240449 | 0.415 | 0.095 | 0.256 | -0.174 | SCRT1; 340393; FBXL6; FLJ11856; ADCK5; CPSF1; SLC39A4; VPS28; NFKBIL2; CYHR1; KIFC2; FOXH1; PPP1R16A; GPT; LOC113655; RECQL4; LRRC14; ZNF34; RPL8; ZNF7; HT002 |
| 10444 | C9orf60 | 9 | 128571619 | 9q34.13 | gneg 14; | -1.41 | 0.0005175 | 0.329 | -0.554 | -0.001 | 1.016 | TMSL4; C9orf74; CEECAM1; ODF2; GLE1L; SPTAN1; MGC20486; SET; pknbeta; ZDHHC12; C9orf60; TBC1D13; ENDOG; HSPC109; CCBL1; LRRC8; TMEM15; SH3GLB2; C9orf54; DOLPP1; CRAT |
| 57109 | XPMC2H | 9 | 133300741 | 9q34.3 | gneg 4; gpos 5; | 1.54 | 0.207252 | 0.327 | 0.238 | -0.002 | -0.436 | RALGDS; FS; OBP2B; ABO; SURF6; SURF5; RPL7A; SURF1; SURF2; SURF4; XPMC2H; ADAMTS13; C9orf7; SLC2A6; KIAA0605; DBH; SARDH; VAV2; BRD3; WDR5; RXRA |
| 1645 | AKR1C1 | 10 | 4995617 | 10p15-p14 | gneg 8; gpos 1; | 1.31 | 0.8492905 | 0.316 | -0.037 | -0.085 | 0.068 | IDI1; KIAA0982; 399706; ADARB2; 399708; 387630; PFKP; PITRM1; COPEB; AKR1CL2; AKR1C1; AKR1C2; AKR1C3; AKR1C4; UCN3; FLJ21665; NET1; CALML5; CALML3; ASB13; GDI2 |
| 84647 | PLA2G12B | 10 | 74364943 | 10q22.3 | gneg 8; gpos 5; | -4.27 | 0.0058637 | 0.303 | -0.471 | -0.001 | 0.864 | CDH23; PSAP; CHST3; CGI-18; LOC119504; RTP801; DNAJB12; CBARA1; C10orf42; FLJ39116; PLA2G12B; P4HA1; NUDT13; HSGT1; DNAJC9; 128710; MRPS16; LOC118491; ANXA7; ZMYND17; PPP3CB |
| 119559 | SFXN4 | 10 | 120890431 | 10q26.13 | gneg 8; gpos 4; | 1.18 | 0.8082657 | 0.3 | -0.047 | -0.066 | 0.086 | LOC118987; EMX2; Rab11-FIP2; C10orf5; FLJ13188; GPR10; C10orf46; NANOS1; EIF3S10; HT011; SFXN4; PRDX3; GPRK5; RGS10; TIAL1; BAG3; INPP5F; FLJ13081; SEC23IP; LOC196051; WDR11 |
| 10410 | IFITM3 | 11 | 309668 | 11p15.5 | gneg 18; | 1.01 | 0.3018034 | 0.414 | -0.197 | 0.073 | 0.36 | RYD5; ODF3; BET1L; RIC-8; SIRT3; PSMD13; NALP6; FLJ22635; IFITM2; IFITM1; IFITM3; FLJ25045; PKP3; SIGIRR; PTDSS2; RNH; HRAS; DKFZp761L1518; MGC35138; C11orf13; IRF7 |
| 338707 | FLJ25045 | 11 | 359803 | 11p15.5 | gneg 18; | 1.24 | 0.3110737 | 0.323 | 0.193 | 0.014 | -0.354 | ODF3; BET1L; RIC-8; SIRT3; PSMD13; NALP6; FLJ22635; IFITM2; IFITM1; IFITM3; FLJ25045; PKP3; SIGIRR; PTDSS2; RNH; HRAS; DKFZp761L1518; MGC35138; C11orf13; IRF7; MUCDHL |
| 8045 | C11orf13 | 11 | 551449 | 11p15.5 | gneg 18; | 1.14 | 0.9041576 | 0.391 | 0.023 | -0.013 | -0.043 | IFITM1; IFITM3; FLJ25045; PKP3; SIGIRR; PTDSS2; RNH; HRAS; DKFZp761L1518; MGC35138; C11orf13; IRF7; MUCDHL; SCT; DRD4; DEAF1; LOC283232; EPS8L2; TALDO1; FLJ34283; BM88 |
| 1815 | DRD4 | 11 | 627304 | 11p15.5 | gneg 18; | -1.68 | 0.22136 | 0.329 | -0.231 | -0.193 | 0.424 | SIGIRR; PTDSS2; RNH; HRAS; DKFZp761L1518; MGC35138; C11orf13; IRF7; MUCDHL; SCT; DRD4; DEAF1; LOC283232; EPS8L2; TALDO1; FLJ34283; BM88; SLC25A22; LRDD; RPLP2; MGC45840 |
| 283232 | LOC283232 | 11 | 685642 | 11p15.5 | gneg 18; | 1.09 | 0.5357936 | 0.318 | 0.119 | -0.216 | -0.219 | RNH; HRAS; DKFZp761L1518; MGC35138; C11orf13; IRF7; MUCDHL; SCT; DRD4; DEAF1; LOC283232; EPS8L2; TALDO1; FLJ34283; BM88; SLC25A22; LRDD; RPLP2; MGC45840; CD151; POLR2L |
| 6888 | TALDO1 | 11 | 737431 | 11p15.5-p15.4 | gneg 18; | 1.51 | 0.0099946 | 0.373 | 0.447 | 0.083 | -0.82 | DKFZp761L1518; MGC35138; C11orf13; IRF7; MUCDHL; SCT; DRD4; DEAF1; LOC283232; EPS8L2; TALDO1; FLJ34283; BM88; SLC25A22; LRDD; RPLP2; MGC45840; CD151; POLR2L; TM4SF7; MGC3234 |
| 347862 | FLJ34283 | 11 | 757224 | 11p15.5 | gneg 18; | 1.09 | 0.527309 | 0.386 | -0.122 | -0.07 | 0.223 | MGC35138; C11orf13; IRF7; MUCDHL; SCT; DRD4; DEAF1; LOC283232; EPS8L2; TALDO1; FLJ34283; BM88; SLC25A22; LRDD; RPLP2; MGC45840; CD151; POLR2L; TM4SF7; MGC3234; AP2A2 |
| 51286 | BM88 | 11 | 777109 | 11p15.5 | gneg 18; | 4.97 | 0.1374831 | 0.412 | 0.277 | -0.079 | -0.508 | C11orf13; IRF7; MUCDHL; SCT; DRD4; DEAF1; LOC283232; EPS8L2; TALDO1; FLJ34283; BM88; SLC25A22; LRDD; RPLP2; MGC45840; CD151; POLR2L; TM4SF7; MGC3234; AP2A2; 338731 |
| 7106 | TM4SF7 | 11 | 832823 | 11p15.5 | gneg 17; | 1.06 | 0.1541835 | 0.318 | -0.267 | -0.006 | 0.489 | EPS8L2; TALDO1; FLJ34283; BM88; SLC25A22; LRDD; RPLP2; MGC45840; CD151; POLR2L; TM4SF7; MGC3234; AP2A2; 338731; MUC2; TOLLIP; STK29; HCCA2; DUSP8; CTSD; SYT8 |
| 9409 | PEX16 | 11 | 45888300 | 11p11.2 | gneg 15; | 1.28 | 0.7976853 | 0.302 | 0.05 | -0.027 | -0.091 | LOC90139; PRDM11; 219638; SYT13; CHST1; DKFZp779M0652; SLC35C1; CRY2; MAPK8IP1; 143678; PEX16; GYLTL1B; BHC80; CREB3L1; DGKZ; MDK; CHRM4; 387765; FLJ20294; FLJ32675; KIAA0652 |
| 120071 | GYLTL1B | 11 | 45899771 | 11p11.2 | gneg 14; | 8.41 | 0.0001796 | 0.322 | 0.581 | 0.017 | -1.065 | PRDM11; 219638; SYT13; CHST1; DKFZp779M0652; SLC35C1; CRY2; MAPK8IP1; 143678; PEX16; GYLTL1B; BHC80; CREB3L1; DGKZ; MDK; CHRM4; 387765; FLJ20294; FLJ32675; KIAA0652; ARHGAP1 |
| 84304 | MGC13045 | 11 | 63750337 | 11q13.1 | acen 1; gneg 8; | 1.24 | 0.7401628 | 0.331 | -0.064 | 0.374 | 0.118 | MARK2; LOC283248; FLJ13848; COX8; OTUB1; LRP16; FLRT1; STIP1; URP2; MGC11134; MGC13045; DNAJC4; VEGFB; FKBP2; PLCB3; BAD; C11ORF4; KCNK4; ESRRA; HSPC152; PRDX5 |
| 741 | C11orf5 | 11 | 64640451 | 11q13 | gneg 5; gpos 1; | 1.76 | 0.2056486 | 0.385 | 0.239 | 0.075 | -0.438 | ARL2; SNX15; HSU79266; NAALADL1; 256676; CDCA5; ZFPL1; 399904; C11orf2; TM7SF2; C11orf5; FAU; MRPL49; HRD1; CAPN1; POLA2; CDC42EP2; DPF2; TIGD3; LOC283130; FKSG44 |
| 84447 | HRD1 | 11 | 64651328 | 11q13 | gneg 5; gpos 1; | -1.11 | 0.6493922 | 0.385 | -0.088 | 0.043 | 0.161 | NAALADL1; 256676; CDCA5; ZFPL1; 399904; C11orf2; TM7SF2; C11orf5; FAU; MRPL49; HRD1; CAPN1; POLA2; CDC42EP2; DPF2; TIGD3; LOC283130; FKSG44; SCYL1; LTBP3; SSSCA1 |
| 823 | CAPN1 | 11 | 64705918 | 11q13 | gneg 5; gpos 1; | 1.51 | 0.2001129 | 0.396 | 0.242 | 0.063 | -0.443 | 256676; CDCA5; ZFPL1; 399904; C11orf2; TM7SF2; C11orf5; FAU; MRPL49; HRD1; CAPN1; POLA2; CDC42EP2; DPF2; TIGD3; LOC283130; FKSG44; SCYL1; LTBP3; SSSCA1; MTVR1 |
| 23649 | POLA2 | 11 | 64786007 | 11q13.1 | gneg 4; gpos 1; | 1.53 | 0.2312435 | 0.422 | 0.226 | -0.176 | -0.415 | CDCA5; ZFPL1; 399904; C11orf2; TM7SF2; C11orf5; FAU; MRPL49; HRD1; CAPN1; POLA2; CDC42EP2; DPF2; TIGD3; LOC283130; FKSG44; SCYL1; LTBP3; SSSCA1; MTVR1; KCNK7 |
| 10435 | CDC42EP2 | 11 | 64838906 | 11q13 | gneg 4; | -1.49 | 0.2186042 | 0.383 | -0.232 | 0.096 | 0.426 | ZFPL1; 399904; C11orf2; TM7SF2; C11orf5; FAU; MRPL49; HRD1; CAPN1; POLA2; CDC42EP2; DPF2; TIGD3; LOC283130; FKSG44; SCYL1; LTBP3; SSSCA1; MTVR1; KCNK7; MAP3K11 |
| 83786 | FKSG44 | 11 | 64910698 | 11q13 | gneg 5; | 1.2 | 0.8391701 | 0.393 | 0.039 | 0.054 | -0.072 | C11orf5; FAU; MRPL49; HRD1; CAPN1; POLA2; CDC42EP2; DPF2; TIGD3; LOC283130; FKSG44; SCYL1; LTBP3; SSSCA1; MTVR1; KCNK7; MAP3K11; SIPA1; RELA; HTATIP; AYP1 |
| 4054 | LTBP3 | 11 | 65062851 | 11q12 | gneg 6; | 1.03 | 0.2794194 | 0.363 | -0.206 | 0.136 | 0.377 | MRPL49; HRD1; CAPN1; POLA2; CDC42EP2; DPF2; TIGD3; LOC283130; FKSG44; SCYL1; LTBP3; SSSCA1; MTVR1; KCNK7; MAP3K11; SIPA1; RELA; HTATIP; AYP1; LOC91056; OVOL1 |
| 51585 | PCF11 | 11 | 82545860 | 11q13 | gneg 3; gpos 9; | 1.38 | 0.0963881 | 0.332 | 0.308 | 0.049 | -0.564 | NDUFC2; ALG8; GAB2; FLJ23441; ODZ4; MGC33846; PRCP; FLJ25416; RAB30; 399933; PCF11; FLJ37874; MDS025; FLJ37266; DLG2; 400690; HT007; DKFZp586C1924; ZF; FLJ38159; SYTL2 |
| 219595 | PSMAL/GCP III | 11 | 89032112 | 11q14.3 | gneg 3; gpos 9; | 3.36 | 0.2904118 | -0.046 | 0.201 | 0.038 | -0.369 | FLJ23514; ME3; SPUVE; FZD4; FLJ22104; RAB38; CTSC; GRM5; TYR; NOX4; PSMAL/GCP; RNF18; 399940; 120146; NAALAD2; CHORDC1; KIAA1917; 120105; FAT3; MTNR1B; SLC36A4 |
| 6588 | SLN | 11 | 107083318 | 11q22-q23 | gneg 7; gpos 1; | -79.72 | 0.0014943 | 0.322 | -0.522 | 0.149 | 0.958 | COP; ICEBERG; GRIA4; KIAA1826; KBTBD3; AASDHPPT; GUCY1A2; FLJ32343; LOC91801; DKFZp547C176; SLN; SLC35F2; CUL5; ACAT1; NPAT; ATM; MGC33948; MGC33424; SLAC2-B; DDX10; RDX |
| 4863 | NPAT | 11 | 107533329 | 11q22-q23 | gneg 7; gpos 3; | 2.01 | 0.0006373 | 0.318 | 0.549 | 0.142 | -1.006 | KBTBD3; AASDHPPT; GUCY1A2; FLJ32343; LOC91801; DKFZp547C176; SLN; SLC35F2; CUL5; ACAT1; NPAT; ATM; MGC33948; MGC33424; SLAC2-B; DDX10; RDX; FDX1; ARHGAP20; 402057; MGC50104 |
| 22822 | PHLDA1 | 12 | 74709366 | 12q15 | gneg 5; gpos 11; | 2.4 | 0.0206377 | 0.311 | 0.41 | 0.099 | -0.752 | RAB21; FLJ12085; TPH2; TRHDE; KCNC2; CAPS2; MGC26856; MGC39497; GLIPR1; HRB2; PHLDA1; NAP1L1; FLJ23560; OSBPL8; HIP14; CSRP2; E2F7; NAV3; SYT1; 338756; PAWR |
| 4673 | NAP1L1 | 12 | 74726224 | 12q21.1 | gneg 4; gpos 11; | 1.6 | 0.0050143 | 0.311 | 0.478 | -0.091 | -0.876 | FLJ12085; TPH2; TRHDE; KCNC2; CAPS2; MGC26856; MGC39497; GLIPR1; HRB2; PHLDA1; NAP1L1; FLJ23560; OSBPL8; HIP14; CSRP2; E2F7; NAV3; SYT1; 338756; PAWR; PPP1R12A |
| 1466 | CSRP2 | 12 | 75754963 | 12q21.1 | gneg 3; gpos 10; | -1.16 | 0.0822881 | 0.319 | -0.32 | 0.027 | 0.587 | CAPS2; MGC26856; MGC39497; GLIPR1; HRB2; PHLDA1; NAP1L1; FLJ23560; OSBPL8; HIP14; CSRP2; E2F7; NAV3; SYT1; 338756; PAWR; PPP1R12A; FLJ90579; 387870; MYF6; MYF5 |
| 7334 | UBE2N | 12 | 92304555 | 12q22 | gneg 9; gpos 6; | 1.76 | 0.0124588 | 0.419 | 0.437 | 0.295 | -0.8 | DSPG3; KERA; LUM; DCN; LOC256021; BTG1; 387872; LOC338809; EEA1; NUDT4; UBE2N; MRPL42; SOCS2; CRADD; PLXNC1; NY-REN-58; KIAA1145; DAP13; NR2C1; VEZATIN; METAP2 |
| 28977 | MRPL42 | 12 | 92363737 | 12q22 | gneg 9; gpos 7; | 2.33 | 7.78E-05 | 0.38 | 0.599 | 0.215 | -1.098 | KERA; LUM; DCN; LOC256021; BTG1; 387872; LOC338809; EEA1; NUDT4; UBE2N; MRPL42; SOCS2; CRADD; PLXNC1; NY-REN-58; KIAA1145; DAP13; NR2C1; VEZATIN; METAP2; USP44 |
| 8835 | SOCS2 | 12 | 92466065 | 12q | gneg 8; gpos 7; | 1.01 | 0.607386 | 0.403 | -0.099 | -0.297 | 0.182 | LUM; DCN; LOC256021; BTG1; 387872; LOC338809; EEA1; NUDT4; UBE2N; MRPL42; SOCS2; CRADD; PLXNC1; NY-REN-58; KIAA1145; DAP13; NR2C1; VEZATIN; METAP2; USP44; NTN4 |
| 8738 | CRADD | 12 | 92573618 | 12q21.33-q23.1 | gneg 8; gpos 8; | 1.31 | 0.3440739 | 0.394 | 0.181 | 0.186 | -0.331 | DCN; LOC256021; BTG1; 387872; LOC338809; EEA1; NUDT4; UBE2N; MRPL42; SOCS2; CRADD; PLXNC1; NY-REN-58; KIAA1145; DAP13; NR2C1; VEZATIN; METAP2; USP44; NTN4; SNRPF |
| 55967 | DAP13 | 12 | 93867576 | 12q23.1 | gneg 7; gpos 9; | 1.39 | 0.1865863 | 0.337 | 0.249 | 0.282 | -0.456 | LOC338809; EEA1; NUDT4; UBE2N; MRPL42; SOCS2; CRADD; PLXNC1; NY-REN-58; KIAA1145; DAP13; NR2C1; VEZATIN; METAP2; USP44; NTN4; SNRPF; FLJ40089; MGC35366; HAL; LTA4H |
| 55591 | VEZATIN | 12 | 94148188 | 12q23.1 | gneg 5; gpos 10; | 1.48 | 0.2178984 | 0.341 | 0.233 | -0.276 | -0.427 | NUDT4; UBE2N; MRPL42; SOCS2; CRADD; PLXNC1; NY-REN-58; KIAA1145; DAP13; NR2C1; VEZATIN; METAP2; USP44; NTN4; SNRPF; FLJ40089; MGC35366; HAL; LTA4H; ELK3; PCTK2 |
| 10988 | METAP2 | 12 | 94370389 | 12q23.1 | gneg 5; gpos 11; | 1.96 | 0.0004045 | 0.344 | 0.561 | 0.289 | -1.028 | UBE2N; MRPL42; SOCS2; CRADD; PLXNC1; NY-REN-58; KIAA1145; DAP13; NR2C1; VEZATIN; METAP2; USP44; NTN4; SNRPF; FLJ40089; MGC35366; HAL; LTA4H; ELK3; PCTK2; FLJ44112 |
| 3621 | ING1 | 13 | 110163083 | 13q34 | gneg 13; gpos 2; | 1.44 | 0.4784277 | 0.377 | -0.136 | -0.059 | 0.25 | 122335; LIG4; FLJ14906; TNFSF13B; IRS2; COL4A1; COL4A2; RAB20; FLJ10769; FLJ12118; ING1; 387946; LOC283487; ANKRD10; ARHGEF7; 121792; MGC35169; SOX1; 400161; 401740; 400163 |
| 1241 | LTB4R | 14 | 23852356 | 14q11.2-q12 | gneg 19; | 1.42 | 0.7714569 | 0.386 | 0.056 | 0.002 | -0.103 | MGC5987; NEDD8; GMPR2; TINF2; TGM1; RABGGTA; DHRS1; C14orf21; CIDEB; LTB4R2; LTB4R; ADCY4; RIPK3; NFATC4; KIAA0323; HCDI; CMA1; CTSG; GZMH; GZMB; STXBP6 |
| 55837 | C14orf11 | 14 | 34054886 | 14q13.1 | gneg 5; gpos 11; | 1.2 | 0.8425869 | 0.352 | 0.038 | 0.108 | -0.071 | STRN3; AP4S1; HECTD1; C14orf126; C14orf127; ARHGAP5; AKAP6; NPAS3; EGLN3; C14orf147; C14orf11; SNX6; CFL2; BAZ1A; SRP54; C14orf24; C14orf10; PSMA6; NFKBIA; INSM2; GARNL1 |
| 58533 | SNX6 | 14 | 34100366 | 14q13.1 | gneg 6; gpos 11; | -1.14 | 0.1172732 | 0.3 | -0.291 | -0.114 | 0.534 | AP4S1; HECTD1; C14orf126; C14orf127; ARHGAP5; AKAP6; NPAS3; EGLN3; C14orf147; C14orf11; SNX6; CFL2; BAZ1A; SRP54; C14orf24; C14orf10; PSMA6; NFKBIA; INSM2; GARNL1; 342865 |
| 11177 | BAZ1A | 14 | 34291689 | 14q12-q13 | gneg 6; gpos 10; | 1.05 | 0.6536474 | 0.329 | -0.087 | 0.097 | 0.159 | C14orf126; C14orf127; ARHGAP5; AKAP6; NPAS3; EGLN3; C14orf147; C14orf11; SNX6; CFL2; BAZ1A; SRP54; C14orf24; C14orf10; PSMA6; NFKBIA; INSM2; GARNL1; 342865; MBIP; TITF1 |
| 23116 | KIAA0423 | 14 | 44501160 | 14q21.3 | gneg 8; gpos 9; | -1.49 | 0.0036953 | 0.306 | -0.49 | 0.155 | 0.898 | SEC23A; SIP1; TRAPPC6B; PNN; MIA2; MGEA6; FBXO33; C14orf146; DKFZP434F1017; BTBD5; KIAA0423; PRPF39; FKBP3; KIAA1596; C14orf106; RPL10L; MAMDC1; 284184; RPS29; PPIL5; RPL36AL |
| 55015 | PRPF39 | 14 | 44623073 | 14q21.3 | gneg 7; gpos 10; | 2.09 | 0.0003089 | 0.351 | 0.568 | -0.089 | -1.041 | SIP1; TRAPPC6B; PNN; MIA2; MGEA6; FBXO33; C14orf146; DKFZP434F1017; BTBD5; KIAA0423; PRPF39; FKBP3; KIAA1596; C14orf106; RPL10L; MAMDC1; 284184; RPS29; PPIL5; RPL36AL; C14orf104 |
| 2287 | FKBP3 | 14 | 44654858 | 14q21.3 | gneg 7; gpos 10; | 1.48 | 0.0234097 | 0.371 | 0.403 | 0.17 | -0.739 | TRAPPC6B; PNN; MIA2; MGEA6; FBXO33; C14orf146; DKFZP434F1017; BTBD5; KIAA0423; PRPF39; FKBP3; KIAA1596; C14orf106; RPL10L; MAMDC1; 284184; RPS29; PPIL5; RPL36AL; C14orf104; POLE2 |
| 23768 | FLRT2 | 14 | 85157274 | 14q24-q32 | gneg 2; gpos 11; | -4.18 | 2.76E-05 | 0.319 | -0.618 | -0.108 | 1.134 | FLJ25976; ADCK1; FLJ14502; NRXN3; DIO2; TSHR; GTF2A1; STN2; SEL1L; 283583; FLRT2; 283584; 283586; GALC; GPR65; KCNK10; SPATA7; PTPN21; FLJ11806; EML5; TTC8 |
| 4351 | MPI | 15 | 72969462 | 15q22-qter | gpos 12; | 1.04 | 0.9296513 | 0.33 | 0.017 | -0.075 | -0.031 | MGC14421; DRIL2; CLK3; FLJ21128; CYP1A1; CYP1A2; CSK; LMAN1L; DKFZP434C131; SCAMP2; MPI; C15orf17; COX5A; Rpp25; SCAMP5; MDS018; DKFZP434H132; FLJ20452; NEIL1; MAN2C1; SIN3A |
| 54913 | Rpp25 | 15 | 73034703 | 15q23 | gpos 13; | -1.09 | 0.9718694 | 0.348 | -0.007 | 0.005 | 0.013 | FLJ21128; CYP1A1; CYP1A2; CSK; LMAN1L; DKFZP434C131; SCAMP2; MPI; C15orf17; COX5A; Rpp25; SCAMP5; MDS018; DKFZP434H132; FLJ20452; NEIL1; MAN2C1; SIN3A; RNUT1; C15orf12; MGC32065 |
| 192683 | SCAMP5 | 15 | 73074953 | 15q23 | gpos 13; | 2.11 | 0.1654873 | 0.352 | 0.26 | -0.096 | -0.477 | CYP1A1; CYP1A2; CSK; LMAN1L; DKFZP434C131; SCAMP2; MPI; C15orf17; COX5A; Rpp25; SCAMP5; MDS018; DKFZP434H132; FLJ20452; NEIL1; MAN2C1; SIN3A; RNUT1; C15orf12; MGC32065; CSPG4 |
| 56905 | DKFZP434H132 | 15 | 73281301 | 15q23 | gpos 15; | -1.82 | 0.1499978 | 0.405 | -0.269 | 0.072 | 0.494 | CSK; LMAN1L; DKFZP434C131; SCAMP2; MPI; C15orf17; COX5A; Rpp25; SCAMP5; MDS018; DKFZP434H132; FLJ20452; NEIL1; MAN2C1; SIN3A; RNUT1; C15orf12; MGC32065; CSPG4; MGC48986; LOC92912 |
| 79661 | NEIL1 | 15 | 73426462 | 15q23 | gpos 16; | 1.08 | 0.4145411 | 0.336 | 0.156 | -0.073 | -0.287 | DKFZP434C131; SCAMP2; MPI; C15orf17; COX5A; Rpp25; SCAMP5; MDS018; DKFZP434H132; FLJ20452; NEIL1; MAN2C1; SIN3A; RNUT1; C15orf12; MGC32065; CSPG4; MGC48986; LOC92912; FBXO22; LOC145957 |
| 197336 | LOC197336 | 16 | 654988 | 16p13.3 | gneg 21; | 1.25 | 0.2676238 | 0.343 | 0.21 | -0.041 | -0.386 | Rab11-FIP3; SOLH; LOC146325; FLJ36208; PIGQ; RAB40C; WFIKKN; MGC13114; MGC15416; KIAA1924; LOC197336; ARHT2; RHBDL1; STUB1; 339123; DKFZp434F054; MGC33974; MGC2601; MGC2494; FLJ34512; HAGHL |
| 4913 | NTHL1 | 16 | 2029816 | 16p13.3 | gneg 19; | 2.02 | 0.1034894 | 0.39 | 0.302 | 0.031 | -0.553 | SEPX1; RPL3L; NDUFB10; RPS2; TBL3; NOXO1; GFER; SYNGR3; LOC90850; SLC9A3R2; NTHL1; TSC2; PKD1; RAB26; RFWD1; CASKIN1; GBL; MGC21830; LOC283871; E4F1; DNASE1L2 |
| 7249 | TSC2 | 16 | 2038599 | 16p13.3 | gneg 19; | -1.27 | 0.1064668 | 0.384 | -0.299 | 0.035 | 0.549 | RPL3L; NDUFB10; RPS2; TBL3; NOXO1; GFER; SYNGR3; LOC90850; SLC9A3R2; NTHL1; TSC2; PKD1; RAB26; RFWD1; CASKIN1; GBL; MGC21830; LOC283871; E4F1; DNASE1L2; DCI |
| 5310 | PKD1 | 16 | 2078711 | 16p13.3 | gneg 19; | -1.36 | 0.0002955 | 0.31 | -0.569 | 0.032 | 1.043 | NDUFB10; RPS2; TBL3; NOXO1; GFER; SYNGR3; LOC90850; SLC9A3R2; NTHL1; TSC2; PKD1; RAB26; RFWD1; CASKIN1; GBL; MGC21830; LOC283871; E4F1; DNASE1L2; DCI; RNPS1 |
| 84231 | RFWD1 | 16 | 2145799 | 16p13.3 | gneg 19; | 1.36 | 0.1875446 | 0.405 | 0.248 | 0 | -0.455 | TBL3; NOXO1; GFER; SYNGR3; LOC90850; SLC9A3R2; NTHL1; TSC2; PKD1; RAB26; RFWD1; CASKIN1; GBL; MGC21830; LOC283871; E4F1; DNASE1L2; DCI; RNPS1; ABCA3; FLJ13909 |
| 1877 | E4F1 | 16 | 2213589 | 16p13.3 | gneg 21; | -1.19 | 0.0875883 | 0.342 | -0.315 | 0.124 | 0.578 | SLC9A3R2; NTHL1; TSC2; PKD1; RAB26; RFWD1; CASKIN1; GBL; MGC21830; LOC283871; E4F1; DNASE1L2; DCI; RNPS1; ABCA3; FLJ13909; NTN2L; ATP6V0C; CGI-14; PDPK1; FLJ39075 |
| 21 | ABCA3 | 16 | 2265883 | 16p13.3 | gneg 21; | 1.33 | 0.2499358 | 0.348 | 0.218 | 0.044 | -0.4 | RAB26; RFWD1; CASKIN1; GBL; MGC21830; LOC283871; E4F1; DNASE1L2; DCI; RNPS1; ABCA3; FLJ13909; NTN2L; ATP6V0C; CGI-14; PDPK1; FLJ39075; KCTD5; MPN; LOC146443; SRRM2 |
| 1387 | CREBBP | 16 | 3716569 | 16p13.3 | gneg 20; | 1.25 | 0.3569323 | 0.306 | 0.176 | -0.128 | -0.323 | ZNF434; ZNF174; FLJ33071; FLJ14154; KIAA0643; NOD3; BTBD12; KIAA1987; DNASE1; TRAP1; CREBBP; ADCY9; SRL; TFAP4; GLIS2; Magmas; FLJ22021; KIAA0542; LOC114990; DNAJA3; HSCARG |
| 7284 | TUFM | 16 | 28761613 | 16p11.2 | gneg 5; gpos 13; | 1.31 | 0.1185055 | 0.376 | 0.29 | 0.15 | -0.532 | MGC18079; EIF3S8; CLN3; 400514; IL27; P8; LOC112869; SULT1A2; SULT1A1; A2LP; TUFM; SH2B; ATP2A1; FRA; CD19; FLJ14639; SPINL; LAT; MGC5178; SULT1A3; QPRT |
| 23475 | QPRT | 16 | 29597941 | 16p12.1 | gneg 5; gpos 15; | -1.34 | 0.0723548 | 0.315 | -0.33 | 0.04 | 0.605 | TUFM; SH2B; ATP2A1; FRA; CD19; FLJ14639; SPINL; LAT; MGC5178; SULT1A3; QPRT; FLJ35681; ZG16; KIF22; MAZ; LOC112476; MGC4606; MVP; CDIPT; PSK-1; LOC253982 |
| 8479 | HIRIP3 | 16 | 29911817 | 16p12.1 | gneg 4; gpos 13; | 1.19 | 0.2742949 | 0.308 | 0.208 | 0.15 | -0.381 | KIF22; MAZ; LOC112476; MGC4606; MVP; CDIPT; PSK-1; LOC253982; KCTD13; TAO1; HIRIP3; FLJ90652; DOC2A; DKFZP434I2117; ALDOA; PPP4C; TBX6; MGC10500; MGC4171; MAPK3; CORO1A |
| 10295 | BCKDK | 16 | 31027221 | 16p11.2 | gneg 16; | 1.07 | 0.236274 | 0.301 | -0.224 | 0.107 | 0.411 | CTF1; LOC283932; DKFZP434K0410; MGC13024; HSD3B7; STX1B2; STX4A; FLJ13479; KIAA0296; IMAGE3455200; BCKDK; MYST1; PRSS8; FLJ90661; FUS; ASC; PYC1; ITGAM; ITGAX; COX6A2; MGC46336 |
| 2521 | FUS | 16 | 31098959 | 16p11.2 | gneg 16; | 1.93 | 0.0096568 | 0.308 | 0.449 | -0.409 | -0.823 | HSD3B7; STX1B2; STX4A; FLJ13479; KIAA0296; IMAGE3455200; BCKDK; MYST1; PRSS8; FLJ90661; FUS; ASC; PYC1; ITGAM; ITGAX; COX6A2; MGC46336; FLJ13063; TGFB1I1; SLC5A2; FLJ13868 |
| 50855 | PARD6A | 16 | 66252362 | 16q22.1 | gneg 16; | 3.88 | 0.0087074 | 0.305 | 0.454 | 0.161 | -0.832 | FLJ11004; CGI-38; ZDHHC1; HSD11B2; ATP6V0D1; AGRP; FLJ13725; CTCF; LOC146206; 24432; PARD6A; DKFZP434A1319; 388284; MGC11335; RANBP10; TSNAXIP1; FLJ13111; THAP11; NUTF2; RCD-8; UNQ2446 |
| 23450 | SF3B3 | 16 | 69115246 | 16q22.1 | gneg 13; gpos 5; | 1.56 | 0.0145729 | 0.318 | 0.429 | 0.116 | -0.786 | LOC348174; PDPR; MGC34761; MTR3; AARS; DDX19; FLJ11126; SIAT4B; FUK; COG4; SF3B3; MGC34647; FLJ10305; HYDIN; FLJ11171; CALB2; ZNF23; ZNF19; CHST4; TAT; LOC91862 |
| 1984 | EIF5A | 17 | 7151692 | 17p13-p12 | gneg 2; gpos 11; | 3.63 | 0.008608 | 0.301 | 0.454 | -0.152 | -0.833 | DLG4; ACADVL; DVL2; MGC2941; GABARAP; DULLARD; DERP6; CLDN7; SLC2A4; YBX2; EIF5A; GPS2; 84461; CENTB1; KCTD11; LOC339168; TNK1; PLSCR3; MGC40107; NLGN2; LOC374768 |
| 57048 | PLSCR3 | 17 | 7233778 | 17p13.2 | gneg 4; gpos 10; | 1.13 | 0.5778919 | 0.328 | -0.107 | -0.081 | 0.197 | CLDN7; SLC2A4; YBX2; EIF5A; GPS2; 84461; CENTB1; KCTD11; LOC339168; TNK1; PLSCR3; MGC40107; NLGN2; LOC374768; LOC201243; FLJ36878; FGF11; CHRNB1; ZBTB4; POLR2A; TNFSF12 |
| 6665 | SOX15 | 17 | 7432221 | 17p13 | gneg 6; gpos 5; | 3.25 | 0.0285746 | 0.36 | 0.392 | 0.176 | -0.718 | CHRNB1; ZBTB4; POLR2A; TNFSF12; 407977; SENP3; EIF4A1; CD68; 400571; MPDU1; SOX15; FXR2; SAT2; SHBG; ATP1B2; TP53; FLJ10385; EFNB3; 284158; KIAA0346; 92162 |
| 27077 | EPPB9 | 17 | 19187080 | 17p11.2 | acen 1; gneg 17; gpos 1; | 1.57 | 0.0145714 | 0.321 | 0.429 | -0.074 | -0.786 | LOC220594; FLJ11800; FLJ36492; C17orf1A; FAM18B; PRPSAP2; FLJ25217; GRAP; 388348; EPN2; EPPB9; MAPK7; MFAP4; ZNF179; FLJ10847; ALDH3A2; FLJ31196; ALDH3A1; ULK2; AKAP10; HCMOGT-1 |
| 55244 | FLJ10847 | 17 | 19377758 | 17p11.2 | acen 1; gneg 17; gpos 1; | -1.84 | 0.1800767 | 0.317 | -0.252 | 0.046 | 0.462 | FAM18B; PRPSAP2; FLJ25217; GRAP; 388348; EPN2; EPPB9; MAPK7; MFAP4; ZNF179; FLJ10847; ALDH3A2; FLJ31196; ALDH3A1; ULK2; AKAP10; HCMOGT-1; 256223; DKFZp566O084; C17orf35; MGC33894 |
| 224 | ALDH3A2 | 17 | 19492682 | 17p11.2 | acen 1; gneg 17; gpos 1; | 2.14 | 0.0615346 | 0.325 | 0.342 | -0.053 | -0.626 | PRPSAP2; FLJ25217; GRAP; 388348; EPN2; EPPB9; MAPK7; MFAP4; ZNF179; FLJ10847; ALDH3A2; FLJ31196; ALDH3A1; ULK2; AKAP10; HCMOGT-1; 256223; DKFZp566O084; C17orf35; MGC33894; MAP2K3 |
| 113235 | MGC9564 | 17 | 23750178 | 17q11.2 | gneg 12; gpos 2; | -1.1 | 0.0397221 | 0.302 | -0.371 | -0.04 | 0.681 | NLK; PYY2; PPY2; FLJ40504; LOC90410; TNFAIP1; PDIP38; MGC45714; VTN; SARM1; MGC9564; SLC13A2; FOXN1; UNC119; PIGS; ALDOC; SPAG5; FLJ25006; SDF2; MGC39650; RAB34 |
| 10615 | SPAG5 | 17 | 23928714 | 17q11.2 | acen 1; gneg 12; gpos 2; | 1.32 | 0.0991451 | 0.32 | 0.305 | 0.008 | -0.56 | PDIP38; MGC45714; VTN; SARM1; MGC9564; SLC13A2; FOXN1; UNC119; PIGS; ALDOC; SPAG5; FLJ25006; SDF2; MGC39650; RAB34; RPL23A; LOC116238; NEK8; TRAF4; FLJ10700; ERAL1 |
| 8687 | KRTHA8 | 17 | 36846146 | 17q12-q21 | gpos 2; | 1.23 | 0.6099166 | 0.305 | -0.098 | 0.482 | 0.181 | KRTAP4-10; KRTAP9-2; KRTAP9-3; KRTAP9-4; KRTAP17-1; KRTHA3A; KRTHA3B; KRTHA4; KRTHA1; KRTHA7; KRTHA8; KRTHA2; KRTHA5; KRTHA6; KRT13; KRT15; KRT19; KRT9; KRT14; KRT16; KRT17 |
| 3886 | KRTHA5 | 17 | 36886466 | 17q12-q21 | gneg 1; gpos 2; | 1.39 | 0.7541849 | 0.318 | 0.061 | 0.462 | -0.111 | KRTAP9-3; KRTAP9-4; KRTAP17-1; KRTHA3A; KRTHA3B; KRTHA4; KRTHA1; KRTHA7; KRTHA8; KRTHA2; KRTHA5; KRTHA6; KRT13; KRT15; KRT19; KRT9; KRT14; KRT16; KRT17; SUI1; GAS |
| 8689 | KRTHA6 | 17 | 36896153 | 17q12-q21 | gneg 1; gpos 2; | 1.4 | 0.5916146 | 0.366 | 0.104 | 0.195 | -0.19 | KRTAP9-4; KRTAP17-1; KRTHA3A; KRTHA3B; KRTHA4; KRTHA1; KRTHA7; KRTHA8; KRTHA2; KRTHA5; KRTHA6; KRT13; KRT15; KRT19; KRT9; KRT14; KRT16; KRT17; SUI1; GAS; HAP1 |
| 3866 | KRT15 | 17 | 36923523 | 17q21.2 | gneg 2; gpos 2; | 1.23 | 0.8727952 | 0.326 | -0.031 | 0.394 | 0.057 | KRTHA3A; KRTHA3B; KRTHA4; KRTHA1; KRTHA7; KRTHA8; KRTHA2; KRTHA5; KRTHA6; KRT13; KRT15; KRT19; KRT9; KRT14; KRT16; KRT17; SUI1; GAS; HAP1; JUP; SC65 |
| 3861 | KRT14 | 17 | 36992058 | 17q12-q21 | gneg 4; gpos 3; | 1.27 | 0.5524495 | 0.354 | 0.114 | 0.382 | -0.21 | KRTHA1; KRTHA7; KRTHA8; KRTHA2; KRTHA5; KRTHA6; KRT13; KRT15; KRT19; KRT9; KRT14; KRT16; KRT17; SUI1; GAS; HAP1; JUP; SC65; FKBP10; MGC20781; KLHL10 |
| 3868 | KRT16 | 17 | 37019558 | 17q12-q21 | gneg 5; gpos 3; | -1.2 | 0.0355552 | 0.338 | -0.378 | 0.251 | 0.694 | KRTHA7; KRTHA8; KRTHA2; KRTHA5; KRTHA6; KRT13; KRT15; KRT19; KRT9; KRT14; KRT16; KRT17; SUI1; GAS; HAP1; JUP; SC65; FKBP10; MGC20781; KLHL10; FLJ10572 |
| 6774 | STAT3 | 17 | 37718868 | 17q21 | gneg 10; gpos 1; | 1.71 | 0.0279238 | 0.37 | 0.393 | -0.171 | -0.72 | 201181; LGP2; GCN5L2; HspB9; RAB5C; KCNH4; HCRT; LGP1; STAT5B; STAT5A; STAT3; PTRF; ATP6V0A1; NAGLU; HSD17B1; DPCK; TCFL4; HUMGT198A; LOC162427; TUBG1; TUBG2 |
| 1353 | COX11 | 17 | 50384265 | 17q22 | gneg 5; gpos 8; | -1.19 | 0.0654226 | 0.324 | -0.337 | 0.155 | 0.618 | SPAG9; NME1; NME2; MBTD1; CGI-48; 400605; CA10; 339209; LOC84643; TOM1L1; COX11; STXBP4; HLF; MMD; FLJ10970; PCTP; FLJ38335; 342600; NOG; DGKE; ZNF147 |
| 1468 | SLC25A10 | 17 | 77289775 | 17q25.3 | gneg 12; | 1.75 | 0.191005 | 0.35 | 0.246 | 0.404 | -0.451 | FLJ38792; ACTG1; FSCN2; FLJ22175; NPL4; OCSP; PDE6G; LOC339231; HGS; MRPL12; SLC25A10; P4HB; ARHGDIA; THOC4; ANAPC11; NPB; PCYT2; SIRT7; MAFG; PYCR1; 255275 |
| 5034 | P4HB | 17 | 77394325 | 17q25 | gneg 12; | -1.31 | 0.0022555 | 0.326 | -0.508 | -0.217 | 0.932 | ACTG1; FSCN2; FLJ22175; NPL4; OCSP; PDE6G; LOC339231; HGS; MRPL12; SLC25A10; P4HB; ARHGDIA; THOC4; ANAPC11; NPB; PCYT2; SIRT7; MAFG; PYCR1; 255275; LOC147111 |
| 396 | ARHGDIA | 17 | 77418886 | 17q25.3 | gneg 12; | -1.2 | 0.0170992 | 0.31 | -0.42 | -0.006 | 0.771 | FSCN2; FLJ22175; NPL4; OCSP; PDE6G; LOC339231; HGS; MRPL12; SLC25A10; P4HB; ARHGDIA; THOC4; ANAPC11; NPB; PCYT2; SIRT7; MAFG; PYCR1; 255275; LOC147111; ASPSCR1 |
| 10189 | THOC4 | 17 | 77439015 | 17q25.3 | gneg 13; | 1.71 | 0.0138243 | 0.327 | 0.431 | 0.431 | -0.791 | FLJ22175; NPL4; OCSP; PDE6G; LOC339231; HGS; MRPL12; SLC25A10; P4HB; ARHGDIA; THOC4; ANAPC11; NPB; PCYT2; SIRT7; MAFG; PYCR1; 255275; LOC147111; ASPSCR1; STRA13 |
| 5833 | PCYT2 | 17 | 77455383 | 17q25.3 | gneg 14; | 1.07 | 0.3257993 | 0.307 | -0.187 | 0.546 | 0.344 | PDE6G; LOC339231; HGS; MRPL12; SLC25A10; P4HB; ARHGDIA; THOC4; ANAPC11; NPB; PCYT2; SIRT7; MAFG; PYCR1; 255275; LOC147111; ASPSCR1; STRA13; MGC20806; RAC3; DCXR |
| 56651 | C18orf2 | 18 | 1244389 | 18p11 | gneg 6; gpos 5; | 3.19 | 0.3043104 | 0.313 | 0.196 | -0.066 | -0.359 | USP14; THOC1; COLEC12; CETN1; CLUL1; TYMS; HSRTSBETA; YES1; ADCYAP1; 388456; C18orf2; METTL4; KNTC2; EMILIN2; LPIN2; MYOM1; MRCL3; MRLC2; TGIF; DLGAP1; ZFP161 |
| 103910 | MRLC2 | 18 | 3252134 | 18p11.31 | gneg 2; gpos 5; | -1.04 | 0.1068213 | 0.318 | -0.299 | 0.013 | 0.548 | YES1; ADCYAP1; 388456; C18orf2; METTL4; KNTC2; EMILIN2; LPIN2; MYOM1; MRCL3; MRLC2; TGIF; DLGAP1; ZFP161; EPB41L3; FLJ35936; 388461; 400643; LAMA1; PTPRM; NDUFV2 |
| 5725 | PTBP1 | 19 | 748410 | 19p13.3 | gneg 20; | 1.39 | 0.0313254 | 0.344 | 0.386 | 0 | -0.708 | CDC34; GZMM; BSG; HCN2; POLRMT; FGF22; RNF126; FSTL3; PALM; LOC126353; PTBP1; FLJ11535; AZU1; PRTN3; ELA2; DF; TRAP95; MGC16353; GPR54; DRIL1; WDR18 |
| 79948 | FLJ11535 | 19 | 763517 | 19p13.3 | gneg 20; | -1.24 | 0.0670217 | 0.37 | -0.335 | 0.159 | 0.615 | GZMM; BSG; HCN2; POLRMT; FGF22; RNF126; FSTL3; PALM; LOC126353; PTBP1; FLJ11535; AZU1; PRTN3; ELA2; DF; TRAP95; MGC16353; GPR54; DRIL1; WDR18; GRIN3B |
| 10025 | TRAP95 | 19 | 818961 | 19p13.3 | acen 1; gneg 19; | 1.11 | 0.8536704 | 0.319 | 0.036 | -0.004 | -0.066 | RNF126; FSTL3; PALM; LOC126353; PTBP1; FLJ11535; AZU1; PRTN3; ELA2; DF; TRAP95; MGC16353; GPR54; DRIL1; WDR18; GRIN3B; C19orf6; CNN2; ABCA7; HA-1; POLR2E |
| 84634 | GPR54 | 19 | 868502 | 19p13.3 | acen 1; gneg 20; | 4.57 | 0.0407238 | 0.357 | 0.37 | -0.109 | -0.678 | PALM; LOC126353; PTBP1; FLJ11535; AZU1; PRTN3; ELA2; DF; TRAP95; MGC16353; GPR54; DRIL1; WDR18; GRIN3B; C19orf6; CNN2; ABCA7; HA-1; POLR2E; GPX4; STK11 |
| 5605 | MAP2K2 | 19 | 4041321 | 7q32 | gneg 19; | 1.05 | 0.2767723 | 0.309 | -0.207 | -0.34 | 0.379 | MRPL54; MGC15631; MATK; KIAA1086; ATCAY; ITGB1BP3; DAPK3; EEF2; PIASY; FBI1; MAP2K2; CREB3L3; SIRT6; KIAA1981; EBI3; FLJ10374; SHD; MGC23244; FSD1; STAP2; FLJ14981 |
| 51548 | SIRT6 | 19 | 4125105 | 19p13.3 | gneg 19; | 1.2 | 0.7624272 | 0.312 | -0.059 | 0.35 | 0.107 | MATK; KIAA1086; ATCAY; ITGB1BP3; DAPK3; EEF2; PIASY; FBI1; MAP2K2; CREB3L3; SIRT6; KIAA1981; EBI3; FLJ10374; SHD; MGC23244; FSD1; STAP2; FLJ14981; SH3GL1; CHAF1A |
| 170961 | KIAA1981 | 19 | 4163657 | 19p13.3 | gneg 18; | 3.49 | 0.0053918 | 0.337 | 0.475 | 0.341 | -0.87 | KIAA1086; ATCAY; ITGB1BP3; DAPK3; EEF2; PIASY; FBI1; MAP2K2; CREB3L3; SIRT6; KIAA1981; EBI3; FLJ10374; SHD; MGC23244; FSD1; STAP2; FLJ14981; SH3GL1; CHAF1A; UBXD1 |
| 10908 | NTE | 19 | 7505074 | 19p13.3-p13.2 | gneg 15; | -1.04 | 0.2915955 | 0.303 | -0.201 | 0.226 | 0.368 | EMR1; EMR4; MBD3L2; MGC4054; INSR; ARHGEF18; PEX11G; FLJ35784; ZNF358; MCOLN1; NTE; XAB2; STXBP2; RETN; LOC199675; TRAPPC5; FCER2; UNQ431; CD209; LOC115704; FLJ23420 |
| 53637 | EDG8 | 19 | 10484622 | 19p13.2 | gneg 1; gpos 15; | 1.21 | 0.6838314 | 0.338 | 0.079 | -0.192 | -0.144 | 402414; ICAM4; ICAM5; MGC19604; RAVER1; ICAM3; TYK2; CDC37; PDE4A; KEAP1; EDG8; AUTL4; FLJ12949; CDKN2D; AP1M2; CTL2; ILF3; QTRT1; DNM2; IL1RL1LG; 255809 |
| 65095 | FLJ12949 | 19 | 10524760 | 19p13.2 | gneg 1; gpos 17; | 1.33 | 0.3435611 | 0.335 | 0.181 | -0.062 | -0.332 | ICAM5; MGC19604; RAVER1; ICAM3; TYK2; CDC37; PDE4A; KEAP1; EDG8; AUTL4; FLJ12949; CDKN2D; AP1M2; CTL2; ILF3; QTRT1; DNM2; IL1RL1LG; 255809; CARM1; MGC3262 |
| 10053 | AP1M2 | 19 | 10544346 | 19p13.2 | gneg 1; gpos 17; | 3.87 | 0.0024505 | 0.429 | 0.505 | 0.027 | -0.926 | RAVER1; ICAM3; TYK2; CDC37; PDE4A; KEAP1; EDG8; AUTL4; FLJ12949; CDKN2D; AP1M2; CTL2; ILF3; QTRT1; DNM2; IL1RL1LG; 255809; CARM1; MGC3262; LOC90580; SMARCA4 |
| 57153 | CTL2 | 19 | 10597228 | 19p13.1 | gneg 2; gpos 16; | -1.12 | 0.0181995 | 0.339 | -0.417 | 0.092 | 0.765 | ICAM3; TYK2; CDC37; PDE4A; KEAP1; EDG8; AUTL4; FLJ12949; CDKN2D; AP1M2; CTL2; ILF3; QTRT1; DNM2; IL1RL1LG; 255809; CARM1; MGC3262; LOC90580; SMARCA4; LDLR |
| 3609 | ILF3 | 19 | 10625987 | 19p13.2 | gneg 2; gpos 17; | 2.75 | 2.17E-05 | 0.402 | 0.622 | 0.075 | -1.141 | TYK2; CDC37; PDE4A; KEAP1; EDG8; AUTL4; FLJ12949; CDKN2D; AP1M2; CTL2; ILF3; QTRT1; DNM2; IL1RL1LG; 255809; CARM1; MGC3262; LOC90580; SMARCA4; LDLR; FLJ90806 |
| 81890 | QTRT1 | 19 | 10673130 | 19p13.3 | gneg 2; gpos 17; | 1.72 | 0.3581203 | 0.417 | 0.176 | -0.142 | -0.322 | CDC37; PDE4A; KEAP1; EDG8; AUTL4; FLJ12949; CDKN2D; AP1M2; CTL2; ILF3; QTRT1; DNM2; IL1RL1LG; 255809; CARM1; MGC3262; LOC90580; SMARCA4; LDLR; FLJ90806; KIAA1518 |
| 1785 | DNM2 | 19 | 10689773 | 19p13.2 | gneg 2; gpos 17; | 1.11 | 0.6981927 | 0.457 | -0.075 | -0.186 | 0.137 | PDE4A; KEAP1; EDG8; AUTL4; FLJ12949; CDKN2D; AP1M2; CTL2; ILF3; QTRT1; DNM2; IL1RL1LG; 255809; CARM1; MGC3262; LOC90580; SMARCA4; LDLR; FLJ90806; KIAA1518; DOCK6 |
| 10498 | CARM1 | 19 | 10843252 | 19p13.2 | gneg 2; gpos 17; | 1.52 | 0.0471985 | 0.381 | 0.36 | 0.049 | -0.66 | AUTL4; FLJ12949; CDKN2D; AP1M2; CTL2; ILF3; QTRT1; DNM2; IL1RL1LG; 255809; CARM1; MGC3262; LOC90580; SMARCA4; LDLR; FLJ90806; KIAA1518; DOCK6; LOC55908; TM4-B; RAB3D |
| 6597 | SMARCA4 | 19 | 10932605 | 19p13.2 | gneg 2; gpos 17; | 1.29 | 0.2899209 | 0.308 | 0.201 | -0.016 | -0.369 | AP1M2; CTL2; ILF3; QTRT1; DNM2; IL1RL1LG; 255809; CARM1; MGC3262; LOC90580; SMARCA4; LDLR; FLJ90806; KIAA1518; DOCK6; LOC55908; TM4-B; RAB3D; LPPR2; FLJ35119; EPOR |
| 2193 | FARSLA | 19 | 12894292 | 19p13.2 | gneg 5; gpos 13; gvar 1; | 1.52 | 0.1406985 | 0.303 | 0.275 | 0.32 | -0.505 | VMD2L1; HOOK2; JUNB; PRDX2; RNASEH2A; RTBDN; SAST; DNASE2; KLF1; GCDH; FARSLA; CALR; RAD23A; PLINP-1; FLJ38607; NFIX; LYL1; FLJ20244; BTBD14B; STX10; ETR101 |
| 115098 | LOC115098 | 19 | 17906904 | 19p13.12 | gneg 3; gpos 9; | 1.01 | 0.1760792 | 0.345 | -0.254 | 0.28 | 0.466 | PGLS; BCNP1; FLJ22329; VCY2IP1; FCHO1; B3GNT3; INSL3; JAK3; RPL18A; SLC5A5; LOC115098; KCNN1; CLONE24945; IL12RB1; PIK3R2; IFI30; MGC12972; RAB3A; PDE4C; JUND; LSM4 |
| 27106 | CLONE24945 | 19 | 17979976 | 19p13.12 | gneg 4; gpos 7; | 2.75 | 0.0158252 | 0.345 | 0.424 | 0.063 | -0.778 | FLJ22329; VCY2IP1; FCHO1; B3GNT3; INSL3; JAK3; RPL18A; SLC5A5; LOC115098; KCNN1; CLONE24945; IL12RB1; PIK3R2; IFI30; MGC12972; RAB3A; PDE4C; JUND; LSM4; PGPEP1; PLAB |
| 54929 | FLJ20422 | 19 | 19091430 | 19p13.11 | gneg 10; gpos 1; gvar 7; | 1.3 | 0.5210507 | 0.326 | 0.124 | -0.147 | -0.226 | MECT1; COMP; RENT1; GDF1; LASS1; COPE; DDX49; HOMER3; SFRS14; MGC19595; FLJ20422; MEF2B; RFXANK; TRA16; CSPG3; 404037; SF4; KIAA0892; p66alpha; SSTK; GRIM19 |
| 54815 | p66alpha | 19 | 19430396 | 19p13.11 | gneg 11; gpos 1; gvar 6; | 1.96 | 0.0015268 | 0.335 | 0.522 | -0.073 | -0.956 | SFRS14; MGC19595; FLJ20422; MEF2B; RFXANK; TRA16; CSPG3; 404037; SF4; KIAA0892; p66alpha; SSTK; GRIM19; FLJ44968; CLIP-2; PBX4; EDG4; GMIP; CGI-152; HZF12; ZNF14 |
| 83983 | SSTK | 19 | 19486027 | 19p13.11 | gneg 12; gpos 1; gvar 5; | 1.45 | 0.1109172 | 0.388 | 0.296 | -0.157 | -0.543 | MGC19595; FLJ20422; MEF2B; RFXANK; TRA16; CSPG3; 404037; SF4; KIAA0892; p66alpha; SSTK; GRIM19; FLJ44968; CLIP-2; PBX4; EDG4; GMIP; CGI-152; HZF12; ZNF14; ZNF253 |
| 126299 | MGC51082 | 19 | 48803216 | 19q13.32 | gpos 18; | -1.38 | 0.0012128 | 0.366 | -0.529 | 0.139 | 0.97 | PSG2; PSG5; PSG4; PSG9; PRV1; MGC4766; LOC284345; ETHE1; LOC284346; MGC2508; MGC51082; TSLL2; PLAUR; R30953_1; FLJ12886; KCNN4; FLJ30469; ZNF283; ZNF45; ZNF221; ZNF155 |
| 5329 | PLAUR | 19 | 48844571 | 19q13 | gpos 19; | 1.93 | 0.5875524 | 0.304 | -0.105 | 0.145 | 0.192 | PSG4; PSG9; PRV1; MGC4766; LOC284345; ETHE1; LOC284346; MGC2508; MGC51082; TSLL2; PLAUR; R30953_1; FLJ12886; KCNN4; FLJ30469; ZNF283; ZNF45; ZNF221; ZNF155; ZNF230; ZNF222 |
| 3783 | KCNN4 | 19 | 48962524 | 19q13.2 | gpos 19; | 1.66 | 0.5842376 | 0.309 | -0.106 | 0.129 | 0.194 | MGC4766; LOC284345; ETHE1; LOC284346; MGC2508; MGC51082; TSLL2; PLAUR; R30953_1; FLJ12886; KCNN4; FLJ30469; ZNF283; ZNF45; ZNF221; ZNF155; ZNF230; ZNF222; ZNF223; ZNF224; ZNF225 |
| 90843 | MGC45400 | X | 102314070 | Xq22.2 | gneg 6; gpos 7; | 1.17 | 0.9662998 | 0.34 | -0.008 | 0.469 | 0.015 | my048; LOC340542; NXF2; TMSNB; FLJ12969; LOC114928; KIAA1701; BEX1; NXF3; FLJ10097; MGC45400; MGC23947; LOC51186; NGFRAP1; RAB40A; FLJ21174; MGC15737; TCEAL1; MORF4L2; MGC39655; PLP1 |
| 27018 | NGFRAP1 | X | 102437412 | Xq22.2 | gneg 4; gpos 9; | 1.21 | 0.52028 | 0.418 | 0.124 | 0.523 | -0.227 | TMSNB; FLJ12969; LOC114928; KIAA1701; BEX1; NXF3; FLJ10097; MGC45400; MGC23947; LOC51186; NGFRAP1; RAB40A; FLJ21174; MGC15737; TCEAL1; MORF4L2; MGC39655; PLP1; RAB9B; MGC39900; LOC158983 |
| 79921 | FLJ21174 | X | 102637303 | Xq22.2 | gneg 5; gpos 10; | 1.48 | 0.3012389 | 0.329 | 0.197 | 0.227 | -0.361 | LOC114928; KIAA1701; BEX1; NXF3; FLJ10097; MGC45400; MGC23947; LOC51186; NGFRAP1; RAB40A; FLJ21174; MGC15737; TCEAL1; MORF4L2; MGC39655; PLP1; RAB9B; MGC39900; LOC158983; LOC286436; ESX1L |
| 85012 | MGC15737 | X | 102668978 | Xq22.2 | gneg 4; gpos 10; | 2.2 | 0.3856911 | 0.338 | 0.166 | 0.12 | -0.304 | KIAA1701; BEX1; NXF3; FLJ10097; MGC45400; MGC23947; LOC51186; NGFRAP1; RAB40A; FLJ21174; MGC15737; TCEAL1; MORF4L2; MGC39655; PLP1; RAB9B; MGC39900; LOC158983; LOC286436; ESX1L; IL1RAPL2 |
| 9338 | TCEAL1 | X | 102690046 | Xq22.1 | gneg 4; gpos 10; | 1.24 | 0.6680832 | 0.337 | 0.083 | 0.359 | -0.152 | BEX1; NXF3; FLJ10097; MGC45400; MGC23947; LOC51186; NGFRAP1; RAB40A; FLJ21174; MGC15737; TCEAL1; MORF4L2; MGC39655; PLP1; RAB9B; MGC39900; LOC158983; LOC286436; ESX1L; IL1RAPL2; TEX13A |
| 9643 | MORF4L2 | X | 102736577 | Xq22 | gneg 5; gpos 10; | 1.72 | 0.0766437 | 0.323 | 0.325 | 0.195 | -0.597 | NXF3; FLJ10097; MGC45400; MGC23947; LOC51186; NGFRAP1; RAB40A; FLJ21174; MGC15737; TCEAL1; MORF4L2; MGC39655; PLP1; RAB9B; MGC39900; LOC158983; LOC286436; ESX1L; IL1RAPL2; TEX13A; DKFZp686A17109 |
| 23439 | ATP1B4 | X | 119277848 | Xq25 | gneg 2; gpos 13; | 1.96 | 0.7607385 | 0.395 | -0.059 | 0.024 | 0.108 | UPF3B; ZNF183; NDUFA1; AKAP28; NKAP; PEPP-2; OTEX; FLJ36576; ZNF-kaiso; FLJ20716; ATP1B4; LAMP2; CUL4B; MCTS1; C1GALT2; LOC255313; GLUD2; GRIA3; THOC2; BIRC4; STAG2 |
| 8450 | CUL4B | X | 119442379 | Xq23 | gneg 2; gpos 14; | -1.1 | 0.0729165 | 0.361 | -0.329 | -0.191 | 0.604 | NDUFA1; AKAP28; NKAP; PEPP-2; OTEX; FLJ36576; ZNF-kaiso; FLJ20716; ATP1B4; LAMP2; CUL4B; MCTS1; C1GALT2; LOC255313; GLUD2; GRIA3; THOC2; BIRC4; STAG2; SH2D1A; ODZ1 |
| 8287 | USP9Y | Y | 13251290 | Yq11.2 | gneg 10; gpos 5; | 1.12 | 0.7183002 | 0.427 | -0.07 | 0.233 | 0.128 | SRY; RPS4Y; ZFY; TGIF2LY; PCDH11Y; TSPYQ1; AMELY; TBL1Y; PRKY; TSPY; USP9Y; DDX3Y; UTY; TMSB4Y; VCY; NLGN4Y; XKRY; CDY2; FLJ25453; CD24; FLJ39821 |
| 8653 | DDX3Y | Y | 13454907 | Yq11 | gneg 11; gpos 5; | -1.21 | 0.7085255 | 0.403 | 0.072 | 0.146 | -0.133 | RPS4Y; ZFY; TGIF2LY; PCDH11Y; TSPYQ1; AMELY; TBL1Y; PRKY; TSPY; USP9Y; DDX3Y; UTY; TMSB4Y; VCY; NLGN4Y; XKRY; CDY2; FLJ25453; CD24; FLJ39821; CYorf15B |
| 7404 | UTY | Y | 13847520 | Yq11 | gneg 11; gpos 5; | -2.09 | 0.1344167 | 0.39 | -0.279 | 0.159 | 0.512 | ZFY; TGIF2LY; PCDH11Y; TSPYQ1; AMELY; TBL1Y; PRKY; TSPY; USP9Y; DDX3Y; UTY; TMSB4Y; VCY; NLGN4Y; XKRY; CDY2; FLJ25453; CD24; FLJ39821; CYorf15B; SMCY |
| 9084 | VCY | Y | 14535782 | Yq11.221 | gneg 12; gpos 5; | 3.12 | 0.1813827 | 0.366 | 0.251 | 0.26 | -0.461 | PCDH11Y; TSPYQ1; AMELY; TBL1Y; PRKY; TSPY; USP9Y; DDX3Y; UTY; TMSB4Y; VCY; NLGN4Y; XKRY; CDY2; FLJ25453; CD24; FLJ39821; CYorf15B; SMCY; EIF1AY; RPS4Y2 |
| 22829 | NLGN4Y | Y | 15074584 | Yq11.21 | gneg 12; gpos 5; | -1.18 | 0.7979699 | 0.519 | 0.05 | 0.045 | -0.091 | TSPYQ1; AMELY; TBL1Y; PRKY; TSPY; USP9Y; DDX3Y; UTY; TMSB4Y; VCY; NLGN4Y; XKRY; CDY2; FLJ25453; CD24; FLJ39821; CYorf15B; SMCY; EIF1AY; RPS4Y2; RBMY1A1 |
| 84663 | CYorf15B | Y | 20142460 | Yq11.222 | gneg 7; gpos 9; | -1.01 | 0.9616912 | 0.361 | 0.009 | 0.177 | -0.017 | DDX3Y; UTY; TMSB4Y; VCY; NLGN4Y; XKRY; CDY2; FLJ25453; CD24; FLJ39821; CYorf15B; SMCY; EIF1AY; RPS4Y2; RBMY1A1; PRY; MGC33094; TTY7; VCY2; DAZ4; DAZ2 |
| 8284 | SMCY | Y | 20255430 | Yq11 | gneg 8; gpos 9; | -1.47 | 0.5764414 | 0.327 | -0.108 | 0.037 | 0.197 | UTY; TMSB4Y; VCY; NLGN4Y; XKRY; CDY2; FLJ25453; CD24; FLJ39821; CYorf15B; SMCY; EIF1AY; RPS4Y2; RBMY1A1; PRY; MGC33094; TTY7; VCY2; DAZ4; DAZ2; DAZ3 |
