# Supplementary material for: Transcriptome coexpression map of human embryonic stem cells
Source: BMC Genomics. 2006 May 2;7:103. doi: 10.1186/1471-2164-7-103 (PMC1523211; doi:10.1186/1471-2164-7-103)
Supplement: Additional File 8 — Supplementary Table S3 (Supplementary Table S3 Coexpression chrom domains in EB.doc). List of coexpression chromosomal domains identified in EB at the co-expression index threshold 0.3 and the window size of 20, and associated GO terms (Fisher P ≤ 0.05). [file 1471-2164-7-103-S8.doc]

Supplementary Table S4. List of coexpression chromosomal domains identified in EB cells at the coexpression index threshold 0.3 and the window size of 20 genes. *gneg - Giemsa negative bands, gpos - Giemsa positive bands, acen - centromeric regions, Gvar - variable length heterochromatic regions. ** P value is derived by ANOVA test between ES and EB. *** the genes are in the from5’ to 3’ order as on the chromosome

| Gene ID | Gene Symbol | Chromosome | Chromosome position | Chromosomal band | Freq in cytogeneric pattern * | Fold change (ES : EB) | P value (ES - EB) ** | Co-Exp Index (ES) | Exp_value (ES) | Co-Exp_Index (EB) | Exp_value (EB) | gene in Domain *** |
| --- | --- | --- | --- | --- | --- | --- | --- | --- | --- | --- | --- | --- |
| 9636 | G1P2 | 1 | 988945 | 1p36.33 | gneg 17; | -1.06 | 0.195375107 | -0.018 | -0.244 | 0.311 | 0.447 | 286080; FLJ22639; LOC284591; FLJ39609; MGC45873; DKFZP564C186; LOC339451; DKFZP434H2010; MGC13275; Hes4; G1P2; FLJ20584; LOC254099; FLJ36119; TNFRSF18; TNFRSF4; Cab45; B3GALT6; UBE2J2; SCNN1D; CENTB5 |
| 54991 | FLJ20584 | 1 | 1057127 | 1p36.33 | gneg 17; | -1.23 | 0.017671672 | 0.075 | -0.419 | 0.336 | 0.767 | FLJ22639; LOC284591; FLJ39609; MGC45873; DKFZP564C186; LOC339451; DKFZP434H2010; MGC13275; Hes4; G1P2; FLJ20584; LOC254099; FLJ36119; TNFRSF18; TNFRSF4; Cab45; B3GALT6; UBE2J2; SCNN1D; CENTB5; FLJ90811 |
| 8784 | TNFRSF18 | 1 | 1178811 | 1p36.3 | gneg 16; | -1.54 | 0.297963492 | -0.053 | -0.198 | 0.332 | 0.363 | MGC45873; DKFZP564C186; LOC339451; DKFZP434H2010; MGC13275; Hes4; G1P2; FLJ20584; LOC254099; FLJ36119; TNFRSF18; TNFRSF4; Cab45; B3GALT6; UBE2J2; SCNN1D; CENTB5; FLJ90811; FLJ20542; DVL1; AKIP |
| 51150 | Cab45 | 1 | 1192211 | 1p36.33 | gneg 15; | -1.56 | 0.057258649 | 0 | -0.347 | 0.396 | 0.636 | LOC339451; DKFZP434H2010; MGC13275; Hes4; G1P2; FLJ20584; LOC254099; FLJ36119; TNFRSF18; TNFRSF4; Cab45; B3GALT6; UBE2J2; SCNN1D; CENTB5; FLJ90811; FLJ20542; DVL1; AKIP; CCNL2; MRPL20 |
| 116983 | CENTB5 | 1 | 1268866 | 1p36 | gneg 14; | 2.76 | 0.895665091 | -0.033 | 0.025 | 0.321 | -0.047 | G1P2; FLJ20584; LOC254099; FLJ36119; TNFRSF18; TNFRSF4; Cab45; B3GALT6; UBE2J2; SCNN1D; CENTB5; FLJ90811; FLJ20542; DVL1; AKIP; CCNL2; MRPL20; TTBK1; 400729; WARP; FLJ37183 |
| 126789 | FLJ90811 | 1 | 1283916 | 1p36.33 | gneg 14; | 1.41 | 0.667947449 | 0.022 | 0.083 | 0.341 | -0.152 | FLJ20584; LOC254099; FLJ36119; TNFRSF18; TNFRSF4; Cab45; B3GALT6; UBE2J2; SCNN1D; CENTB5; FLJ90811; FLJ20542; DVL1; AKIP; CCNL2; MRPL20; TTBK1; 400729; WARP; FLJ37183; TOB3 |
| 54973 | FLJ20542 | 1 | 1286901 | 1p36.33 | gneg 14; | 1.17 | 0.699596941 | 0 | -0.075 | 0.341 | 0.137 | LOC254099; FLJ36119; TNFRSF18; TNFRSF4; Cab45; B3GALT6; UBE2J2; SCNN1D; CENTB5; FLJ90811; FLJ20542; DVL1; AKIP; CCNL2; MRPL20; TTBK1; 400729; WARP; FLJ37183; TOB3; FLJ10709 |
| 54998 | AKIP | 1 | 1349032 | 1p36.33 | gneg 14; | 1.08 | 0.454055907 | 0.073 | -0.144 | 0.421 | 0.264 | TNFRSF18; TNFRSF4; Cab45; B3GALT6; UBE2J2; SCNN1D; CENTB5; FLJ90811; FLJ20542; DVL1; AKIP; CCNL2; MRPL20; TTBK1; 400729; WARP; FLJ37183; TOB3; FLJ10709; 339453; HSPC182 |
| 55052 | MRPL20 | 1 | 1422563 | 1p36.3-p36.2 | gneg 14; | 1.38 | 0.054780375 | -0.062 | 0.35 | 0.371 | -0.641 | Cab45; B3GALT6; UBE2J2; SCNN1D; CENTB5; FLJ90811; FLJ20542; DVL1; AKIP; CCNL2; MRPL20; TTBK1; 400729; WARP; FLJ37183; TOB3; FLJ10709; 339453; HSPC182; CDC2L2; CDC2L1 |
| 9410 | HPRP8BP | 1 | 31401613 | 1p35.1 | gneg 2; gpos 8; | 1.78 | 0.004070072 | 0.06 | 0.486 | 0.357 | -0.891 | OPRD1; 399474; SFRS4; CGI-63; PTPRU; MATN1; LAPTM5; 400748; PUM1; FLJ12650; HPRP8BP; PS1D; 128499; FABP3; TDE2L; LOC284551; LCN7; HCRTR1; PEF; COL16A1; BAI2 |
| 51538 | PS1D | 1 | 31438934 | 1p35.1 | gneg 2; gpos 9; | 1.27 | 0.693543019 | -0.059 | -0.076 | 0.336 | 0.14 | 399474; SFRS4; CGI-63; PTPRU; MATN1; LAPTM5; 400748; PUM1; FLJ12650; HPRP8BP; PS1D; 128499; FABP3; TDE2L; LOC284551; LCN7; HCRTR1; PEF; COL16A1; BAI2; FLJ25348 |
| 128499 | not_found | 1 | 31500894 | 20q13.12 | gneg 2; gpos 10; | -1.41 | 0.001664704 | 0.005 | -0.519 | 0.3 | 0.951 | SFRS4; CGI-63; PTPRU; MATN1; LAPTM5; 400748; PUM1; FLJ12650; HPRP8BP; PS1D; 128499; FABP3; TDE2L; LOC284551; LCN7; HCRTR1; PEF; COL16A1; BAI2; FLJ25348; SEMACAP3 |
| 2170 | FABP3 | 1 | 31507593 | 1p33-p32 | gneg 1; gpos 10; | -1.1 | 0.288135814 | 0.055 | -0.202 | 0.306 | 0.37 | CGI-63; PTPRU; MATN1; LAPTM5; 400748; PUM1; FLJ12650; HPRP8BP; PS1D; 128499; FABP3; TDE2L; LOC284551; LCN7; HCRTR1; PEF; COL16A1; BAI2; FLJ25348; SEMACAP3; PTP4A2 |
| 27095 | TRAPPC3 | 1 | 36271267 | 1p34.3 | gneg 13; gpos 3; | 1.03 | 0.102890815 | 0.053 | -0.302 | 0.304 | 0.554 | PSMB2; FLJ38984; CLSPN; EIF2C4; EIF2C1; EIF2C3; LOC132241; TEKT2; ADPRHL2; COL8A2; TRAPPC3; FLJ10350; TRAP150; FLJ22938; FLJ10647; 387647; MGC4796; LSM10; NOR1; MRPS15; CSF3R |
| 55929 | DMAP1 | 1 | 44348255 | 1p34 | gneg 12; gpos 1; | 1.17 | 0.915174864 | 0.038 | -0.021 | 0.354 | 0.038 | SIAT6; ARTN; IPO13; DPH2L2; ATP6V0B; B4GALT2; MGC45441; SLC6A9; 400751; FLJ40160; DMAP1; PRNPIP; FLJ10597; FLJ22353; 339541; KIF2C; RPS8; VMD2L2; CNK; 343521; LOC149478 |
| 11004 | KIF2C | 1 | 44874643 | 1p34.1 | gneg 12; gpos 1; | 1.38 | 0.186527837 | 0.003 | 0.249 | 0.342 | -0.456 | B4GALT2; MGC45441; SLC6A9; 400751; FLJ40160; DMAP1; PRNPIP; FLJ10597; FLJ22353; 339541; KIF2C; RPS8; VMD2L2; CNK; 343521; LOC149478; PTCH2; EIF2B3; FLJ21156; UROD; ZSWIM5 |
| 2060 | EPS15 | 1 | 51532919 | 1p32 | gneg 6; gpos 7; | 1.43 | 0.332989836 | 0.143 | 0.185 | 0.319 | -0.339 | LOC115548; FLJ14442; FLJ11588; ELAVL4; DMRTA2; FAF1; MGC20533; CDKN2C; 284546; RNF11; EPS15; OSBPL9; NRD1; RAB3B; TLP19; LOC112970; KIAA1340; MADHIP; KIAA1836; ORC1L; FLJ14936 |
| 4898 | NRD1 | 1 | 51966904 | 1p32.2-p32.1 | gneg 8; gpos 5; | 1.26 | 0.625132384 | 0.122 | 0.094 | 0.302 | -0.173 | FLJ11588; ELAVL4; DMRTA2; FAF1; MGC20533; CDKN2C; 284546; RNF11; EPS15; OSBPL9; NRD1; RAB3B; TLP19; LOC112970; KIAA1340; MADHIP; KIAA1836; ORC1L; FLJ14936; KIAA0191; MGC52498 |
| 25950 | RWDD3 | 1 | 95411787 | 1p22.1 | gneg 6; gpos 4; | 1.24 | 0.082423129 | -0.069 | 0.32 | 0.349 | -0.586 | GCLM; ABCA4; PARG1; ABCD3; F3; 400763; MGC45474; CNN3; MGC19780; FLJ31842; RWDD3; PTBP2; DPYD; SNX7; LOC163404; PALMD; AGL; SLC35A3; HIAT1; DKFZp761A078; FLJ10287 |
| 23443 | SLC35A3 | 1 | 100147560 | 1p21 | gneg 3; gpos 7; | 1.5 | 0.277506772 | 0.096 | 0.206 | 0.316 | -0.378 | CNN3; MGC19780; FLJ31842; RWDD3; PTBP2; DPYD; SNX7; LOC163404; PALMD; AGL; SLC35A3; HIAT1; DKFZp761A078; FLJ10287; MGC14816; DBT; RTCD1; CDC14A; GPR88; VCAM1; EXTL2 |
| 3749 | KCNC4 | 1 | 110466018 | 1p21 | gneg 6; gpos 8; | 2.08 | 0.046520868 | 0.004 | 0.361 | 0.315 | -0.662 | GSTM2; GSTM1; GSTM5; GSTM3; EPS8L3; CSF1; AHCYL1; FLJ14743; ALX3; 164153; KCNC4; RBM15; SLC16A4; HBXIP; PROK1; KCNA10; KCNA2; KCNA3; CD53; RIF1; MGC54289 |
| 149018 | not_found | 1 | 149989036 | 1q22 | gneg 3; gpos 3; | 1.64 | 0.407094984 | 0.018 | 0.159 | 0.338 | -0.291 | SPRL2A; LEP1; MCSP; IVL; SPRR4; SPRR1A; SPRR3; SPRR1B; SPRR2B; SPRR2A; 149018; LOR; PGLYRPIalpha; PGLYRPIbeta; S100A9; S100A12; S100A8; S100A15; S100A7; S100A6; S100A5 |
| 114771 | PGLYRPIalpha | 1 | 150083410 | 1q21 | gneg 1; gpos 3; | 2.16 | 0.547770297 | -0.135 | 0.116 | 0.369 | -0.212 | MCSP; IVL; SPRR4; SPRR1A; SPRR3; SPRR1B; SPRR2B; SPRR2A; 149018; LOR; PGLYRPIalpha; PGLYRPIbeta; S100A9; S100A12; S100A8; S100A15; S100A7; S100A6; S100A5; S100A4; 389562 |
| 338324 | S100A15 | 1 | 150202072 | 1q22 | gneg 1; gpos 2; | 1.49 | 0.433309656 | 0.003 | -0.15 | 0.418 | 0.276 | SPRR1B; SPRR2B; SPRR2A; 149018; LOR; PGLYRPIalpha; PGLYRPIbeta; S100A9; S100A12; S100A8; S100A15; S100A7; S100A6; S100A5; S100A4; 389562; S100A3; S100A2; 401922; S100A16; S100A14 |
| 6277 | S100A6 | 1 | 150320148 | 1q21 | gneg 1; gpos 2; | -1.75 | 0.062596466 | 0.177 | -0.34 | 0.411 | 0.624 | SPRR2A; 149018; LOR; PGLYRPIalpha; PGLYRPIbeta; S100A9; S100A12; S100A8; S100A15; S100A7; S100A6; S100A5; S100A4; 389562; S100A3; S100A2; 401922; S100A16; S100A14; S100A13; S100A1 |
| 6275 | S100A4 | 1 | 150329170 | 1q21 | gneg 1; gpos 3; | -3.6 | 0.025078954 | 0.22 | -0.399 | 0.363 | 0.732 | LOR; PGLYRPIalpha; PGLYRPIbeta; S100A9; S100A12; S100A8; S100A15; S100A7; S100A6; S100A5; S100A4; 389562; S100A3; S100A2; 401922; S100A16; S100A14; S100A13; S100A1; DKFZP547E1010; SNAPAP |
| 389562 | not_found | 1 | 150329227 | 1q21 | gneg 1; gpos 4; | -1.24 | 0.030913553 | 0.237 | -0.387 | 0.364 | 0.709 | PGLYRPIalpha; PGLYRPIbeta; S100A9; S100A12; S100A8; S100A15; S100A7; S100A6; S100A5; S100A4; 389562; S100A3; S100A2; 401922; S100A16; S100A14; S100A13; S100A1; DKFZP547E1010; SNAPAP; ILF2 |
| 6274 | S100A3 | 1 | 150332881 | 1q21 | gneg 1; gpos 4; | -2.44 | 0.061491482 | 0.253 | -0.342 | 0.309 | 0.626 | PGLYRPIbeta; S100A9; S100A12; S100A8; S100A15; S100A7; S100A6; S100A5; S100A4; 389562; S100A3; S100A2; 401922; S100A16; S100A14; S100A13; S100A1; DKFZP547E1010; SNAPAP; ILF2; NPR1 |
| 6273 | S100A2 | 1 | 150346659 | 1q21 | gneg 1; gpos 5; | 1.64 | 0.84341297 | 0.165 | -0.038 | 0.305 | 0.07 | S100A9; S100A12; S100A8; S100A15; S100A7; S100A6; S100A5; S100A4; 389562; S100A3; S100A2; 401922; S100A16; S100A14; S100A13; S100A1; DKFZP547E1010; SNAPAP; ILF2; NPR1; FLJ21919 |
| 23381 | EST1B | 1 | 153032089 | 1q21.2 | gneg 10; gpos 5; gvar 1; | 1.31 | 0.587397234 | 0.008 | -0.105 | 0.443 | 0.192 | MAPBPIP; RAB25; LOC92312; LMNA; LOC169834; FLJ12287; KIAA0446; PMF1; BGLAP; PAQR6; EST1B; MGC41903; MGC13102; MGC31963; CCT3; SSTK-IP; CROC4; MEF2D; IQGAP3; 164118; APOA1BP |
| 7203 | CCT3 | 1 | 153091832 | 1q23 | gneg 12; gpos 4; gvar 1; | 1.37 | 0.595784979 | -0.049 | 0.102 | 0.369 | -0.188 | LOC169834; FLJ12287; KIAA0446; PMF1; BGLAP; PAQR6; EST1B; MGC41903; MGC13102; MGC31963; CCT3; SSTK-IP; CROC4; MEF2D; IQGAP3; 164118; APOA1BP; FLJ20249; BRAL1; BCAN; NES |
| 128229 | SSTK-IP | 1 | 153120177 | 1q23.1 | gneg 13; gpos 3; gvar 1; | 4.13 | 0.000487069 | 0.014 | 0.556 | 0.38 | -1.019 | FLJ12287; KIAA0446; PMF1; BGLAP; PAQR6; EST1B; MGC41903; MGC13102; MGC31963; CCT3; SSTK-IP; CROC4; MEF2D; IQGAP3; 164118; APOA1BP; FLJ20249; BRAL1; BCAN; NES; CRABP2 |
| 63923 | TNN | 1 | 171827760 | 1q23-q24 | gneg 3; gpos 3; | 1.12 | 0.348272648 | -0.013 | -0.179 | 0.313 | 0.328 | FLJ10514; MGC2629; SERPINC1; KIAA2025; KIAA0492; HHL; GPR52; MRF2; SIP; MRPS14; TNN; KIAA0040; TNR; COP1; PLAC3; LOC57795; LOC284307; 400796; RGPR; 389316; RASAL2 |
| 60676 | PLAC3 | 1 | 173163963 | 1q23-q25 | gpos 6; | 1.62 | 0.789413024 | 0.046 | 0.052 | 0.306 | -0.095 | KIAA0492; HHL; GPR52; MRF2; SIP; MRPS14; TNN; KIAA0040; TNR; COP1; PLAC3; LOC57795; LOC284307; 400796; RGPR; 389316; RASAL2; DKFZP564J047; FLJ10244; ANGPTL1; FAM20B |
| 9641 | IKBKE | 1 | 203032190 | 1q32.1 | gneg 8; gpos 1; | -1.02 | 0.160184733 | -0.097 | -0.263 | 0.385 | 0.483 | ELK4; Prostein; NUCKS; RAB7L1; LOC255812; SLC41A1; FLJ32569; SLC26A9; AVPR1B; CTSE; IKBKE; RASSF5; LGTN; DYRK3; MAPKAPK2; IL10; IL19; IL20; IL24; TOSO; PIGR |
| 9261 | MAPKAPK2 | 1 | 203246683 | 1q32 | gneg 8; gpos 1; | -1.03 | 0.085726122 | 0.071 | -0.317 | 0.433 | 0.581 | LOC255812; SLC41A1; FLJ32569; SLC26A9; AVPR1B; CTSE; IKBKE; RASSF5; LGTN; DYRK3; MAPKAPK2; IL10; IL19; IL20; IL24; TOSO; PIGR; 441471; FKSG87; SARG; PFKFB2 |
| 29949 | IL19 | 1 | 203360609 | 1q32.2 | gneg 6; gpos 1; | 1.26 | 0.253046716 | 0.113 | -0.217 | 0.336 | 0.397 | FLJ32569; SLC26A9; AVPR1B; CTSE; IKBKE; RASSF5; LGTN; DYRK3; MAPKAPK2; IL10; IL19; IL20; IL24; TOSO; PIGR; 441471; FKSG87; SARG; PFKFB2; C4BPB; C4BPA |
| 50604 | IL20 | 1 | 203427548 | 1q32 | gneg 5; gpos 1; | 1.33 | 0.566595406 | -0.007 | -0.11 | 0.322 | 0.203 | SLC26A9; AVPR1B; CTSE; IKBKE; RASSF5; LGTN; DYRK3; MAPKAPK2; IL10; IL19; IL20; IL24; TOSO; PIGR; 441471; FKSG87; SARG; PFKFB2; C4BPB; C4BPA; DAF |
| 148304 | FLJ25078 | 1 | 206344056 | 1q32.2 | gneg 4; gpos 3; | 1.89 | 0.018892929 | -0.135 | 0.415 | 0.367 | -0.761 | MCP; 388732; CD34; 442769; CAMK1G; LAMB3; DKFZp762K222; G0S2; HSD11B1; T3JAM; FLJ25078; IRF6; MGC29875; SYT14; FLJ10724; KCNH1; FLJ10876; TRAF5; MGC14801; C1orf36; SLC30A1 |
| 79805 | FLJ12505 | 1 | 209512371 | 1q32.3 | gneg 10; gpos 7; | 3.33 | 0.247569322 | 0.025 | 0.219 | 0.308 | -0.402 | PPP2R5A; FLJ10874; SPUF; ATF3; FLJ32796; SNFT; DC8; 149643; FLVCR; MESDC2; FLJ12505; LOC90806; RPS6KC1; PROX1; SMYD2; PTPN14; CENPF; KCNK2; KCTD3; USH2A; ESRRG |
| 84886 | FLJ14525 | 1 | 227279600 | 1q42.13-q43 | gneg 2; gpos 8; | -1.4 | 0.01458324 | 0.059 | -0.429 | 0.35 | 0.786 | ACTA1; NUP133; ABCB10; TAF5L; 401491; GALNT2; PGBD5; COG2; AGT; CAPN9; FLJ14525; TTC13; ARV1; MGC15887; C20orf82; DKFZp547B1713; GNPAT; EXOC8; DKFZP547N043; EGLN1; TSNAX |
| 64801 | ARV1 | 1 | 227421557 | 1q42.2 | gneg 1; gpos 9; | 1.34 | 0.326752385 | 0.243 | 0.187 | 0.345 | -0.343 | ABCB10; TAF5L; 401491; GALNT2; PGBD5; COG2; AGT; CAPN9; FLJ14525; TTC13; ARV1; MGC15887; C20orf82; DKFZp547B1713; GNPAT; EXOC8; DKFZP547N043; EGLN1; TSNAX; DISC1; SIPA1L2 |
| 375061 | MGC15887 | 1 | 227461667 | 1q42.2 | gneg 1; gpos 10; | -4.05 | 0.000846792 | 0.191 | -0.54 | 0.359 | 0.99 | TAF5L; 401491; GALNT2; PGBD5; COG2; AGT; CAPN9; FLJ14525; TTC13; ARV1; MGC15887; C20orf82; DKFZp547B1713; GNPAT; EXOC8; DKFZP547N043; EGLN1; TSNAX; DISC1; SIPA1L2; MGC13186 |
| 128061 | DKFZp547B1713 | 1 | 227666245 | 1q42.2 | gpos 11; | 1.21 | 0.260636986 | 0.165 | 0.213 | 0.31 | -0.391 | GALNT2; PGBD5; COG2; AGT; CAPN9; FLJ14525; TTC13; ARV1; MGC15887; C20orf82; DKFZp547B1713; GNPAT; EXOC8; DKFZP547N043; EGLN1; TSNAX; DISC1; SIPA1L2; MGC13186; FLJ11383; KIAA1804 |
| 149371 | EXOC8 | 1 | 227775216 | 1q42.2 | gneg 1; gpos 10; | 1.32 | 0.086851268 | -0.036 | 0.316 | 0.314 | -0.579 | COG2; AGT; CAPN9; FLJ14525; TTC13; ARV1; MGC15887; C20orf82; DKFZp547B1713; GNPAT; EXOC8; DKFZP547N043; EGLN1; TSNAX; DISC1; SIPA1L2; MGC13186; FLJ11383; KIAA1804; KCNK1; SLC35F3 |
| 83932 | DKFZP547N043 | 1 | 227780465 | 1q42.12-q43 | gneg 2; gpos 9; | 1.38 | 0.209356476 | 0.137 | 0.237 | 0.334 | -0.434 | AGT; CAPN9; FLJ14525; TTC13; ARV1; MGC15887; C20orf82; DKFZp547B1713; GNPAT; EXOC8; DKFZP547N043; EGLN1; TSNAX; DISC1; SIPA1L2; MGC13186; FLJ11383; KIAA1804; KCNK1; SLC35F3; TARBP1 |
| 54583 | EGLN1 | 1 | 227808165 | 1q42.1 | gneg 3; gpos 9; | 1.13 | 0.911301067 | -0.137 | -0.022 | 0.397 | 0.04 | CAPN9; FLJ14525; TTC13; ARV1; MGC15887; C20orf82; DKFZp547B1713; GNPAT; EXOC8; DKFZP547N043; EGLN1; TSNAX; DISC1; SIPA1L2; MGC13186; FLJ11383; KIAA1804; KCNK1; SLC35F3; TARBP1; IRF2BP2 |
| 7257 | TSNAX | 1 | 227971133 | 1q42.1 | gneg 3; gpos 9; | 1.91 | 0.01184154 | 0.101 | 0.439 | 0.336 | -0.805 | FLJ14525; TTC13; ARV1; MGC15887; C20orf82; DKFZp547B1713; GNPAT; EXOC8; DKFZP547N043; EGLN1; TSNAX; DISC1; SIPA1L2; MGC13186; FLJ11383; KIAA1804; KCNK1; SLC35F3; TARBP1; IRF2BP2; 400961 |
| 84451 | KIAA1804 | 1 | 229770248 | 1q42 | gneg 5; gpos 5; | 7.16 | 0.036709061 | -0.16 | 0.376 | 0.373 | -0.69 | DKFZp547B1713; GNPAT; EXOC8; DKFZP547N043; EGLN1; TSNAX; DISC1; SIPA1L2; MGC13186; FLJ11383; KIAA1804; KCNK1; SLC35F3; TARBP1; IRF2BP2; 400961; 284527; KIAA0117; TOMM20; 387791; RBP1L1 |
| 3775 | KCNK1 | 1 | 230056484 | 1q42-q43 | gneg 5; gpos 5; | 2.87 | 0.025436522 | -0.096 | 0.398 | 0.352 | -0.73 | GNPAT; EXOC8; DKFZP547N043; EGLN1; TSNAX; DISC1; SIPA1L2; MGC13186; FLJ11383; KIAA1804; KCNK1; SLC35F3; TARBP1; IRF2BP2; 400961; 284527; KIAA0117; TOMM20; 387791; RBP1L1; GGPS1 |
| 81469 | OR2G3 | 1 | 244094928 | 1 | gneg 18; | 2.43 | 0.950809515 | 0.007 | -0.012 | 0.331 | 0.022 | LOC90333; MGC12466; FLJ12606; ZNF124; VN1R5; ZNF496; CIAS1; 127623; OR2C3; LOC148823; OR2G3; 343170; OR1C1; BIA2; 343171; 343172; MGC40047; 391191; 127059; OR2M4; 391195 |
| 25893 | BIA2 | 1 | 244346541 | 1q44 | gneg 18; | -1.71 | 0.031012867 | 0.095 | -0.387 | 0.373 | 0.709 | ZNF124; VN1R5; ZNF496; CIAS1; 127623; OR2C3; LOC148823; OR2G3; 343170; OR1C1; BIA2; 343171; 343172; MGC40047; 391191; 127059; OR2M4; 391195; 127066; 127074; OR2T1 |
| 3032 | HADHB | 2 | 26379471 | 2p23 | gneg 4; gpos 8; | 1.05 | 0.032005672 | 0.069 | -0.385 | 0.314 | 0.705 | KIAA0953; POMC; DNMT3A; DTNB; ASXL2; KIF3C; RAB10; 389749; LOC150946; HADHA; HADHB; GPR113; MGC16372; OTOF; 130106; KCNK3; C2orf18; CENPA; DPYSL5; MAPRE3; FLJ20254 |
| 388565 | not_found | 2 | 64691996 | 11q12.1 | gneg 6; gpos 6; | 1.51 | 0.554251869 | -0.096 | -0.114 | 0.316 | 0.209 | KIAA0903; OTX1; LOC51057; MDH1; 388955; UGP2; VPS54; PELI1; HSPC159; FLJ20080; 388565; 400958; SLC1A4; 200731; KIAA0582; RAB1A; ACTR2; SPRED2; MEIS1; 219537; ETAA16 |
| 5861 | RAB1A | 2 | 65225639 | 2p14 | gneg 7; gpos 6; | 1.11 | 0.680861206 | 0.076 | -0.08 | 0.301 | 0.146 | UGP2; VPS54; PELI1; HSPC159; FLJ20080; 388565; 400958; SLC1A4; 200731; KIAA0582; RAB1A; ACTR2; SPRED2; MEIS1; 219537; ETAA16; C1D; LOC116143; LOC56902; PPP3R1; DKFZP566K1924 |
| 51255 | LOC51255 | 2 | 85734514 | 2p11.2 | gneg 13; gpos 2; | -1.08 | 0.079521796 | 0.076 | -0.323 | 0.358 | 0.592 | C14orf125; HLA-DRB4; FLJ20296; FLJ21977; CAPG; LOC284948; MAT2A; GGCX; VAMP8; VAMP5; LOC51255; FLJ90024; USP39; SFTPB; GNLY; HATH6; SIAT9; POLR1A; FLJ20758; IMMT; MRPL35 |
| 10713 | USP39 | 2 | 85754966 | 2p11.2 | gneg 14; gpos 2; | 1.51 | 0.002067306 | -0.1 | 0.511 | 0.332 | -0.938 | FLJ20296; FLJ21977; CAPG; LOC284948; MAT2A; GGCX; VAMP8; VAMP5; LOC51255; FLJ90024; USP39; SFTPB; GNLY; HATH6; SIAT9; POLR1A; FLJ20758; IMMT; MRPL35; FLJ13110; MYR8 |
| 30061 | SLC40A1 | 2 | 190250823 | 2q32 | gneg 5; gpos 6; | -82.8 | 0.000872962 | 0.248 | -0.539 | 0.337 | 0.989 | ITGAV; KIAA1946; ZSWIM2; CALCRL; TFPI; GULP1; DIRC1; COL3A1; COL5A2; FLJ12519; SLC40A1; NS3TP1; NUP160; OSGEPL1; ORMDL1; PMS1; GDF8; MGC13057; HIBCH; INPP1; FLJ20160 |
| 23279 | NUP160 | 2 | 190438139 | 11p11.12 | gneg 4; gpos 7; | 2.08 | 5.50E-05 | -0.151 | 0.606 | 0.3 | -1.111 | ZSWIM2; CALCRL; TFPI; GULP1; DIRC1; COL3A1; COL5A2; FLJ12519; SLC40A1; NS3TP1; NUP160; OSGEPL1; ORMDL1; PMS1; GDF8; MGC13057; HIBCH; INPP1; FLJ20160; KIAA0564; 401026 |
| 79840 | FLJ12610 | 2 | 219765552 | 2q36.1 | gneg 5; gpos 11; | 1.41 | 0.575921499 | -0.032 | 0.108 | 0.482 | -0.198 | WNT6; WNT10A; LOC285401; CDK5R2; LOC151300; HSRNAFEV; CRYBA2; DKFZp434O0527; MGC35338; IHH; FLJ12610; SLC23A3; CGI-57; C2orf17; LOC130617; ABCB6; FLJ22169; FLJ10415; GLB1L; STK16; TUBA1 |
| 151295 | SLC23A3 | 2 | 219851694 | 2q36.1 | gneg 4; gpos 12; | -1.1 | 0.036788414 | -0.049 | -0.376 | 0.444 | 0.69 | WNT10A; LOC285401; CDK5R2; LOC151300; HSRNAFEV; CRYBA2; DKFZp434O0527; MGC35338; IHH; FLJ12610; SLC23A3; CGI-57; C2orf17; LOC130617; ABCB6; FLJ22169; FLJ10415; GLB1L; STK16; TUBA1; TUBA4 |
| 27013 | CGI-57 | 2 | 219862123 | 2q36.1 | gneg 3; gpos 12; | 1.14 | 0.49746666 | 0.073 | -0.131 | 0.556 | 0.239 | LOC285401; CDK5R2; LOC151300; HSRNAFEV; CRYBA2; DKFZp434O0527; MGC35338; IHH; FLJ12610; SLC23A3; CGI-57; C2orf17; LOC130617; ABCB6; FLJ22169; FLJ10415; GLB1L; STK16; TUBA1; TUBA4; DNAJB2 |
| 130617 | LOC130617 | 2 | 219897042 | 2q36.1 | gneg 1; gpos 13; | -1.52 | 0.004094771 | -0.04 | -0.486 | 0.563 | 0.891 | LOC151300; HSRNAFEV; CRYBA2; DKFZp434O0527; MGC35338; IHH; FLJ12610; SLC23A3; CGI-57; C2orf17; LOC130617; ABCB6; FLJ22169; FLJ10415; GLB1L; STK16; TUBA1; TUBA4; DNAJB2; PTPRN; DNPEP |
| 10058 | ABCB6 | 2 | 219899998 | 2q36 | gneg 1; gpos 13; | 1.45 | 0.466492012 | 0.163 | 0.14 | 0.546 | -0.256 | HSRNAFEV; CRYBA2; DKFZp434O0527; MGC35338; IHH; FLJ12610; SLC23A3; CGI-57; C2orf17; LOC130617; ABCB6; FLJ22169; FLJ10415; GLB1L; STK16; TUBA1; TUBA4; DNAJB2; PTPRN; DNPEP; DES |
| 79065 | FLJ22169 | 2 | 219909606 | 2q36.1 | gneg 1; gpos 14; | 1.03 | 0.090520773 | 0.157 | -0.313 | 0.588 | 0.573 | CRYBA2; DKFZp434O0527; MGC35338; IHH; FLJ12610; SLC23A3; CGI-57; C2orf17; LOC130617; ABCB6; FLJ22169; FLJ10415; GLB1L; STK16; TUBA1; TUBA4; DNAJB2; PTPRN; DNPEP; DES; APEG1 |
| 55139 | FLJ10415 | 2 | 219920046 | 2q36.1 | gneg 1; gpos 15; | 1.13 | 0.952016887 | 0.149 | -0.012 | 0.519 | 0.021 | DKFZp434O0527; MGC35338; IHH; FLJ12610; SLC23A3; CGI-57; C2orf17; LOC130617; ABCB6; FLJ22169; FLJ10415; GLB1L; STK16; TUBA1; TUBA4; DNAJB2; PTPRN; DNPEP; DES; APEG1; GMPPA |
| 7277 | TUBA1 | 2 | 219940505 | 2q36.1 | gneg 1; gpos 16; | 4.99 | 0.008614336 | 0.242 | 0.454 | 0.582 | -0.833 | FLJ12610; SLC23A3; CGI-57; C2orf17; LOC130617; ABCB6; FLJ22169; FLJ10415; GLB1L; STK16; TUBA1; TUBA4; DNAJB2; PTPRN; DNPEP; DES; APEG1; GMPPA; ACCN4; CHPF; 130612 |
| 3300 | DNAJB2 | 2 | 219969602 | 2q32-q34 | gneg 1; gpos 15; | -1.54 | 8.64E-06 | -0.027 | -0.637 | 0.527 | 1.167 | CGI-57; C2orf17; LOC130617; ABCB6; FLJ22169; FLJ10415; GLB1L; STK16; TUBA1; TUBA4; DNAJB2; PTPRN; DNPEP; DES; APEG1; GMPPA; ACCN4; CHPF; 130612; KIAA0657; INHA |
| 5798 | PTPRN | 2 | 219979851 | 2q35-q36.1 | gneg 1; gpos 15; | 1.6 | 0.473014865 | -0.054 | -0.138 | 0.335 | 0.253 | C2orf17; LOC130617; ABCB6; FLJ22169; FLJ10415; GLB1L; STK16; TUBA1; TUBA4; DNAJB2; PTPRN; DNPEP; DES; APEG1; GMPPA; ACCN4; CHPF; 130612; KIAA0657; INHA; STK11IP |
| 23549 | DNPEP | 2 | 220064421 | 2q36.1 | gneg 1; gpos 14; | 1.33 | 0.953737522 | 0.245 | -0.011 | 0.376 | 0.021 | LOC130617; ABCB6; FLJ22169; FLJ10415; GLB1L; STK16; TUBA1; TUBA4; DNAJB2; PTPRN; DNPEP; DES; APEG1; GMPPA; ACCN4; CHPF; 130612; KIAA0657; INHA; STK11IP; SLC4A3 |
| 1674 | DES | 2 | 220108609 | 2q35 | gneg 1; gpos 14; | -4.26 | 0.018691981 | -0.116 | -0.416 | 0.31 | 0.762 | ABCB6; FLJ22169; FLJ10415; GLB1L; STK16; TUBA1; TUBA4; DNAJB2; PTPRN; DNPEP; DES; APEG1; GMPPA; ACCN4; CHPF; 130612; KIAA0657; INHA; STK11IP; SLC4A3; EPHA4 |
| 10290 | APEG1 | 2 | 220133900 | 2q36.1 | gneg 2; gpos 14; | -1.37 | 0.000266289 | -0.076 | -0.572 | 0.407 | 1.048 | FLJ22169; FLJ10415; GLB1L; STK16; TUBA1; TUBA4; DNAJB2; PTPRN; DNPEP; DES; APEG1; GMPPA; ACCN4; CHPF; 130612; KIAA0657; INHA; STK11IP; SLC4A3; EPHA4; PAX3 |
| 55515 | ACCN4 | 2 | 220204556 | 2q36.1 | gneg 2; gpos 14; | 1.28 | 0.2639588 | 0.155 | -0.212 | 0.372 | 0.389 | GLB1L; STK16; TUBA1; TUBA4; DNAJB2; PTPRN; DNPEP; DES; APEG1; GMPPA; ACCN4; CHPF; 130612; KIAA0657; INHA; STK11IP; SLC4A3; EPHA4; PAX3; FLJ32447; SGPP2 |
| 23363 | KIAA0657 | 2 | 220246823 | 2q36.1 | gneg 3; gpos 12; | 1.36 | 0.526210568 | 0.2 | -0.122 | 0.45 | 0.224 | TUBA4; DNAJB2; PTPRN; DNPEP; DES; APEG1; GMPPA; ACCN4; CHPF; 130612; KIAA0657; INHA; STK11IP; SLC4A3; EPHA4; PAX3; FLJ32447; SGPP2; FARSLB; DGAT2L1; FACL3 |
| 3623 | INHA | 2 | 220262458 | 2q33-q36 | gneg 3; gpos 12; | 1.17 | 0.346492517 | 0.074 | -0.18 | 0.382 | 0.33 | DNAJB2; PTPRN; DNPEP; DES; APEG1; GMPPA; ACCN4; CHPF; 130612; KIAA0657; INHA; STK11IP; SLC4A3; EPHA4; PAX3; FLJ32447; SGPP2; FARSLB; DGAT2L1; FACL3; KCNE4 |
| 84522 | GL009 | 3 | 9907271 | 3p25.2 | gneg 15; gpos 1; | 1.26 | 0.846259374 | -0.06 | -0.038 | 0.35 | 0.069 | FLJ22405; LOC151835; BRPF1; OGG1; CAMK1; TADA3L; ARPC4; TTLL3; MGC29784; CIDE-3; GL009; IL17RE; IL17RC; CRELD1; LOC55831; 401052; FANCD2; MGC40179; MDS027; VHL; IRAK2 |
| 2177 | FANCD2 | 3 | 10043127 | 3p26 | gneg 13; gpos 1; | 1.24 | 0.710157632 | -0.15 | -0.072 | 0.304 | 0.132 | ARPC4; TTLL3; MGC29784; CIDE-3; GL009; IL17RE; IL17RC; CRELD1; LOC55831; 401052; FANCD2; MGC40179; MDS027; VHL; IRAK2; KIAA0218; GHRL; SEC13L1; ATP2B2; SLC6A11; SLC6A1 |
| 112616 | CKLFSF7 | 3 | 32408166 | 3p22.3 | gneg 5; gpos 11; | 1.3 | 0.32328131 | -0.029 | 0.188 | 0.338 | -0.345 | MGC61571; AZ2; LOC152098; FLJ33651; RBMS3; TGFBR2; SIMP; OSBPL10; KIAA0089; CKLFSF8; CKLFSF7; CKLFSF6; DNCLI1; CNOT10; 389102; 131405; CCR4; GLB1; CRTAP; FBXL2; UBP1 |
| 54918 | CKLFSF6 | 3 | 32497807 | 3p22.3 | gneg 4; gpos 12; | 1.12 | 0.610108442 | -0.027 | 0.098 | 0.301 | -0.18 | AZ2; LOC152098; FLJ33651; RBMS3; TGFBR2; SIMP; OSBPL10; KIAA0089; CKLFSF8; CKLFSF7; CKLFSF6; DNCLI1; CNOT10; 389102; 131405; CCR4; GLB1; CRTAP; FBXL2; UBP1; PDCD6IP |
| 8309 | ACOX2 | 3 | 58465903 | 3p14.3 | gneg 5; gpos 11; | -1.06 | 0.980743143 | 0.144 | 0.005 | 0.319 | -0.009 | ARF4; FLJ34969; SLMAP; FLNB; DNASE1L3; ABHD6; RPP14; PXK; PDHB; KCTD6; ACOX2; TU3A; FAM3D; FLJ42117; 339902; FHIT; PTPRG; AOF1; HT021; FEZL; CADPS |
| 57650 | KIAA1524 | 3 | 109752094 | 3q13.13 | gneg 5; gpos 8; | 2.13 | 0.002564516 | 0.136 | 0.504 | 0.313 | -0.923 | CBLB; 344595; NYD-SP17; BBX; LOC151658; LOC285205; CD47; ESRRBL1; HHLA2; KIAA1000; KIAA1524; DZIP3; RETNLB; TRIM; GUCA1C; MORC; LOC151871; DPPA4; FLJ38508; 401082; 151760 |
| 23434 | GR6 | 3 | 129773541 | 3q21 | gneg 11; gpos 1; | 1.06 | 0.615236555 | -0.076 | -0.097 | 0.314 | 0.178 | TPRA40; MCM2; PODLX2; ABTB1; MGLL; SEC61A1; RUVBL1; SELB; DNAJB8; GATA2; GR6; RPN1; RAB7; 389147; ACAD9; KIAA1281; KIAA1257; 401087; GP9; LOC339122; KIAA1160 |
| 23129 | PLXND1 | 3 | 130756715 | 3q21.3 | gneg 8; gpos 6; | -2.21 | 0.033223898 | -0.039 | -0.382 | 0.375 | 0.701 | ZNF9; COPG; PLXNB2; DC12; H1FX; LOC339942; MBD4; WDR10; RHO; H1FOO; PLXND1; KIAA0779; TRH; 387746; FLJ35880; LOC131873; PIK3R4; ATP2C1; HT001; NEK11; LOC152195 |
| 5217 | PFN2 | 3 | 151165391 | 3q25.1-q25.2 | gneg 10; gpos 3; | 1.25 | 0.744427401 | 0.072 | 0.063 | 0.321 | -0.116 | SMARCA3; LOC284825; HPS3; CP; LOC116441; TM4SF1; TM4SF4; LOC51122; 389161; RNF13; PFN2; KIAA0669; SERP1; KIAA1753; eIF2A; SELT; MGC39662; SIAH2; USH3A; TRALPUSH; H963 |
| 51714 | SELT | 3 | 151803763 | 3q25.1 | gneg 11; gpos 2; | 2 | 0.060240676 | 0.286 | 0.343 | 0.336 | -0.629 | TM4SF1; TM4SF4; LOC51122; 389161; RNF13; PFN2; KIAA0669; SERP1; KIAA1753; eIF2A; SELT; MGC39662; SIAH2; USH3A; TRALPUSH; H963; GPR105; GPR87; GPR86; P2RY12; FLJ25972 |
| 54819 | FLJ20094 | 5 | 132360577 | 5q31.1 | gneg 11; | 1.92 | 0.060669418 | 0.209 | 0.343 | 0.328 | -0.628 | IL13; IL4; KIF3A; SEPT8; LOC134548; APXL2; GDF9; QP-C; LEAP-2; AF5Q31; FLJ20094; HSPA4; KIAA1061; HTGN29; VDAC1; TCF7; SKP1A; 347442; PPP2CA; CDKL3; UBE2B |
| 3308 | HSPA4 | 5 | 132415560 | 5q31.1-q31.2 | gneg 11; gpos 1; | 2.17 | 0.001102461 | -0.168 | 0.532 | 0.311 | -0.976 | IL4; KIF3A; SEPT8; LOC134548; APXL2; GDF9; QP-C; LEAP-2; AF5Q31; FLJ20094; HSPA4; KIAA1061; HTGN29; VDAC1; TCF7; SKP1A; 347442; PPP2CA; CDKL3; UBE2B; MGC13017 |
| 5515 | PPP2CA | 5 | 133560319 | 5q23-q31 | gneg 7; gpos 5; | 1.27 | 0.060319672 | 0.166 | 0.343 | 0.32 | -0.629 | LEAP-2; AF5Q31; FLJ20094; HSPA4; KIAA1061; HTGN29; VDAC1; TCF7; SKP1A; 347442; PPP2CA; CDKL3; UBE2B; MGC13017; FLJ12270; PHF15; SARA2; 387643; CAMLG; DDX46; FLJ37562 |
| 202051 | LOC202051 | 5 | 138760381 | 5q31.3 | gneg 12; | 2.42 | 0.001555404 | 0.107 | 0.521 | 0.304 | -0.955 | EGR1; ETF1; HSPA9B; CTNNA1; 401210; SIL1; MATR3; PAIP2; SLC23A1; PACAP; LOC202051; MGC29463; LOC340061; UBE2D2; HSPC195; DKFZp761B0514; NRG2; PURA; ORF1-FL49; PFDN1; DTR |
| 83884 | SLC25A2 | 5 | 140662379 | 5q31 | gneg 3; | 1.47 | 0.674451746 | -0.143 | 0.081 | 0.314 | -0.149 | PCDHB7; PCDHB8; PCDHB16; PCDHB9; PCDHB10; PCDHB11; PCDHB12; PCDHB13; PCDHB14; PCDHB15; SLC25A2; TAF7; PCDHGA1; PCDHGA2; PCDHGA3; DIAPH1; HDAC3; C5orf16; FLJ00007; ARAP3; PCDH1 |
| 51164 | DCTN4 | 5 | 150068502 | 5q31-q32 | gneg 12; gpos 1; | -1.25 | 0.003312919 | -0.028 | -0.494 | 0.303 | 0.906 | SLC6A7; CAMK2A; 340075; TCOF1; CD74; RPS14; NDST1; SYNPO; MYOZ3; FLJ10290; DCTN4; NID67; LRG47; ZNF300; LOC134466; GPX3; TNIP1; ANXA6; DKFZP434C171; GM2A; SLC36A3 |
| 91975 | ZNF300 | 5 | 150254156 | 5q33.1 | gneg 12; gpos 1; | 1.94 | 0.266341333 | -0.15 | 0.211 | 0.3 | -0.387 | TCOF1; CD74; RPS14; NDST1; SYNPO; MYOZ3; FLJ10290; DCTN4; NID67; LRG47; ZNF300; LOC134466; GPX3; TNIP1; ANXA6; DKFZP434C171; GM2A; SLC36A3; SLC36A2; SLC36A1; FAT2 |
| 2878 | GPX3 | 5 | 150380111 | 5q23 | gneg 12; gpos 1; | 1.18 | 0.642238879 | 0.034 | -0.09 | 0.32 | 0.165 | RPS14; NDST1; SYNPO; MYOZ3; FLJ10290; DCTN4; NID67; LRG47; ZNF300; LOC134466; GPX3; TNIP1; ANXA6; DKFZP434C171; GM2A; SLC36A3; SLC36A2; SLC36A1; FAT2; SPARC; ATOX1 |
| 10318 | TNIP1 | 5 | 150389709 | 5q32-q33.1 | gneg 13; gpos 1; | -1.19 | 0.027865943 | 0.073 | -0.393 | 0.31 | 0.721 | NDST1; SYNPO; MYOZ3; FLJ10290; DCTN4; NID67; LRG47; ZNF300; LOC134466; GPX3; TNIP1; ANXA6; DKFZP434C171; GM2A; SLC36A3; SLC36A2; SLC36A1; FAT2; SPARC; ATOX1; G3BP |
| 309 | ANXA6 | 5 | 150460862 | 5q32-q34 | gneg 12; gpos 2; | -2.13 | 0.000149866 | 0.055 | -0.585 | 0.344 | 1.073 | SYNPO; MYOZ3; FLJ10290; DCTN4; NID67; LRG47; ZNF300; LOC134466; GPX3; TNIP1; ANXA6; DKFZP434C171; GM2A; SLC36A3; SLC36A2; SLC36A1; FAT2; SPARC; ATOX1; G3BP; GLRA1 |
| 3006 | HIST1H1C | 6 | 26163946 | 6p21.3 | gneg 1; | -1 | 0.588964651 | 0.298 | -0.104 | 0.401 | 0.191 | SLC17A2; TRIM38; HIST1H1A; HIST1H3A; HIST1H4A; HIST1H4B; HIST1H3B; HIST1H2AB; HIST1H2BB; HIST1H3C; HIST1H1C; HFE; HIST1H4C; HIST1H1T; HIST1H2BC; HIST1H2AC; LOC286144; HIST1H1E; HIST1H2BD; HIST1H2BE; HIST1H4D |
| 8364 | HIST1H4C | 6 | 26212154 | 6p21.3 | gneg 1; | 1.13 | 0.177331918 | 0.377 | 0.254 | 0.416 | -0.465 | HIST1H1A; HIST1H3A; HIST1H4A; HIST1H4B; HIST1H3B; HIST1H2AB; HIST1H2BB; HIST1H3C; HIST1H1C; HFE; HIST1H4C; HIST1H1T; HIST1H2BC; HIST1H2AC; LOC286144; HIST1H1E; HIST1H2BD; HIST1H2BE; HIST1H4D; HIST1H3D; HIST1H2AD |
| 8347 | HIST1H2BC | 6 | 26231673 | 6p21.3 | gneg 1; | 1.05 | 0.298899852 | 0.228 | -0.198 | 0.512 | 0.362 | HIST1H4A; HIST1H4B; HIST1H3B; HIST1H2AB; HIST1H2BB; HIST1H3C; HIST1H1C; HFE; HIST1H4C; HIST1H1T; HIST1H2BC; HIST1H2AC; LOC286144; HIST1H1E; HIST1H2BD; HIST1H2BE; HIST1H4D; HIST1H3D; HIST1H2AD; HIST1H2BF; HIST1H4E |
| 3008 | HIST1H1E | 6 | 26264537 | 6p21.3 | gneg 1; | 1.88 | 0.400936324 | 0.293 | 0.161 | 0.509 | -0.295 | HIST1H2AB; HIST1H2BB; HIST1H3C; HIST1H1C; HFE; HIST1H4C; HIST1H1T; HIST1H2BC; HIST1H2AC; LOC286144; HIST1H1E; HIST1H2BD; HIST1H2BE; HIST1H4D; HIST1H3D; HIST1H2AD; HIST1H2BF; HIST1H4E; HIST1H2BG; HIST1H2AE; HIST1H3E |
| 8344 | HIST1H2BE | 6 | 26292002 | 6p21.3 | gneg 1; | -1.19 | 0.092722891 | 0.357 | -0.311 | 0.423 | 0.57 | HIST1H3C; HIST1H1C; HFE; HIST1H4C; HIST1H1T; HIST1H2BC; HIST1H2AC; LOC286144; HIST1H1E; HIST1H2BD; HIST1H2BE; HIST1H4D; HIST1H3D; HIST1H2AD; HIST1H2BF; HIST1H4E; HIST1H2BG; HIST1H2AE; HIST1H3E; HIST1H1D; HIST1H4F |
| 8360 | HIST1H4D | 6 | 26296916 | 6p21.3 | gneg 1; | 1.19 | 0.570057963 | 0.411 | -0.11 | 0.475 | 0.201 | HIST1H1C; HFE; HIST1H4C; HIST1H1T; HIST1H2BC; HIST1H2AC; LOC286144; HIST1H1E; HIST1H2BD; HIST1H2BE; HIST1H4D; HIST1H3D; HIST1H2AD; HIST1H2BF; HIST1H4E; HIST1H2BG; HIST1H2AE; HIST1H3E; HIST1H1D; HIST1H4F; HIST1H4G |
| 8351 | HIST1H3D | 6 | 26304990 | 6p21.3 | gneg 1; gpos 1; | 2.31 | 0.342228674 | 0.232 | 0.181 | 0.531 | -0.332 | HFE; HIST1H4C; HIST1H1T; HIST1H2BC; HIST1H2AC; LOC286144; HIST1H1E; HIST1H2BD; HIST1H2BE; HIST1H4D; HIST1H3D; HIST1H2AD; HIST1H2BF; HIST1H4E; HIST1H2BG; HIST1H2AE; HIST1H3E; HIST1H1D; HIST1H4F; HIST1H4G; HIST1H3F |
| 3013 | HIST1H2AD | 6 | 26306990 | 6p21.3 | gneg 1; gpos 1; | 1.34 | 0.596780202 | 0.214 | -0.102 | 0.548 | 0.187 | HIST1H4C; HIST1H1T; HIST1H2BC; HIST1H2AC; LOC286144; HIST1H1E; HIST1H2BD; HIST1H2BE; HIST1H4D; HIST1H3D; HIST1H2AD; HIST1H2BF; HIST1H4E; HIST1H2BG; HIST1H2AE; HIST1H3E; HIST1H1D; HIST1H4F; HIST1H4G; HIST1H3F; HIST1H2BH |
| 8343 | HIST1H2BF | 6 | 26307765 | 6p21.3 | gneg 1; gpos 1; | 2.86 | 0.935829526 | -0.141 | 0.016 | 0.318 | -0.029 | HIST1H1T; HIST1H2BC; HIST1H2AC; LOC286144; HIST1H1E; HIST1H2BD; HIST1H2BE; HIST1H4D; HIST1H3D; HIST1H2AD; HIST1H2BF; HIST1H4E; HIST1H2BG; HIST1H2AE; HIST1H3E; HIST1H1D; HIST1H4F; HIST1H4G; HIST1H3F; HIST1H2BH; HIST1H3G |
| 8339 | HIST1H2BG | 6 | 26324406 | 6p21.3 | gneg 1; gpos 1; | 1.62 | 0.913645588 | 0.231 | -0.021 | 0.431 | 0.039 | HIST1H2AC; LOC286144; HIST1H1E; HIST1H2BD; HIST1H2BE; HIST1H4D; HIST1H3D; HIST1H2AD; HIST1H2BF; HIST1H4E; HIST1H2BG; HIST1H2AE; HIST1H3E; HIST1H1D; HIST1H4F; HIST1H4G; HIST1H3F; HIST1H2BH; HIST1H3G; HIST1H2BI; HIST1H4H |
| 3012 | HIST1H2AE | 6 | 26325126 | 6p22.2-p21.1 | gneg 1; gpos 2; | 1.45 | 0.988982508 | 0.194 | -0.003 | 0.354 | 0.005 | LOC286144; HIST1H1E; HIST1H2BD; HIST1H2BE; HIST1H4D; HIST1H3D; HIST1H2AD; HIST1H2BF; HIST1H4E; HIST1H2BG; HIST1H2AE; HIST1H3E; HIST1H1D; HIST1H4F; HIST1H4G; HIST1H3F; HIST1H2BH; HIST1H3G; HIST1H2BI; HIST1H4H; BTN3A2 |
| 8353 | HIST1H3E | 6 | 26333361 | 6p21.3 | gpos 3; | 1.41 | 0.766682166 | 0.335 | -0.057 | 0.389 | 0.105 | HIST1H1E; HIST1H2BD; HIST1H2BE; HIST1H4D; HIST1H3D; HIST1H2AD; HIST1H2BF; HIST1H4E; HIST1H2BG; HIST1H2AE; HIST1H3E; HIST1H1D; HIST1H4F; HIST1H4G; HIST1H3F; HIST1H2BH; HIST1H3G; HIST1H2BI; HIST1H4H; BTN3A2; BTN2A2 |
| 3007 | HIST1H1D | 6 | 26342418 | 6p21.3 | gpos 4; | 7.7 | 0.129457478 | -0.05 | 0.283 | 0.38 | -0.518 | HIST1H2BD; HIST1H2BE; HIST1H4D; HIST1H3D; HIST1H2AD; HIST1H2BF; HIST1H4E; HIST1H2BG; HIST1H2AE; HIST1H3E; HIST1H1D; HIST1H4F; HIST1H4G; HIST1H3F; HIST1H2BH; HIST1H3G; HIST1H2BI; HIST1H4H; BTN3A2; BTN2A2; BTN3A1 |
| 8369 | HIST1H4G | 6 | 26354817 | 6p21.3 | gpos 5; | 1.11 | 0.88273518 | 0.058 | -0.029 | 0.494 | 0.052 | HIST1H4D; HIST1H3D; HIST1H2AD; HIST1H2BF; HIST1H4E; HIST1H2BG; HIST1H2AE; HIST1H3E; HIST1H1D; HIST1H4F; HIST1H4G; HIST1H3F; HIST1H2BH; HIST1H3G; HIST1H2BI; HIST1H4H; BTN3A2; BTN2A2; BTN3A1; BTN2A3; BTN3A3 |
| 8968 | HIST1H3F | 6 | 26358348 | 6p22.1 | gpos 6; | -1.26 | 0.270712741 | 0.069 | -0.209 | 0.438 | 0.383 | HIST1H3D; HIST1H2AD; HIST1H2BF; HIST1H4E; HIST1H2BG; HIST1H2AE; HIST1H3E; HIST1H1D; HIST1H4F; HIST1H4G; HIST1H3F; HIST1H2BH; HIST1H3G; HIST1H2BI; HIST1H4H; BTN3A2; BTN2A2; BTN3A1; BTN2A3; BTN3A3; BTN2A1 |
| 8345 | HIST1H2BH | 6 | 26359857 | 6p21.3 | gpos 7; | 1.18 | 0.653683077 | 0.079 | -0.087 | 0.413 | 0.159 | HIST1H2AD; HIST1H2BF; HIST1H4E; HIST1H2BG; HIST1H2AE; HIST1H3E; HIST1H1D; HIST1H4F; HIST1H4G; HIST1H3F; HIST1H2BH; HIST1H3G; HIST1H2BI; HIST1H4H; BTN3A2; BTN2A2; BTN3A1; BTN2A3; BTN3A3; BTN2A1; BTN1A1 |
| 8329 | HIST1H2AI | 6 | 27883955 | 6p22-p21.3 | gneg 5; gpos 2; | -1.45 | 0.490625866 | -0.015 | -0.133 | 0.409 | 0.243 | HIST1H2BJ; HIST1H2AG; HIST1H2BK; HIST1H4I; HIST1H2AH; PRSS16; FKSG83; 346157; ZNF184; HIST1H2BL; HIST1H2AI; HIST1H3H; HIST1H2AJ; HIST1H2BM; HIST1H4J; HIST1H4K; HIST1H2AK; HIST1H2BN; HIST1H2AL; HIST1H1B; HIST1H3I |
| 8357 | HIST1H3H | 6 | 27885820 | 6p22-p21.3 | gneg 5; gpos 1; | -1.44 | 0.360304628 | 0.114 | -0.175 | 0.334 | 0.321 | HIST1H2AG; HIST1H2BK; HIST1H4I; HIST1H2AH; PRSS16; FKSG83; 346157; ZNF184; HIST1H2BL; HIST1H2AI; HIST1H3H; HIST1H2AJ; HIST1H2BM; HIST1H4J; HIST1H4K; HIST1H2AK; HIST1H2BN; HIST1H2AL; HIST1H1B; HIST1H3I; HIST1H4L |
| 8331 | HIST1H2AJ | 6 | 27890058 | 6p22-p21.3 | gneg 5; | -1.23 | 0.013344186 | 0.113 | -0.433 | 0.324 | 0.794 | HIST1H2BK; HIST1H4I; HIST1H2AH; PRSS16; FKSG83; 346157; ZNF184; HIST1H2BL; HIST1H2AI; HIST1H3H; HIST1H2AJ; HIST1H2BM; HIST1H4J; HIST1H4K; HIST1H2AK; HIST1H2BN; HIST1H2AL; HIST1H1B; HIST1H3I; HIST1H4L; HIST1H3J |
| 8342 | HIST1H2BM | 6 | 27890800 | 6p22-p21.3 | gneg 4; | 1.01 | 0.433104942 | 0.019 | -0.15 | 0.451 | 0.276 | HIST1H4I; HIST1H2AH; PRSS16; FKSG83; 346157; ZNF184; HIST1H2BL; HIST1H2AI; HIST1H3H; HIST1H2AJ; HIST1H2BM; HIST1H4J; HIST1H4K; HIST1H2AK; HIST1H2BN; HIST1H2AL; HIST1H1B; HIST1H3I; HIST1H4L; HIST1H3J; HIST1H2AM |
| 8363 | HIST1H4J | 6 | 27899881 | 6p22-p21.3 | gneg 3; | 1.65 | 0.854721218 | 0.034 | 0.035 | 0.423 | -0.065 | HIST1H2AH; PRSS16; FKSG83; 346157; ZNF184; HIST1H2BL; HIST1H2AI; HIST1H3H; HIST1H2AJ; HIST1H2BM; HIST1H4J; HIST1H4K; HIST1H2AK; HIST1H2BN; HIST1H2AL; HIST1H1B; HIST1H3I; HIST1H4L; HIST1H3J; HIST1H2AM; HIST1H2BO |
| 8332 | HIST1H2AL | 6 | 27941085 | 6p22-p21.3 | gneg 1; | 1.86 | 0.866086811 | -0.002 | 0.033 | 0.366 | -0.06 | ZNF184; HIST1H2BL; HIST1H2AI; HIST1H3H; HIST1H2AJ; HIST1H2BM; HIST1H4J; HIST1H4K; HIST1H2AK; HIST1H2BN; HIST1H2AL; HIST1H1B; HIST1H3I; HIST1H4L; HIST1H3J; HIST1H2AM; HIST1H2BO; OR2B2; OR2B6; ZNF165; ZNF435 |
| 3009 | HIST1H1B | 6 | 27942548 | 6p22-p21.3 | gneg 1; | -1.76 | 0.047117868 | -0.054 | -0.36 | 0.316 | 0.66 | HIST1H2BL; HIST1H2AI; HIST1H3H; HIST1H2AJ; HIST1H2BM; HIST1H4J; HIST1H4K; HIST1H2AK; HIST1H2BN; HIST1H2AL; HIST1H1B; HIST1H3I; HIST1H4L; HIST1H3J; HIST1H2AM; HIST1H2BO; OR2B2; OR2B6; ZNF165; ZNF435; ZNF192 |
| 8354 | HIST1H3I | 6 | 27947601 | 6p22-p21.3 | gneg 2; | 1.24 | 0.688410154 | -0.088 | -0.078 | 0.342 | 0.142 | HIST1H2AI; HIST1H3H; HIST1H2AJ; HIST1H2BM; HIST1H4J; HIST1H4K; HIST1H2AK; HIST1H2BN; HIST1H2AL; HIST1H1B; HIST1H3I; HIST1H4L; HIST1H3J; HIST1H2AM; HIST1H2BO; OR2B2; OR2B6; ZNF165; ZNF435; ZNF192; 222701 |
| 81797 | OR12D3 | 6 | 29449178 | 6p21.33 | gneg 7; gpos 1; | 1.27 | 0.211496672 | -0.053 | -0.236 | 0.315 | 0.432 | ZNF323; ZNF306; ZNF305; ZNF453; GPX5; RFP; ZNF311; OR2W1; OR2J2; OR5V1; OR12D3; OR12D2; OR11A1; OR2H1; MAS1L; UBD; OR2H3; GABBR1; MOG; 346171; HLA-F |
| 26529 | OR12D2 | 6 | 29472455 | 6p22.2-p21.31 | gneg 7; gpos 1; | 3.96 | 0.238737571 | -0.004 | 0.223 | 0.318 | -0.409 | ZNF306; ZNF305; ZNF453; GPX5; RFP; ZNF311; OR2W1; OR2J2; OR5V1; OR12D3; OR12D2; OR11A1; OR2H1; MAS1L; UBD; OR2H3; GABBR1; MOG; 346171; HLA-F; HLA-G |
| 26716 | OR2H1 | 6 | 29534208 | 6p21.3 | gneg 6; gpos 1; | 1.31 | 0.970872347 | 0.135 | -0.007 | 0.334 | 0.013 | ZNF453; GPX5; RFP; ZNF311; OR2W1; OR2J2; OR5V1; OR12D3; OR12D2; OR11A1; OR2H1; MAS1L; UBD; OR2H3; GABBR1; MOG; 346171; HLA-F; HLA-G; HLA-A; HCG9 |
| 7932 | OR2H3 | 6 | 29663700 | 6p21.3 | gneg 4; gpos 1; | 1.14 | 0.630327497 | -0.045 | 0.093 | 0.304 | -0.17 | ZNF311; OR2W1; OR2J2; OR5V1; OR12D3; OR12D2; OR11A1; OR2H1; MAS1L; UBD; OR2H3; GABBR1; MOG; 346171; HLA-F; HLA-G; HLA-A; HCG9; ZNRD1; PPP1R11; RNF39 |
| 8870 | IER3 | 6 | 30818955 | 6p21.3 | gneg 2; gpos 5; | 1.1 | 0.466169093 | 0.098 | -0.14 | 0.309 | 0.257 | GNL1; PRR3; ABCF1; PPP1R10; MRPS18B; C6orf134; C6orf136; DHX16; NRM; FLOT1; IER3; DDR1; 389376; DPCR1; C6orf15; PSORS1C1; CDSN; PSORS1C2; C6orf18; TCF19; POU5F1 |
| 221504 | MGC23166 | 6 | 33530346 | 6p21.31 | gneg 5; gpos 1; | -1.2 | 0.03083002 | 0.056 | -0.387 | 0.408 | 0.71 | RPS18; B3GALT4; C6orf11; HKE2; RAB2L; TAPBP; ZNF297; DAXX; PHF1; C6orf82; MGC23166; BAK1; ITPR3; C6orf125; IHPK3; LEMD2; MLN; GRM4; HMGA1; MGC57858; NUDT3 |
| 578 | BAK1 | 6 | 33648306 | 6p21.3 | gneg 6; gpos 1; | 1.02 | 0.223646879 | 0.02 | -0.23 | 0.489 | 0.422 | B3GALT4; C6orf11; HKE2; RAB2L; TAPBP; ZNF297; DAXX; PHF1; C6orf82; MGC23166; BAK1; ITPR3; C6orf125; IHPK3; LEMD2; MLN; GRM4; HMGA1; MGC57858; NUDT3; RPS10 |
| 3710 | ITPR3 | 6 | 33697321 | 6p21 | gneg 6; gpos 1; | 3.38 | 0.000757887 | 0.155 | 0.544 | 0.467 | -0.996 | C6orf11; HKE2; RAB2L; TAPBP; ZNF297; DAXX; PHF1; C6orf82; MGC23166; BAK1; ITPR3; C6orf125; IHPK3; LEMD2; MLN; GRM4; HMGA1; MGC57858; NUDT3; RPS10; PACSIN1 |
| 84300 | C6orf125 | 6 | 33773323 | 6p21.31 | gneg 6; gpos 1; | 1.13 | 0.689411673 | 0.018 | -0.077 | 0.447 | 0.142 | HKE2; RAB2L; TAPBP; ZNF297; DAXX; PHF1; C6orf82; MGC23166; BAK1; ITPR3; C6orf125; IHPK3; LEMD2; MLN; GRM4; HMGA1; MGC57858; NUDT3; RPS10; PACSIN1; SPDEF |
| 221496 | LEMD2 | 6 | 33846968 | 6p21.31 | gneg 8; gpos 1; | -1.04 | 0.025911464 | 0.15 | -0.397 | 0.414 | 0.728 | TAPBP; ZNF297; DAXX; PHF1; C6orf82; MGC23166; BAK1; ITPR3; C6orf125; IHPK3; LEMD2; MLN; GRM4; HMGA1; MGC57858; NUDT3; RPS10; PACSIN1; SPDEF; C6orf106; SNRPC |
| 4295 | MLN | 6 | 33870426 | 6p21.3 | gneg 8; gpos 1; | 3.12 | 0.203871218 | -0.023 | 0.24 | 0.31 | -0.439 | ZNF297; DAXX; PHF1; C6orf82; MGC23166; BAK1; ITPR3; C6orf125; IHPK3; LEMD2; MLN; GRM4; HMGA1; MGC57858; NUDT3; RPS10; PACSIN1; SPDEF; C6orf106; SNRPC; C6orf107 |
| 2914 | GRM4 | 6 | 34097606 | 6p21.3 | gneg 9; gpos 1; | 1.35 | 0.630164963 | 0.013 | 0.093 | 0.57 | -0.17 | DAXX; PHF1; C6orf82; MGC23166; BAK1; ITPR3; C6orf125; IHPK3; LEMD2; MLN; GRM4; HMGA1; MGC57858; NUDT3; RPS10; PACSIN1; SPDEF; C6orf106; SNRPC; C6orf107; TAF11 |
| 3159 | HMGA1 | 6 | 34312906 | 6p21 | gneg 10; gpos 1; | 3.27 | 0.236281069 | -0.051 | 0.224 | 0.383 | -0.411 | PHF1; C6orf82; MGC23166; BAK1; ITPR3; C6orf125; IHPK3; LEMD2; MLN; GRM4; HMGA1; MGC57858; NUDT3; RPS10; PACSIN1; SPDEF; C6orf106; SNRPC; C6orf107; TAF11; ANKS1 |
| 11165 | NUDT3 | 6 | 34363975 | 6p21.2 | gneg 10; gpos 1; | 1.46 | 0.535465889 | 0.062 | 0.119 | 0.41 | -0.219 | MGC23166; BAK1; ITPR3; C6orf125; IHPK3; LEMD2; MLN; GRM4; HMGA1; MGC57858; NUDT3; RPS10; PACSIN1; SPDEF; C6orf106; SNRPC; C6orf107; TAF11; ANKS1; TCP11; 401256 |
| 266727 | MDGA1 | 6 | 37708261 | 6p21 | gneg 4; gpos 7; | 1.15 | 0.689203198 | -0.062 | -0.077 | 0.301 | 0.142 | C6orf89; PI16; MTCH1; FGD2; PIM1; C6orf128; C6orf197; RNF8; KIAA0082; C6orf129; MDGA1; TEX27; BTBD9; GLO1; DNAH8; GLP1R; C6orf64; KCNK5; KCNK17; KCNK16; C6orf102 |
| 60481 | ELOVL5 | 6 | 53240154 | 6p21.1-p12.1 | gneg 1; gpos 10; | 1.86 | 0.00444004 | -0.267 | 0.483 | 0.344 | -0.885 | 401264; TMEM14A; GSTA2; GSTA1; GSTA5; GSTA3; GSTA4; ICK; FBXO9; GCM1; ELOVL5; KIAA1509; GCLC; 401265; LRRC1; C6orf142; TINAG; 221344; C6orf143; HCRTR2; BMP5 |
| 23469 | PHF3 | 6 | 64414389 | 6q12 | gneg 5; gpos 8; | 1.71 | 0.019213672 | 0.077 | 0.414 | 0.3 | -0.759 | C6orf65; ZNF451; KIAA1586; BAG2; RAB23; PRIM2A; SLM1; GLULD1; 389403; PTP4A1; PHF3; EGFL11; BAI3; C6orf209; COL19A1; COL9A1; C6orf57; SMAP1; B3GAT2; 342918; OGFRL1 |
| 167681 | C6orf158 | 6 | 84278992 | 6q15 | gneg 8; gpos 2; | -12.01 | 0.001199158 | -0.063 | -0.53 | 0.301 | 0.971 | TTK; BCKDHB; 340171; C6orf37; TPBG; C6orf157; KIAA1117; PGM3; RWDD2; ME1; C6orf158; C6orf159; NCB5OR; C6orf117; NT5E; SNX14; SYNCRIP; HTR1E; CGA; 389415; GJB7 |
| 51167 | NCB5OR | 6 | 84626123 | 6pter-q22.33 | gneg 9; gpos 2; | 1.84 | 0.044657552 | 0.077 | 0.364 | 0.352 | -0.667 | 340171; C6orf37; TPBG; C6orf157; KIAA1117; PGM3; RWDD2; ME1; C6orf158; C6orf159; NCB5OR; C6orf117; NT5E; SNX14; SYNCRIP; HTR1E; CGA; 389415; GJB7; C6orf162; C6orf163 |
| 10492 | SYNCRIP | 6 | 86380657 | 6q14-q15 | gneg 10; gpos 2; | 2.51 | 6.56E-06 | -0.031 | 0.641 | 0.307 | -1.174 | KIAA1117; PGM3; RWDD2; ME1; C6orf158; C6orf159; NCB5OR; C6orf117; NT5E; SNX14; SYNCRIP; HTR1E; CGA; 389415; GJB7; C6orf162; C6orf163; C6orf165; SLC35A1; RARSL; ORC3L |
| 7164 | TPD52L1 | 6 | 125516577 | 6q22-q23 | gneg 6; gpos 7; | -1.55 | 0.179534564 | 0.175 | -0.252 | 0.374 | 0.463 | GJA1; HSF2; TDE2; PKIB; FABP7; SMPDL3A; C6orf213; TRDN; TCBA1; IBRDC1; TPD52L1; C6orf74; 256096; HEY2; NCOA7; KIAA1755; HINT3; C6orf75; THSD2; RNF146; ECHDC1 |
| 60487 | C6orf75 | 6 | 126349323 | 6q11.1-q22.33 | gneg 5; gpos 7; | 1.7 | 0.014129276 | 0.074 | 0.43 | 0.325 | -0.789 | TRDN; TCBA1; IBRDC1; TPD52L1; C6orf74; 256096; HEY2; NCOA7; KIAA1755; HINT3; C6orf75; THSD2; RNF146; ECHDC1; PTPRK; LAMA2; ARHGAP18; C6orf191; L3MBTL3; SAMD3; KIAA1913 |
| 81847 | RNF146 | 6 | 127629712 | 6q22.1-q22.33 | gneg 4; gpos 7; | 1.15 | 0.284171928 | -0.045 | 0.204 | 0.419 | -0.373 | IBRDC1; TPD52L1; C6orf74; 256096; HEY2; NCOA7; KIAA1755; HINT3; C6orf75; THSD2; RNF146; ECHDC1; PTPRK; LAMA2; ARHGAP18; C6orf191; L3MBTL3; SAMD3; KIAA1913; EPB41L2; AKAP7 |
| 84456 | L3MBTL3 | 6 | 130429194 | 6q23 | gneg 5; gpos 4; | 1.13 | 0.483819165 | 0.092 | 0.135 | 0.302 | -0.247 | KIAA1755; HINT3; C6orf75; THSD2; RNF146; ECHDC1; PTPRK; LAMA2; ARHGAP18; C6orf191; L3MBTL3; SAMD3; KIAA1913; EPB41L2; AKAP7; ARG1; CRSP3; ENPP3; OR2A4; ENPP1; CTGF |
| 114801 | KIAA1913 | 6 | 130799954 | 6q23.1 | gneg 6; gpos 2; | 1.3 | 0.923411543 | -0.105 | -0.019 | 0.312 | 0.034 | C6orf75; THSD2; RNF146; ECHDC1; PTPRK; LAMA2; ARHGAP18; C6orf191; L3MBTL3; SAMD3; KIAA1913; EPB41L2; AKAP7; ARG1; CRSP3; ENPP3; OR2A4; ENPP1; CTGF; MOXD1; STX7 |
| 84262 | MGC10911 | 7 | 1380212 | 7p22.3 | gneg 13; | 1.08 | 0.52435987 | 0.034 | -0.123 | 0.361 | 0.225 | CENTA1; FLJ20359; MGC11257; GPR146; GPR30; LOC90637; 340260; FLJ23471; MAFK; MGC9712; MGC10911; 392617; MAD1L1; FTSJ2; NUDT1; SNX8; EIF3S9; CHST12; C7orf27; KIAA1023; GNA12 |
| 392617 | not_found | 7 | 1560233 | 7p22 | gneg 12; | 1.39 | 0.997362198 | -0.019 | -0.001 | 0.365 | 0.001 | FLJ20359; MGC11257; GPR146; GPR30; LOC90637; 340260; FLJ23471; MAFK; MGC9712; MGC10911; 392617; MAD1L1; FTSJ2; NUDT1; SNX8; EIF3S9; CHST12; C7orf27; KIAA1023; GNA12; CARD11 |
| 4521 | NUDT1 | 7 | 2055097 | 7p22 | gneg 9; gpos 1; | 1.44 | 0.319486015 | -0.078 | 0.19 | 0.466 | -0.348 | GPR30; LOC90637; 340260; FLJ23471; MAFK; MGC9712; MGC10911; 392617; MAD1L1; FTSJ2; NUDT1; SNX8; EIF3S9; CHST12; C7orf27; KIAA1023; GNA12; CARD11; SDK1; FLJ10324; PAPOLB |
| 29886 | SNX8 | 7 | 2067881 | 7p22.3 | gneg 9; gpos 2; | -1.06 | 0.113220289 | -0.052 | -0.294 | 0.482 | 0.539 | LOC90637; 340260; FLJ23471; MAFK; MGC9712; MGC10911; 392617; MAD1L1; FTSJ2; NUDT1; SNX8; EIF3S9; CHST12; C7orf27; KIAA1023; GNA12; CARD11; SDK1; FLJ10324; PAPOLB; PAQR10 |
| 8662 | EIF3S9 | 7 | 2167741 | 7p22.3 | gneg 8; gpos 3; | 1.58 | 0.115600974 | 0.094 | 0.292 | 0.41 | -0.536 | 340260; FLJ23471; MAFK; MGC9712; MGC10911; 392617; MAD1L1; FTSJ2; NUDT1; SNX8; EIF3S9; CHST12; C7orf27; KIAA1023; GNA12; CARD11; SDK1; FLJ10324; PAPOLB; PAQR10; RBAK |
| 55501 | CHST12 | 7 | 2216463 | 7p22 | gneg 7; gpos 3; | 1.02 | 0.275315502 | -0.008 | -0.207 | 0.354 | 0.38 | FLJ23471; MAFK; MGC9712; MGC10911; 392617; MAD1L1; FTSJ2; NUDT1; SNX8; EIF3S9; CHST12; C7orf27; KIAA1023; GNA12; CARD11; SDK1; FLJ10324; PAPOLB; PAQR10; RBAK; 389458 |
| 23288 | KIAA1023 | 7 | 2371872 | 7p22.3 | gneg 6; gpos 5; | 1.17 | 0.480789665 | 0.025 | -0.136 | 0.322 | 0.248 | MGC9712; MGC10911; 392617; MAD1L1; FTSJ2; NUDT1; SNX8; EIF3S9; CHST12; C7orf27; KIAA1023; GNA12; CARD11; SDK1; FLJ10324; PAPOLB; PAQR10; RBAK; 389458; DKFZP434J154; SLC29A4 |
| 28969 | BZW2 | 7 | 16458998 | 7p21.2 | gneg 5; gpos 9; | 1.02 | 0.081375903 | 0.092 | -0.321 | 0.358 | 0.588 | FLJ11273; FLJ14712; SCIN; ARL4; ETV1; DGKB; 392636; MEOX2; SOSTDC1; DKFZP564O043; BZW2; TM4SF13; AGR2; BCMP11; AHR; SNX13; PRPS1L1; 402463; HDAC9; TWIST1; FERD3L |
| 221823 | PRPS1L1 | 7 | 17839637 | 7p21.1 | gneg 9; gpos 7; | 10.55 | 0.014470147 | 0.08 | 0.429 | 0.324 | -0.787 | 392636; MEOX2; SOSTDC1; DKFZP564O043; BZW2; TM4SF13; AGR2; BCMP11; AHR; SNX13; PRPS1L1; 402463; HDAC9; TWIST1; FERD3L; TWISTNB; MGC42090; 7A5; ITGB8; ABCB5; SP8 |
| 221785 | ZNF498 | 7 | 98859221 | 7q22.1 | gneg 14; | 1.27 | 0.759242767 | -0.004 | -0.059 | 0.318 | 0.109 | ARPC1B; PDAP1; G10; KIAA0632; CPSF4; ATP5J2; ZNF394; ZFP95; DKFZp727G131; VIK; ZNF498; CYP3A5; CYP3A7; CYP3A4; CYP3A43; TRIM4; GJE1; AZGP1; 402571; ZNF38; ZNF3 |
| 64599 | PERQ1 | 7 | 99923616 | 7q22 | gneg 3; | 1.57 | 0.026367565 | 0.127 | 0.396 | 0.3 | -0.727 | THG-1; FLJ37538; HRBL; LRRN4; FBXO24; PCOLCE; AF053356_CDS3; TFR2; ACTL6; GNB2; PERQ1; RPP20; EPO; ZAN; EPHB4; 285995; SLC12A9; TRIP6; ARS2; ACHE; MUC17 |
| 10248 | RPP20 | 7 | 99948419 | 7q22 | gneg 4; | 1.94 | 7.56E-06 | -0.039 | 0.639 | 0.353 | -1.171 | FLJ37538; HRBL; LRRN4; FBXO24; PCOLCE; AF053356_CDS3; TFR2; ACTL6; GNB2; PERQ1; RPP20; EPO; ZAN; EPHB4; 285995; SLC12A9; TRIP6; ARS2; ACHE; MUC17; TRIM56 |
| 7205 | TRIP6 | 7 | 100109695 | 7q22 | gneg 5; | 1.45 | 0.580593429 | 0.188 | 0.107 | 0.402 | -0.195 | TFR2; ACTL6; GNB2; PERQ1; RPP20; EPO; ZAN; EPHB4; 285995; SLC12A9; TRIP6; ARS2; ACHE; MUC17; TRIM56; SERPINE1; AP1S1; VGF; FLJ39237; PLOD3; ZNHIT1 |
| 43 | ACHE | 7 | 100132268 | 7q22 | gneg 6; gpos 1; | 1.45 | 0.483827494 | -0.078 | 0.135 | 0.387 | -0.247 | GNB2; PERQ1; RPP20; EPO; ZAN; EPHB4; 285995; SLC12A9; TRIP6; ARS2; ACHE; MUC17; TRIM56; SERPINE1; AP1S1; VGF; FLJ39237; PLOD3; ZNHIT1; CLDN15; TTC11 |
| 140453 | MUC17 | 7 | 100256798 | 7q22 | gneg 7; gpos 1; | 1.27 | 0.831809467 | -0.114 | -0.041 | 0.447 | 0.075 | PERQ1; RPP20; EPO; ZAN; EPHB4; 285995; SLC12A9; TRIP6; ARS2; ACHE; MUC17; TRIM56; SERPINE1; AP1S1; VGF; FLJ39237; PLOD3; ZNHIT1; CLDN15; TTC11; FLJ14117 |
| 60412 | SEC8L1 | 7 | 132395083 | 7q31 | gneg 3; gpos 10; | -1.08 | 0.055779188 | 0.066 | -0.349 | 0.301 | 0.639 | MEST; COPG2; TSGA13; KLF14; 392791; MKLN1; PODXL; DKFZp434G0625; FLJ40288; FLJ20420; SEC8L1; FLJ32786; SLC35B4; AKR1B1; AKR1B10; BPGM; CALD1; LOC340351; FLJ11000; MGC5242; KIAA1466 |
| 673 | BRAF | 7 | 139887462 | 7q34 | gneg 13; gpos 1; | 2.09 | 0.085356375 | -0.141 | 0.317 | 0.309 | -0.582 | HSPC268; LUC7L2; FLJ44186; HIPK2; TBXAS1; ZC3HDC1; SLC37A3; MKRN1; ADCK2; NDUFB2; BRAF; MRPS33; FLJ10842; FLJ40852; SSBP1; TAS2R3; TAS2R4; TAS2R5; 136242; 130075; CLECSF5 |
| 50831 | TAS2R3 | 7 | 140917142 | 7q31.3-q32 | gneg 14; gpos 1; | 1.38 | 0.869387221 | 0.08 | 0.032 | 0.386 | -0.058 | ZC3HDC1; SLC37A3; MKRN1; ADCK2; NDUFB2; BRAF; MRPS33; FLJ10842; FLJ40852; SSBP1; TAS2R3; TAS2R4; TAS2R5; 136242; 130075; CLECSF5; TAS2R38; MGAM; PRSS1; PRSS2; EPHB6 |
| 136242 | not_found | 7 | 140989346 | 7q34 | gneg 11; gpos 3; | 1.43 | 0.558934828 | 0.038 | 0.113 | 0.334 | -0.207 | ADCK2; NDUFB2; BRAF; MRPS33; FLJ10842; FLJ40852; SSBP1; TAS2R3; TAS2R4; TAS2R5; 136242; 130075; CLECSF5; TAS2R38; MGAM; PRSS1; PRSS2; EPHB6; TRPV6; TRPV5; C7orf34 |
| 23601 | CLECSF5 | 7 | 141080340 | 7q33 | gneg 10; gpos 5; | 1.26 | 0.849184212 | 0.115 | -0.037 | 0.332 | 0.068 | BRAF; MRPS33; FLJ10842; FLJ40852; SSBP1; TAS2R3; TAS2R4; TAS2R5; 136242; 130075; CLECSF5; TAS2R38; MGAM; PRSS1; PRSS2; EPHB6; TRPV6; TRPV5; C7orf34; KEL; 135924 |
| 155066 | C7orf32 | 7 | 149007704 | 7q36.1 | gneg 16; gpos 2; | 1.14 | 0.76530437 | 0.037 | -0.058 | 0.325 | 0.106 | CUL1; EZH2; ERP70; DKFZp762I137; ZNF398; ZNF282; ZNF212; KIAA1285; FLJ31413; FLJ12700; C7orf32; MGC3036; C7orf29; RARRES2; LOC203069; REPIN1; MGC33584; LOC155036; LOC285972; hIAN6; hIAN7 |
| 5919 | RARRES2 | 7 | 149473066 | 7q36.1 | gneg 18; gpos 1; | 2.61 | 0.00332317 | 0.205 | 0.494 | 0.311 | -0.906 | DKFZp762I137; ZNF398; ZNF282; ZNF212; KIAA1285; FLJ31413; FLJ12700; C7orf32; MGC3036; C7orf29; RARRES2; LOC203069; REPIN1; MGC33584; LOC155036; LOC285972; hIAN6; hIAN7; HIMAP4; hIAN2; HIMAP2 |
| 8822 | FGF17 | 8 | 21956373 | 8p21 | gneg 9; gpos 5; | 2.2 | 0.24163558 | 0.024 | 0.222 | 0.311 | -0.407 | FLJ10569; LPL; SLC18A1; ATP6V1B2; LZTS1; FLJ40311; GFRA2; DOK2; XPO7; NPM2; FGF17; EPB49; RAI16; FLJ22494; HR; FLJ22246; LGI3; SFTPC; BMP1; PHYHIP; POLR3D |
| 8797 | TNFRSF10A | 8 | 23104915 | 8p21 | gpos 13; | 1.99 | 0.93231322 | 0.054 | 0.016 | 0.323 | -0.03 | PDLIM2; DBC-1; BIN3; FLJ14107; EGR3; MGC22776; RHOBTB2; TNFRSF10B; TNFRSF10C; TNFRSF10D; TNFRSF10A; MGC29816; LOXL2; LYSAL1; MSCP; NKX3-1; 137814; STC1; ADAM28; ADAMDEC1; ADAM7 |
| 91782 | MGC29816 | 8 | 23160103 | 8p21.2 | gpos 12; | 1.08 | 0.195546274 | 0.053 | -0.244 | 0.367 | 0.447 | DBC-1; BIN3; FLJ14107; EGR3; MGC22776; RHOBTB2; TNFRSF10B; TNFRSF10C; TNFRSF10D; TNFRSF10A; MGC29816; LOXL2; LYSAL1; MSCP; NKX3-1; 137814; STC1; ADAM28; ADAMDEC1; ADAM7; NEF3 |
| 51312 | MSCP | 8 | 23442424 | 8p21.2 | gpos 10; | -1.7 | 0.049096708 | 0.043 | -0.357 | 0.38 | 0.655 | EGR3; MGC22776; RHOBTB2; TNFRSF10B; TNFRSF10C; TNFRSF10D; TNFRSF10A; MGC29816; LOXL2; LYSAL1; MSCP; NKX3-1; 137814; STC1; ADAM28; ADAMDEC1; ADAM7; NEF3; NEFL; DOCK5; GNRH1 |
| 137814 | not_found | 8 | 23616172 | 8p21.2 | gneg 1; gpos 10; | 1.27 | 0.586567137 | 0.016 | 0.105 | 0.31 | -0.192 | RHOBTB2; TNFRSF10B; TNFRSF10C; TNFRSF10D; TNFRSF10A; MGC29816; LOXL2; LYSAL1; MSCP; NKX3-1; 137814; STC1; ADAM28; ADAMDEC1; ADAM7; NEF3; NEFL; DOCK5; GNRH1; KCTD9; CDCA2 |
| 6781 | STC1 | 8 | 23755378 | 8p21-p11.2 | gneg 1; gpos 10; | 2.93 | 0.000208783 | 0.099 | 0.577 | 0.359 | -1.059 | TNFRSF10B; TNFRSF10C; TNFRSF10D; TNFRSF10A; MGC29816; LOXL2; LYSAL1; MSCP; NKX3-1; 137814; STC1; ADAM28; ADAMDEC1; ADAM7; NEF3; NEFL; DOCK5; GNRH1; KCTD9; CDCA2; EBF2 |
| 10863 | ADAM28 | 8 | 24207568 | 8p21.2 | gneg 2; gpos 10; | 1.34 | 0.584515654 | -0.014 | -0.105 | 0.306 | 0.193 | TNFRSF10C; TNFRSF10D; TNFRSF10A; MGC29816; LOXL2; LYSAL1; MSCP; NKX3-1; 137814; STC1; ADAM28; ADAMDEC1; ADAM7; NEF3; NEFL; DOCK5; GNRH1; KCTD9; CDCA2; EBF2; PPP2R2A |
| 389654 | not_found | 8 | 48219712 | 19q13.42 | gneg 9; gpos 2; | 1.16 | 0.862168761 | -0.062 | 0.034 | 0.3 | -0.062 | CHRNB3; CHRNA6; THAP1; DKFZP564A022; HOOK3; FNTA; FLJ23356; 340441; LOC91664; 388561; 389654; CEBPD; PRKDC; MCM4; 400638; UBE2V2; FLJ11767; SNAI2; SNTG1; FLJ25471; LOC115294 |
| 1015 | CDH17 | 8 | 95208567 | 8q22.1 | gneg 11; gpos 3; | -20.5 | 0.001911712 | 0.047 | -0.514 | 0.302 | 0.942 | EFCBP1; DKFZp762O076; CGI-77; SLC26A7; CBFA2T1; LOC137392; 388116; 389677; MGC26979; PPM2C; CDH17; GEM; RAD54B; FSBP; DKFZP434I116; FLJ20171; LOC286148; FLJ20530; CCNE2; TP53INP1; MGC40214 |
| 392255 | not_found | 8 | 97223737 | 8q22 | gneg 13; gpos 1; | -3.12 | 0.007815629 | -0.059 | -0.459 | 0.446 | 0.841 | FSBP; DKFZP434I116; FLJ20171; LOC286148; FLJ20530; CCNE2; TP53INP1; MGC40214; PLEKHF2; LOC157657; 392255; UQCRB; CGI-12; PTDSS1; SDC2; PGCP; KIAA1750; LYRIC; LAPTM4B; MATN2; RPL30 |
| 7381 | UQCRB | 8 | 97311910 | 8q22 | gneg 12; gpos 2; | 1.24 | 0.713165574 | 0.221 | 0.071 | 0.328 | -0.13 | DKFZP434I116; FLJ20171; LOC286148; FLJ20530; CCNE2; TP53INP1; MGC40214; PLEKHF2; LOC157657; 392255; UQCRB; CGI-12; PTDSS1; SDC2; PGCP; KIAA1750; LYRIC; LAPTM4B; MATN2; RPL30; FLJ39553 |
| 51001 | CGI-12 | 8 | 97320820 | 8q22.1 | gneg 11; gpos 2; | 2.14 | 3.71E-06 | -0.05 | 0.648 | 0.394 | -1.188 | FLJ20171; LOC286148; FLJ20530; CCNE2; TP53INP1; MGC40214; PLEKHF2; LOC157657; 392255; UQCRB; CGI-12; PTDSS1; SDC2; PGCP; KIAA1750; LYRIC; LAPTM4B; MATN2; RPL30; FLJ39553; UK114 |
| 23246 | BOP1 | 8 | 145456863 | 8q24.3 | gneg 20; gpos 1; | 1.81 | 0.165507347 | 0.226 | 0.26 | 0.39 | -0.477 | FLJ14464; MGC61633; OPLAH; RRP41; GPAA1; CYC1; DKFZP434N1923; MAF1; KIAA1875; LOC51236; BOP1; HSF1; DGAT1; DKFZp547F072; SCRT1; 340393; FBXL6; FLJ11856; ADCK5; CPSF1; SLC39A4 |
| 8694 | DGAT1 | 8 | 145510762 | 8q24.3 | gneg 20; gpos 1; | -1.37 | 9.02E-05 | 0.098 | -0.596 | 0.361 | 1.093 | OPLAH; RRP41; GPAA1; CYC1; DKFZP434N1923; MAF1; KIAA1875; LOC51236; BOP1; HSF1; DGAT1; DKFZp547F072; SCRT1; 340393; FBXL6; FLJ11856; ADCK5; CPSF1; SLC39A4; VPS28; NFKBIL2 |
| 84234 | DKFZp547F072 | 8 | 145525261 | 8q24.3 | gneg 19; gpos 1; | 1.04 | 0.073654821 | -0.231 | -0.328 | 0.467 | 0.602 | RRP41; GPAA1; CYC1; DKFZP434N1923; MAF1; KIAA1875; LOC51236; BOP1; HSF1; DGAT1; DKFZp547F072; SCRT1; 340393; FBXL6; FLJ11856; ADCK5; CPSF1; SLC39A4; VPS28; NFKBIL2; CYHR1 |
| 340393 | not_found | 8 | 145547695 | 8q24.3 | gneg 19; gpos 1; | 1.63 | 0.542162302 | -0.103 | 0.117 | 0.463 | -0.215 | CYC1; DKFZP434N1923; MAF1; KIAA1875; LOC51236; BOP1; HSF1; DGAT1; DKFZp547F072; SCRT1; 340393; FBXL6; FLJ11856; ADCK5; CPSF1; SLC39A4; VPS28; NFKBIL2; CYHR1; KIFC2; FOXH1 |
| 51160 | VPS28 | 8 | 145619807 | 8q24.3 | gneg 19; gpos 1; | 1.1 | 0.351601275 | -0.056 | -0.178 | 0.3 | 0.326 | HSF1; DGAT1; DKFZp547F072; SCRT1; 340393; FBXL6; FLJ11856; ADCK5; CPSF1; SLC39A4; VPS28; NFKBIL2; CYHR1; KIFC2; FOXH1; PPP1R16A; GPT; LOC113655; RECQL4; LRRC14; ZNF34 |
| 4920 | ROR2 | 9 | 91564438 | 9q22 | gneg 2; gpos 6; | -1.39 | 0.306590839 | -0.068 | -0.195 | 0.388 | 0.357 | CKS2; SBP2; LOC349236; SEMA4D; GADD45G; 401539; DIRAS2; SYK; AUH; NFIL3; ROR2; SPTLC1; 389772; IARS; NOL8; OGN; OMD; ASPN; ECM2; C9orf12; BICD2 |
| 80142 | PTGES2 | 9 | 127962526 | 9q34.13 | gneg 12; gpos 1; | 1.7 | 0.480878393 | 0.181 | 0.136 | 0.314 | -0.248 | LOC283050; AK1; ST6GALNAC6; SIAT7D; MGC46424; DPM2; 399665; DKFZp762G199; MCSC; LOC286208; PTGES2; LCN2; C9orf16; CIZ1; DNM1; GOLGA2; TRUB2; COQ4; SLC27A4; TMSL4; C9orf74 |
| 79095 | C9orf16 | 9 | 128002188 | 9q34.1 | gneg 14; | -1.07 | 0.028632438 | 0.084 | -0.391 | 0.453 | 0.718 | ST6GALNAC6; SIAT7D; MGC46424; DPM2; 399665; DKFZp762G199; MCSC; LOC286208; PTGES2; LCN2; C9orf16; CIZ1; DNM1; GOLGA2; TRUB2; COQ4; SLC27A4; TMSL4; C9orf74; CEECAM1; ODF2 |
| 25792 | CIZ1 | 9 | 128007897 | 9q34.1 | gneg 14; | 1.03 | 0.111070202 | -0.21 | -0.296 | 0.441 | 0.542 | SIAT7D; MGC46424; DPM2; 399665; DKFZp762G199; MCSC; LOC286208; PTGES2; LCN2; C9orf16; CIZ1; DNM1; GOLGA2; TRUB2; COQ4; SLC27A4; TMSL4; C9orf74; CEECAM1; ODF2; GLE1L |
| 1759 | DNM1 | 9 | 128045240 | 9q34 | gneg 14; | -1.48 | 0.026213517 | 0.172 | -0.397 | 0.329 | 0.727 | MGC46424; DPM2; 399665; DKFZp762G199; MCSC; LOC286208; PTGES2; LCN2; C9orf16; CIZ1; DNM1; GOLGA2; TRUB2; COQ4; SLC27A4; TMSL4; C9orf74; CEECAM1; ODF2; GLE1L; SPTAN1 |
| 2801 | GOLGA2 | 9 | 128097662 | 9q34.13 | gneg 14; | -1.99 | 0.03028658 | -0.013 | -0.388 | 0.405 | 0.712 | DPM2; 399665; DKFZp762G199; MCSC; LOC286208; PTGES2; LCN2; C9orf16; CIZ1; DNM1; GOLGA2; TRUB2; COQ4; SLC27A4; TMSL4; C9orf74; CEECAM1; ODF2; GLE1L; SPTAN1; MGC20486 |
| 26995 | TRUB2 | 9 | 128150949 | 9q34.13 | gneg 13; | 1.57 | 0.110670452 | 0.114 | 0.296 | 0.343 | -0.543 | 399665; DKFZp762G199; MCSC; LOC286208; PTGES2; LCN2; C9orf16; CIZ1; DNM1; GOLGA2; TRUB2; COQ4; SLC27A4; TMSL4; C9orf74; CEECAM1; ODF2; GLE1L; SPTAN1; MGC20486; SET |
| 51117 | COQ4 | 9 | 128164368 | 9q34.13 | gneg 14; | -1.4 | 0.012573483 | 0.146 | -0.436 | 0.358 | 0.8 | DKFZp762G199; MCSC; LOC286208; PTGES2; LCN2; C9orf16; CIZ1; DNM1; GOLGA2; TRUB2; COQ4; SLC27A4; TMSL4; C9orf74; CEECAM1; ODF2; GLE1L; SPTAN1; MGC20486; SET; pknbeta |
| 81605 | C9orf74 | 9 | 128213193 | 9q34.13 | gneg 14; | 1.03 | 0.090236634 | 0.168 | -0.313 | 0.342 | 0.573 | PTGES2; LCN2; C9orf16; CIZ1; DNM1; GOLGA2; TRUB2; COQ4; SLC27A4; TMSL4; C9orf74; CEECAM1; ODF2; GLE1L; SPTAN1; MGC20486; SET; pknbeta; ZDHHC12; C9orf60; TBC1D13 |
| 4957 | ODF2 | 9 | 128297985 | 9q34.11 | gneg 14; | -1.55 | 0.008295288 | -0.152 | -0.456 | 0.358 | 0.836 | C9orf16; CIZ1; DNM1; GOLGA2; TRUB2; COQ4; SLC27A4; TMSL4; C9orf74; CEECAM1; ODF2; GLE1L; SPTAN1; MGC20486; SET; pknbeta; ZDHHC12; C9orf60; TBC1D13; ENDOG; HSPC109 |
| 2733 | GLE1L | 9 | 128346524 | 9q34.13 | gneg 15; | 1.29 | 0.961127391 | -0.091 | 0.009 | 0.353 | -0.017 | CIZ1; DNM1; GOLGA2; TRUB2; COQ4; SLC27A4; TMSL4; C9orf74; CEECAM1; ODF2; GLE1L; SPTAN1; MGC20486; SET; pknbeta; ZDHHC12; C9orf60; TBC1D13; ENDOG; HSPC109; CCBL1 |
| 142680 | SLC34A3 | 9 | 137401221 | 9q34 | gneg 12; | -1.7 | 0.324511096 | -0.014 | -0.188 | 0.356 | 0.344 | DPP7; GRIN1; 389816; ANAPC2; SSNA1; C9orf75; MGC14327; NR1; DKFZP761H1710; MGC59937; SLC34A3; COBRA1; FLJ20245; FLJ20433; NOXA1; UNQ2492; NELF; FLJ43070; MRPL41; LOC92715; ZMYND19 |
| 25920 | COBRA1 | 9 | 137425783 | 9q34 | gneg 12; | 1.25 | 0.925136369 | 0.193 | 0.018 | 0.413 | -0.033 | GRIN1; 389816; ANAPC2; SSNA1; C9orf75; MGC14327; NR1; DKFZP761H1710; MGC59937; SLC34A3; COBRA1; FLJ20245; FLJ20433; NOXA1; UNQ2492; NELF; FLJ43070; MRPL41; LOC92715; ZMYND19; MGC40555 |
| 10811 | NOXA1 | 9 | 137593683 | 9 | gneg 12; | -1 | 0.166795626 | -0.032 | -0.259 | 0.361 | 0.476 | SSNA1; C9orf75; MGC14327; NR1; DKFZP761H1710; MGC59937; SLC34A3; COBRA1; FLJ20245; FLJ20433; NOXA1; UNQ2492; NELF; FLJ43070; MRPL41; LOC92715; ZMYND19; MGC40555; C9orf37; Eu-HMTase1; CACNA1B |
| 1109 | AKR1C4 | 10 | 5228797 | 10p15-p14 | gneg 8; gpos 1; | 1.46 | 0.862936263 | -0.042 | 0.033 | 0.321 | -0.061 | ADARB2; 399708; 387630; PFKP; PITRM1; COPEB; AKR1CL2; AKR1C1; AKR1C2; AKR1C3; AKR1C4; UCN3; FLJ21665; NET1; CALML5; CALML3; ASB13; GDI2; ANKRD16; FBXO18; IL15RA |
| 810 | CALML3 | 10 | 5556923 | 10pter-p13 | gneg 7; gpos 1; | 1.72 | 0.724771753 | 0.16 | 0.068 | 0.309 | -0.125 | COPEB; AKR1CL2; AKR1C1; AKR1C2; AKR1C3; AKR1C4; UCN3; FLJ21665; NET1; CALML5; CALML3; ASB13; GDI2; ANKRD16; FBXO18; IL15RA; IL2RA; SPF45; PFKFB3; 399716; PRKCQ |
| 2665 | GDI2 | 10 | 5847191 | 10p15 | gneg 8; | 1.17 | 0.916424894 | -0.056 | -0.02 | 0.308 | 0.037 | AKR1C1; AKR1C2; AKR1C3; AKR1C4; UCN3; FLJ21665; NET1; CALML5; CALML3; ASB13; GDI2; ANKRD16; FBXO18; IL15RA; IL2RA; SPF45; PFKFB3; 399716; PRKCQ; ITIH5; ITIH2 |
| 22929 | SEPHS1 | 10 | 13400088 | 10p14 | gneg 5; gpos 13; | 3.92 | 2.11E-06 | -0.13 | 0.655 | 0.302 | -1.201 | DHTKD1; SEC61A2; NUDT5; C10orf7; CAMK1D; DKFZP761F241; OPTN; MCM10; LOC221044; PHYH; SEPHS1; 389873; C10orf30; 342371; PRPF18; FLJ10210; C10orf45; HSP70-4; SUV39H2; DCLRE1C; FLJ11106 |
| 338755 | not_found | 11 | 6745813 | 11p15.4 | gneg 2; gpos 13; | 1.04 | 0.223341762 | -0.014 | -0.23 | 0.304 | 0.422 | TRIM3; ARFIP2; FXC1; FLJ35709; FLJ32752; KIAA0409; ILK; TAF10; CLN2; PCDH16; 338755; 144125; OR6A1; JCG6; 120776; 120775; ZNF215; ZNF214; NALP14; HNRNPG-T; LOC143425 |
| 26610 | ELP4 | 11 | 31487900 | 11p13 | gneg 14; gpos 6; | 1.4 | 0.018743703 | 0.231 | 0.415 | 0.313 | -0.762 | BDNF; DKFZP434G2226; FLJ33979; KCNA4; FSHB; FLJ38968; C11orf8; FLJ46154; LOC341019; FLJ25059; ELP4; PAX6; RCN1; WT1; WIT-1; GA17; TMG4; FLJ21924; LOC91614; FLJ11336; CSTF3 |
| 283257 | not_found | 11 | 54807993 | 11p11.12 | acen 1; gneg 1; gpos 18; | -2.28 | 0.01222313 | 0.001 | -0.438 | 0.357 | 0.802 | 119764; 390113; 256148; 256144; FOLH1; 283247; 283092; 283093; 119749; TRIM48; 283257; 219428; 219429; 219431; 219432; 219436; 219437; 219438; OR5L2; TRIM51; OR5I1 |
| 5553 | PRG2 | 11 | 56911410 | 11q12 | acen 1; gpos 9; | 2.11 | 0.337612371 | 0.082 | 0.183 | 0.307 | -0.336 | 219479; 390162; 219482; 219493; 219527; AGTRL1; TNKS1BP1; SSRP1; P2RX3; PRG3; PRG2; EEG1; NGRH1; SLC43A1; TIMM10; UBE2L6; SERPING1; FLJ30213; HEAB; ZDHHC5; LCMR1 |
| 219539 | FLJ30213 | 11 | 57169136 | 11q12.1 | acen 2; gpos 9; | 1.06 | 0.241602044 | -0.075 | -0.222 | 0.3 | 0.407 | SSRP1; P2RX3; PRG3; PRG2; EEG1; NGRH1; SLC43A1; TIMM10; UBE2L6; SERPING1; FLJ30213; HEAB; ZDHHC5; LCMR1; TMX2; SELH; CTNND1; 219952; 219954; 219956; 219957 |
| 10978 | HEAB | 11 | 57181205 | 11q12 | acen 2; gpos 10; | 1.59 | 0.246735091 | 0.012 | 0.219 | 0.394 | -0.402 | P2RX3; PRG3; PRG2; EEG1; NGRH1; SLC43A1; TIMM10; UBE2L6; SERPING1; FLJ30213; HEAB; ZDHHC5; LCMR1; TMX2; SELH; CTNND1; 219952; 219954; 219956; 219957; 219959 |
| 25921 | ZDHHC5 | 11 | 57192049 | 11q12.1 | acen 2; gpos 11; | 1.23 | 0.681221459 | 0.128 | -0.079 | 0.377 | 0.146 | PRG3; PRG2; EEG1; NGRH1; SLC43A1; TIMM10; UBE2L6; SERPING1; FLJ30213; HEAB; ZDHHC5; LCMR1; TMX2; SELH; CTNND1; 219952; 219954; 219956; 219957; 219959; 219960 |
| 26580 | BSCL2 | 11 | 62214322 | 11q12-q13.5 | gneg 2; gpos 13; | -1.2 | 0.106862376 | -0.099 | -0.299 | 0.309 | 0.548 | FLJ22347; MTA2; FLJ35827; ROM1; B3GAT3; G2AN; KIAA1698; MGC2477; LOC51035; 221091; BSCL2; GNG3; MGC29649; ZBTB3; POLR2G; TAF6L; LOC374395; NXF1; STX5A; FLJ10439; SLC3A2 |
| 10482 | NXF1 | 11 | 62316219 | 11q12-q13 | acen 1; gneg 1; gpos 11; | -1.04 | 0.046226621 | 0.043 | -0.361 | 0.389 | 0.662 | MGC2477; LOC51035; 221091; BSCL2; GNG3; MGC29649; ZBTB3; POLR2G; TAF6L; LOC374395; NXF1; STX5A; FLJ10439; SLC3A2; CHRM1; SLC22A6; SLC22A8; MGC34821; LOC387601; HRLP5; LGALS12 |
| 6811 | STX5A | 11 | 62330945 | 11q12.3 | acen 1; gneg 1; gpos 10; | -1.78 | 0.001065371 | -0.095 | -0.533 | 0.35 | 0.978 | LOC51035; 221091; BSCL2; GNG3; MGC29649; ZBTB3; POLR2G; TAF6L; LOC374395; NXF1; STX5A; FLJ10439; SLC3A2; CHRM1; SLC22A6; SLC22A8; MGC34821; LOC387601; HRLP5; LGALS12; RARRES3 |
| 54663 | FLJ10439 | 11 | 62356959 | 11q12.3 | acen 1; gneg 2; gpos 9; | 2.33 | 0.003268744 | -0.043 | 0.495 | 0.327 | -0.907 | 221091; BSCL2; GNG3; MGC29649; ZBTB3; POLR2G; TAF6L; LOC374395; NXF1; STX5A; FLJ10439; SLC3A2; CHRM1; SLC22A6; SLC22A8; MGC34821; LOC387601; HRLP5; LGALS12; RARRES3; HRASLS2 |
| 1128 | CHRM1 | 11 | 62432727 | 11q13 | acen 1; gneg 4; gpos 8; | 1.13 | 0.565766852 | -0.02 | -0.111 | 0.303 | 0.203 | GNG3; MGC29649; ZBTB3; POLR2G; TAF6L; LOC374395; NXF1; STX5A; FLJ10439; SLC3A2; CHRM1; SLC22A6; SLC22A8; MGC34821; LOC387601; HRLP5; LGALS12; RARRES3; HRASLS2; HRASLS3; DKFZP564J0863 |
| 9376 | SLC22A8 | 11 | 62516872 | 11q11 | acen 1; gneg 5; gpos 7; | 1.15 | 0.196602162 | 0.088 | -0.243 | 0.315 | 0.446 | ZBTB3; POLR2G; TAF6L; LOC374395; NXF1; STX5A; FLJ10439; SLC3A2; CHRM1; SLC22A6; SLC22A8; MGC34821; LOC387601; HRLP5; LGALS12; RARRES3; HRASLS2; HRASLS3; DKFZP564J0863; RTN3; LOC144097 |
| 85329 | LGALS12 | 11 | 63030131 | 11q13 | acen 1; gneg 6; gpos 4; | -1.1 | 0.135139146 | 0.056 | -0.279 | 0.366 | 0.511 | NXF1; STX5A; FLJ10439; SLC3A2; CHRM1; SLC22A6; SLC22A8; MGC34821; LOC387601; HRLP5; LGALS12; RARRES3; HRASLS2; HRASLS3; DKFZP564J0863; RTN3; LOC144097; MARK2; LOC283248; FLJ13848; COX8 |
| 25923 | DKFZP564J0863 | 11 | 63153012 | 11q13.1 | acen 2; gneg 7; gpos 2; | 1.53 | 0.984526489 | -0.161 | -0.004 | 0.326 | 0.007 | CHRM1; SLC22A6; SLC22A8; MGC34821; LOC387601; HRLP5; LGALS12; RARRES3; HRASLS2; HRASLS3; DKFZP564J0863; RTN3; LOC144097; MARK2; LOC283248; FLJ13848; COX8; OTUB1; LRP16; FLRT1; STIP1 |
| 1351 | COX8 | 11 | 63498654 | 11q12-q13 | acen 1; gneg 10; | 1.27 | 0.112225802 | 0.088 | 0.295 | 0.351 | -0.541 | LGALS12; RARRES3; HRASLS2; HRASLS3; DKFZP564J0863; RTN3; LOC144097; MARK2; LOC283248; FLJ13848; COX8; OTUB1; LRP16; FLRT1; STIP1; URP2; MGC11134; MGC13045; DNAJC4; VEGFB; FKBP2 |
| 10963 | STIP1 | 11 | 63710247 | 11q13 | acen 1; gneg 9; | 1.36 | 0.473323105 | 0.231 | 0.138 | 0.322 | -0.253 | DKFZP564J0863; RTN3; LOC144097; MARK2; LOC283248; FLJ13848; COX8; OTUB1; LRP16; FLRT1; STIP1; URP2; MGC11134; MGC13045; DNAJC4; VEGFB; FKBP2; PLCB3; BAD; C11ORF4; KCNK4 |
| 83706 | URP2 | 11 | 63730788 | 11q13.1 | acen 1; gneg 8; | 1.17 | 0.741920764 | -0.422 | -0.064 | 0.312 | 0.117 | RTN3; LOC144097; MARK2; LOC283248; FLJ13848; COX8; OTUB1; LRP16; FLRT1; STIP1; URP2; MGC11134; MGC13045; DNAJC4; VEGFB; FKBP2; PLCB3; BAD; C11ORF4; KCNK4; ESRRA |
| 84304 | MGC13045 | 11 | 63750337 | 11q13.1 | acen 1; gneg 8; | 1.24 | 0.740162773 | 0.331 | -0.064 | 0.374 | 0.118 | MARK2; LOC283248; FLJ13848; COX8; OTUB1; LRP16; FLRT1; STIP1; URP2; MGC11134; MGC13045; DNAJC4; VEGFB; FKBP2; PLCB3; BAD; C11ORF4; KCNK4; ESRRA; HSPC152; PRDX5 |
| 2286 | FKBP2 | 11 | 63766163 | 11q13.1-q13.3 | acen 1; gneg 6; gpos 1; | 1.24 | 0.571812793 | -0.31 | -0.109 | 0.354 | 0.2 | COX8; OTUB1; LRP16; FLRT1; STIP1; URP2; MGC11134; MGC13045; DNAJC4; VEGFB; FKBP2; PLCB3; BAD; C11ORF4; KCNK4; ESRRA; HSPC152; PRDX5; FLJ37970; RPS6KA4; 387777 |
| 5331 | PLCB3 | 11 | 63775697 | 11q13 | acen 1; gneg 7; gpos 1; | 2.45 | 0.016895829 | 0.29 | 0.421 | 0.307 | -0.772 | OTUB1; LRP16; FLRT1; STIP1; URP2; MGC11134; MGC13045; DNAJC4; VEGFB; FKBP2; PLCB3; BAD; C11ORF4; KCNK4; ESRRA; HSPC152; PRDX5; FLJ37970; RPS6KA4; 387777; SLC22A11 |
| 50801 | KCNK4 | 11 | 63815456 | 11q13 | gneg 7; gpos 1; | 1.02 | 0.389757859 | 0.08 | -0.165 | 0.337 | 0.302 | STIP1; URP2; MGC11134; MGC13045; DNAJC4; VEGFB; FKBP2; PLCB3; BAD; C11ORF4; KCNK4; ESRRA; HSPC152; PRDX5; FLJ37970; RPS6KA4; 387777; SLC22A11; SLC22A12; NRXN2; RASGRP2 |
| 9158 | FIBP | 11 | 65407786 | 11q13.1 | gneg 11; gpos 2; | 1.19 | 0.461696955 | 0.154 | -0.141 | 0.306 | 0.259 | RELA; HTATIP; AYP1; LOC91056; OVOL1; FLJ30934; CFL1; MUS81; EFEMP2; CTSW; FIBP; DIPA; FOSL1; P5326; DRAP1; FLJ32880; SART1; MGC11102; BANF1; CST6; CATSPER1 |
| 11007 | DIPA | 11 | 65414450 | 11q12.1 | gneg 11; gpos 2; | 1.42 | 0.382244454 | 0.184 | -0.167 | 0.343 | 0.307 | HTATIP; AYP1; LOC91056; OVOL1; FLJ30934; CFL1; MUS81; EFEMP2; CTSW; FIBP; DIPA; FOSL1; P5326; DRAP1; FLJ32880; SART1; MGC11102; BANF1; CST6; CATSPER1; GAL3ST2 |
| 9986 | RCE1 | 11 | 66367458 | 11q13 | gneg 9; gpos 1; | 1.3 | 0.173378209 | 0.034 | 0.256 | 0.325 | -0.469 | ACTN3; CTSF; FLJ10786; CCS; RBM14; MGC15912; RBM4; MGC10871; SPTBN2; FLJ22531; RCE1; PC; MGC3103; 254439; SYT12; ARHD; FBXL11; ADRBK1; SSH-3; POLD4; RAD9A |
| 78999 | MGC3103 | 11 | 66381548 | 11q13.1 | gneg 10; gpos 1; | 1.91 | 0.03033588 | 0.17 | 0.388 | 0.31 | -0.711 | FLJ10786; CCS; RBM14; MGC15912; RBM4; MGC10871; SPTBN2; FLJ22531; RCE1; PC; MGC3103; 254439; SYT12; ARHD; FBXL11; ADRBK1; SSH-3; POLD4; RAD9A; PPP1CA; FLJ00332 |
| 254439 | not_found | 11 | 66500596 | 11q13.1 | gneg 10; gpos 1; | 1.89 | 0.185027486 | -0.001 | 0.249 | 0.334 | -0.457 | CCS; RBM14; MGC15912; RBM4; MGC10871; SPTBN2; FLJ22531; RCE1; PC; MGC3103; 254439; SYT12; ARHD; FBXL11; ADRBK1; SSH-3; POLD4; RAD9A; PPP1CA; FLJ00332; RPS6KB2 |
| 29984 | ARHD | 11 | 66580896 | 11q14.3 | gneg 11; gpos 1; | -1.63 | 0.00038991 | 0.168 | -0.562 | 0.459 | 1.03 | MGC15912; RBM4; MGC10871; SPTBN2; FLJ22531; RCE1; PC; MGC3103; 254439; SYT12; ARHD; FBXL11; ADRBK1; SSH-3; POLD4; RAD9A; PPP1CA; FLJ00332; RPS6KB2; PTPRCAP; CORO1B |
| 22992 | FBXL11 | 11 | 66644073 | 11q13.1 | gneg 11; gpos 1; | 1.22 | 0.312665649 | 0.001 | 0.192 | 0.365 | -0.353 | RBM4; MGC10871; SPTBN2; FLJ22531; RCE1; PC; MGC3103; 254439; SYT12; ARHD; FBXL11; ADRBK1; SSH-3; POLD4; RAD9A; PPP1CA; FLJ00332; RPS6KB2; PTPRCAP; CORO1B; CABP4 |
| 54961 | SSH-3 | 11 | 66827672 | 11q13.1 | gneg 13; gpos 1; | 1.23 | 0.528502046 | 0.027 | -0.121 | 0.508 | 0.223 | SPTBN2; FLJ22531; RCE1; PC; MGC3103; 254439; SYT12; ARHD; FBXL11; ADRBK1; SSH-3; POLD4; RAD9A; PPP1CA; FLJ00332; RPS6KB2; PTPRCAP; CORO1B; CABP4; FLJ21749; AIP |
| 5883 | RAD9A | 11 | 66915998 | 11q13.1-q13.2 | gneg 12; gpos 1; | 1.21 | 0.732365935 | 0.082 | 0.066 | 0.503 | -0.121 | RCE1; PC; MGC3103; 254439; SYT12; ARHD; FBXL11; ADRBK1; SSH-3; POLD4; RAD9A; PPP1CA; FLJ00332; RPS6KB2; PTPRCAP; CORO1B; CABP4; FLJ21749; AIP; PITPNM1; DOC-1R |
| 5499 | PPP1CA | 11 | 66922227 | 11q13 | gneg 13; gpos 1; | -1.04 | 0.081342265 | 0.031 | -0.321 | 0.535 | 0.588 | PC; MGC3103; 254439; SYT12; ARHD; FBXL11; ADRBK1; SSH-3; POLD4; RAD9A; PPP1CA; FLJ00332; RPS6KB2; PTPRCAP; CORO1B; CABP4; FLJ21749; AIP; PITPNM1; DOC-1R; CABP2 |
| 6199 | RPS6KB2 | 11 | 66952562 | 11q13.1 | gneg 13; gpos 1; | -1.57 | 0.001023028 | -0.017 | -0.534 | 0.522 | 0.98 | 254439; SYT12; ARHD; FBXL11; ADRBK1; SSH-3; POLD4; RAD9A; PPP1CA; FLJ00332; RPS6KB2; PTPRCAP; CORO1B; CABP4; FLJ21749; AIP; PITPNM1; DOC-1R; CABP2; GSTP1; FLJ90834 |
| 5790 | PTPRCAP | 11 | 66959556 | 11q13.3 | gneg 12; gpos 1; | 1.44 | 0.920913349 | 0.078 | -0.019 | 0.465 | 0.035 | SYT12; ARHD; FBXL11; ADRBK1; SSH-3; POLD4; RAD9A; PPP1CA; FLJ00332; RPS6KB2; PTPRCAP; CORO1B; CABP4; FLJ21749; AIP; PITPNM1; DOC-1R; CABP2; GSTP1; FLJ90834; NDUFV1 |
| 57175 | CORO1B | 11 | 66962093 | 11q13.1 | gneg 12; gpos 1; | -1.04 | 0.034403075 | 0.202 | -0.38 | 0.473 | 0.697 | ARHD; FBXL11; ADRBK1; SSH-3; POLD4; RAD9A; PPP1CA; FLJ00332; RPS6KB2; PTPRCAP; CORO1B; CABP4; FLJ21749; AIP; PITPNM1; DOC-1R; CABP2; GSTP1; FLJ90834; NDUFV1; NUDT8 |
| 9049 | AIP | 11 | 67007096 | 11q13.3 | gneg 13; | -1.32 | 0.002073381 | 0.036 | -0.511 | 0.483 | 0.937 | SSH-3; POLD4; RAD9A; PPP1CA; FLJ00332; RPS6KB2; PTPRCAP; CORO1B; CABP4; FLJ21749; AIP; PITPNM1; DOC-1R; CABP2; GSTP1; FLJ90834; NDUFV1; NUDT8; TBX10; ACY-3; ALDH3B2 |
| 51475 | CABP2 | 11 | 67042993 | 11q13.1 | gneg 12; | 1.38 | 0.810941687 | -0.031 | 0.046 | 0.415 | -0.085 | PPP1CA; FLJ00332; RPS6KB2; PTPRCAP; CORO1B; CABP4; FLJ21749; AIP; PITPNM1; DOC-1R; CABP2; GSTP1; FLJ90834; NDUFV1; NUDT8; TBX10; ACY-3; ALDH3B2; UNC93B1; ALDH3B1; NDUFS8 |
| 2950 | GSTP1 | 11 | 67107861 | 11q13 | gneg 12; | -1.05 | 0.211914304 | 0.124 | -0.236 | 0.322 | 0.432 | FLJ00332; RPS6KB2; PTPRCAP; CORO1B; CABP4; FLJ21749; AIP; PITPNM1; DOC-1R; CABP2; GSTP1; FLJ90834; NDUFV1; NUDT8; TBX10; ACY-3; ALDH3B2; UNC93B1; ALDH3B1; NDUFS8; TCIRG1 |
| 254552 | NUDT8 | 11 | 67151985 | 11q13.1 | gneg 10; gpos 1; | -1.19 | 0.016772781 | 0.114 | -0.421 | 0.337 | 0.773 | CORO1B; CABP4; FLJ21749; AIP; PITPNM1; DOC-1R; CABP2; GSTP1; FLJ90834; NDUFV1; NUDT8; TBX10; ACY-3; ALDH3B2; UNC93B1; ALDH3B1; NDUFS8; TCIRG1; CHK; CGI-85; C11orf24 |
| 51083 | GAL | 11 | 68208610 | 11q13.1 | gneg 5; gpos 3; | 8.58 | 0.114073509 | 0.056 | 0.294 | 0.322 | -0.538 | UNC93B1; ALDH3B1; NDUFS8; TCIRG1; CHK; CGI-85; C11orf24; LRP5; 284422; C11orf23; GAL; MTL5; CPT1A; MRPL21; IGHMBP2; MRGD; MGC21621; TPCN2; MYEOV; CCND1; ORAOV1 |
| 219927 | MRPL21 | 11 | 68415322 | 11q13.1 | gneg 7; gpos 3; | 1.16 | 0.887826582 | -0.092 | 0.027 | 0.332 | -0.05 | TCIRG1; CHK; CGI-85; C11orf24; LRP5; 284422; C11orf23; GAL; MTL5; CPT1A; MRPL21; IGHMBP2; MRGD; MGC21621; TPCN2; MYEOV; CCND1; ORAOV1; FGF19; FGF4; FGF3 |
| 5058 | PAK1 | 11 | 76710708 | 11q13-q14 | gneg 8; gpos 9; | 3.09 | 6.28E-05 | -0.117 | 0.603 | 0.352 | -1.106 | WNT11; PRKRIR; C11orf30; E2IG4; PHCA; IMAGE:4907098; CAPN5; OMP; MYO7A; LOC220032; PAK1; AQP11; CLNS1A; HBXAP; PTD015; MGC16733; KCTD14; THRSP; NDUFC2; ALG8; GAB2 |
| 65987 | KCTD14 | 11 | 77404409 | 11q13.4 | gneg 5; gpos 10; | 2.44 | 0.004102278 | -0.059 | 0.486 | 0.321 | -0.891 | CAPN5; OMP; MYO7A; LOC220032; PAK1; AQP11; CLNS1A; HBXAP; PTD015; MGC16733; KCTD14; THRSP; NDUFC2; ALG8; GAB2; FLJ23441; ODZ4; MGC33846; PRCP; FLJ25416; RAB30 |
| 120103 | SLC36A4 | 11 | 92520499 | 11q14.3 | gneg 8; gpos 6; | 1.15 | 0.448226257 | 0.145 | 0.146 | 0.366 | -0.267 | PSMAL/GCP; RNF18; 399940; 120146; NAALAD2; CHORDC1; KIAA1917; 120105; FAT3; MTNR1B; SLC36A4; FLJ25393; FN5; MGC5306; PTD012; CRSP6; LOC341208; PANX1; 387805; GPR83; MRE11A |
| 338661 | not_found | 11 | 123260430 | 11q24.1 | gneg 4; gpos 10; | 1.57 | 0.964898645 | 0.048 | -0.009 | 0.385 | 0.016 | TECTA; SC5DL; SORL1; KIAA1959; CRTAM; FLJ23554; HSPA8; ASAM; SCN3B; ZNF202; 338661; 338662; 219875; 219874; 219873; 390264; 219870; 219869; 390265; 399961; LOH11CR2A |
| 219874 | not_found | 11 | 123318783 | 11q24.1 | gneg 6; gpos 10; | -1 | 0.169221802 | -0.055 | -0.258 | 0.382 | 0.473 | KIAA1959; CRTAM; FLJ23554; HSPA8; ASAM; SCN3B; ZNF202; 338661; 338662; 219875; 219874; 219873; 390264; 219870; 219869; 390265; 399961; LOH11CR2A; 219865; 283159; 283160 |
| 219873 | not_found | 11 | 123352612 | 11q24.1 | gneg 6; gpos 9; | 1.61 | 0.905263173 | 0.037 | -0.023 | 0.4 | 0.042 | CRTAM; FLJ23554; HSPA8; ASAM; SCN3B; ZNF202; 338661; 338662; 219875; 219874; 219873; 390264; 219870; 219869; 390265; 399961; LOH11CR2A; 219865; 283159; 283160; 390271 |
| 219870 | not_found | 11 | 123398929 | 11q24.2 | gneg 8; gpos 8; | 1.41 | 0.864556524 | 0.03 | -0.033 | 0.32 | 0.061 | HSPA8; ASAM; SCN3B; ZNF202; 338661; 338662; 219875; 219874; 219873; 390264; 219870; 219869; 390265; 399961; LOH11CR2A; 219865; 283159; 283160; 390271; 283162; OR8B8 |
| 219869 | not_found | 11 | 123405539 | 11q24.2 | gneg 9; gpos 7; | -1 | 0.057318487 | 0.032 | -0.347 | 0.329 | 0.636 | ASAM; SCN3B; ZNF202; 338661; 338662; 219875; 219874; 219873; 390264; 219870; 219869; 390265; 399961; LOH11CR2A; 219865; 283159; 283160; 390271; 283162; OR8B8; 219858 |
| 390265 | not_found | 11 | 123413982 | 11q23 | gneg 9; gpos 6; | 1.02 | 0.292614716 | -0.111 | -0.2 | 0.372 | 0.367 | SCN3B; ZNF202; 338661; 338662; 219875; 219874; 219873; 390264; 219870; 219869; 390265; 399961; LOH11CR2A; 219865; 283159; 283160; 390271; 283162; OR8B8; 219858; 390275 |
| 283160 | not_found | 11 | 123694367 | 11q24.2 | gneg 11; gpos 2; | -1 | 0.027615941 | 0.11 | -0.394 | 0.343 | 0.722 | 219874; 219873; 390264; 219870; 219869; 390265; 399961; LOH11CR2A; 219865; 283159; 283160; 390271; 283162; OR8B8; 219858; 390275; PANX3; TBRG1; CSE-C; SPA17; NRGN |
| 116337 | PANX3 | 11 | 123986662 | 11q24.2 | gneg 14; | 2.99 | 0.295426496 | 0.069 | 0.199 | 0.305 | -0.365 | 399961; LOH11CR2A; 219865; 283159; 283160; 390271; 283162; OR8B8; 219858; 390275; PANX3; TBRG1; CSE-C; SPA17; NRGN; CTXL; ESAM; FLJ23342; ROBO4; FLJ25530; FLJ13215 |
| 84897 | TBRG1 | 11 | 123997980 | 11q24.2 | gneg 15; | -1.8 | 0.058716893 | -0.012 | -0.345 | 0.327 | 0.632 | LOH11CR2A; 219865; 283159; 283160; 390271; 283162; OR8B8; 219858; 390275; PANX3; TBRG1; CSE-C; SPA17; NRGN; CTXL; ESAM; FLJ23342; ROBO4; FLJ25530; FLJ13215; SLC37A2 |
| 6484 | SIAT4C | 11 | 125731305 | 11q23-q24 | gneg 8; gpos 3; | 1.84 | 0.911706031 | 0.05 | 0.021 | 0.32 | -0.039 | DDX25; LOC338667; CDON; FLJ14494; FLJ21103; SRPR; H17; TIRAP; DCPS; 399972; SIAT4C; KIRREL3; FLJ40224; 283166; ETS1; 387821; FLI1; KCNJ1; KCNJ5; MGC35558; P53AIP1 |
| 51729 | WBP11 | 12 | 14830680 | 12p13.1 | gneg 6; gpos 7; | 1.63 | 0.013610985 | -0.04 | 0.432 | 0.306 | -0.792 | HEBP1; GSG1; EMP1; FLJ33810; GRIN2B; ATF7IP; FLJ22662; GUCY2C; HIST4H4; H2AFJ; WBP11; MGC47869; DO; MGP; FLJ32115; ARHGDIB; PDE6H; RERG; PTPRO; EPS8; UNRIP |
| 420 | DO | 12 | 14873513 | 12q13.2-q13.3 | gneg 7; gpos 5; | -26.07 | 0.001717068 | -0.032 | -0.518 | 0.313 | 0.949 | EMP1; FLJ33810; GRIN2B; ATF7IP; FLJ22662; GUCY2C; HIST4H4; H2AFJ; WBP11; MGC47869; DO; MGP; FLJ32115; ARHGDIB; PDE6H; RERG; PTPRO; EPS8; UNRIP; CGI-26; MGST1 |
| 4256 | MGP | 12 | 14926093 | 12p13.1-p12.3 | gneg 8; gpos 4; | -30.15 | 0.010458214 | -0.044 | -0.445 | 0.327 | 0.816 | FLJ33810; GRIN2B; ATF7IP; FLJ22662; GUCY2C; HIST4H4; H2AFJ; WBP11; MGC47869; DO; MGP; FLJ32115; ARHGDIB; PDE6H; RERG; PTPRO; EPS8; UNRIP; CGI-26; MGST1; DAT1 |
| 85004 | RERG | 12 | 15151984 | 12p13.1 | gneg 8; gpos 4; | -5.26 | 0.000857176 | 0.184 | -0.54 | 0.379 | 0.99 | GUCY2C; HIST4H4; H2AFJ; WBP11; MGC47869; DO; MGP; FLJ32115; ARHGDIB; PDE6H; RERG; PTPRO; EPS8; UNRIP; CGI-26; MGST1; DAT1; FLJ22655; PIK3C2G; PLCZ1; CAPPA3 |
| 11171 | UNRIP | 12 | 15926612 | 12p13.1 | gneg 7; gpos 5; | 1.54 | 0.018145342 | -0.06 | 0.417 | 0.319 | -0.765 | WBP11; MGC47869; DO; MGP; FLJ32115; ARHGDIB; PDE6H; RERG; PTPRO; EPS8; UNRIP; CGI-26; MGST1; DAT1; FLJ22655; PIK3C2G; PLCZ1; CAPPA3; PEPP2; AEBP2; PDE3A |
| 2647 | GCN5L1 | 12 | 54396087 | 12q13-q14 | gneg 10; | -1 | 0.23330064 | 0.083 | -0.226 | 0.311 | 0.413 | PDE1B; PPP1R1A; LACRT; DCD; LOC118430; NEUROD4; 254786; 390326; MGC17301; ITGA7; GCN5L1; RDH5; CD63; GDF11; CIP29; ORMDL2; DNAJ; MMP19; PYM; DGKA; SILV |
| 2583 | GALGT | 12 | 56305944 | 12q13.3 | gneg 3; gpos 8; | 3.63 | 0.977537572 | 0.097 | -0.005 | 0.305 | 0.01 | ARHGAP9; MARS; DDIT3; MBD6; DCTN2; KIF5A; PIP5K2C; FLJ34766; GEFT; SLC26A10; GALGT; OS-9; CENTG1; SAS; CDK4; LOC92979; CYP27B1; METTL1; DKFZP586D0919; TSFM; AVIL |
| 6636 | SNRPF | 12 | 94755206 | 12q23.1 | gneg 4; gpos 12; | 1.98 | 8.14E-06 | 0.085 | 0.637 | 0.324 | -1.169 | CRADD; PLXNC1; NY-REN-58; KIAA1145; DAP13; NR2C1; VEZATIN; METAP2; USP44; NTN4; SNRPF; FLJ40089; MGC35366; HAL; LTA4H; ELK3; PCTK2; FLJ44112; NEDD1; TMPO; SLC25A3 |
| 4048 | LTA4H | 12 | 94897078 | 12q22 | gneg 3; gpos 12; | 1.68 | 0.005060482 | -0.072 | 0.477 | 0.353 | -0.875 | DAP13; NR2C1; VEZATIN; METAP2; USP44; NTN4; SNRPF; FLJ40089; MGC35366; HAL; LTA4H; ELK3; PCTK2; FLJ44112; NEDD1; TMPO; SLC25A3; FLJ31051; APAF1; EB-1; MGC39520 |
| 5128 | PCTK2 | 12 | 95174507 | 12q23.1 | gneg 3; gpos 12; | 1.38 | 0.312336136 | 0.11 | 0.192 | 0.353 | -0.353 | VEZATIN; METAP2; USP44; NTN4; SNRPF; FLJ40089; MGC35366; HAL; LTA4H; ELK3; PCTK2; FLJ44112; NEDD1; TMPO; SLC25A3; FLJ31051; APAF1; EB-1; MGC39520; DKFZp434M0331; ACTR6 |
| 121441 | NEDD1 | 12 | 95803711 | 12q23.1 | gneg 3; gpos 12; | 1.77 | 0.020121094 | 0.049 | 0.412 | 0.425 | -0.755 | USP44; NTN4; SNRPF; FLJ40089; MGC35366; HAL; LTA4H; ELK3; PCTK2; FLJ44112; NEDD1; TMPO; SLC25A3; FLJ31051; APAF1; EB-1; MGC39520; DKFZp434M0331; ACTR6; FLJ33505; FLJ10074 |
| 7112 | TMPO | 12 | 97411909 | 12q22 | gneg 3; gpos 12; | 3.24 | 2.05E-05 | 0.137 | 0.623 | 0.357 | -1.143 | NTN4; SNRPF; FLJ40089; MGC35366; HAL; LTA4H; ELK3; PCTK2; FLJ44112; NEDD1; TMPO; SLC25A3; FLJ31051; APAF1; EB-1; MGC39520; DKFZp434M0331; ACTR6; FLJ33505; FLJ10074; SLC17A8 |
| 83468 | LOC83468 | 12 | 102885231 | 12q | gneg 5; gpos 4; | 1.37 | 0.756261326 | 0.026 | 0.06 | 0.383 | -0.11 | PMCH; IGF1; PAH; ASCL1; FLJ25323; STAB2; TU12B1-TY; 253724; TRA1; TDG; LOC83468; HCF-2; NFYB; TXNRD1; CHST11; DKFZP434K0427; MGC40397; KIAA1033; DIP13B; ARK5; CKAP4 |
| 51228 | GLTP | 12 | 108751468 | 12q24.12 | gneg 4; gpos 9; | 1.59 | 0.729613435 | 0.042 | 0.067 | 0.306 | -0.123 | 121642; UNG; ACACB; FLJ37587; KCTD10; UBE3B; MMAB; MVK; FLJ14721; TRPV4; GLTP; MGC10854; GIT2; ANKRD13; CDV-1; ATP2A2; ANAPC7; ARPC3; MGC14560; HSU79274; VPS29 |
| 84260 | MGC10854 | 12 | 108801042 | 12q24.12 | gneg 5; gpos 8; | 4.42 | 0.164497775 | 0.043 | 0.261 | 0.345 | -0.478 | UNG; ACACB; FLJ37587; KCTD10; UBE3B; MMAB; MVK; FLJ14721; TRPV4; GLTP; MGC10854; GIT2; ANKRD13; CDV-1; ATP2A2; ANAPC7; ARPC3; MGC14560; HSU79274; VPS29; RAD9B |
| 9815 | GIT2 | 12 | 108848588 | 12q24.1 | gneg 6; gpos 8; | 1.73 | 0.036315831 | -0.084 | 0.377 | 0.364 | -0.691 | ACACB; FLJ37587; KCTD10; UBE3B; MMAB; MVK; FLJ14721; TRPV4; GLTP; MGC10854; GIT2; ANKRD13; CDV-1; ATP2A2; ANAPC7; ARPC3; MGC14560; HSU79274; VPS29; RAD9B; TA-PP2C |
| 488 | ATP2A2 | 12 | 109182151 | 12q23-q24.1 | gneg 8; gpos 6; | 1.68 | 0.021664725 | 0.027 | 0.407 | 0.377 | -0.747 | UBE3B; MMAB; MVK; FLJ14721; TRPV4; GLTP; MGC10854; GIT2; ANKRD13; CDV-1; ATP2A2; ANAPC7; ARPC3; MGC14560; HSU79274; VPS29; RAD9B; TA-PP2C; FLJ21127; MGC15619; PPP1CC |
| 10094 | ARPC3 | 12 | 109335426 | 12q24.11 | gneg 9; gpos 5; | 1.22 | 0.598802951 | 0.115 | -0.102 | 0.443 | 0.186 | MVK; FLJ14721; TRPV4; GLTP; MGC10854; GIT2; ANKRD13; CDV-1; ATP2A2; ANAPC7; ARPC3; MGC14560; HSU79274; VPS29; RAD9B; TA-PP2C; FLJ21127; MGC15619; PPP1CC; FLJ35843; MYL2 |
| 29902 | HSU79274 | 12 | 109368984 | 12q24.13 | gneg 11; gpos 4; | 2.04 | 0.013495466 | 0.052 | 0.433 | 0.343 | -0.793 | TRPV4; GLTP; MGC10854; GIT2; ANKRD13; CDV-1; ATP2A2; ANAPC7; ARPC3; MGC14560; HSU79274; VPS29; RAD9B; TA-PP2C; FLJ21127; MGC15619; PPP1CC; FLJ35843; MYL2; CUTL2; FLJ32356 |
| 51699 | VPS29 | 12 | 109392049 | 12q24 | gneg 11; gpos 4; | 1.54 | 0.020659119 | 0.087 | 0.41 | 0.326 | -0.752 | GLTP; MGC10854; GIT2; ANKRD13; CDV-1; ATP2A2; ANAPC7; ARPC3; MGC14560; HSU79274; VPS29; RAD9B; TA-PP2C; FLJ21127; MGC15619; PPP1CC; FLJ35843; MYL2; CUTL2; FLJ32356; LNK |
| 144715 | RAD9B | 12 | 109402819 | 12q24.13 | gneg 11; gpos 3; | -1.76 | 0.06622415 | 0.051 | -0.336 | 0.327 | 0.617 | MGC10854; GIT2; ANKRD13; CDV-1; ATP2A2; ANAPC7; ARPC3; MGC14560; HSU79274; VPS29; RAD9B; TA-PP2C; FLJ21127; MGC15619; PPP1CC; FLJ35843; MYL2; CUTL2; FLJ32356; LNK; SCA2 |
| 51569 | BM-002 | 13 | 37822017 | 13q13.3 | gneg 3; gpos 8; | 1.48 | 0.257132641 | 0.042 | 0.215 | 0.3 | -0.394 | CCNA1; 400120; RFXAP; MADH9; ALG5; OIP2; P38IP; MGC33182; OSF-2; TRPC4; BM-002; DKFZp686J0811; STOML3; FLJ12661; LHFP; COG6; 400123; FOXO1A; MRPS31; SLC25A15; ELF1 |
| 80209 | FLJ12661 | 13 | 38482002 | 13q13.3 | gneg 3; gpos 11; | 2.42 | 0.000617523 | -0.197 | 0.549 | 0.367 | -1.007 | MADH9; ALG5; OIP2; P38IP; MGC33182; OSF-2; TRPC4; BM-002; DKFZp686J0811; STOML3; FLJ12661; LHFP; COG6; 400123; FOXO1A; MRPS31; SLC25A15; ELF1; WBP4; KBTBD6; KBTBD7 |
| 10186 | LHFP | 13 | 38815029 | 13q12 | gneg 3; gpos 11; | -1.44 | 0.151203883 | 0.116 | -0.269 | 0.36 | 0.493 | ALG5; OIP2; P38IP; MGC33182; OSF-2; TRPC4; BM-002; DKFZp686J0811; STOML3; FLJ12661; LHFP; COG6; 400123; FOXO1A; MRPS31; SLC25A15; ELF1; WBP4; KBTBD6; KBTBD7; MTRF1 |
| 57511 | COG6 | 13 | 39127813 | 13q13.3 | gneg 2; gpos 12; | -1.13 | 0.49447963 | 0.016 | -0.131 | 0.362 | 0.241 | OIP2; P38IP; MGC33182; OSF-2; TRPC4; BM-002; DKFZp686J0811; STOML3; FLJ12661; LHFP; COG6; 400123; FOXO1A; MRPS31; SLC25A15; ELF1; WBP4; KBTBD6; KBTBD7; MTRF1; FLJ22054 |
| 10166 | SLC25A15 | 13 | 40261727 | 13q14 | gpos 13; | 1.62 | 0.003832007 | 0.094 | 0.489 | 0.341 | -0.896 | TRPC4; BM-002; DKFZp686J0811; STOML3; FLJ12661; LHFP; COG6; 400123; FOXO1A; MRPS31; SLC25A15; ELF1; WBP4; KBTBD6; KBTBD7; MTRF1; FLJ22054; RGC32; 400125; DGKH; AKAP11 |
| 11193 | WBP4 | 13 | 40533696 | 13q13.3 | gneg 1; gpos 12; | 1.12 | 0.493189254 | 0.084 | 0.132 | 0.324 | -0.242 | DKFZp686J0811; STOML3; FLJ12661; LHFP; COG6; 400123; FOXO1A; MRPS31; SLC25A15; ELF1; WBP4; KBTBD6; KBTBD7; MTRF1; FLJ22054; RGC32; 400125; DGKH; AKAP11; TNFSF11; FLJ40919 |
| 84078 | KBTBD7 | 13 | 40663710 | 13q13.3 | gneg 1; gpos 11; | 3.41 | 0.00675212 | -0.069 | 0.465 | 0.339 | -0.853 | FLJ12661; LHFP; COG6; 400123; FOXO1A; MRPS31; SLC25A15; ELF1; WBP4; KBTBD6; KBTBD7; MTRF1; FLJ22054; RGC32; 400125; DGKH; AKAP11; TNFSF11; FLJ40919; EPSTI1; DNAJD1 |
| 9617 | MTRF1 | 13 | 40688515 | 13q14.1-q14.3 | gneg 2; gpos 10; | 1.07 | 0.897107027 | 0.152 | -0.025 | 0.346 | 0.046 | LHFP; COG6; 400123; FOXO1A; MRPS31; SLC25A15; ELF1; WBP4; KBTBD6; KBTBD7; MTRF1; FLJ22054; RGC32; 400125; DGKH; AKAP11; TNFSF11; FLJ40919; EPSTI1; DNAJD1; FLJ10094 |
| 51339 | DACT1 | 14 | 58174611 | 14q23.1 | gneg 6; gpos 11; | -1.6 | 0.370757023 | 0.143 | -0.171 | 0.321 | 0.314 | SEC10L1; C14orf108; C14orf35; C14orf105; LOC341880; ACTR10; PSMA3; RBBP1; TIMM9; KIAA0586; DACT1; DAAM1; GPR135; FLJ25436; C14orf100; RTN1; FLJ46156; C14orf135; DHRS7; PPM1A; C14orf39 |
| 23002 | DAAM1 | 14 | 58725151 | 14q23.1 | gneg 5; gpos 11; | -1.05 | 0.878880494 | -0.012 | 0.03 | 0.323 | -0.054 | C14orf108; C14orf35; C14orf105; LOC341880; ACTR10; PSMA3; RBBP1; TIMM9; KIAA0586; DACT1; DAAM1; GPR135; FLJ25436; C14orf100; RTN1; FLJ46156; C14orf135; DHRS7; PPM1A; C14orf39; SIX6 |
| 51528 | C14orf100 | 14 | 59020978 | 14q23.1 | gneg 2; gpos 12; | 1.1 | 0.950997519 | 0.215 | -0.012 | 0.385 | 0.022 | LOC341880; ACTR10; PSMA3; RBBP1; TIMM9; KIAA0586; DACT1; DAAM1; GPR135; FLJ25436; C14orf100; RTN1; FLJ46156; C14orf135; DHRS7; PPM1A; C14orf39; SIX6; SIX1; SIX4; MNAT1 |
| 64430 | C14orf135 | 14 | 59652410 | 14q23.1 | gpos 14; | -1.03 | 0.737478826 | 0.129 | -0.065 | 0.404 | 0.119 | RBBP1; TIMM9; KIAA0586; DACT1; DAAM1; GPR135; FLJ25436; C14orf100; RTN1; FLJ46156; C14orf135; DHRS7; PPM1A; C14orf39; SIX6; SIX1; SIX4; MNAT1; SLC38A6; LOC161291; PRKCH |
| 51635 | DHRS7 | 14 | 59681251 | 14q23.1 | gpos 14; | -1.14 | 0.145468549 | 0.093 | -0.272 | 0.441 | 0.499 | TIMM9; KIAA0586; DACT1; DAAM1; GPR135; FLJ25436; C14orf100; RTN1; FLJ46156; C14orf135; DHRS7; PPM1A; C14orf39; SIX6; SIX1; SIX4; MNAT1; SLC38A6; LOC161291; PRKCH; HIF1A |
| 677 | ZFP36L1 | 14 | 68324127 | 14q22-q24 | gneg 12; | -1.26 | 0.135561447 | 0.023 | -0.279 | 0.323 | 0.511 | PLEK2; FLJ33387; PIGH; ARG2; VTI1B; 389246; RDH11; RDH12; ZFYVE26; RAD51L1; ZFP36L1; ACTN1; C14orf114; GALNTL1; ERH; SLC39A9; 400224; C14orf162; 387994; KIAA0247; SFRS5 |
| 81892 | DC50 | 14 | 77244187 | 14q24.3 | gneg 8; gpos 3; | 1.82 | 7.92E-09 | -0.005 | 0.7 | 0.308 | -1.283 | C14orf59; NGB; POMT2; GSTZ1; LOC122945; C14orf133; AHSA1; THSD3; SPTLC2; ALKBH; DC50; SNW1; FLJ25976; ADCK1; FLJ14502; NRXN3; DIO2; TSHR; GTF2A1; STN2; SEL1L |
| 55775 | TDP1 | 14 | 89492047 | 14q32.11 | gneg 3; gpos 9; | 1.88 | 5.67E-05 | -0.103 | 0.605 | 0.311 | -1.11 | GPR65; KCNK10; SPATA7; PTPN21; FLJ11806; EML5; TTC8; CHES1; 400236; C14orf143; TDP1; KCNK13; PSMC1; C14orf102; CALM1; 400238; RPS6KA5; C14orf159; GPR68; KIAA2010; C14orf161 |
| 122618 | LOC122618 | 14 | 104462268 | 14q32.33 | gneg 11; gpos 2; | 1.06 | 0.252847491 | -0.075 | -0.217 | 0.324 | 0.397 | LOC374569; KIF26A; 400258; LOC339344; MGC13251; FLJ22056; 388022; ADSSL1; SIVA; AKT1; LOC122618; C14orf79; CDCA4; GPR132; JAG2; NUDT14; BRF1; BTBD6; MTA1; CRIP2; CRIP1 |
| 57722 | NOPE | 15 | 63460877 | 15q22.2 | gpos 12; | -2.07 | 0.061644547 | -0.037 | -0.342 | 0.415 | 0.626 | LOC348094; ACP33; MtFMT; OSTbeta; RIS; PDCD7; CLPX; CILP; FLJ20509; PUNC; NOPE; DPP8; HSPC121; DKFZP564O1664; SLC24A1; IRLB; RAB11A; MEGF11; MGC4562; FLJ20516; MAP2K1 |
| 9187 | SLC24A1 | 15 | 63701322 | 15q22 | gpos 11; | 1.32 | 0.204722505 | -0.061 | 0.239 | 0.469 | -0.439 | RIS; PDCD7; CLPX; CILP; FLJ20509; PUNC; NOPE; DPP8; HSPC121; DKFZP564O1664; SLC24A1; IRLB; RAB11A; MEGF11; MGC4562; FLJ20516; MAP2K1; SNAPC5; RPL4; FLJ10036; MADH6 |
| 10260 | IRLB | 15 | 63740595 | 15q22.2 | gpos 11; | -1.89 | 0.039451485 | -0.104 | -0.372 | 0.374 | 0.681 | PDCD7; CLPX; CILP; FLJ20509; PUNC; NOPE; DPP8; HSPC121; DKFZP564O1664; SLC24A1; IRLB; RAB11A; MEGF11; MGC4562; FLJ20516; MAP2K1; SNAPC5; RPL4; FLJ10036; MADH6; MADH3 |
| 8766 | RAB11A | 15 | 63948849 | 15q21.3-q22.31 | gneg 1; gpos 10; | 1.43 | 0.144639016 | -0.035 | 0.273 | 0.541 | -0.5 | CLPX; CILP; FLJ20509; PUNC; NOPE; DPP8; HSPC121; DKFZP564O1664; SLC24A1; IRLB; RAB11A; MEGF11; MGC4562; FLJ20516; MAP2K1; SNAPC5; RPL4; FLJ10036; MADH6; MADH3; DKFZP586N0721 |
| 5604 | MAP2K1 | 15 | 64466678 | 15q22.1-q22.33 | gneg 5; gpos 9; | 1.89 | 0.000373652 | -0.122 | 0.563 | 0.383 | -1.032 | NOPE; DPP8; HSPC121; DKFZP564O1664; SLC24A1; IRLB; RAB11A; MEGF11; MGC4562; FLJ20516; MAP2K1; SNAPC5; RPL4; FLJ10036; MADH6; MADH3; DKFZP586N0721; FLJ11506; FLJ12476; 145853; MAP2K5 |
| 65990 | MGC2494 | 16 | 711158 | 16p13.3 | gneg 20; | -1.05 | 0.025937274 | 0.107 | -0.397 | 0.324 | 0.728 | MGC15416; KIAA1924; LOC197336; ARHT2; RHBDL1; STUB1; 339123; DKFZp434F054; MGC33974; MGC2601; MGC2494; FLJ34512; HAGHL; NARFL; MSLN; C16orf40; CHTF18; GNG13; 388199; FLJ12681; SOX8 |
| 64788 | FLJ12681 | 16 | 843638 | 16p13.3 | gneg 20; | 1.25 | 0.824871138 | 0.157 | -0.043 | 0.32 | 0.079 | MGC2601; MGC2494; FLJ34512; HAGHL; NARFL; MSLN; C16orf40; CHTF18; GNG13; 388199; FLJ12681; SOX8; FLJ32252; SSTR5; CACNA1H; TPSG1; TPS1; TPSB1; TPSB2; TPSD1; UBE2I |
| 9894 | KIAA0683 | 16 | 1483422 | 16p13.3 | gneg 20; | 1.62 | 0.03913774 | 0.156 | 0.372 | 0.301 | -0.682 | TPSB1; TPSB2; TPSD1; UBE2I; BAIAP3; CAB56184; FLJ23360; UNKL; LOC283951; CLCN7; KIAA0683; KIAA0590; FLJ20898; C16orf34; MAPK8IP3; NME3; MRPS34; SSB3; NUBP2; IGFALS; HAGH |
| 65993 | MRPS34 | 16 | 1761897 | 16p13.3 | gneg 20; | 1.49 | 0.194857565 | 0.139 | 0.244 | 0.315 | -0.448 | FLJ23360; UNKL; LOC283951; CLCN7; KIAA0683; KIAA0590; FLJ20898; C16orf34; MAPK8IP3; NME3; MRPS34; SSB3; NUBP2; IGFALS; HAGH; DKFZP566J2046; MGC35212; 342460; SEPX1; RPL3L; NDUFB10 |
| 9074 | CLDN6 | 16 | 3004714 | 16p13.3 | gneg 19; | 1.88 | 0.01515513 | 0.053 | 0.427 | 0.34 | -0.782 | LOC124220; MGC52282; PRSS22; LOC114984; KREMEN2; FLJ30002; PKMYT1; 388205; 388206; CLDN9; CLDN6; TNFRSF12A; HCFC1R1; 146439; MGC2655; MMP25; MMPL1; NK4; ZNF206; ZNF205; ZNF213 |
| 84309 | SDOS | 16 | 4683716 | 16p13.3 | gneg 21; | 1.18 | 0.788982032 | 0.072 | -0.052 | 0.306 | 0.095 | Magmas; FLJ22021; KIAA0542; LOC114990; DNAJA3; HSCARG; HMOX2; C16orf5; LOC124402; MGRN1; SDOS; KIAA1977; LOC146562; FLJ25410; FLJ22386; N-PAC; UBN1; PPL; KIAA0420; NAGPA; MGC45438 |
| 55623 | FLJ20274 | 16 | 20654214 | 16p13.11 | gneg 14; gpos 1; | 1.4 | 0.048523833 | 0.076 | 0.358 | 0.381 | -0.657 | MGC16824; MGC35048; GPRC5B; 124274; GP2; UMOD; LOC204474; FLJ20581; LOC348158; BUCS1; FLJ20274; SAH; MGC16943; LOC81691; MGC48972; LOC57149; DNAH3; LOC57146; ZP2; FLJ38819; CRYM |
| 81691 | LOC81691 | 16 | 20725333 | 16p13.11 | gneg 12; gpos 1; | 1.22 | 0.547225611 | 0.075 | -0.116 | 0.38 | 0.213 | 124274; GP2; UMOD; LOC204474; FLJ20581; LOC348158; BUCS1; FLJ20274; SAH; MGC16943; LOC81691; MGC48972; LOC57149; DNAH3; LOC57146; ZP2; FLJ38819; CRYM; IMAA; DREV1; IGSF6 |
| 57149 | LOC57149 | 16 | 20819926 | 16p11.2 | gneg 12; gpos 1; | -1.24 | 0.021493474 | 0.099 | -0.408 | 0.353 | 0.748 | UMOD; LOC204474; FLJ20581; LOC348158; BUCS1; FLJ20274; SAH; MGC16943; LOC81691; MGC48972; LOC57149; DNAH3; LOC57146; ZP2; FLJ38819; CRYM; IMAA; DREV1; IGSF6; OTOA; 388238 |
| 27327 | TNRC6 | 16 | 24649072 | 16p11.2 | gneg 8; gpos 5; | 1.79 | 0.005817643 | 0.064 | 0.471 | 0.317 | -0.864 | GGA2; UBPH; NDUFAB1; FLJ21816; MGC3248; PLK; LOC63928; PRKCB1; CACNG3; RBBP6; TNRC6; KST1; RICH1; 388227; LCMT1; AQP8; 342357; HS3ST4; TNT; FLJ13798; 400512 |
| 25970 | SH2B | 16 | 28782814 | 16p12.1 | gneg 5; gpos 13; | 1.11 | 0.370072779 | 0.08 | -0.171 | 0.356 | 0.314 | EIF3S8; CLN3; 400514; IL27; P8; LOC112869; SULT1A2; SULT1A1; A2LP; TUFM; SH2B; ATP2A1; FRA; CD19; FLJ14639; SPINL; LAT; MGC5178; SULT1A3; QPRT; FLJ35681 |
| 146547 | FLJ90661 | 16 | 31057749 | 16p11.2 | gneg 16; | 1.28 | 0.463505302 | 0.008 | -0.141 | 0.327 | 0.258 | MGC13024; HSD3B7; STX1B2; STX4A; FLJ13479; KIAA0296; IMAGE3455200; BCKDK; MYST1; PRSS8; FLJ90661; FUS; ASC; PYC1; ITGAM; ITGAX; COX6A2; MGC46336; FLJ13063; TGFB1I1; SLC5A2 |
| 4498 | MT1J | 16 | 55227151 | 16q13 | gneg 17; gpos 3; | 1.92 | 0.368470148 | 0.112 | 0.172 | 0.306 | -0.315 | GNAO1; AMFR; CPSF5; FLJ10826; BBS2; MT4; MT3; MT2A; MT1E; MT1K; MT1J; MT1A; MT1B; MT1F; MT1G; MT1H; MT1X; KIAA0095; SLC12A3; HERPUD1; CETP |
| 4494 | MT1F | 16 | 55249355 | 16q13 | gneg 18; gpos 2; | 2.77 | 0.014542897 | 0.094 | 0.429 | 0.345 | -0.786 | FLJ10826; BBS2; MT4; MT3; MT2A; MT1E; MT1K; MT1J; MT1A; MT1B; MT1F; MT1G; MT1H; MT1X; KIAA0095; SLC12A3; HERPUD1; CETP; NOD27; CPNE2; NIP30 |
| 4495 | MT1G | 16 | 55258153 | 16q13 | gneg 18; gpos 2; | 4.56 | 0.000808197 | 0.068 | 0.542 | 0.332 | -0.993 | BBS2; MT4; MT3; MT2A; MT1E; MT1K; MT1J; MT1A; MT1B; MT1F; MT1G; MT1H; MT1X; KIAA0095; SLC12A3; HERPUD1; CETP; NOD27; CPNE2; NIP30; KIAA1972 |
| 4496 | MT1H | 16 | 55261274 | 16q13 | gneg 19; gpos 1; | 2.49 | 0.025377353 | 0.133 | 0.399 | 0.326 | -0.731 | MT4; MT3; MT2A; MT1E; MT1K; MT1J; MT1A; MT1B; MT1F; MT1G; MT1H; MT1X; KIAA0095; SLC12A3; HERPUD1; CETP; NOD27; CPNE2; NIP30; KIAA1972; BART1 |
| 26189 | OR1A2 | 17 | 3047562 | 17p13.3 | gneg 20; | 2 | 0.32849184 | -0.025 | 0.186 | 0.39 | -0.342 | FLJ10534; KIAA0397; MNT; MGC3329; PAFAH1B1; KIAA0664; KIAA1039; OR1D5; OR1D2; OR1G1; OR1A2; OR1A1; OR3A2; OR3A1; OR1E1; OR3A3; OR1E2; NYD-SP20; ASPA; TRPV3; TRPV1 |
| 8383 | OR1A1 | 17 | 3065664 | 17p13.3 | gneg 19; | 1.8 | 0.404158327 | -0.002 | 0.16 | 0.374 | -0.293 | KIAA0397; MNT; MGC3329; PAFAH1B1; KIAA0664; KIAA1039; OR1D5; OR1D2; OR1G1; OR1A2; OR1A1; OR3A2; OR3A1; OR1E1; OR3A3; OR1E2; NYD-SP20; ASPA; TRPV3; TRPV1; CARKL |
| 4994 | OR3A1 | 17 | 3141678 | 17p13.3 | gneg 17; | -1 | 0.183844701 | 0 | -0.25 | 0.341 | 0.458 | MGC3329; PAFAH1B1; KIAA0664; KIAA1039; OR1D5; OR1D2; OR1G1; OR1A2; OR1A1; OR3A2; OR3A1; OR1E1; OR3A3; OR1E2; NYD-SP20; ASPA; TRPV3; TRPV1; CARKL; CTNS; TIP-1 |
| 54785 | FLJ20014 | 17 | 8032376 | 17p13.1 | gneg 15; gpos 2; | -1.45 | 0.003773709 | 0.093 | -0.489 | 0.424 | 0.897 | TRAPPC1; LIP8; GUCY2D; ALOX15B; ALOX12B; ALOXE3; HES7; PER1; VAMP2; MGC10744; FLJ20014; AURKB; FLJ34790; FLJ22170; PFAS; 399512; ARHGEF15; ODF4; RPL26; NDEL1; MYH10 |
| 9212 | AURKB | 17 | 8048781 | 17p13.1 | gneg 16; gpos 1; | 1.69 | 0.003271023 | 0.238 | 0.495 | 0.36 | -0.907 | LIP8; GUCY2D; ALOX15B; ALOX12B; ALOXE3; HES7; PER1; VAMP2; MGC10744; FLJ20014; AURKB; FLJ34790; FLJ22170; PFAS; 399512; ARHGEF15; ODF4; RPL26; NDEL1; MYH10; FLJ32734 |
| 284029 | FLJ34790 | 17 | 8064691 | 17p13.1 | gneg 17; | -3.48 | 0.04980813 | 0.017 | -0.356 | 0.43 | 0.653 | GUCY2D; ALOX15B; ALOX12B; ALOXE3; HES7; PER1; VAMP2; MGC10744; FLJ20014; AURKB; FLJ34790; FLJ22170; PFAS; 399512; ARHGEF15; ODF4; RPL26; NDEL1; MYH10; FLJ32734; FLJ35773 |
| 5198 | PFAS | 17 | 8093361 | 17p13.1 | gneg 16; | 2.94 | 0.001255383 | 0.188 | 0.528 | 0.318 | -0.968 | ALOX12B; ALOXE3; HES7; PER1; VAMP2; MGC10744; FLJ20014; AURKB; FLJ34790; FLJ22170; PFAS; 399512; ARHGEF15; ODF4; RPL26; NDEL1; MYH10; FLJ32734; FLJ35773; P101-PI3K; NTN1 |
| 4626 | MYH8 | 17 | 10234366 | 17p13.1 | gneg 15; gpos 2; | -1.56 | 0.666009757 | -0.093 | -0.083 | 0.329 | 0.153 | FLJ35773; P101-PI3K; NTN1; STX8; LOC146845; 201140; GLP2R; RCV1; GAS7; MYH13; MYH8; MYH4; MYH1; MYH2; MYH3; SCO1; MDS006; LOC284033; DNAH9; MAP2K4; MYCD |
| 6341 | SCO1 | 17 | 10524379 | 17p12-p13 | gneg 13; gpos 2; | 1.79 | 0.007076767 | 0.105 | 0.463 | 0.346 | -0.849 | 201140; GLP2R; RCV1; GAS7; MYH13; MYH8; MYH4; MYH1; MYH2; MYH3; SCO1; MDS006; LOC284033; DNAH9; MAP2K4; MYCD; KIAA0672; ELAC2; HS3ST3A1; 400574; COX10 |
| 9618 | TRAF4 | 17 | 24095172 | 17q11-q12 | acen 1; gneg 13; gpos 1; | -1.23 | 0.192478215 | -0.034 | -0.245 | 0.316 | 0.45 | PIGS; ALDOC; SPAG5; FLJ25006; SDF2; MGC39650; RAB34; RPL23A; LOC116238; NEK8; TRAF4; FLJ10700; ERAL1; FLOT2; MGC23280; PHF12; SEZ6; PIPOX; 399687; TIAF1; CRYBA1 |
| 26284 | ERAL1 | 17 | 24206168 | 17q11.2 | acen 1; gneg 15; | 1.35 | 0.240524408 | 0.192 | 0.222 | 0.463 | -0.407 | SPAG5; FLJ25006; SDF2; MGC39650; RAB34; RPL23A; LOC116238; NEK8; TRAF4; FLJ10700; ERAL1; FLOT2; MGC23280; PHF12; SEZ6; PIPOX; 399687; TIAF1; CRYBA1; 182-FIP; KIAA1361 |
| 2319 | FLOT2 | 17 | 24230483 | 17q11-q12 | acen 1; gneg 15; | 1.07 | 0.325985776 | 0.073 | -0.187 | 0.331 | 0.343 | FLJ25006; SDF2; MGC39650; RAB34; RPL23A; LOC116238; NEK8; TRAF4; FLJ10700; ERAL1; FLOT2; MGC23280; PHF12; SEZ6; PIPOX; 399687; TIAF1; CRYBA1; 182-FIP; KIAA1361; LOC116236 |
| 147015 | MGC23280 | 17 | 24248927 | 17q11.2 | acen 1; gneg 15; | 1.18 | 0.801768148 | 0.099 | 0.049 | 0.305 | -0.089 | SDF2; MGC39650; RAB34; RPL23A; LOC116238; NEK8; TRAF4; FLJ10700; ERAL1; FLOT2; MGC23280; PHF12; SEZ6; PIPOX; 399687; TIAF1; CRYBA1; 182-FIP; KIAA1361; LOC116236; GIT1 |
| 124925 | SEZ6 | 17 | 24306073 | 17q11.2 | acen 1; gneg 15; | 1.17 | 0.284615981 | -0.12 | -0.203 | 0.414 | 0.373 | RAB34; RPL23A; LOC116238; NEK8; TRAF4; FLJ10700; ERAL1; FLOT2; MGC23280; PHF12; SEZ6; PIPOX; 399687; TIAF1; CRYBA1; 182-FIP; KIAA1361; LOC116236; GIT1; FLJ25555; FLJ14871 |
| 51268 | PIPOX | 17 | 24394326 | 17q11.2 | acen 1; gneg 15; | 5.25 | 0.00025992 | 0.087 | 0.572 | 0.479 | -1.049 | RPL23A; LOC116238; NEK8; TRAF4; FLJ10700; ERAL1; FLOT2; MGC23280; PHF12; SEZ6; PIPOX; 399687; TIAF1; CRYBA1; 182-FIP; KIAA1361; LOC116236; GIT1; FLJ25555; FLJ14871; SSH2 |
| 57532 | 182-FIP | 17 | 24612771 | 17q11.2 | gneg 17; | 1.4 | 0.773615936 | -0.013 | -0.056 | 0.491 | 0.102 | FLJ10700; ERAL1; FLOT2; MGC23280; PHF12; SEZ6; PIPOX; 399687; TIAF1; CRYBA1; 182-FIP; KIAA1361; LOC116236; GIT1; FLJ25555; FLJ14871; SSH2; FLJ46247; DKFZP434K1421; SLC6A4; BLMH |
| 57551 | KIAA1361 | 17 | 24742068 | 17q11.2 | gneg 16; | -1.16 | 0.178808548 | 0.131 | -0.253 | 0.468 | 0.463 | ERAL1; FLOT2; MGC23280; PHF12; SEZ6; PIPOX; 399687; TIAF1; CRYBA1; 182-FIP; KIAA1361; LOC116236; GIT1; FLJ25555; FLJ14871; SSH2; FLJ46247; DKFZP434K1421; SLC6A4; BLMH; 388364 |
| 54475 | FLJ10458 | 17 | 30482482 | 17q21.1 | gneg 16; gpos 1; | 1.96 | 0.017154794 | -0.081 | 0.42 | 0.332 | -0.77 | CCL11; CCL8; CCL13; CCL1; CCT6B; MGC20398; LIG3; LOC117584; RAD51L3; DKFZp434H2215; FLJ10458; CMYA4; FLJ40154; MGC19764; FLJ34922; FLJ10260; FLJ31952; PEX12; AP2B1; VTS58635; GAR17 |
| 162394 | MGC19764 | 17 | 30594204 | 17q21.1 | gneg 15; gpos 1; | -1.83 | 0.003003142 | 0.098 | -0.498 | 0.302 | 0.913 | CCL1; CCT6B; MGC20398; LIG3; LOC117584; RAD51L3; DKFZp434H2215; FLJ10458; CMYA4; FLJ40154; MGC19764; FLJ34922; FLJ10260; FLJ31952; PEX12; AP2B1; VTS58635; GAR17; LOC146853; MMP28; TAF15 |
| 11056 | DDX52 | 17 | 33046525 | 17q21.1 | gneg 9; gpos 7; | 1.19 | 0.678592148 | 0.036 | -0.08 | 0.334 | 0.147 | LZK1; MGC4172; FLJ22578; LHX1; AATF; ACACA; FLJ39647; TADA2L; DUSP14; AP1GBP1; DDX52; TCF2; LOC284100; TBC1D3; MRPL45; NAP4; MLLT6; LOC139231; RNF110; PSMB3; PIP5K2B |
| 85280 | KRTAP9-4 | 17 | 36659464 | 17q12-q21 |  | 1.17 | 0.419047369 | 0.088 | -0.155 | 0.377 | 0.284 | KRTAP1-1; KRTAP2-4; KRTAP4-14; KRTAP4-12; KRTAP4-5; KRTAP4-4; KRTAP4-2; KRTAP4-10; KRTAP9-2; KRTAP9-3; KRTAP9-4; KRTAP17-1; KRTHA3A; KRTHA3B; KRTHA4; KRTHA1; KRTHA7; KRTHA8; KRTHA2; KRTHA5; KRTHA6 |
| 83902 | KRTAP17-1 | 17 | 36724696 | 17q12-q21 |  | -1.56 | 0.331768606 | -0.075 | -0.185 | 0.338 | 0.339 | KRTAP2-4; KRTAP4-14; KRTAP4-12; KRTAP4-5; KRTAP4-4; KRTAP4-2; KRTAP4-10; KRTAP9-2; KRTAP9-3; KRTAP9-4; KRTAP17-1; KRTHA3A; KRTHA3B; KRTHA4; KRTHA1; KRTHA7; KRTHA8; KRTHA2; KRTHA5; KRTHA6; KRT13 |
| 3883 | KRTHA3A | 17 | 36755896 | 17q12-q21 | gpos 1; | 4.92 | 0.173010365 | 0.045 | 0.256 | 0.373 | -0.469 | KRTAP4-14; KRTAP4-12; KRTAP4-5; KRTAP4-4; KRTAP4-2; KRTAP4-10; KRTAP9-2; KRTAP9-3; KRTAP9-4; KRTAP17-1; KRTHA3A; KRTHA3B; KRTHA4; KRTHA1; KRTHA7; KRTHA8; KRTHA2; KRTHA5; KRTHA6; KRT13; KRT15 |
| 8687 | KRTHA8 | 17 | 36846146 | 17q12-q21 | gpos 2; | 1.23 | 0.609916624 | 0.305 | -0.098 | 0.482 | 0.181 | KRTAP4-10; KRTAP9-2; KRTAP9-3; KRTAP9-4; KRTAP17-1; KRTHA3A; KRTHA3B; KRTHA4; KRTHA1; KRTHA7; KRTHA8; KRTHA2; KRTHA5; KRTHA6; KRT13; KRT15; KRT19; KRT9; KRT14; KRT16; KRT17 |
| 3886 | KRTHA5 | 17 | 36886466 | 17q12-q21 | gneg 1; gpos 2; | 1.39 | 0.754184905 | 0.318 | 0.061 | 0.462 | -0.111 | KRTAP9-3; KRTAP9-4; KRTAP17-1; KRTHA3A; KRTHA3B; KRTHA4; KRTHA1; KRTHA7; KRTHA8; KRTHA2; KRTHA5; KRTHA6; KRT13; KRT15; KRT19; KRT9; KRT14; KRT16; KRT17; SUI1; GAS |
| 3860 | KRT13 | 17 | 36910759 | 17q12-q21.2 | gneg 1; gpos 2; | 3.1 | 0.509575992 | 0.189 | 0.127 | 0.363 | -0.233 | KRTAP17-1; KRTHA3A; KRTHA3B; KRTHA4; KRTHA1; KRTHA7; KRTHA8; KRTHA2; KRTHA5; KRTHA6; KRT13; KRT15; KRT19; KRT9; KRT14; KRT16; KRT17; SUI1; GAS; HAP1; JUP |
| 3866 | KRT15 | 17 | 36923523 | 17q21.2 | gneg 2; gpos 2; | 1.23 | 0.872795231 | 0.326 | -0.031 | 0.394 | 0.057 | KRTHA3A; KRTHA3B; KRTHA4; KRTHA1; KRTHA7; KRTHA8; KRTHA2; KRTHA5; KRTHA6; KRT13; KRT15; KRT19; KRT9; KRT14; KRT16; KRT17; SUI1; GAS; HAP1; JUP; SC65 |
| 3861 | KRT14 | 17 | 36992058 | 17q12-q21 | gneg 4; gpos 3; | 1.27 | 0.552449467 | 0.354 | 0.114 | 0.382 | -0.21 | KRTHA1; KRTHA7; KRTHA8; KRTHA2; KRTHA5; KRTHA6; KRT13; KRT15; KRT19; KRT9; KRT14; KRT16; KRT17; SUI1; GAS; HAP1; JUP; SC65; FKBP10; MGC20781; KLHL10 |
| 3872 | KRT17 | 17 | 37029220 | 17q12-q21 | gneg 5; gpos 3; | -1.43 | 0.007012092 | 0.181 | -0.463 | 0.349 | 0.85 | KRTHA8; KRTHA2; KRTHA5; KRTHA6; KRT13; KRT15; KRT19; KRT9; KRT14; KRT16; KRT17; SUI1; GAS; HAP1; JUP; SC65; FKBP10; MGC20781; KLHL10; FLJ10572; ACLY |
| 3212 | HOXB2 | 17 | 43975019 | 17q21-q22 | gneg 1; gpos 8; | -3.15 | 0.016215973 | 0.083 | -0.423 | 0.397 | 0.776 | SP2; PNPO; MGC11242; CDK5RAP3; COPZ2; NFE2L1; CBX1; SNX11; SCAP1; HOXB1; HOXB2; HOXB3; HOXB4; HOXB5; HOXB6; HOXB7; HOXB8; HOXB9; PRAC; HOXB13; FLJ35808 |
| 2793 | GNGT2 | 17 | 44638595 | 17q21 | gneg 12; gpos 2; | 1.77 | 0.654232155 | 0.073 | -0.087 | 0.308 | 0.159 | HOXB9; PRAC; HOXB13; FLJ35808; ATP5G1; FLJ13855; EAP30; GIP; IMP-1; GALGT2; GNGT2; NESH; PHOSPHO1; 124871; PHB; NGFR; NXPH3; SPOP; SLC35B1; LOC81558; MYST2 |
| 5245 | PHB | 17 | 44836418 | 17q21 | gneg 14; gpos 1; | 1.88 | 0.009886658 | 0.096 | 0.448 | 0.352 | -0.821 | ATP5G1; FLJ13855; EAP30; GIP; IMP-1; GALGT2; GNGT2; NESH; PHOSPHO1; 124871; PHB; NGFR; NXPH3; SPOP; SLC35B1; LOC81558; MYST2; TAC4; DLX4; DLX3; ITGA3 |
| 3396 | ICT1 | 17 | 70520374 | 17q25.2 | gpos 16; | 1.64 | 0.089518701 | 0.107 | 0.313 | 0.38 | -0.575 | DKFZP564C103; FLJ20255; GRIN2C; FDXR; LOC283985; USH1G; OTOP2; OTOP3; C17orf28; HUMPPA; ICT1; ATP5H; SLC16A5; FLJ22160; NT5C; HN1; SMT3H2; PCNT1; GGA3; MRPS7; AD023 |
| 79637 | FLJ22160 | 17 | 70617676 | 17q25.2 | gneg 2; gpos 14; | 1.14 | 0.473228004 | 0.194 | -0.138 | 0.323 | 0.253 | FDXR; LOC283985; USH1G; OTOP2; OTOP3; C17orf28; HUMPPA; ICT1; ATP5H; SLC16A5; FLJ22160; NT5C; HN1; SMT3H2; PCNT1; GGA3; MRPS7; AD023; SLC25A19; GRB2; KIAA0195 |
| 30833 | NT5C | 17 | 70637918 | 17q25.2 | gneg 3; gpos 14; | 1.41 | 0.48423558 | 0.122 | 0.134 | 0.352 | -0.247 | LOC283985; USH1G; OTOP2; OTOP3; C17orf28; HUMPPA; ICT1; ATP5H; SLC16A5; FLJ22160; NT5C; HN1; SMT3H2; PCNT1; GGA3; MRPS7; AD023; SLC25A19; GRB2; KIAA0195; CASKIN2 |
| 51155 | HN1 | 17 | 70642937 | 17q25.2 | gneg 3; gpos 13; | 1.5 | 0.010797664 | 0.231 | 0.444 | 0.31 | -0.813 | USH1G; OTOP2; OTOP3; C17orf28; HUMPPA; ICT1; ATP5H; SLC16A5; FLJ22160; NT5C; HN1; SMT3H2; PCNT1; GGA3; MRPS7; AD023; SLC25A19; GRB2; KIAA0195; CASKIN2; LLGL2 |
| 79902 | PCNT1 | 17 | 70713380 | 17q25.2 | gneg 4; gpos 11; | 1.9 | 0.000221782 | 0.102 | 0.576 | 0.349 | -1.056 | OTOP3; C17orf28; HUMPPA; ICT1; ATP5H; SLC16A5; FLJ22160; NT5C; HN1; SMT3H2; PCNT1; GGA3; MRPS7; AD023; SLC25A19; GRB2; KIAA0195; CASKIN2; LLGL2; RECQL5; HCNGP |
| 57513 | CASKIN2 | 17 | 71007936 | 17q25.3 | gneg 6; gpos 5; | 1.95 | 0.528396572 | 0.006 | 0.121 | 0.356 | -0.223 | NT5C; HN1; SMT3H2; PCNT1; GGA3; MRPS7; AD023; SLC25A19; GRB2; KIAA0195; CASKIN2; LLGL2; RECQL5; HCNGP; ITGB4; GALK1; H3F3B; WBP2; TRIM47; LOC201292; MRPL38 |
| 9400 | RECQL5 | 17 | 71134519 | 17q25.2-q25.3 | gneg 6; gpos 3; | 1.37 | 0.176067463 | 0.014 | 0.254 | 0.314 | -0.466 | SMT3H2; PCNT1; GGA3; MRPS7; AD023; SLC25A19; GRB2; KIAA0195; CASKIN2; LLGL2; RECQL5; HCNGP; ITGB4; GALK1; H3F3B; WBP2; TRIM47; LOC201292; MRPL38; ACOX1; CDK3 |
| 29115 | HCNGP | 17 | 71174993 | 17q25.3 | gneg 6; gpos 3; | 1 | 0.204100108 | 0.164 | -0.24 | 0.388 | 0.439 | PCNT1; GGA3; MRPS7; AD023; SLC25A19; GRB2; KIAA0195; CASKIN2; LLGL2; RECQL5; HCNGP; ITGB4; GALK1; H3F3B; WBP2; TRIM47; LOC201292; MRPL38; ACOX1; CDK3; EVPL |
| 3021 | H3F3B | 17 | 71284111 | 17q25 | gneg 9; gpos 1; | 1.15 | 0.459818288 | -0.221 | -0.142 | 0.369 | 0.26 | AD023; SLC25A19; GRB2; KIAA0195; CASKIN2; LLGL2; RECQL5; HCNGP; ITGB4; GALK1; H3F3B; WBP2; TRIM47; LOC201292; MRPL38; ACOX1; CDK3; EVPL; SRP68; GALR2; LGICZ |
| 201292 | LOC201292 | 17 | 71396637 | 17q25.3 | gneg 10; | 1.83 | 0.29653469 | 0.206 | 0.199 | 0.376 | -0.364 | KIAA0195; CASKIN2; LLGL2; RECQL5; HCNGP; ITGB4; GALK1; H3F3B; WBP2; TRIM47; LOC201292; MRPL38; ACOX1; CDK3; EVPL; SRP68; GALR2; LGICZ; EXOC7; FOXJ1; MGC29814 |
| 64978 | MRPL38 | 17 | 71406318 | 17q25.3 | gneg 10; | 1.38 | 0.618933246 | 0.096 | 0.096 | 0.454 | -0.176 | CASKIN2; LLGL2; RECQL5; HCNGP; ITGB4; GALK1; H3F3B; WBP2; TRIM47; LOC201292; MRPL38; ACOX1; CDK3; EVPL; SRP68; GALR2; LGICZ; EXOC7; FOXJ1; MGC29814; DKFZP434P0316 |
| 8811 | GALR2 | 17 | 71582486 | 17q25.3 | gneg 10; gpos 1; | 3.92 | 0.040784505 | 0.083 | 0.37 | 0.37 | -0.678 | GALK1; H3F3B; WBP2; TRIM47; LOC201292; MRPL38; ACOX1; CDK3; EVPL; SRP68; GALR2; LGICZ; EXOC7; FOXJ1; MGC29814; DKFZP434P0316; PRPSAP1; SPHK1; E2-230K; AANAT; FLJ22341 |
| 353174 | LGICZ | 17 | 71586903 | 17q25.3 | gneg 11; gpos 1; | 1.79 | 0.226320761 | -0.126 | 0.229 | 0.329 | -0.419 | H3F3B; WBP2; TRIM47; LOC201292; MRPL38; ACOX1; CDK3; EVPL; SRP68; GALR2; LGICZ; EXOC7; FOXJ1; MGC29814; DKFZP434P0316; PRPSAP1; SPHK1; E2-230K; AANAT; FLJ22341; CYGB |
| 283991 | MGC29814 | 17 | 71773042 | 17q25.3 | gneg 13; gpos 1; | 1.01 | 0.117347175 | -0.036 | -0.291 | 0.321 | 0.534 | LOC201292; MRPL38; ACOX1; CDK3; EVPL; SRP68; GALR2; LGICZ; EXOC7; FOXJ1; MGC29814; DKFZP434P0316; PRPSAP1; SPHK1; E2-230K; AANAT; FLJ22341; CYGB; 400621; SIAT7B; SIAT7A |
| 84074 | DKFZP434P0316 | 17 | 71781726 | 17q25.3 | gneg 12; gpos 1; | 1.65 | 0.898805574 | 0.02 | -0.025 | 0.324 | 0.045 | MRPL38; ACOX1; CDK3; EVPL; SRP68; GALR2; LGICZ; EXOC7; FOXJ1; MGC29814; DKFZP434P0316; PRPSAP1; SPHK1; E2-230K; AANAT; FLJ22341; CYGB; 400621; SIAT7B; SIAT7A; PTDSR |
| 79651 | FLJ22341 | 17 | 71978745 | 17q25.3 | gneg 13; gpos 1; | 1.47 | 0.308203078 | -0.149 | 0.194 | 0.317 | -0.356 | GALR2; LGICZ; EXOC7; FOXJ1; MGC29814; DKFZP434P0316; PRPSAP1; SPHK1; E2-230K; AANAT; FLJ22341; CYGB; 400621; SIAT7B; SIAT7A; PTDSR; SFRS2; ET; LOC283994; GnT-IX; SEC14L1 |
| 284186 | FLJ38792 | 17 | 76899668 | 17q25.3 | gneg 13; | -1.02 | 0.153086453 | -0.057 | -0.268 | 0.336 | 0.49 | KIAA1554; FLJ35220; NPTX1; raptor; FLJ11749; BAIAP2; AZI1; FLJ31528; MGC15523; FLJ39421; FLJ38792; ACTG1; FSCN2; FLJ22175; NPL4; OCSP; PDE6G; LOC339231; HGS; MRPL12; SLC25A10 |
| 55666 | NPL4 | 17 | 77134358 | 17qter | gneg 13; | -1.1 | 0.05475611 | 0.149 | -0.35 | 0.414 | 0.641 | FLJ11749; BAIAP2; AZI1; FLJ31528; MGC15523; FLJ39421; FLJ38792; ACTG1; FSCN2; FLJ22175; NPL4; OCSP; PDE6G; LOC339231; HGS; MRPL12; SLC25A10; P4HB; ARHGDIA; THOC4; ANAPC11 |
| 5148 | PDE6G | 17 | 77227654 | 17q25 | gneg 14; | 2.79 | 0.082679975 | 0.248 | 0.32 | 0.375 | -0.586 | AZI1; FLJ31528; MGC15523; FLJ39421; FLJ38792; ACTG1; FSCN2; FLJ22175; NPL4; OCSP; PDE6G; LOC339231; HGS; MRPL12; SLC25A10; P4HB; ARHGDIA; THOC4; ANAPC11; NPB; PCYT2 |
| 339231 | LOC339231 | 17 | 77258810 | 17q25.3 | gneg 13; | -1.06 | 0.172718807 | -0.213 | -0.256 | 0.443 | 0.469 | FLJ31528; MGC15523; FLJ39421; FLJ38792; ACTG1; FSCN2; FLJ22175; NPL4; OCSP; PDE6G; LOC339231; HGS; MRPL12; SLC25A10; P4HB; ARHGDIA; THOC4; ANAPC11; NPB; PCYT2; SIRT7 |
| 9146 | HGS | 17 | 77261424 | 17q25 | gneg 12; | 1.06 | 0.054037015 | 0.266 | -0.351 | 0.421 | 0.643 | MGC15523; FLJ39421; FLJ38792; ACTG1; FSCN2; FLJ22175; NPL4; OCSP; PDE6G; LOC339231; HGS; MRPL12; SLC25A10; P4HB; ARHGDIA; THOC4; ANAPC11; NPB; PCYT2; SIRT7; MAFG |
| 6182 | MRPL12 | 17 | 77280811 | 17q25 | gneg 12; | 1.12 | 0.525190954 | 0.281 | 0.122 | 0.423 | -0.224 | FLJ39421; FLJ38792; ACTG1; FSCN2; FLJ22175; NPL4; OCSP; PDE6G; LOC339231; HGS; MRPL12; SLC25A10; P4HB; ARHGDIA; THOC4; ANAPC11; NPB; PCYT2; SIRT7; MAFG; PYCR1 |
| 1468 | SLC25A10 | 17 | 77289775 | 17q25.3 | gneg 12; | 1.75 | 0.191004984 | 0.35 | 0.246 | 0.404 | -0.451 | FLJ38792; ACTG1; FSCN2; FLJ22175; NPL4; OCSP; PDE6G; LOC339231; HGS; MRPL12; SLC25A10; P4HB; ARHGDIA; THOC4; ANAPC11; NPB; PCYT2; SIRT7; MAFG; PYCR1; 255275 |
| 10189 | THOC4 | 17 | 77439015 | 17q25.3 | gneg 13; | 1.71 | 0.013824292 | 0.327 | 0.431 | 0.431 | -0.791 | FLJ22175; NPL4; OCSP; PDE6G; LOC339231; HGS; MRPL12; SLC25A10; P4HB; ARHGDIA; THOC4; ANAPC11; NPB; PCYT2; SIRT7; MAFG; PYCR1; 255275; LOC147111; ASPSCR1; STRA13 |
| 51529 | ANAPC11 | 17 | 77442894 | 17q25.3 | gneg 13; | 1.13 | 0.691044794 | 0.144 | 0.077 | 0.49 | -0.141 | NPL4; OCSP; PDE6G; LOC339231; HGS; MRPL12; SLC25A10; P4HB; ARHGDIA; THOC4; ANAPC11; NPB; PCYT2; SIRT7; MAFG; PYCR1; 255275; LOC147111; ASPSCR1; STRA13; MGC20806 |
| 5833 | PCYT2 | 17 | 77455383 | 17q25.3 | gneg 14; | 1.07 | 0.325799279 | 0.307 | -0.187 | 0.546 | 0.344 | PDE6G; LOC339231; HGS; MRPL12; SLC25A10; P4HB; ARHGDIA; THOC4; ANAPC11; NPB; PCYT2; SIRT7; MAFG; PYCR1; 255275; LOC147111; ASPSCR1; STRA13; MGC20806; RAC3; DCXR |
| 51547 | SIRT7 | 17 | 77463106 | 17q25 | gneg 15; | 1.19 | 0.739598811 | -0.119 | -0.064 | 0.484 | 0.118 | LOC339231; HGS; MRPL12; SLC25A10; P4HB; ARHGDIA; THOC4; ANAPC11; NPB; PCYT2; SIRT7; MAFG; PYCR1; 255275; LOC147111; ASPSCR1; STRA13; MGC20806; RAC3; DCXR; GPS1 |
| 79058 | ASPSCR1 | 17 | 77528714 | 17q25 | gneg 16; | 1.05 | 0.273493675 | -0.208 | -0.208 | 0.343 | 0.381 | ARHGDIA; THOC4; ANAPC11; NPB; PCYT2; SIRT7; MAFG; PYCR1; 255275; LOC147111; ASPSCR1; STRA13; MGC20806; RAC3; DCXR; GPS1; PP3111; FASN; FLJ23754; LOC284001; SLC16A3 |
| 201254 | STRA13 | 17 | 77569867 | 17q25.3 | gneg 15; | 1.31 | 0.430229967 | 0.115 | 0.151 | 0.314 | -0.277 | THOC4; ANAPC11; NPB; PCYT2; SIRT7; MAFG; PYCR1; 255275; LOC147111; ASPSCR1; STRA13; MGC20806; RAC3; DCXR; GPS1; PP3111; FASN; FLJ23754; LOC284001; SLC16A3; 388434 |
| 147463 | LOC147463 | 18 | 19434007 | 18q11.2 | acen 1; gneg 16; | 2.33 | 0.836653414 | -0.07 | 0.04 | 0.318 | -0.073 | MIB; 201484; GATA6; CTAGE-1; RBBP8; CABLES1; MGC11386; RIOK3; C18orf8; NPC1; LOC147463; LAMA3; FLJ33761; CABYR; OSBPL1A; IMPACT; HRH4; 147468; EHZF; SS18; MGC26605 |
| 114876 | OSBPL1A | 18 | 19996010 | 18q11.1 | acen 1; gneg 16; gpos 1; | 1.65 | 0.476938507 | 0.062 | 0.137 | 0.388 | -0.251 | RBBP8; CABLES1; MGC11386; RIOK3; C18orf8; NPC1; LOC147463; LAMA3; FLJ33761; CABYR; OSBPL1A; IMPACT; HRH4; 147468; EHZF; SS18; MGC26605; AQP4; FLJ30507; CHST9; CDH2 |
| 340527 | not_found | 19 | 2380267 | Xq13.1 | gneg 17; | 1.67 | 0.973269537 | -0.148 | 0.006 | 0.412 | -0.012 | AMH; FLJ32416; OAZ1; FLJ45778; 388489; LSM7; SPPL2B; LOC360200; TIMM13; LMNB2; 340527; GADD45B; GNG7; DIRAS1; SLC39A3; SGTA; THOP1; FLJ34817; MGC26707; FLJ11637; LOC126295 |
| 29985 | SLC39A3 | 19 | 2683524 | 19p13.3 | gneg 17; | 1.4 | 0.556451474 | -0.002 | 0.113 | 0.325 | -0.208 | 388489; LSM7; SPPL2B; LOC360200; TIMM13; LMNB2; 340527; GADD45B; GNG7; DIRAS1; SLC39A3; SGTA; THOP1; FLJ34817; MGC26707; FLJ11637; LOC126295; ZNF77; FLJ14009; TLE2; AES |
| 4145 | MATK | 19 | 3728967 | 19p13.3 | gneg 19; | 1.39 | 0.68833511 | -0.206 | 0.078 | 0.3 | -0.142 | FZR1; MGC20700; HMG20B; GIPC3; TBXA2R; PIP5K1C; TJP3; APBA3; MRPL54; MGC15631; MATK; KIAA1086; ATCAY; ITGB1BP3; DAPK3; EEF2; PIASY; FBI1; MAP2K2; CREB3L3; SIRT6 |
| 27231 | ITGB1BP3 | 19 | 3884100 | 19p13.3 | gneg 19; | 2.71 | 0.273724885 | 0.048 | 0.208 | 0.359 | -0.381 | GIPC3; TBXA2R; PIP5K1C; TJP3; APBA3; MRPL54; MGC15631; MATK; KIAA1086; ATCAY; ITGB1BP3; DAPK3; EEF2; PIASY; FBI1; MAP2K2; CREB3L3; SIRT6; KIAA1981; EBI3; FLJ10374 |
| 51341 | FBI1 | 19 | 3996216 | 19p13.3 | gneg 19; | -1 | 0.217275579 | 0.079 | -0.233 | 0.379 | 0.427 | APBA3; MRPL54; MGC15631; MATK; KIAA1086; ATCAY; ITGB1BP3; DAPK3; EEF2; PIASY; FBI1; MAP2K2; CREB3L3; SIRT6; KIAA1981; EBI3; FLJ10374; SHD; MGC23244; FSD1; STAP2 |
| 51548 | SIRT6 | 19 | 4125105 | 19p13.3 | gneg 19; | 1.2 | 0.762427213 | 0.312 | -0.059 | 0.35 | 0.107 | MATK; KIAA1086; ATCAY; ITGB1BP3; DAPK3; EEF2; PIASY; FBI1; MAP2K2; CREB3L3; SIRT6; KIAA1981; EBI3; FLJ10374; SHD; MGC23244; FSD1; STAP2; FLJ14981; SH3GL1; CHAF1A |
| 170961 | KIAA1981 | 19 | 4163657 | 19p13.3 | gneg 18; | 3.49 | 0.005391786 | 0.337 | 0.475 | 0.341 | -0.87 | KIAA1086; ATCAY; ITGB1BP3; DAPK3; EEF2; PIASY; FBI1; MAP2K2; CREB3L3; SIRT6; KIAA1981; EBI3; FLJ10374; SHD; MGC23244; FSD1; STAP2; FLJ14981; SH3GL1; CHAF1A; UBXD1 |
| 55702 | FLJ10374 | 19 | 4198107 | 19p13.3 | gneg 18; | 1.35 | 0.928086366 | 0.145 | -0.017 | 0.346 | 0.032 | ITGB1BP3; DAPK3; EEF2; PIASY; FBI1; MAP2K2; CREB3L3; SIRT6; KIAA1981; EBI3; FLJ10374; SHD; MGC23244; FSD1; STAP2; FLJ14981; SH3GL1; CHAF1A; UBXD1; MGC2641; LRG |
| 79187 | FSD1 | 19 | 4255690 | 19p13.3 | gneg 19; | 1.79 | 0.115562346 | 0.205 | 0.292 | 0.339 | -0.536 | PIASY; FBI1; MAP2K2; CREB3L3; SIRT6; KIAA1981; EBI3; FLJ10374; SHD; MGC23244; FSD1; STAP2; FLJ14981; SH3GL1; CHAF1A; UBXD1; MGC2641; LRG; SEMA6B; MGC17791; C19orf10 |
| 55620 | STAP2 | 19 | 4275040 | 19p13.3 | gneg 19; | -1.11 | 0.129541629 | 0.103 | -0.283 | 0.312 | 0.518 | FBI1; MAP2K2; CREB3L3; SIRT6; KIAA1981; EBI3; FLJ10374; SHD; MGC23244; FSD1; STAP2; FLJ14981; SH3GL1; CHAF1A; UBXD1; MGC2641; LRG; SEMA6B; MGC17791; C19orf10; DPP9 |
| 80700 | UBXD1 | 19 | 4396260 | 19p13 | gneg 20; | -1.29 | 0.002850419 | -0.102 | -0.5 | 0.334 | 0.916 | KIAA1981; EBI3; FLJ10374; SHD; MGC23244; FSD1; STAP2; FLJ14981; SH3GL1; CHAF1A; UBXD1; MGC2641; LRG; SEMA6B; MGC17791; C19orf10; DPP9; PGSF1; FEM1A; TRIF; TIP47 |
| 163154 | MGC24975 | 19 | 5733970 | 19p13.3 | gneg 18; gpos 1; | 1.24 | 0.52533827 | 0.044 | -0.122 | 0.387 | 0.224 | PTPRS; LOC148066; PLAC2; SAFB2; SAFB; 125988; SCDR10; RPL36; PRSS15; MGC39581; MGC24975; LOC56931; FUT6; NRTN; FUT3; FUT5; NDUFA11; CAPS; RANBP3; RFX2; BGR |
| 56931 | LOC56931 | 19 | 5736154 | 19p13.3 | gneg 18; gpos 1; | 1.71 | 0.094317865 | 0.029 | 0.309 | 0.42 | -0.567 | LOC148066; PLAC2; SAFB2; SAFB; 125988; SCDR10; RPL36; PRSS15; MGC39581; MGC24975; LOC56931; FUT6; NRTN; FUT3; FUT5; NDUFA11; CAPS; RANBP3; RFX2; BGR; MLLT1 |
| 2528 | FUT6 | 19 | 5767403 | 19p13.3 | gneg 18; gpos 1; | -1.71 | 0.041905159 | 0.124 | -0.368 | 0.392 | 0.674 | PLAC2; SAFB2; SAFB; 125988; SCDR10; RPL36; PRSS15; MGC39581; MGC24975; LOC56931; FUT6; NRTN; FUT3; FUT5; NDUFA11; CAPS; RANBP3; RFX2; BGR; MLLT1; ASAH3 |
| 126328 | NDUFA11 | 19 | 5845685 | 19p13.3 | gneg 19; gpos 1; | 1.01 | 0.012064681 | -0.225 | -0.438 | 0.343 | 0.803 | SCDR10; RPL36; PRSS15; MGC39581; MGC24975; LOC56931; FUT6; NRTN; FUT3; FUT5; NDUFA11; CAPS; RANBP3; RFX2; BGR; MLLT1; ASAH3; CLPP; SPATA11; PSPN; GTF2F1 |
| 828 | CAPS | 19 | 5865218 | 19p13.3 | gneg 19; gpos 1; | 1.24 | 0.819326556 | 0.177 | -0.044 | 0.329 | 0.081 | RPL36; PRSS15; MGC39581; MGC24975; LOC56931; FUT6; NRTN; FUT3; FUT5; NDUFA11; CAPS; RANBP3; RFX2; BGR; MLLT1; ASAH3; CLPP; SPATA11; PSPN; GTF2F1; KHSRP |
| 5609 | MAP2K7 | 19 | 7874764 | 19p13.3-p13.2 | gneg 9; gpos 4; | -1 | 0.183844701 | 0 | -0.25 | 0.308 | 0.458 | XAB2; STXBP2; RETN; LOC199675; TRAPPC5; FCER2; UNQ431; CD209; LOC115704; FLJ23420; MAP2K7; SNAPC2; 404217; TIMM44; ELAVL1; CCL25; FBN3; LASS4; 8D6A; NDUFA7; RPS28 |
| 51293 | 8D6A | 19 | 8273010 | 19p13.3-p13.2 | gneg 9; gpos 4; | 1.47 | 0.669346856 | 0.083 | 0.083 | 0.309 | -0.151 | LOC115704; FLJ23420; MAP2K7; SNAPC2; 404217; TIMM44; ELAVL1; CCL25; FBN3; LASS4; 8D6A; NDUFA7; RPS28; FLJ46061; ANGPTL4; RAB11B; LOC51257; HNRPM; PRAM-1; MGC15716; MYO1F |
| 6234 | RPS28 | 19 | 8292383 | 19p13.2 | gneg 8; gpos 5; | -1.1 | 0.063492273 | -0.147 | -0.339 | 0.366 | 0.622 | MAP2K7; SNAPC2; 404217; TIMM44; ELAVL1; CCL25; FBN3; LASS4; 8D6A; NDUFA7; RPS28; FLJ46061; ANGPTL4; RAB11B; LOC51257; HNRPM; PRAM-1; MGC15716; MYO1F; ADAMTS10; MGC33407 |
| 51257 | LOC51257 | 19 | 8384186 | 19p13.3 | gneg 8; gpos 9; | -1.13 | 0.020332954 | -0.016 | -0.411 | 0.354 | 0.754 | ELAVL1; CCL25; FBN3; LASS4; 8D6A; NDUFA7; RPS28; FLJ46061; ANGPTL4; RAB11B; LOC51257; HNRPM; PRAM-1; MGC15716; MYO1F; ADAMTS10; MGC33407; 284383; FLJ30932; MBD3L1; FLJ31986 |
| 84330 | MGC15716 | 19 | 8482223 | 19p13.3 | gneg 8; gpos 9; | 1.43 | 0.96544463 | -0.041 | -0.008 | 0.316 | 0.015 | LASS4; 8D6A; NDUFA7; RPS28; FLJ46061; ANGPTL4; RAB11B; LOC51257; HNRPM; PRAM-1; MGC15716; MYO1F; ADAMTS10; MGC33407; 284383; FLJ30932; MBD3L1; FLJ31986; 125963; 125962; ZNF317 |
| 57693 | ZNF317 | 19 | 9112072 | 19p13.2 | gneg 2; gpos 17; | 1.33 | 0.696928113 | -0.136 | 0.075 | 0.341 | -0.138 | MGC15716; MYO1F; ADAMTS10; MGC33407; 284383; FLJ30932; MBD3L1; FLJ31986; 125963; 125962; ZNF317; FLJ38149; 125958; MGC13105; ZNF177; ZNF266; ZNF426; MGC45408; FLJ20079; 342969; LOC162993 |
| 54811 | FLJ20079 | 19 | 9620352 | 19p13.2 | gneg 1; gpos 17; | -1.03 | 0.375292843 | -0.004 | -0.17 | 0.366 | 0.311 | 125963; 125962; ZNF317; FLJ38149; 125958; MGC13105; ZNF177; ZNF266; ZNF426; MGC45408; FLJ20079; 342969; LOC162993; FBXL12; UBL5; PIN1; OLFM2; COL5A3; RDH8; FLJ11286; ANGPTL6 |
| 162993 | LOC162993 | 19 | 9759083 | 19p13.2 | gneg 1; gpos 16; | 1.47 | 0.902278347 | -0.058 | 0.024 | 0.381 | -0.044 | ZNF317; FLJ38149; 125958; MGC13105; ZNF177; ZNF266; ZNF426; MGC45408; FLJ20079; 342969; LOC162993; FBXL12; UBL5; PIN1; OLFM2; COL5A3; RDH8; FLJ11286; ANGPTL6; PPAN; P2RY11 |
| 5300 | PIN1 | 19 | 9807012 | 19p13 | gneg 1; gpos 17; | 1 | 0.11720926 | -0.041 | -0.291 | 0.329 | 0.534 | MGC13105; ZNF177; ZNF266; ZNF426; MGC45408; FLJ20079; 342969; LOC162993; FBXL12; UBL5; PIN1; OLFM2; COL5A3; RDH8; FLJ11286; ANGPTL6; PPAN; P2RY11; EIF3S4; DNMT1; EDG5 |
| 93145 | OLFM2 | 19 | 9825394 | 19p13.2 | gneg 1; gpos 16; | 1.52 | 0.039753011 | -0.041 | 0.371 | 0.351 | -0.681 | ZNF177; ZNF266; ZNF426; MGC45408; FLJ20079; 342969; LOC162993; FBXL12; UBL5; PIN1; OLFM2; COL5A3; RDH8; FLJ11286; ANGPTL6; PPAN; P2RY11; EIF3S4; DNMT1; EDG5; 388504 |
| 50509 | COL5A3 | 19 | 9931236 | 19p13.2 | gneg 1; gpos 15; | 2.47 | 0.337973855 | 0.023 | 0.183 | 0.355 | -0.335 | ZNF266; ZNF426; MGC45408; FLJ20079; 342969; LOC162993; FBXL12; UBL5; PIN1; OLFM2; COL5A3; RDH8; FLJ11286; ANGPTL6; PPAN; P2RY11; EIF3S4; DNMT1; EDG5; 388504; MRPL4 |
| 50700 | RDH8 | 19 | 9984924 | 19p13.2-p13.3 | gneg 1; gpos 14; | -1 | 0.990871722 | 0.097 | 0.002 | 0.327 | -0.004 | ZNF426; MGC45408; FLJ20079; 342969; LOC162993; FBXL12; UBL5; PIN1; OLFM2; COL5A3; RDH8; FLJ11286; ANGPTL6; PPAN; P2RY11; EIF3S4; DNMT1; EDG5; 388504; MRPL4; ICAM1 |
| 55337 | FLJ11286 | 19 | 10058008 | 19p13.2 | gneg 1; gpos 13; | 1.78 | 0.018481491 | 0.068 | 0.416 | 0.377 | -0.763 | MGC45408; FLJ20079; 342969; LOC162993; FBXL12; UBL5; PIN1; OLFM2; COL5A3; RDH8; FLJ11286; ANGPTL6; PPAN; P2RY11; EIF3S4; DNMT1; EDG5; 388504; MRPL4; ICAM1; 402414 |
| 56342 | PPAN | 19 | 10077964 | 19p13 | gneg 1; gpos 12; | 2.1 | 0.105276635 | 0.007 | 0.3 | 0.453 | -0.551 | 342969; LOC162993; FBXL12; UBL5; PIN1; OLFM2; COL5A3; RDH8; FLJ11286; ANGPTL6; PPAN; P2RY11; EIF3S4; DNMT1; EDG5; 388504; MRPL4; ICAM1; 402414; ICAM4; ICAM5 |
| 5032 | P2RY11 | 19 | 10083196 | 19p13.2 | gneg 1; gpos 12; | -1.44 | 0.115782243 | -0.131 | -0.292 | 0.531 | 0.536 | LOC162993; FBXL12; UBL5; PIN1; OLFM2; COL5A3; RDH8; FLJ11286; ANGPTL6; PPAN; P2RY11; EIF3S4; DNMT1; EDG5; 388504; MRPL4; ICAM1; 402414; ICAM4; ICAM5; MGC19604 |
| 8666 | EIF3S4 | 19 | 10086690 | 19p13.2 | gneg 1; gpos 12; | 1.04 | 0.21487246 | 0.076 | -0.234 | 0.432 | 0.429 | FBXL12; UBL5; PIN1; OLFM2; COL5A3; RDH8; FLJ11286; ANGPTL6; PPAN; P2RY11; EIF3S4; DNMT1; EDG5; 388504; MRPL4; ICAM1; 402414; ICAM4; ICAM5; MGC19604; RAVER1 |
| 9294 | EDG5 | 19 | 10195519 | 19p13.2 | gpos 12; | 1.25 | 0.687132563 | -0.052 | -0.078 | 0.407 | 0.143 | PIN1; OLFM2; COL5A3; RDH8; FLJ11286; ANGPTL6; PPAN; P2RY11; EIF3S4; DNMT1; EDG5; 388504; MRPL4; ICAM1; 402414; ICAM4; ICAM5; MGC19604; RAVER1; ICAM3; TYK2 |
| 388504 | not_found | 19 | 10202079 | 19p13 | gpos 13; | -1.39 | 0.062297124 | -0.152 | -0.341 | 0.452 | 0.625 | OLFM2; COL5A3; RDH8; FLJ11286; ANGPTL6; PPAN; P2RY11; EIF3S4; DNMT1; EDG5; 388504; MRPL4; ICAM1; 402414; ICAM4; ICAM5; MGC19604; RAVER1; ICAM3; TYK2; CDC37 |
| 51073 | MRPL4 | 19 | 10223639 | 19p13 | gpos 13; | 1.18 | 0.398637338 | -0.04 | -0.162 | 0.405 | 0.296 | COL5A3; RDH8; FLJ11286; ANGPTL6; PPAN; P2RY11; EIF3S4; DNMT1; EDG5; 388504; MRPL4; ICAM1; 402414; ICAM4; ICAM5; MGC19604; RAVER1; ICAM3; TYK2; CDC37; PDE4A |
| 3383 | ICAM1 | 19 | 10242778 | 19p13.3-p13.2 | gpos 13; | 1.24 | 0.888918515 | -0.071 | 0.027 | 0.338 | -0.05 | RDH8; FLJ11286; ANGPTL6; PPAN; P2RY11; EIF3S4; DNMT1; EDG5; 388504; MRPL4; ICAM1; 402414; ICAM4; ICAM5; MGC19604; RAVER1; ICAM3; TYK2; CDC37; PDE4A; KEAP1 |
| 163051 | FLJ38281 | 19 | 12433372 | 19p13.2 | gpos 14; | 1.04 | 0.141075663 | 0.051 | -0.275 | 0.46 | 0.504 | 388507; ZNF20; LOC90589; ZNF136; 388509; ZNF44; LOC147837; ZNF442; 403113; ZNF443; FLJ38281; MGC26914; ZNF490; FLJ90396; MAN2B1; PTD008; MGC4238; DHPS; MGC10870; TNPO2; MGC2803 |
| 163050 | MGC26914 | 19 | 12497184 | 19p13.2 | gpos 14; | 1.17 | 0.940199102 | 0.01 | -0.015 | 0.344 | 0.027 | ZNF20; LOC90589; ZNF136; 388509; ZNF44; LOC147837; ZNF442; 403113; ZNF443; FLJ38281; MGC26914; ZNF490; FLJ90396; MAN2B1; PTD008; MGC4238; DHPS; MGC10870; TNPO2; MGC2803; ASNA1 |
| 163049 | FLJ90396 | 19 | 12582752 | 19p13.2 | gpos 15; | -1.54 | 0.034331354 | -0.025 | -0.38 | 0.469 | 0.697 | ZNF136; 388509; ZNF44; LOC147837; ZNF442; 403113; ZNF443; FLJ38281; MGC26914; ZNF490; FLJ90396; MAN2B1; PTD008; MGC4238; DHPS; MGC10870; TNPO2; MGC2803; ASNA1; VMD2L1; HOOK2 |
| 4125 | MAN2B1 | 19 | 12618321 | 19cen-q13.1 | gpos 16; | 1.03 | 0.034853177 | 0.029 | -0.38 | 0.326 | 0.696 | 388509; ZNF44; LOC147837; ZNF442; 403113; ZNF443; FLJ38281; MGC26914; ZNF490; FLJ90396; MAN2B1; PTD008; MGC4238; DHPS; MGC10870; TNPO2; MGC2803; ASNA1; VMD2L1; HOOK2; JUNB |
| 51398 | PTD008 | 19 | 12639886 | 19p13.2 | gpos 17; | -1.2 | 0.000221538 | -0.122 | -0.576 | 0.356 | 1.056 | ZNF44; LOC147837; ZNF442; 403113; ZNF443; FLJ38281; MGC26914; ZNF490; FLJ90396; MAN2B1; PTD008; MGC4238; DHPS; MGC10870; TNPO2; MGC2803; ASNA1; VMD2L1; HOOK2; JUNB; PRDX2 |
| 84292 | MGC4238 | 19 | 12641583 | 19p13.2 | gpos 17; | 1.26 | 0.543326986 | 0.024 | 0.117 | 0.396 | -0.215 | LOC147837; ZNF442; 403113; ZNF443; FLJ38281; MGC26914; ZNF490; FLJ90396; MAN2B1; PTD008; MGC4238; DHPS; MGC10870; TNPO2; MGC2803; ASNA1; VMD2L1; HOOK2; JUNB; PRDX2; RNASEH2A |
| 84261 | MGC10870 | 19 | 12660730 | 19p13.2 | gpos 16; gvar 1; | -1.2 | 0.041333253 | -0.092 | -0.369 | 0.475 | 0.676 | 403113; ZNF443; FLJ38281; MGC26914; ZNF490; FLJ90396; MAN2B1; PTD008; MGC4238; DHPS; MGC10870; TNPO2; MGC2803; ASNA1; VMD2L1; HOOK2; JUNB; PRDX2; RNASEH2A; RTBDN; SAST |
| 30000 | TNPO2 | 19 | 12671014 | 19p13.2 | gpos 17; gvar 1; | -1.22 | 0.267730467 | 0.045 | -0.21 | 0.332 | 0.386 | ZNF443; FLJ38281; MGC26914; ZNF490; FLJ90396; MAN2B1; PTD008; MGC4238; DHPS; MGC10870; TNPO2; MGC2803; ASNA1; VMD2L1; HOOK2; JUNB; PRDX2; RNASEH2A; RTBDN; SAST; DNASE2 |
| 79002 | MGC2803 | 19 | 12702454 | 19p13.2 | gpos 16; gvar 1; | -1.09 | 0.02372979 | -0.134 | -0.402 | 0.496 | 0.738 | FLJ38281; MGC26914; ZNF490; FLJ90396; MAN2B1; PTD008; MGC4238; DHPS; MGC10870; TNPO2; MGC2803; ASNA1; VMD2L1; HOOK2; JUNB; PRDX2; RNASEH2A; RTBDN; SAST; DNASE2; KLF1 |
| 439 | ASNA1 | 19 | 12709305 | 19q13.3 | gpos 16; gvar 1; | 1.44 | 0.892171957 | -0.196 | 0.026 | 0.464 | -0.048 | MGC26914; ZNF490; FLJ90396; MAN2B1; PTD008; MGC4238; DHPS; MGC10870; TNPO2; MGC2803; ASNA1; VMD2L1; HOOK2; JUNB; PRDX2; RNASEH2A; RTBDN; SAST; DNASE2; KLF1; GCDH |
| 54831 | VMD2L1 | 19 | 12724471 | 19p13.2 | gpos 16; gvar 1; | 1.75 | 0.031308872 | 0.124 | 0.386 | 0.46 | -0.708 | ZNF490; FLJ90396; MAN2B1; PTD008; MGC4238; DHPS; MGC10870; TNPO2; MGC2803; ASNA1; VMD2L1; HOOK2; JUNB; PRDX2; RNASEH2A; RTBDN; SAST; DNASE2; KLF1; GCDH; FARSLA |
| 7001 | PRDX2 | 19 | 12772426 | 19p13.2 | gpos 16; gvar 1; | -2.07 | 0.109446008 | 0.064 | -0.297 | 0.405 | 0.545 | PTD008; MGC4238; DHPS; MGC10870; TNPO2; MGC2803; ASNA1; VMD2L1; HOOK2; JUNB; PRDX2; RNASEH2A; RTBDN; SAST; DNASE2; KLF1; GCDH; FARSLA; CALR; RAD23A; PLINP-1 |
| 10535 | RNASEH2A | 19 | 12778427 | 19p13.2 | gpos 16; gvar 1; | 1.43 | 0.05301588 | 0.255 | 0.352 | 0.402 | -0.645 | MGC4238; DHPS; MGC10870; TNPO2; MGC2803; ASNA1; VMD2L1; HOOK2; JUNB; PRDX2; RNASEH2A; RTBDN; SAST; DNASE2; KLF1; GCDH; FARSLA; CALR; RAD23A; PLINP-1; FLJ38607 |
| 2193 | FARSLA | 19 | 12894292 | 19p13.2 | gneg 5; gpos 13; gvar 1; | 1.52 | 0.140698493 | 0.303 | 0.275 | 0.32 | -0.505 | VMD2L1; HOOK2; JUNB; PRDX2; RNASEH2A; RTBDN; SAST; DNASE2; KLF1; GCDH; FARSLA; CALR; RAD23A; PLINP-1; FLJ38607; NFIX; LYL1; FLJ20244; BTBD14B; STX10; ETR101 |
| 3640 | INSL3 | 19 | 17788321 | 19p13.2-p12 | gneg 1; gpos 11; | 6.76 | 0.052230218 | 0.143 | 0.353 | 0.41 | -0.647 | BST2; LOC93343; LOC115861; SLC27A1; PGLS; BCNP1; FLJ22329; VCY2IP1; FCHO1; B3GNT3; INSL3; JAK3; RPL18A; SLC5A5; LOC115098; KCNN1; CLONE24945; IL12RB1; PIK3R2; IFI30; MGC12972 |
| 3718 | JAK3 | 19 | 17797960 | 19p13.1 | gneg 1; gpos 11; | -1.42 | 0.294625777 | 0.219 | -0.199 | 0.35 | 0.366 | LOC93343; LOC115861; SLC27A1; PGLS; BCNP1; FLJ22329; VCY2IP1; FCHO1; B3GNT3; INSL3; JAK3; RPL18A; SLC5A5; LOC115098; KCNN1; CLONE24945; IL12RB1; PIK3R2; IFI30; MGC12972; RAB3A |
| 6528 | SLC5A5 | 19 | 17843781 | 19p13.2-p12 | gneg 2; gpos 10; | 1.16 | 0.412065046 | 0.13 | -0.157 | 0.329 | 0.288 | SLC27A1; PGLS; BCNP1; FLJ22329; VCY2IP1; FCHO1; B3GNT3; INSL3; JAK3; RPL18A; SLC5A5; LOC115098; KCNN1; CLONE24945; IL12RB1; PIK3R2; IFI30; MGC12972; RAB3A; PDE4C; JUND |
| 55049 | FLJ20850 | 19 | 18560607 | 19p13.11 | gneg 9; gvar 6; | 1.24 | 0.406840276 | 0.227 | 0.159 | 0.325 | -0.291 | LSM4; PGPEP1; PLAB; MAPA; SSBP4; ISYNA1; ELL; FKBP8; MGC2749; UBA52; FLJ20850; CRLF1; C19orf4; FLJ11078; MECT1; COMP; RENT1; GDF1; LASS1; COPE; DDX49 |
| 80264 | ZNF430 | 19 | 20995336 | 19p13.11 | gneg 13; gvar 2; | -1.74 | 0.030819896 | 0.299 | -0.387 | 0.361 | 0.71 | EDG4; GMIP; CGI-152; HZF12; ZNF14; ZNF253; ZNF505; LOC91120; LOC199777; ZNF85; ZNF430; ZNF431; ZNF493; LOC115648; 400680; ZNF100; ZNF43; ZNF208; ZNF257; ZNF492; 126502 |
| 170959 | ZNF431 | 19 | 21116727 | 19p13.11 | gneg 13; gvar 1; | 1.09 | 0.578701559 | 0.028 | -0.107 | 0.44 | 0.196 | GMIP; CGI-152; HZF12; ZNF14; ZNF253; ZNF505; LOC91120; LOC199777; ZNF85; ZNF430; ZNF431; ZNF493; LOC115648; 400680; ZNF100; ZNF43; ZNF208; ZNF257; ZNF492; 126502; ZNF91 |
| 115648 | LOC115648 | 19 | 21371797 | 19p13.11 | gneg 12; gvar 3; | 1.38 | 0.626094898 | -0.01 | -0.094 | 0.355 | 0.172 | HZF12; ZNF14; ZNF253; ZNF505; LOC91120; LOC199777; ZNF85; ZNF430; ZNF431; ZNF493; LOC115648; 400680; ZNF100; ZNF43; ZNF208; ZNF257; ZNF492; 126502; ZNF91; TIZ; LOC148213 |
| 400680 | not_found | 19 | 21472851 | 19p12 | gneg 11; gvar 3; | -1.88 | 0.020711177 | 0.297 | -0.41 | 0.339 | 0.752 | ZNF14; ZNF253; ZNF505; LOC91120; LOC199777; ZNF85; ZNF430; ZNF431; ZNF493; LOC115648; 400680; ZNF100; ZNF43; ZNF208; ZNF257; ZNF492; 126502; ZNF91; TIZ; LOC148213; ZNF254 |
| 57615 | ZNF492 | 19 | 22618076 | 19p13.11 | gneg 10; gvar 3; | -1.77 | 0.04323019 | 0.257 | -0.366 | 0.353 | 0.671 | ZNF85; ZNF430; ZNF431; ZNF493; LOC115648; 400680; ZNF100; ZNF43; ZNF208; ZNF257; ZNF492; 126502; ZNF91; TIZ; LOC148213; ZNF254; 399655; UQCRFS1; POP4; PLEKHF1; DKFZP762D096 |
| 126502 | not_found | 19 | 22731437 | 19p13.11 | gneg 10; gvar 4; | -1.69 | 0.014390701 | -0.051 | -0.429 | 0.374 | 0.787 | ZNF430; ZNF431; ZNF493; LOC115648; 400680; ZNF100; ZNF43; ZNF208; ZNF257; ZNF492; 126502; ZNF91; TIZ; LOC148213; ZNF254; 399655; UQCRFS1; POP4; PLEKHF1; DKFZP762D096; CCNE1 |
| 7644 | ZNF91 | 19 | 23333882 | 19p13.1-p12 | gneg 9; gvar 5; | -1.52 | 0.05130834 | 0.181 | -0.354 | 0.315 | 0.65 | ZNF431; ZNF493; LOC115648; 400680; ZNF100; ZNF43; ZNF208; ZNF257; ZNF492; 126502; ZNF91; TIZ; LOC148213; ZNF254; 399655; UQCRFS1; POP4; PLEKHF1; DKFZP762D096; CCNE1; C19orf2 |
| 57817 | HAMP | 19 | 40465249 | 19q13.1 | gneg 7; | 1.04 | 0.478834865 | 0.077 | -0.136 | 0.361 | 0.25 | SCN1B; HPN; FXYD3; LGI4; FXYD1; FXYD7; FXYD5; FLJ25660; LISCH7; USF2; HAMP; MAG; CD22; GPR40; GPR41; GPR42; GPR43; ZD52F10; UNQ698; GAPDS; NIFIE14 |
| 2866 | GPR42 | 19 | 40554101 | 19q13.1 | gneg 8; | -1.17 | 0.721755502 | 0.025 | -0.069 | 0.424 | 0.126 | FXYD7; FXYD5; FLJ25660; LISCH7; USF2; HAMP; MAG; CD22; GPR40; GPR41; GPR42; GPR43; ZD52F10; UNQ698; GAPDS; NIFIE14; ATP4A; MGC10433; LOC339321; COX6B; UPK1A |
| 10430 | NIFIE14 | 19 | 40728384 | 19q13.1 | gneg 8; | 1.27 | 0.277227525 | -0.096 | 0.206 | 0.328 | -0.378 | HAMP; MAG; CD22; GPR40; GPR41; GPR42; GPR43; ZD52F10; UNQ698; GAPDS; NIFIE14; ATP4A; MGC10433; LOC339321; COX6B; UPK1A; TZFP; MLL4; FLJ22573; U2AF1L3; PEN2 |
| 84518 | LOC84518 | 19 | 47583012 | 19q13.31 | gneg 9; gpos 9; | 1.43 | 0.870058324 | 0.182 | -0.032 | 0.31 | 0.058 | FLJ22059; POU2F2; DEDD2; KIAA1951; GSK3A; ERF; CIC; PAFAH1B3; FLJ90805; SBP1; LOC84518; LIPE; UNQ473; CEACAM1; CEACAM8; PSG3; PSG1; PSG6; PSG7; PSG11; PSG2 |
| 79760 | GEMIN7 | 19 | 50274386 | 19q13.32 | gpos 12; | 1.32 | 0.737477489 | 0.003 | 0.065 | 0.315 | -0.119 | LU; PVRL2; APOE; APOC1; APOC4; APOC2; CLPTM1; RELB; SFRS16; ZNF342; GEMIN7; FLJ33600; MGC2650; XTP7; MARK4; CKM; KLC2L; ERCC2; RAI; ASE-1; ERCC1 |
| 79090 | MGC2650 | 19 | 50358026 | 19q13.32 | gpos 13; | 1.15 | 0.554594207 | -0.007 | -0.114 | 0.349 | 0.209 | APOE; APOC1; APOC4; APOC2; CLPTM1; RELB; SFRS16; ZNF342; GEMIN7; FLJ33600; MGC2650; XTP7; MARK4; CKM; KLC2L; ERCC2; RAI; ASE-1; ERCC1; FOSB; RTN2 |
| 90332 | XTP7 | 19 | 50407718 | 19q13.32 | gpos 13; | -1.03 | 0.164682348 | -0.039 | -0.261 | 0.388 | 0.478 | APOC1; APOC4; APOC2; CLPTM1; RELB; SFRS16; ZNF342; GEMIN7; FLJ33600; MGC2650; XTP7; MARK4; CKM; KLC2L; ERCC2; RAI; ASE-1; ERCC1; FOSB; RTN2; FLJ40125 |
| 147700 | KLC2L | 19 | 50540497 | 19q13 | gpos 11; | 1.78 | 0.245713676 | 0.122 | 0.22 | 0.498 | -0.403 | CLPTM1; RELB; SFRS16; ZNF342; GEMIN7; FLJ33600; MGC2650; XTP7; MARK4; CKM; KLC2L; ERCC2; RAI; ASE-1; ERCC1; FOSB; RTN2; FLJ40125; VASP; OPA3; GPR4 |
| 2068 | ERCC2 | 19 | 50546685 | 19q13.3 | gpos 12; | -1.07 | 0.043931389 | 0.012 | -0.365 | 0.457 | 0.669 | RELB; SFRS16; ZNF342; GEMIN7; FLJ33600; MGC2650; XTP7; MARK4; CKM; KLC2L; ERCC2; RAI; ASE-1; ERCC1; FOSB; RTN2; FLJ40125; VASP; OPA3; GPR4; EML2 |
| 10849 | ASE-1 | 19 | 50601306 | 19q13.3 | gpos 12; | 3.66 | 5.53E-05 | -0.024 | 0.606 | 0.468 | -1.111 | ZNF342; GEMIN7; FLJ33600; MGC2650; XTP7; MARK4; CKM; KLC2L; ERCC2; RAI; ASE-1; ERCC1; FOSB; RTN2; FLJ40125; VASP; OPA3; GPR4; EML2; GIPR; SNRPD2 |
| 2067 | ERCC1 | 19 | 50604711 | 19q13.2-q13.3 | gpos 12; | -1.29 | 0.001758451 | -0.147 | -0.517 | 0.399 | 0.948 | GEMIN7; FLJ33600; MGC2650; XTP7; MARK4; CKM; KLC2L; ERCC2; RAI; ASE-1; ERCC1; FOSB; RTN2; FLJ40125; VASP; OPA3; GPR4; EML2; GIPR; SNRPD2; FLJ20084 |
| 2354 | FOSB | 19 | 50663092 | 19q13.32 | gpos 12; | 1.03 | 0.268219121 | -0.104 | -0.21 | 0.33 | 0.385 | FLJ33600; MGC2650; XTP7; MARK4; CKM; KLC2L; ERCC2; RAI; ASE-1; ERCC1; FOSB; RTN2; FLJ40125; VASP; OPA3; GPR4; EML2; GIPR; SNRPD2; FLJ20084; SIX5 |
| 147699 | FLJ40125 | 19 | 50693704 | 19q13.32 | gpos 10; | 3.84 | 0.204610225 | -0.011 | 0.239 | 0.304 | -0.439 | XTP7; MARK4; CKM; KLC2L; ERCC2; RAI; ASE-1; ERCC1; FOSB; RTN2; FLJ40125; VASP; OPA3; GPR4; EML2; GIPR; SNRPD2; FLJ20084; SIX5; DMPK; RSHL1 |
| 7408 | VASP | 19 | 50702615 | 19q13.2-q13.3 | gpos 9; | 1.23 | 0.47035047 | 0.008 | 0.139 | 0.348 | -0.254 | MARK4; CKM; KLC2L; ERCC2; RAI; ASE-1; ERCC1; FOSB; RTN2; FLJ40125; VASP; OPA3; GPR4; EML2; GIPR; SNRPD2; FLJ20084; SIX5; DMPK; RSHL1; SYMPK |
| 80207 | OPA3 | 19 | 50747641 | 19q13.32 | gpos 9; | 1.09 | 0.265540016 | -0.102 | -0.211 | 0.445 | 0.387 | CKM; KLC2L; ERCC2; RAI; ASE-1; ERCC1; FOSB; RTN2; FLJ40125; VASP; OPA3; GPR4; EML2; GIPR; SNRPD2; FLJ20084; SIX5; DMPK; RSHL1; SYMPK; FOXA3 |
| 2696 | GIPR | 19 | 50863341 | 19q13.3 | gpos 11; | -1.28 | 0.263154243 | -0.016 | -0.212 | 0.406 | 0.389 | RAI; ASE-1; ERCC1; FOSB; RTN2; FLJ40125; VASP; OPA3; GPR4; EML2; GIPR; SNRPD2; FLJ20084; SIX5; DMPK; RSHL1; SYMPK; FOXA3; IRF2BP1; NANOS2; NOVA2 |
| 6633 | SNRPD2 | 19 | 50882557 | 19q13.2 | gpos 10; | 2.03 | 0.122949373 | -0.026 | 0.287 | 0.45 | -0.526 | ASE-1; ERCC1; FOSB; RTN2; FLJ40125; VASP; OPA3; GPR4; EML2; GIPR; SNRPD2; FLJ20084; SIX5; DMPK; RSHL1; SYMPK; FOXA3; IRF2BP1; NANOS2; NOVA2; PGLYRP |
| 81492 | RSHL1 | 19 | 50990808 | 19q13.3 | gneg 4; gpos 8; | 1.45 | 0.730792911 | -0.11 | 0.067 | 0.37 | -0.122 | FLJ40125; VASP; OPA3; GPR4; EML2; GIPR; SNRPD2; FLJ20084; SIX5; DMPK; RSHL1; SYMPK; FOXA3; IRF2BP1; NANOS2; NOVA2; PGLYRP; 147920; DKFZp434J0226; UNQ644; HIF3A |
| 8189 | SYMPK | 19 | 51010561 | 19q13.3 | gneg 4; gpos 7; | 1.14 | 0.37104321 | -0.043 | -0.171 | 0.336 | 0.314 | VASP; OPA3; GPR4; EML2; GIPR; SNRPD2; FLJ20084; SIX5; DMPK; RSHL1; SYMPK; FOXA3; IRF2BP1; NANOS2; NOVA2; PGLYRP; 147920; DKFZp434J0226; UNQ644; HIF3A; PPP5C |
| 11133 | KPTN | 19 | 52670219 | 19q13.33 | gneg 9; | 1.93 | 0.008760908 | 0.178 | 0.453 | 0.415 | -0.831 | GRLF1; NPAS1; FLJ20512; SAE1; BBC3; DKFZP586M1019; C5R1; GPR77; DHX34; SLC8A2; KPTN; NAPA; DKFZp434I1930; GLTSCR1; EHD2; GLTSCR2; SEPW1; FLJ40321; CRX; SULT2A1; ELSPBP1 |
| 23521 | RPL13A | 19 | 54682676 | 19q13.3 | gneg 8; | 1.23 | 0.03279013 | 0.238 | 0.383 | 0.311 | -0.703 | TRPM4; SLC6A16; CD37; TEAD2; DKKL1-pending; TIP39; SLC17A7; FLJ20643; MGC10204; FLT3LG; RPL13A; RPS11; FCGRT; RCN3; NOSIP; PRRG2; RRAS; SR-A1; IRF3; BCL2L12; HRMT1L2 |
| 6205 | RPS11 | 19 | 54691445 | 19q13.3 | gneg 8; | 2.69 | 9.33E-06 | 0.05 | 0.636 | 0.402 | -1.165 | SLC6A16; CD37; TEAD2; DKKL1-pending; TIP39; SLC17A7; FLJ20643; MGC10204; FLT3LG; RPL13A; RPS11; FCGRT; RCN3; NOSIP; PRRG2; RRAS; SR-A1; IRF3; BCL2L12; HRMT1L2; LOC199800 |
| 51070 | NOSIP | 19 | 54750781 | 19q13.33 | gneg 10; | 1.02 | 0.454693626 | -0.039 | -0.144 | 0.342 | 0.263 | DKKL1-pending; TIP39; SLC17A7; FLJ20643; MGC10204; FLT3LG; RPL13A; RPS11; FCGRT; RCN3; NOSIP; PRRG2; RRAS; SR-A1; IRF3; BCL2L12; HRMT1L2; LOC199800; CPT1C; TSKS; AP2A1 |
| 11284 | PNKP | 19 | 55056273 | 19q13.3-q13.4 | gneg 11; | 1.2 | 0.882051254 | 0.169 | 0.029 | 0.362 | -0.053 | IRF3; BCL2L12; HRMT1L2; LOC199800; CPT1C; TSKS; AP2A1; FLJ22688; TCBAP0758; PTOV1; PNKP; MGC2865; FLJ12168; IL4I1; NUP62; ATF5; SIGLEC11; VRK3; ZNF473; 400710; MGC33947 |
| 126119 | SBBI54 | 19 | 55701070 | 19q13.41 | gneg 5; gpos 5; | -2.15 | 0.004543903 | 0.022 | -0.482 | 0.332 | 0.883 | ZNF473; 400710; MGC33947; MYH14; KCNC3; NR1H2; POLD1; SPIB; MYBPC2; LOC284361; SBBI54; DKFZp761A179; SYT3; SHANK1; SCGF; GPR32; ACPT; MGC13170; KLK1; KLK15; KLK3 |
| 6320 | SCGF | 19 | 55918416 | 19q13.3 | gneg 2; gpos 7; | -2.14 | 1.10E-06 | 0.068 | -0.662 | 0.371 | 1.214 | KCNC3; NR1H2; POLD1; SPIB; MYBPC2; LOC284361; SBBI54; DKFZp761A179; SYT3; SHANK1; SCGF; GPR32; ACPT; MGC13170; KLK1; KLK15; KLK3; KLK2; KLK4; KLK5; KLK6 |
| 2109 | ETFB | 19 | 56540236 | 19q13.3 | gpos 10; | -1.26 | 0.00175272 | 0.086 | -0.517 | 0.351 | 0.948 | KLK13; KLK14; LOC90353; SIGLEC9; SIGLEC7; LOC284367; CD33; FLJ40235; 339351; LOC147645; ETFB; MGC33839; NKG7; LIM2; LOC147646; SIGLEC10; SIGLEC8; SIGLECL1; SIGLEC6; ZNF175; 339352 |
| 7728 | ZNF175 | 19 | 56766342 | 19q13.4 | gpos 8; | 1.06 | 0.126779238 | 0.029 | -0.284 | 0.353 | 0.522 | LOC147645; ETFB; MGC33839; NKG7; LIM2; LOC147646; SIGLEC10; SIGLEC8; SIGLECL1; SIGLEC6; ZNF175; 339352; SIGLEC5; HAS1; FPR1; FPRL1; FPRL2; MGC4400; FLJ12644; FLJ13590; ZNF21 |
| 339352 | not_found | 19 | 56796537 | 19q13.41 | gpos 8; | -1.65 | 0.002088378 | 0.028 | -0.511 | 0.322 | 0.937 | ETFB; MGC33839; NKG7; LIM2; LOC147646; SIGLEC10; SIGLEC8; SIGLECL1; SIGLEC6; ZNF175; 339352; SIGLEC5; HAS1; FPR1; FPRL1; FPRL2; MGC4400; FLJ12644; FLJ13590; ZNF21; ZNF350 |
| 84765 | MGC4400 | 19 | 57066361 | 19q13.41 | gpos 11; | -1.21 | 0.212840833 | 0.125 | -0.235 | 0.306 | 0.431 | SIGLEC8; SIGLECL1; SIGLEC6; ZNF175; 339352; SIGLEC5; HAS1; FPR1; FPRL1; FPRL2; MGC4400; FLJ12644; FLJ13590; ZNF21; ZNF350; FLJ33710; FLJ21941; ZNF432; LOC90317; PPP2R1A; MGC32104 |
| 79986 | FLJ12985 | 19 | 58163316 | 19q13.42 | gneg 13; gpos 3; | -2.03 | 0.001017098 | 0.23 | -0.535 | 0.381 | 0.98 | MGC32104; FLJ36040; KIAA1827; FLJ31384; FLJ10891; ZNF137; ZNF83; MGC5384; KR-ZNF1; 399669; FLJ12985; FLJ32214; ZNF160; ZNF415; ZNF347; FLJ14345; MGC48625; VN1R2; VN1R4; BIRC8; KIAA1979 |
| 90338 | ZNF160 | 19 | 58260991 | 19q13.42 | gneg 14; gpos 1; | -1.02 | 0.384665407 | -0.021 | -0.166 | 0.328 | 0.305 | KIAA1827; FLJ31384; FLJ10891; ZNF137; ZNF83; MGC5384; KR-ZNF1; 399669; FLJ12985; FLJ32214; ZNF160; ZNF415; ZNF347; FLJ14345; MGC48625; VN1R2; VN1R4; BIRC8; KIAA1979; LOC91661; ZNF331 |
| 54776 | PPP1R12C | 19 | 60294094 | 19q13.42 | gneg 9; | -1.14 | 0.024910795 | 0.074 | -0.4 | 0.37 | 0.733 | KIR2DL4; KIR2DS4; KIR3DL2; FCAR; NCR1; NALP7; NALP2; GP6; RDH13; EPS8L1; PPP1R12C; TNNT1; TNNI3; LOC352909; SYT5; PTPRH; 388563; MGC30208; HSPBP1; KIAA1811; 284417 |
| 5794 | PTPRH | 19 | 60384427 | 19q13.4 | gneg 11; | -1.21 | 0.036737467 | -0.037 | -0.376 | 0.377 | 0.69 | NALP7; NALP2; GP6; RDH13; EPS8L1; PPP1R12C; TNNT1; TNNI3; LOC352909; SYT5; PTPRH; 388563; MGC30208; HSPBP1; KIAA1811; 284417; MGC2705; COXVIB2; IL11; MDAC1; RPL28 |
| 255043 | MGC30208 | 19 | 60429925 | 19q13.42 | gneg 10; gpos 1; | -1.08 | 0.309193994 | -0.049 | -0.194 | 0.41 | 0.355 | GP6; RDH13; EPS8L1; PPP1R12C; TNNT1; TNNI3; LOC352909; SYT5; PTPRH; 388563; MGC30208; HSPBP1; KIAA1811; 284417; MGC2705; COXVIB2; IL11; MDAC1; RPL28; UBE2S; LOC284296 |
| 23640 | HSPBP1 | 19 | 60465410 | 19q13.42 | gneg 11; gpos 1; | 1.3 | 0.835485643 | -0.036 | 0.04 | 0.478 | -0.074 | RDH13; EPS8L1; PPP1R12C; TNNT1; TNNI3; LOC352909; SYT5; PTPRH; 388563; MGC30208; HSPBP1; KIAA1811; 284417; MGC2705; COXVIB2; IL11; MDAC1; RPL28; UBE2S; LOC284296; FLJ23469 |
| 284417 | not_found | 19 | 60516164 | 19q13.42 | gneg 9; gpos 2; | 1.74 | 0.409169571 | -0.06 | -0.158 | 0.358 | 0.29 | PPP1R12C; TNNT1; TNNI3; LOC352909; SYT5; PTPRH; 388563; MGC30208; HSPBP1; KIAA1811; 284417; MGC2705; COXVIB2; IL11; MDAC1; RPL28; UBE2S; LOC284296; FLJ23469; KLP1; 390975 |
| 3589 | IL11 | 19 | 60567568 | 19q13.3-q13.4 | gneg 8; gpos 5; | -2.51 | 0.01866267 | -0.152 | -0.416 | 0.401 | 0.762 | LOC352909; SYT5; PTPRH; 388563; MGC30208; HSPBP1; KIAA1811; 284417; MGC2705; COXVIB2; IL11; MDAC1; RPL28; UBE2S; LOC284296; FLJ23469; KLP1; 390975; FLJ35453; FLJ14768; MGC23143 |
| 27338 | UBE2S | 19 | 60604462 | 19q13.43 | gneg 8; gpos 8; | 1.35 | 0.196190716 | -0.1 | 0.244 | 0.313 | -0.446 | 388563; MGC30208; HSPBP1; KIAA1811; 284417; MGC2705; COXVIB2; IL11; MDAC1; RPL28; UBE2S; LOC284296; FLJ23469; KLP1; 390975; FLJ35453; FLJ14768; MGC23143; 147808; LOC51157; HSPC189 |
| 3704 | ITPA | 20 | 3138055 | 20p | gneg 16; | 1.29 | 0.851045513 | -0.097 | 0.036 | 0.303 | -0.067 | VPS16; PTPRA; GNRH2; MRPS26; OXT; AVP; UBCE7IP5; FLJ13149; ProSAPiP1; C20orf116; ITPA; SLC4A11; ATRN; GFRA4; ADAM33; SN; HSPA12B; C20orf27; C20orf28; CENPB; CDC25B |
| 54976 | C20orf27 | 20 | 3682155 | 20p13 | gneg 15; | 2.25 | 0.007161611 | 0.161 | 0.462 | 0.318 | -0.848 | FLJ13149; ProSAPiP1; C20orf116; ITPA; SLC4A11; ATRN; GFRA4; ADAM33; SN; HSPA12B; C20orf27; C20orf28; CENPB; CDC25B; C20orf29; KIAA1271; PANK2; RNF24; SMOX; ADRA1D; PRNP |
| 1059 | CENPB | 20 | 3712499 | 20p13 | gneg 14; | 1.32 | 0.878549503 | 0.068 | -0.03 | 0.357 | 0.054 | C20orf116; ITPA; SLC4A11; ATRN; GFRA4; ADAM33; SN; HSPA12B; C20orf27; C20orf28; CENPB; CDC25B; C20orf29; KIAA1271; PANK2; RNF24; SMOX; ADRA1D; PRNP; PRND; LOC149830 |
| 26090 | C20orf22 | 20 | 25223379 | 20p11.21 | acen 4; gneg 9; | -1.37 | 0.069170762 | 0.026 | -0.333 | 0.312 | 0.611 | CST5; GGTLA4; C20orf39; CST7; C20orf3; ACAS2L; VSX1; LOC284798; ENTPD6; PYGB; C20orf22; KIAA0980; C20orf147; ZNF337; FLJ38374; DEFB118; DEFB119; DEFB123; REM; HM13; ID1 |
| 245932 | DEFB119 | 20 | 29428628 | 20q11.1 | acen 3; gneg 10; | 2 | 0.851270663 | 0.109 | 0.036 | 0.332 | -0.067 | VSX1; LOC284798; ENTPD6; PYGB; C20orf22; KIAA0980; C20orf147; ZNF337; FLJ38374; DEFB118; DEFB119; DEFB123; REM; HM13; ID1; COX4I2; BCL2L1; TPX2; MYLK2; FKHL18; DUSP15 |
| 28954 | REM | 20 | 29526765 | 20q11.21 | acen 3; gneg 11; | -1.18 | 0.019218087 | 0.069 | -0.414 | 0.376 | 0.759 | ENTPD6; PYGB; C20orf22; KIAA0980; C20orf147; ZNF337; FLJ38374; DEFB118; DEFB119; DEFB123; REM; HM13; ID1; COX4I2; BCL2L1; TPX2; MYLK2; FKHL18; DUSP15; C20orf126; 343702 |
| 3397 | ID1 | 20 | 29656752 | 20q11 | acen 3; gneg 11; | 1.76 | 0.025592232 | 0.025 | 0.398 | 0.302 | -0.73 | C20orf22; KIAA0980; C20orf147; ZNF337; FLJ38374; DEFB118; DEFB119; DEFB123; REM; HM13; ID1; COX4I2; BCL2L1; TPX2; MYLK2; FKHL18; DUSP15; C20orf126; 343702; C20orf160; HCK |
| 3692 | ITGB4BP | 20 | 33330138 | 20q12 | gneg 9; gpos 5; | -1.1 | 0.200606477 | -0.084 | -0.241 | 0.348 | 0.442 | CDC91L1; C20orf110; NCOA6; GGTL3; ACAS2; GSS; TRPC4AP; C20orf31; PROCR; MMP24; ITGB4BP; C20orf128; C20orf44; GDF5; CEP2; 343705; C20orf173; SDBCAG84; SPAG4; CPNE1; RBM12 |
| 55245 | C20orf44 | 20 | 33353794 | 20q11.23 | gneg 11; gpos 3; | 1.67 | 0.340056096 | 0.019 | 0.182 | 0.33 | -0.334 | NCOA6; GGTL3; ACAS2; GSS; TRPC4AP; C20orf31; PROCR; MMP24; ITGB4BP; C20orf128; C20orf44; GDF5; CEP2; 343705; C20orf173; SDBCAG84; SPAG4; CPNE1; RBM12; NFS1; C20orf52 |
| 8904 | CPNE1 | 20 | 33677381 | 20q11.23 | gneg 11; gpos 2; | -1.14 | 0.00544852 | 0.009 | -0.474 | 0.343 | 0.869 | MMP24; ITGB4BP; C20orf128; C20orf44; GDF5; CEP2; 343705; C20orf173; SDBCAG84; SPAG4; CPNE1; RBM12; NFS1; C20orf52; RNPC2; C20orf104; SCAND1; C20orf152; EPB41L1; C20orf4; DAP4 |
| 140823 | C20orf52 | 20 | 33750805 | 20q11.23 | gneg 12; gpos 1; | -1.16 | 0.039898236 | -0.03 | -0.371 | 0.334 | 0.68 | C20orf44; GDF5; CEP2; 343705; C20orf173; SDBCAG84; SPAG4; CPNE1; RBM12; NFS1; C20orf52; RNPC2; C20orf104; SCAND1; C20orf152; EPB41L1; C20orf4; DAP4; MYL9; TGIF2; C20orf24 |
| 51282 | SCAND1 | 20 | 34004959 | 20q11.1-q11.23 | gneg 13; gpos 1; | -1.02 | 0.131734581 | 0.004 | -0.281 | 0.328 | 0.515 | 343705; C20orf173; SDBCAG84; SPAG4; CPNE1; RBM12; NFS1; C20orf52; RNPC2; C20orf104; SCAND1; C20orf152; EPB41L1; C20orf4; DAP4; MYL9; TGIF2; C20orf24; SLA2; NDRG3; C20orf172 |
| 10398 | MYL9 | 20 | 34603310 | 20q11.23 | gneg 13; | -1.85 | 0.000796099 | -0.053 | -0.542 | 0.308 | 0.994 | RBM12; NFS1; C20orf52; RNPC2; C20orf104; SCAND1; C20orf152; EPB41L1; C20orf4; DAP4; MYL9; TGIF2; C20orf24; SLA2; NDRG3; C20orf172; FLJ44670; KIAA0889; 343574; SAMHD1; RBL1 |
| 79980 | C20orf172 | 20 | 34813861 | 20q11.23 | gneg 9; gpos 1; | 1.2 | 0.823785229 | 0.071 | -0.043 | 0.461 | 0.079 | SCAND1; C20orf152; EPB41L1; C20orf4; DAP4; MYL9; TGIF2; C20orf24; SLA2; NDRG3; C20orf172; FLJ44670; KIAA0889; 343574; SAMHD1; RBL1; C20orf132; RPN2; GHRH; MANBAL; SRC |
| 25781 | KIAA0889 | 20 | 34848822 | 20q11.23 | gneg 8; gpos 1; | -1.35 | 0.297335562 | 0.147 | -0.198 | 0.373 | 0.364 | EPB41L1; C20orf4; DAP4; MYL9; TGIF2; C20orf24; SLA2; NDRG3; C20orf172; FLJ44670; KIAA0889; 343574; SAMHD1; RBL1; C20orf132; RPN2; GHRH; MANBAL; SRC; BLCAP; NNAT |
| 5933 | RBL1 | 20 | 35065390 | 20q11.2 | gneg 7; gpos 2; | 1.05 | 0.167486722 | 0.004 | -0.259 | 0.374 | 0.475 | MYL9; TGIF2; C20orf24; SLA2; NDRG3; C20orf172; FLJ44670; KIAA0889; 343574; SAMHD1; RBL1; C20orf132; RPN2; GHRH; MANBAL; SRC; BLCAP; NNAT; 400844; CTNNBL1; C20orf102 |
| 63905 | MANBAL | 20 | 35351464 | 20q11.23-q12 | gneg 4; gpos 4; | -1.32 | 0.002625009 | 0.01 | -0.503 | 0.444 | 0.922 | NDRG3; C20orf172; FLJ44670; KIAA0889; 343574; SAMHD1; RBL1; C20orf132; RPN2; GHRH; MANBAL; SRC; BLCAP; NNAT; 400844; CTNNBL1; C20orf102; KIAA0406; C20orf77; TGM2; BPI |
| 6714 | SRC | 20 | 35406501 | 20q12-q13 | gneg 4; gpos 4; | 1.18 | 0.949679903 | 0.045 | -0.012 | 0.313 | 0.022 | C20orf172; FLJ44670; KIAA0889; 343574; SAMHD1; RBL1; C20orf132; RPN2; GHRH; MANBAL; SRC; BLCAP; NNAT; 400844; CTNNBL1; C20orf102; KIAA0406; C20orf77; TGM2; BPI; LBP |
| 10904 | BLCAP | 20 | 35579233 | 20q11.2-q12 | gneg 3; gpos 5; | 1.11 | 0.154435918 | 0.017 | -0.267 | 0.308 | 0.489 | FLJ44670; KIAA0889; 343574; SAMHD1; RBL1; C20orf132; RPN2; GHRH; MANBAL; SRC; BLCAP; NNAT; 400844; CTNNBL1; C20orf102; KIAA0406; C20orf77; TGM2; BPI; LBP; LOC128439 |
| 56259 | CTNNBL1 | 20 | 35755847 | 20q11.23-q12 | gpos 8; | -1.21 | 0.084889607 | 0.113 | -0.318 | 0.378 | 0.582 | SAMHD1; RBL1; C20orf132; RPN2; GHRH; MANBAL; SRC; BLCAP; NNAT; 400844; CTNNBL1; C20orf102; KIAA0406; C20orf77; TGM2; BPI; LBP; LOC128439; KIAA1219; MGC39724; 343578 |
| 128434 | C20orf102 | 20 | 35964919 | 20q12 | gneg 1; gpos 8; | -1.59 | 0.037986624 | 0.063 | -0.374 | 0.381 | 0.686 | RBL1; C20orf132; RPN2; GHRH; MANBAL; SRC; BLCAP; NNAT; 400844; CTNNBL1; C20orf102; KIAA0406; C20orf77; TGM2; BPI; LBP; LOC128439; KIAA1219; MGC39724; 343578; VIAAT |
| 7052 | TGM2 | 20 | 36190279 | 20q12 | gneg 1; gpos 9; | -3.08 | 2.56E-07 | -0.127 | -0.676 | 0.372 | 1.24 | GHRH; MANBAL; SRC; BLCAP; NNAT; 400844; CTNNBL1; C20orf102; KIAA0406; C20orf77; TGM2; BPI; LBP; LOC128439; KIAA1219; MGC39724; 343578; VIAAT; FLJ12785; PPP1R16B; C20orf129 |
| 63935 | C20orf67 | 20 | 43996723 | 20q13.12 | gpos 11; | 1.22 | 0.8617047 | -0.087 | -0.034 | 0.369 | 0.062 | UBE2C; TNNC2; C20orf161; PTE1; ZSWIM3; ZSWIM1; C20orf165; C20orf163; PPGB; PLTP; C20orf67; ZNF335; MMP9; SLC12A5; NCOA5; TNFRSF5; CDH22; SLC35C2; ELMO2; DKFZp547G0215; ZNF334 |
| 63925 | ZNF335 | 20 | 44010699 | 20q11.21-q13.12 | gpos 10; | 1.05 | 0.430916996 | 0.045 | -0.151 | 0.369 | 0.277 | TNNC2; C20orf161; PTE1; ZSWIM3; ZSWIM1; C20orf165; C20orf163; PPGB; PLTP; C20orf67; ZNF335; MMP9; SLC12A5; NCOA5; TNFRSF5; CDH22; SLC35C2; ELMO2; DKFZp547G0215; ZNF334; SLC13A3 |
| 57727 | NCOA5 | 20 | 44123042 | 20q12-q13.12 | gpos 9; | 1.18 | 0.596864852 | -0.086 | -0.102 | 0.331 | 0.187 | ZSWIM3; ZSWIM1; C20orf165; C20orf163; PPGB; PLTP; C20orf67; ZNF335; MMP9; SLC12A5; NCOA5; TNFRSF5; CDH22; SLC35C2; ELMO2; DKFZp547G0215; ZNF334; SLC13A3; C20orf64; SLC2A10; EYA2 |
| 23626 | SPO11 | 20 | 55338237 | 20q13.2-q13.3 | gneg 10; gpos 4; | 1.35 | 0.444051126 | -0.129 | 0.147 | 0.307 | -0.269 | CBLNL1; MC3R; C20orf108; STK6; CSTF1; C20orf32; C20orf43; TFAP2C; 339583; BMP7; SPO11; RAE1; RNPC1; CTCFL; PCK1; ZBP1; TMEPAI; C20orf85; C20orf86; RAB22A; VAPB |
| 8480 | RAE1 | 20 | 55359723 | 20q13.31 | gneg 10; gpos 5; | 1.26 | 0.520171923 | 0 | 0.124 | 0.327 | -0.227 | MC3R; C20orf108; STK6; CSTF1; C20orf32; C20orf43; TFAP2C; 339583; BMP7; SPO11; RAE1; RNPC1; CTCFL; PCK1; ZBP1; TMEPAI; C20orf85; C20orf86; RAB22A; VAPB; FLJ90166 |
| 337971 | KRTAP19-4 | 21 | 30791044 | 21q22.1 |  | -1 | 0.029379191 | 0.227 | -0.39 | 0.35 | 0.715 | 388818; KRTAP23-1; KRTAP13-2; KRTAP13-1; KRTAP13-3; KRTAP13-4; KRTAP15-1; KRTAP19-1; KRTAP19-2; KRTAP19-3; KRTAP19-4; KRTAP19-5; KRTAP19-6; KRTAP19-7; KRTAP6-3; KRTAP6-2; KRTAP22-1; KRTAP6-1; KRTAP20-1; KRTAP20-2; KRTAP21-2 |
| 337972 | KRTAP19-5 | 21 | 30796060 | 21q22.1 |  | -1 | 0.031411194 | 0.013 | -0.386 | 0.348 | 0.707 | KRTAP23-1; KRTAP13-2; KRTAP13-1; KRTAP13-3; KRTAP13-4; KRTAP15-1; KRTAP19-1; KRTAP19-2; KRTAP19-3; KRTAP19-4; KRTAP19-5; KRTAP19-6; KRTAP19-7; KRTAP6-3; KRTAP6-2; KRTAP22-1; KRTAP6-1; KRTAP20-1; KRTAP20-2; KRTAP21-2; KRTAP21-1 |
| 337966 | KRTAP6-1 | 21 | 30907875 | 21q22.1 | gneg 3; | 1.85 | 0.868728331 | 0.076 | 0.032 | 0.346 | -0.059 | KRTAP19-1; KRTAP19-2; KRTAP19-3; KRTAP19-4; KRTAP19-5; KRTAP19-6; KRTAP19-7; KRTAP6-3; KRTAP6-2; KRTAP22-1; KRTAP6-1; KRTAP20-1; KRTAP20-2; KRTAP21-2; KRTAP21-1; KRTAP8-1; KRTAP11-1; TIAM1; 150051; SOD1; SFRS15 |
| 64976 | MRPL40 | 22 | 17794589 | 22q11.21 | gneg 18; | -1.13 | 0.049860763 | -0.199 | -0.356 | 0.339 | 0.653 | USP18; DGCR6; PRODH; DGCR2; STK22B; DGCR14; GSCL; SLC25A1; CLTCL1; HIRA; MRPL40; LOC128977; UFD1L; CDC45L; CLDN5; PNUTL1; GP1BB; TBX1; GNB1L; FLJ21125; TXNRD2 |
| 7332 | UBE2L3 | 22 | 20246510 | 22q11.21 | gneg 8; gpos 8; | 1 | 0.064511512 | 0.058 | -0.338 | 0.324 | 0.62 | 400890; FLJ30473; LZTR1; THAP7; MGC16703; P2RXL1; SLC7A4; 284861; HIC2; LOC220686; UBE2L3; FLJ36046; SDF2L1; PPIL2; YPEL1; MAPK1; PPM1F; TOP3B; LOC91219; VPREB1; LOC96610 |
| 6634 | SNRPD3 | 22 | 23276171 | 22q11.23 | gneg 12; gpos 3; | 1.92 | 0.251455482 | -0.128 | 0.217 | 0.312 | -0.399 | DDT; HS322B1A; GSTT1; CABIN1; GGTLA1; DKFZP434P211; 388847; KIAA0376; ADORA2A; UPB1; SNRPD3; GGT1; 255349; CRYBB3; CRYBB2; 400920; DKFZp434O0213; 400921; ADRBK2; MYO18B; SEZ6L |
| 1417 | CRYBB3 | 22 | 23920378 | 22q11.23 | gneg 10; gpos 5; | 1.23 | 0.784109214 | 0.112 | -0.053 | 0.341 | 0.097 | CABIN1; GGTLA1; DKFZP434P211; 388847; KIAA0376; ADORA2A; UPB1; SNRPD3; GGT1; 255349; CRYBB3; CRYBB2; 400920; DKFZp434O0213; 400921; ADRBK2; MYO18B; SEZ6L; LOC57168; HPS4; TFIP11 |
| 1415 | CRYBB2 | 22 | 23941938 | 22q11.23 | gneg 9; gpos 6; | 1.27 | 0.651443428 | 0.049 | -0.087 | 0.387 | 0.16 | GGTLA1; DKFZP434P211; 388847; KIAA0376; ADORA2A; UPB1; SNRPD3; GGT1; 255349; CRYBB3; CRYBB2; 400920; DKFZp434O0213; 400921; ADRBK2; MYO18B; SEZ6L; LOC57168; HPS4; TFIP11; TPST2 |
| 157 | ADRBK2 | 22 | 24285499 | 22q12.1 | gneg 7; gpos 9; | 2.52 | 0.056419878 | 0.026 | 0.348 | 0.419 | -0.638 | ADORA2A; UPB1; SNRPD3; GGT1; 255349; CRYBB3; CRYBB2; 400920; DKFZp434O0213; 400921; ADRBK2; MYO18B; SEZ6L; LOC57168; HPS4; TFIP11; TPST2; CRYBB1; CRYBA4; MN1; PITPNB |
| 23544 | SEZ6L | 22 | 24890189 | 22q12.1 | gneg 7; gpos 10; | 1.13 | 0.311628537 | -0.08 | -0.193 | 0.408 | 0.353 | SNRPD3; GGT1; 255349; CRYBB3; CRYBB2; 400920; DKFZp434O0213; 400921; ADRBK2; MYO18B; SEZ6L; LOC57168; HPS4; TFIP11; TPST2; CRYBB1; CRYBA4; MN1; PITPNB; KIAA1043; 284901 |
| 57168 | LOC57168 | 22 | 25149855 | 22q12.1 | gneg 6; gpos 11; | 2.7 | 0.013194317 | -0.003 | 0.434 | 0.354 | -0.795 | GGT1; 255349; CRYBB3; CRYBB2; 400920; DKFZp434O0213; 400921; ADRBK2; MYO18B; SEZ6L; LOC57168; HPS4; TFIP11; TPST2; CRYBB1; CRYBA4; MN1; PITPNB; KIAA1043; 284901; CHEK2 |
| 3162 | HMOX1 | 22 | 34101640 | 22q13.1 | gneg 13; gpos 4; | 1.29 | 0.544515305 | 0.006 | -0.117 | 0.326 | 0.214 | HSPC117; BPIL2; FBXO7; SYN3; TIMP3; LARGE; LOC91464; HMG2L1; 388897; TOM1; HMOX1; MCM5; RASD2; MB; 284912; APOL6; APOL5; RBM9; APOL3; APOL2; APOL1 |
| 84844 | PHF5A | 22 | 40180221 | 22q13.2 | gneg 1; gpos 11; | 1.89 | 0.028178331 | -0.142 | 0.392 | 0.337 | -0.719 | LOC63929; DNAJB7; RBX1; EP300; L3MBTL2; LOC150356; RANGAP1; RoXaN; TEF; TOB2; PHF5A; ACO2; RPC8; PIPPIN; PMM1; NHP2L1; FLJ23584; FLJ22349; SREBF2; TNFRSF13C; C22orf18 |
| 4809 | NHP2L1 | 22 | 40394437 | 22q13.2-q13.31 | gneg 4; gpos 9; | 1.3 | 0.991561848 | -0.113 | 0.002 | 0.312 | -0.004 | LOC150356; RANGAP1; RoXaN; TEF; TOB2; PHF5A; ACO2; RPC8; PIPPIN; PMM1; NHP2L1; FLJ23584; FLJ22349; SREBF2; TNFRSF13C; C22orf18; SEPT3; MGC26816; NAGA; LOC150368; LOC91689 |
| 79019 | C22orf18 | 22 | 40659241 | 22q13.31 | gneg 7; gpos 6; | 1.76 | 0.011870622 | 0.04 | 0.439 | 0.332 | -0.805 | PHF5A; ACO2; RPC8; PIPPIN; PMM1; NHP2L1; FLJ23584; FLJ22349; SREBF2; TNFRSF13C; C22orf18; SEPT3; MGC26816; NAGA; LOC150368; LOC91689; NDUFA6; CYP2D6; TCF20; 150371; NFAM1 |
| 164684 | MGC26816 | 22 | 40719291 | 22q13.31 | gneg 7; gpos 5; | -1.31 | 0.017417596 | -0.082 | -0.419 | 0.405 | 0.769 | RPC8; PIPPIN; PMM1; NHP2L1; FLJ23584; FLJ22349; SREBF2; TNFRSF13C; C22orf18; SEPT3; MGC26816; NAGA; LOC150368; LOC91689; NDUFA6; CYP2D6; TCF20; 150371; NFAM1; SERHL; CGI-96 |
| 150368 | LOC150368 | 22 | 40794786 | 22q13.31 | gneg 8; gpos 4; | 1.09 | 0.659979197 | -0.003 | -0.085 | 0.309 | 0.156 | PMM1; NHP2L1; FLJ23584; FLJ22349; SREBF2; TNFRSF13C; C22orf18; SEPT3; MGC26816; NAGA; LOC150368; LOC91689; NDUFA6; CYP2D6; TCF20; 150371; NFAM1; SERHL; CGI-96; PDIP46; DIA1 |
| 94056 | SYAP1 | X | 16497411 | Xp22.22 | gneg 3; gpos 6; | -1.95 | 0.012398212 | -0.001 | -0.437 | 0.305 | 0.801 | PIR; BMX; ACE2; NX17; CA5B; U2AF1L2; AP1S2; GRPR; CTPS2; CALB3; SYAP1; CXorf15; RBBP7; REPS2; NHS; SCML1; RAI2; MGC33653; SCML2; CDKL5; RS1 |
| 6611 | SMS | X | 21718498 | Xp22.1 | gneg 12; | 2.16 | 0.004401567 | 0.203 | 0.483 | 0.423 | -0.886 | PDHA1; SH3KBP1; LOC256643; FLJ14503; EIF1A; RPS6KA3; CNK2; FLJ34960; SMPX; MBTPS2; SMS; PHEX; FLJ25735; DDX53; FLJ30296; PRDX4; ACATE2; SAT; MGC4825; FLJ25444; EIF2S3 |
| 139411 | FLJ30296 | X | 23112751 | Xp22.13 | gneg 13; gpos 1; | 8.68 | 0.087085906 | 0.235 | 0.316 | 0.33 | -0.579 | EIF1A; RPS6KA3; CNK2; FLJ34960; SMPX; MBTPS2; SMS; PHEX; FLJ25735; DDX53; FLJ30296; PRDX4; ACATE2; SAT; MGC4825; FLJ25444; EIF2S3; ZFX; 170067; PDK3; PCYT1B |
| 10549 | PRDX4 | X | 23445301 | Xp22.13 | gneg 12; gpos 1; | 1.06 | 0.215185299 | 0.181 | -0.234 | 0.34 | 0.429 | RPS6KA3; CNK2; FLJ34960; SMPX; MBTPS2; SMS; PHEX; FLJ25735; DDX53; FLJ30296; PRDX4; ACATE2; SAT; MGC4825; FLJ25444; EIF2S3; ZFX; 170067; PDK3; PCYT1B; POLA |
| 6303 | SAT | X | 23560960 | Xp22.1 | gneg 11; gpos 2; | -1.19 | 0.136187188 | -0.04 | -0.278 | 0.357 | 0.51 | FLJ34960; SMPX; MBTPS2; SMS; PHEX; FLJ25735; DDX53; FLJ30296; PRDX4; ACATE2; SAT; MGC4825; FLJ25444; EIF2S3; ZFX; 170067; PDK3; PCYT1B; POLA; ARX; MGC33889 |
| 79135 | MGC4825 | X | 23611132 | Xp22.13 | gneg 10; gpos 3; | 1.71 | 0.051228043 | 0.115 | 0.354 | 0.335 | -0.65 | SMPX; MBTPS2; SMS; PHEX; FLJ25735; DDX53; FLJ30296; PRDX4; ACATE2; SAT; MGC4825; FLJ25444; EIF2S3; ZFX; 170067; PDK3; PCYT1B; POLA; ARX; MGC33889; MAGEB6 |
| 254158 | FLJ25444 | X | 23685779 | Xp22.13 | gneg 10; gpos 4; | 3.34 | 0.437636419 | 0.069 | 0.149 | 0.357 | -0.273 | MBTPS2; SMS; PHEX; FLJ25735; DDX53; FLJ30296; PRDX4; ACATE2; SAT; MGC4825; FLJ25444; EIF2S3; ZFX; 170067; PDK3; PCYT1B; POLA; ARX; MGC33889; MAGEB6; 347541 |
| 7712 | ZNF157 | X | 46986235 | Xp11.2 | gneg 6; gpos 2; | 4.02 | 0.233547695 | 0.078 | 0.225 | 0.311 | -0.413 | CHST7; SLC9A7; RP2; PHF16; RGN; RBM10; UBE1; INE1; PCTK1; USP11; ZNF157; ZNF41; ARAF1; SYN1; TIMP1; PFC; ELK1; UXT; 389852; SSX6; SSX5 |
| 6839 | SUV39H1 | X | 48311380 | Xp11.23 | gneg 17; | 1.39 | 0.754057422 | 0.013 | 0.061 | 0.322 | -0.111 | SSX9; SSX3; SSX4; SLC38A5; FTSJ1; PPN; EBP; RBM3; WDR13; WAS; SUV39H1; GATA1; HDAC6; ERAS; PCSK1N; TIMM17B; PQBP1; SLC35A2; PIM2; DKFZp761A052; KCND1 |
| 10245 | TIMM17B | X | 48506979 | Xp11.23 | gneg 16; gpos 1; | -1.12 | 0.034734594 | -0.181 | -0.38 | 0.359 | 0.696 | PPN; EBP; RBM3; WDR13; WAS; SUV39H1; GATA1; HDAC6; ERAS; PCSK1N; TIMM17B; PQBP1; SLC35A2; PIM2; DKFZp761A052; KCND1; TFE3; JM11; JM4; JM5; T54 |
| 10084 | PQBP1 | X | 48512040 | Xp11.23 | gneg 16; gpos 1; | -1.11 | 0.120492012 | -0.058 | -0.289 | 0.434 | 0.53 | EBP; RBM3; WDR13; WAS; SUV39H1; GATA1; HDAC6; ERAS; PCSK1N; TIMM17B; PQBP1; SLC35A2; PIM2; DKFZp761A052; KCND1; TFE3; JM11; JM4; JM5; T54; FLJ21687 |
| 7355 | SLC35A2 | X | 48516706 | Xp11.23-p11.22 | gneg 17; gpos 1; | 1.23 | 0.548534076 | -0.138 | -0.116 | 0.335 | 0.212 | RBM3; WDR13; WAS; SUV39H1; GATA1; HDAC6; ERAS; PCSK1N; TIMM17B; PQBP1; SLC35A2; PIM2; DKFZp761A052; KCND1; TFE3; JM11; JM4; JM5; T54; FLJ21687; PLP2 |
| 3750 | KCND1 | X | 48574886 | Xp11.23 | gneg 17; gpos 1; | 1.13 | 0.286718677 | 0.135 | -0.203 | 0.332 | 0.371 | SUV39H1; GATA1; HDAC6; ERAS; PCSK1N; TIMM17B; PQBP1; SLC35A2; PIM2; DKFZp761A052; KCND1; TFE3; JM11; JM4; JM5; T54; FLJ21687; PLP2; LMO6; SYP; CACNA1F |
| 7030 | TFE3 | X | 48642489 | Xp11.22 | gneg 17; gpos 1; | 1.46 | 0.846203471 | -0.007 | 0.038 | 0.305 | -0.069 | GATA1; HDAC6; ERAS; PCSK1N; TIMM17B; PQBP1; SLC35A2; PIM2; DKFZp761A052; KCND1; TFE3; JM11; JM4; JM5; T54; FLJ21687; PLP2; LMO6; SYP; CACNA1F; JM1 |
| 11152 | JM5 | X | 48688548 | Xp11.23 | gneg 15; gpos 1; | -1.32 | 0.001903955 | 0.031 | -0.514 | 0.381 | 0.943 | PCSK1N; TIMM17B; PQBP1; SLC35A2; PIM2; DKFZp761A052; KCND1; TFE3; JM11; JM4; JM5; T54; FLJ21687; PLP2; LMO6; SYP; CACNA1F; JM1; FOXP3; GAGE4; GAGE6 |
| 56271 | FLJ10097 | X | 102276201 | Xq22.1-q22.3 | gneg 6; gpos 7; | 1.26 | 0.61246989 | 0.251 | 0.098 | 0.437 | -0.179 | KIAA1789; my048; LOC340542; NXF2; TMSNB; FLJ12969; LOC114928; KIAA1701; BEX1; NXF3; FLJ10097; MGC45400; MGC23947; LOC51186; NGFRAP1; RAB40A; FLJ21174; MGC15737; TCEAL1; MORF4L2; MGC39655 |
| 90843 | MGC45400 | X | 102314070 | Xq22.2 | gneg 6; gpos 7; | 1.17 | 0.966299805 | 0.34 | -0.008 | 0.469 | 0.015 | my048; LOC340542; NXF2; TMSNB; FLJ12969; LOC114928; KIAA1701; BEX1; NXF3; FLJ10097; MGC45400; MGC23947; LOC51186; NGFRAP1; RAB40A; FLJ21174; MGC15737; TCEAL1; MORF4L2; MGC39655; PLP1 |
| 56849 | MGC23947 | X | 102391309 | Xq22.1 | gneg 6; gpos 7; | 1.14 | 0.736553572 | 0.116 | 0.065 | 0.407 | -0.119 | LOC340542; NXF2; TMSNB; FLJ12969; LOC114928; KIAA1701; BEX1; NXF3; FLJ10097; MGC45400; MGC23947; LOC51186; NGFRAP1; RAB40A; FLJ21174; MGC15737; TCEAL1; MORF4L2; MGC39655; PLP1; RAB9B |
| 51186 | LOC51186 | X | 102417567 | Xq22.2 | gneg 5; gpos 8; | -1.17 | 0.244114356 | 0.278 | -0.221 | 0.517 | 0.404 | NXF2; TMSNB; FLJ12969; LOC114928; KIAA1701; BEX1; NXF3; FLJ10097; MGC45400; MGC23947; LOC51186; NGFRAP1; RAB40A; FLJ21174; MGC15737; TCEAL1; MORF4L2; MGC39655; PLP1; RAB9B; MGC39900 |
| 27018 | NGFRAP1 | X | 102437412 | Xq22.2 | gneg 4; gpos 9; | 1.21 | 0.520279966 | 0.418 | 0.124 | 0.523 | -0.227 | TMSNB; FLJ12969; LOC114928; KIAA1701; BEX1; NXF3; FLJ10097; MGC45400; MGC23947; LOC51186; NGFRAP1; RAB40A; FLJ21174; MGC15737; TCEAL1; MORF4L2; MGC39655; PLP1; RAB9B; MGC39900; LOC158983 |
| 9338 | TCEAL1 | X | 102690046 | Xq22.1 | gneg 4; gpos 10; | 1.24 | 0.668083248 | 0.337 | 0.083 | 0.359 | -0.152 | BEX1; NXF3; FLJ10097; MGC45400; MGC23947; LOC51186; NGFRAP1; RAB40A; FLJ21174; MGC15737; TCEAL1; MORF4L2; MGC39655; PLP1; RAB9B; MGC39900; LOC158983; LOC286436; ESX1L; IL1RAPL2; TEX13A |
| 90161 | HS6ST2 | X | 131485578 | Xq26.2 | gneg 8; gpos 10; | 3.91 | 0.119463428 | -0.08 | 0.29 | 0.331 | -0.531 | CGI-79; COVA1; FLJ30058; IGSF1; 347468; LOC286467; MST4; LOC90167; RAP2C; MBNL3; HS6ST2; USP26; LOC51270; GPC4; GPC3; 347475; PHF6; HPRT1; PLAC1; LOC159090; LOC159091 |
| 2239 | GPC4 | X | 132160583 | Xq26.1 | gneg 8; gpos 10; | 6.06 | 0.001620152 | 0.24 | 0.52 | 0.422 | -0.953 | IGSF1; 347468; LOC286467; MST4; LOC90167; RAP2C; MBNL3; HS6ST2; USP26; LOC51270; GPC4; GPC3; 347475; PHF6; HPRT1; PLAC1; LOC159090; LOC159091; DJ473B4; CXX1; DKFZP564B147 |
| 2719 | GPC3 | X | 132395297 | Xq26.1 | gneg 9; gpos 9; | -1.74 | 0.08921569 | 0.178 | -0.314 | 0.303 | 0.575 | 347468; LOC286467; MST4; LOC90167; RAP2C; MBNL3; HS6ST2; USP26; LOC51270; GPC4; GPC3; 347475; PHF6; HPRT1; PLAC1; LOC159090; LOC159091; DJ473B4; CXX1; DKFZP564B147; FLJ20527 |
| 84295 | PHF6 | X | 133232861 | Xq26 | gneg 11; gpos 7; | 3.02 | 0.003402127 | 0.167 | 0.493 | 0.328 | -0.904 | MST4; LOC90167; RAP2C; MBNL3; HS6ST2; USP26; LOC51270; GPC4; GPC3; 347475; PHF6; HPRT1; PLAC1; LOC159090; LOC159091; DJ473B4; CXX1; DKFZP564B147; FLJ20527; ZNF75; FLJ23614 |
| 3251 | HPRT1 | X | 133319776 | Xq26.1 | gneg 11; gpos 6; | 2.77 | 0.0012249 | 0.136 | 0.529 | 0.391 | -0.97 | LOC90167; RAP2C; MBNL3; HS6ST2; USP26; LOC51270; GPC4; GPC3; 347475; PHF6; HPRT1; PLAC1; LOC159090; LOC159091; DJ473B4; CXX1; DKFZP564B147; FLJ20527; ZNF75; FLJ23614; 399668 |
| 159090 | LOC159090 | X | 133629116 | Xq26.3 | gneg 13; gpos 4; | 2.6 | 0.021254903 | 0.251 | 0.409 | 0.316 | -0.749 | MBNL3; HS6ST2; USP26; LOC51270; GPC4; GPC3; 347475; PHF6; HPRT1; PLAC1; LOC159090; LOC159091; DJ473B4; CXX1; DKFZP564B147; FLJ20527; ZNF75; FLJ23614; 399668; LOC203522; MGC27005 |
| 9087 | TMSB4Y | Y | 14253577 | Yq11.221 | gneg 12; gpos 5; | 1.24 | 0.987282541 | 0.176 | 0.003 | 0.356 | -0.006 | TGIF2LY; PCDH11Y; TSPYQ1; AMELY; TBL1Y; PRKY; TSPY; USP9Y; DDX3Y; UTY; TMSB4Y; VCY; NLGN4Y; XKRY; CDY2; FLJ25453; CD24; FLJ39821; CYorf15B; SMCY; EIF1AY |
